# Pd/BIPHEPHOS is an Efficient Catalyst for the Pd‐Catalyzed *S*‐Allylation of Thiols with High *n*‐Selectivity

**DOI:** 10.1002/adsc.201901250

**Published:** 2019-11-07

**Authors:** Thomas Schlatzer, Hilmar Schröder, Melanie Trobe, Christian Lembacher‐Fadum, Simon Stangl, Christoph Schlögl, Hansjörg Weber, Rolf Breinbauer

**Affiliations:** ^1^ Institute of Organic Chemistry Graz University of Technology Stremayrgasse 9 A-8010 Graz Austria

**Keywords:** crossover, isofunctional reaction, isomerization, thioether, Tsuji-Trost allylation

## Abstract

The Pd‐catalyzed *S*‐allylation of thiols with stable allylcarbonate and allylacetate reagents offers several advantages over established reactions for the formation of thioethers. We could demonstrate that Pd/BIPHEPHOS is a catalyst system which allows the transition metal‐catalyzed *S*‐allylation of thiols with excellent *n*‐regioselectivity. Mechanistic studies showed that this reaction is reversible under the applied reaction conditions. The excellent functional group tolerance of this transformation was demonstrated with a broad variety of thiol nucleophiles (18 examples) and allyl substrates (9 examples), and could even be applied for the late‐stage diversification of cephalosporins, which might find application in the synthesis of new antibiotics.

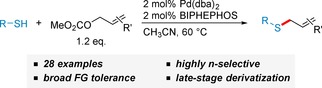

Transition‐metal catalyzed allylation reactions have received considerable attention over the last few decades, especially for the alkylation of *C*‐, *N*‐ and *O*‐nucleophiles. In particular, the Pd‐catalyzed allylation (“Tsuji‐Trost‐allylation”) has become a precious tool in organic synthesis,^1^ which has shown its value even in the total synthesis of natural products^2^ and pharmaceuticals.^3^ Moreover, highly enantioselective variants using chiral ligands allow full control of the absolute stereoconfiguration of the synthesized products.^4^ Other metals such as Ir,^5^ Fe,^6^ and Rh^7^ have been demonstrated to serve as effective catalysts for this transformation, showing different regioselectivity. In contrast to the widespread use of this transformation in organic synthesis the transition‐metal catalyzed allylation of *S*‐nucleophiles has been rarely studied, and only a few examples have been reported.^8^ The limited use of Pd‐catalyzed *S*‐allylation can be associated with two intrinsic difficulties: a) *S*‐nucleophiles can also function as efficient ligands for Pd and poison the catalyst, and b) thiols are easily oxidized and the reactions have to be carried out under exclusion of air. We thus set out to identify a suitable catalyst system, which would enable the efficient *S*‐allylation of thiol nucleophiles. As we did not only envision its application for the synthesis of small molecules but also intended its use for the post‐translational modification of Cys‐containing peptides and proteins,^9^ we were aiming for a catalyst which would favour the unbranched (“linear”) *n*‐isomer.

At the outset of our studies on Pd‐catalyzed *S*‐allylation we performed an extensive reaction optimization on a model reaction with 1‐octanethiol (**1** **a**) and methyl prenyl carbonate (**2** **a**) as representative starting materials (Table 1). Initially we screened 50 bidentate, phosphorous‐based ligands (Table S1) in combination with Pd(dba)_2_ as catalyst precursor and monitored both conversion and regioselectivity (*n*/*i* ratio) by GC‐MS. In particular we were interested if we could observe any trends regarding the influence of the natural bite angle and the electronic properties of the ligands on the reaction outcome.^10^ Phosphine ligands with large bite angles (Table 1, entries 1–3), that are frequently used in Tsuji‐Trost allylations, did not lead to any conversion of **1** **a**. In contrast, bidentate phosphines with mid‐sized bite angles (Table 1, entries 4–8) exhibited significantly higher conversions. Within this group, electron rich alkyl phosphines (Table 1, entries 5 & 8) were inferior to their corresponding aryl phosphines, indicating that electron poor ligands could be beneficial for this reaction. Moreover, structurally more rigid ligands (Table 1, entries 6–8) were found to be superior. The dppf ligand (Table 1, entry 7) meets these criteria and was found to give full conversion of **1** **a** and acceptable regioselectivity (*n*/*i*=92/8). Based on this parent ligand, we tested members of the Josiphos, Mandyphos and Walphos ligand families (Table 1, entries 9–11), which unfortunately did not result in any increased regioselectivity. To our delight, we were instead able to identify the electron‐deficient bidentate phosphite BIPHEPHOS (Table 1, entry 12) as our ligand of choice, affording the thioether **3** **aa** with full conversion and complete *n*‐selectivity.

**Table 1:** Reaction optimization of the Pd‐catalyzed *S*‐allylation.

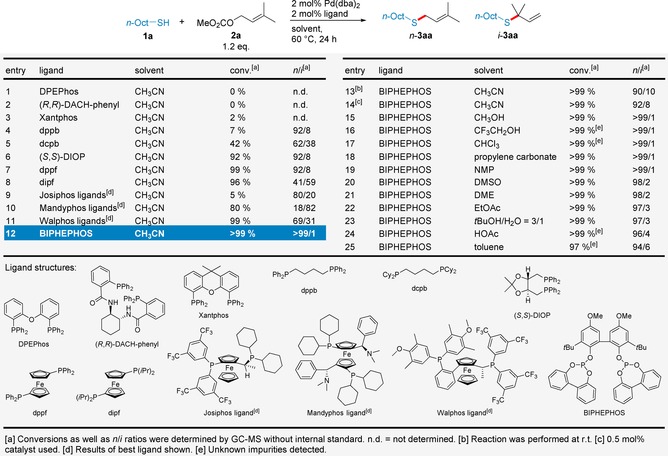

We next conducted optimization studies focusing on the amount of catalyst as well as the reaction temperature. The most suitable operating window of our catalyst system was determined to be 2.0 mol% PdL and 60 °C (Table S2 and S3). Decreased catalyst concentration or temperature (Table 1, entries 13 & 14) led to lower regioselectivity at full conversion.

Finally, we were interested to see if other solvents than acetonitrile can be used in the Pd‐catalyzed *S*‐allylation. Indeed, we could identify several solvents that are suitable alternatives without affecting conversion or selectivity of the reaction, encompassing polar aprotic substitutes such as propylene carbonate, NMP, DMSO, DME and EtOAc (Table 1, entries 18–22) but also rather apolar CHCl_3_ or toluene (Table 1, entries 17 & 25). Moreover, reactions in polar protic solvents such as MeOH (Table 1, entry 15) and even in fairly acidic CF_3_CH_2_OH and HOAc (Table 1, entries 16 & 24) proved to be successful. Importantly, aqueous solvent mixtures (Table 1, entry 23) were also tolerated by our catalyst system.^9^ It is noteworthy that these solvents cover an extremely broad range of polarities, which should enable an easy adjustment of our methodology to any thiol substrate addressing solubility issues. In total 31 solvents were considered in this screening (Table S4).

With our optimized conditions (Table 1, entry 12) in hand we explored the scope of this system using more elaborated thiol substrates and allylation reagents. In order to assess the effect of the nucleophilicity on the reaction yields, we subjected a series of substituted aromatic thiols (Scheme 1, entries **3** **ba**–**3** **ka**) to *S*‐allylation. To our delight, the reaction yields were only slightly affected by electron‐donating (Me, OMe) or ‐withdrawing groups (Cl, F, CF_3_, CO_2_H), and were consistently high (in most cases >80%). Sterically hindered *ortho*‐disubstituted **3** **da**, as well as substrates bearing potentially interfering nucleophilic groups were fully tolerated and selectively afforded the *S*‐allylated products (**3** **ea**, **3** **ga**, **3** **ka**) in high yield. Encouraged by these results, we tested heterocyclic thiols (Scheme 1, entries **3** **la**–**3** **oa**) that could potentially act as potent ligands for palladium and thereby hamper the catalytic process. Fortunately, also these substrates were smoothly converted to their corresponding thioethers even at a lower reaction temperature of 35 °C, which was crucial to avoid thermal *S*→*N* allyl migration.^11^ Cysteine derivatives were another class of non‐aromatic thiols we were particularly interested in, since these represent highly functionalized molecules that can occur in their *S*‐prenylated form in peptides and proteins in nature.^12^ We thus chose protected cysteine and glutathione as model substrates, which could be readily prenylated (**3** **pa**) and farnesylated (**3** **qc**) in very good yields (>80%), demonstrating the versatility of this catalytic system. Even fairly acidic thiobenzoic acid was converted to the corresponding thioester **3** **ra** although in moderate yield.

**Scheme 1:**
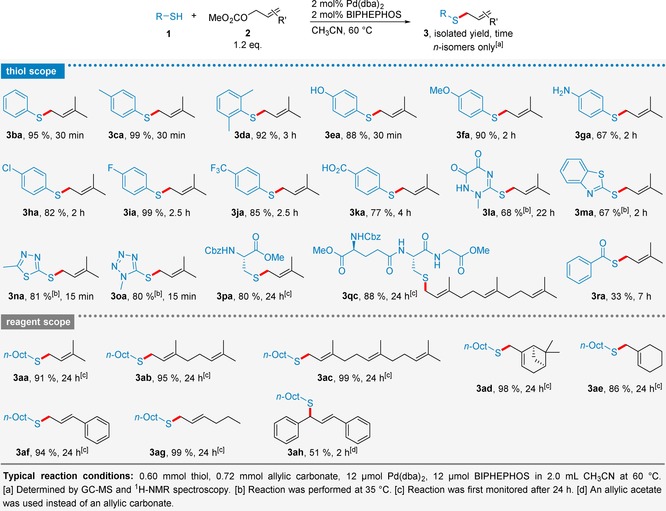
Thiol and reagent scope of the Pd‐catalyzed *S*‐allylation.

In addition, we explored the scope of the allylation reagents, which are rapidly and easily accessible from the corresponding allylic alcohols in a single step and very high yields (see Supporting Information). For this purpose, we subjected various acyclic and more rigid cyclic allylic carbonates to Pd‐catalyzed *S*‐allylation of 1‐octanethiol (**1** **a**) yielding the desired thioethers (Scheme 1, entries **3** **aa**–**3** **ah**) in excellent yields (in most cases >90%). For all employed reagents only the allylic carbonate moiety was activated by the Pd catalyst, while other double bonds remained pristine (i. e. no double bond migration or isomerization was observed).

Importantly, all thioethers of Scheme 1 were isolated in form of their unbranched *n*‐isomer as confirmed by ^1^H‐NMR spectroscopy, highlighting the extraordinary regioselectivity of the Pd/BIPHEPHOS system.

As a next step we were eager to see if this methodology can be applied to the challenging environment of a complex natural product. We noticed that the important class of cephalosporin antibiotics contains an allylic acetate moiety, which could be tested as an electrophile in the Pd‐catalyzed *S*‐allylation. This would be of immediate relevance as several thioether derivatives at this position are in clinical use as antibiotics.^13^ For this purpose, we chose cefalotin, an antibiotic agent, as a representative substrate for late‐stage diversification using several organic thiols (Scheme 2). The corresponding thioethers **5** and **6** were readily isolated after preparative HPLC purification in satisfying yields. To the best of our knowledge, this represents the first Tsuji‐Trost allylation of a cephalosporin at C3’, directly starting from a native substrate. Previous literature precedents^14^ either rely on strong Lewis acids (e. g. BF_3_) and/or require protecting groups.

**Scheme 2:**
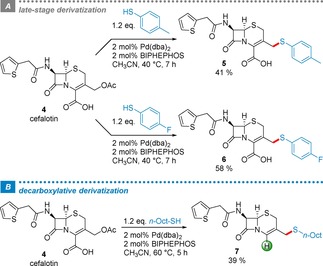
Late‐stage diversification of antibiotic cefalotin via Pd‐catalyzed activation of the allylic acetate moiety under conservation (*A*) or loss (*B*) of the carboxyl group.

Interestingly, if 1‐octanethiol is used as reagent, the resulting product tends to decarboxylate especially at elevated temperature to form **7** in 39% yield.

Finally, we sought to investigate the mechanism of the Pd‐catalyzed *S*‐allylation. First experiments suggested that the unbranched thioether is formed selectively, irrespective of the regiochemistry of the allylic carbonate as indicated by GC‐MS reaction monitoring (Scheme 3A). This corroborates a common π‐allylic intermediate upon Pd‐mediated reagent activation, which is then attacked by the thiol. However, if no thiol was present, the branched allylic carbonate **2** f* was quantitatively isomerized to the unbranched derivative **2** **f** according to ^1^H‐NMR spectroscopy (Scheme 3B).

**Scheme 3:**
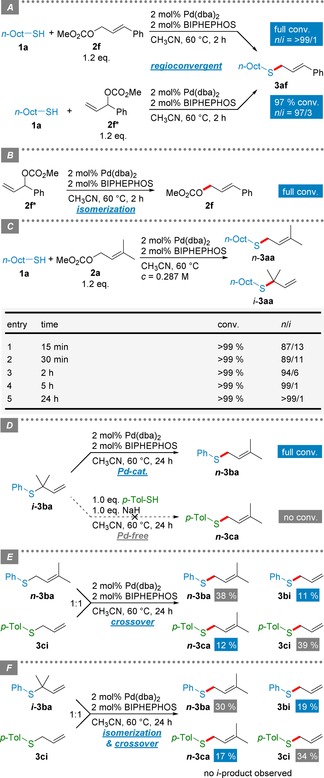
Mechanistic studies of the Pd‐catalyzed *S*‐allylation.

In a further experiment we observed that **1** **a** was completely consumed after only 15 min but only in moderate regioselectivity (*n*/*i*=87/13). The *n*/*i* ratio, however, was found to constantly improve over time to afford the pure *n*‐thioether **3** **aa** after 5 h (Scheme 3C, Table S5) although complete conversion was already reached.^9^ This clearly indicates that the *S*‐allylation is reversible under Pd‐catalyzed conditions. Importantly, the isomerization takes place only in the presence of the Pd catalyst, whereas an intermolecular S_N_2’ attack by thiolate can be excluded (Scheme 3D). Further evidence for the reversibility of this reaction is given by crossover experiments that result in scrambling of the allyl moieties (Scheme 3E,F), indicating that ligand exchange at Pd is possible. The outcome of this successful crossover experiment with thioethers might also imply an opportunity for the scale‐up of this reaction as an isofunctional metathesis reaction,^15^ in which thioether substrates could be a more convenient form of the reagent as the notoriously odorous and oxidation sensitive thiols.

We could establish Pd/BIPHEPHOS as a highly efficient system for the Pd‐catalyzed *S*‐allylation of thiols. The outstanding *n*‐regioselectivity of this system results from a combination of intrinsic ligand properties together with the reversible nature of this reaction. In comparison to established methods for the preparation of thioethers, the Pd/BIPHEPHOS system is distinguished by its very well accessible allylacetate and allylcarbonate substrates, its high functional group tolerance and the mild reaction conditions transferable to many different solvents. These features allow the application of this reaction for the late‐stage diversification of small molecules, as shown for cephalosporin antibiotics in this work, and can even be extended to the modification of peptides and proteins.^9^ The reversible character of this catalytic reaction offers opportunities to be exploited in isofunctional transformations.

## Experimental Section

### General Procedure for the Pd‐Catalyzed *S*‐Allylation

In a flame‐dried and argon‐flushed Schlenk flask, equipped with a Teflon‐coated magnetic stirring bar, Pd(dba)_2_ (12 μmol, 2 mol%) and BIPHEPHOS (12 μmol, 2 mol%) were suspended in anhydrous CH_3_CN (2.0 mL) and stirred in a pre‐heated oil bath at 60 °C for 30 min to obtain a bright yellow solution. Then allylic carbonate (0.72 mmol, 1.2 eq.) and thiol (0.60 mmol, 1 eq.) were added and the resulting mixture was stirred at 60 °C (or lower) until complete consumption of the starting material (according to GC‐MS or TLC). The reaction mixture was cooled to rt and concentrated under reduced pressure. The crude product was purified via flash column chromatography to afford the desired compound.

## Supporting information

As a service to our authors and readers, this journal provides supporting information supplied by the authors. Such materials are peer reviewed and may be re‐organized for online delivery, but are not copy‐edited or typeset. Technical support issues arising from supporting information (other than missing files) should be addressed to the authors.

Supplementary
