# Supplementary material for: Pd/BIPHEPHOS is an Efficient Catalyst for the Pd‐Catalyzed S‐Allylation of Thiols with High n‐Selectivity
Source: Adv Synth Catal. 2019 Nov 7;362(2):331–6. doi: 10.1002/adsc.201901250 (PMC7004212; doi:10.1002/adsc.201901250)
Supplement: Supplementary file 1 — Supplementary [file ADSC-362-331-s001.pdf]

Advanced Synthesis & Catalysis

Supporting Information

© Copyright Wiley-VCH Verlag GmbH & Co. KGaA, 69451 Weinheim, 2020

Pd/BIPHEPHOS is an Efficient Catalyst for the Pd-Catalyzed *S*-Allylation of Thiols with High *n*-Selectivity

Thomas Schlatzer,[+] Hilmar Schröder,[+] Melanie Trobe, Christian Lembacher-Fadum, Simon Stangl, Christoph Schlögl, Hansjörg Weber, and Rolf Breinbauer*© 2019 The Authors. Published by Wiley-VCH Verlag GmbH & Co. KGaA.
This is an open access article under the terms of the Creative Commons Attribution License, which permits use, distribution and reproduction in any medium, provided the original work is properly cited.

Supporting Information

Pd/BIPHEPHOS is an Efficient Catalyst for the Pd-Catalyzed *S*-Allylation of Thiols with High *n*-Selectivity

Thomas Schlatzer,[a] Hilmar Schröder,[a] Melanie Trobe,[a] Christian Lembacher-Fadum,[a] Simon Stangl,[a] Christoph Schlögl,[a] Hansjörg Weber,[a] and Rolf Breinbauer[a],*

[a] Institute of Organic Chemistry, Graz University of Technology, Stremayrgasse 9, A-8010 Graz, Austria

*Correspondence to: breinbauer@tugraz.at

Table of Contents

General Information **5**

Chemicals 5

Thin Layer Chromatography 6

Flash Column Chromatography 6

Gas Chromatography 6

High Performance Liquid Chromatography 6

Semi-Preparative High Performance Liquid Chromatography 7

Nuclear Magnetic Resonance Spectroscopy 7

High Resolution Mass Spectrometry 7

Determination of Melting Points 8

Determination of Optical Rotation 8

Experimental Procedures **9**

Reaction Optimization 9

Ligand Screening 9

Temperature Screening 12

Catalyst Loading Screening 12

Solvent Screening 13

Reversibility of the Pd-Catalyzed Allylation 14

Synthesis of Thiol Substrates 15

L-Cystine 1,1’-dimethyl ester hydrochloride (**S1**) 15

L-Cystine *N*,*N*’-bis[(phenylmethoxy)carbonyl] 1,1’-dimethyl ester (**S2**) 15

Cbz-Cys-OMe (**1p**) 16

Dimethyl 10,10'-(disulfanediylbis(methylene))(5*S*,5'*S*,10*R*,10'*R*)-bis(5-(methoxycarbonyl)-3,8,11-trioxo-1-phenyl-2-oxa-4,9,12-triazatetradecan-14-oate) (**S3**) 17

Cbz-γ-Glu(OMe)-Cys-Gly-OMe (**1q**) 17

Synthesis of Allylic Alcohols 19

Cyclohex-1-en-1-ylmethanol (**S4**) 19

1-Phenylprop-2-en-1-ol (**S5**) 19

Synthesis of Allylic Carbonates 20

General Procedure 20

Methyl (3-methylbut-2-en-1-yl) carbonate (**2a**) 20

(*E*)-3,7-Dimethylocta-2,6-dien-1-yl methyl carbonate (**2b**) 20

((2*E*,6*E*)-3,7,11-Trimethyldodeca-2,6,10-trien-1-yl) methyl carbonate (**2c**) 21

((1*R*,5*S*)-6,6-Dimethylbicyclo[3.1.1]hept-2-en-2-yl)methyl methyl carbonate (**2d**) 21

Cyclohex-1-en-1-ylmethyl methyl carbonate (**2e**) 22
Methyl (*E*)-3-phenyl-2-propenyl carbonate (**2f**) 22
Methyl (1-phenylallyl) carbonate (**2f***) 22
(*E*)-Hex-2-en-1-yl methyl carbonate (**2g**) 23
(*E*)-1,3-Diphenylallyl acetate (**2h**) 23

General Procedure for the Pd-Catalyzed *S*-Allylation 24

Allylic Carbonate Scope 24
(3-Methylbut-2-en-1-yl)(octyl)sulfane (**3aa**) 24
(*E*)-(3,7-Dimethylocta-2,6-dien-1-yl)(octyl)sulfane (**3ab**) 24
((2*E*,6*E*)-3,7,11-Trimethyldodeca-2,6,10-trien-1-yl)(octyl)sulfane (**3ac**) 25
(((1*R*,5*S*)-6,6-Dimethylbicyclo[3.1.1]hept-2-en-2-yl)methyl)(octyl)sulfane (**3ad**) 25
(Cyclohex-1-en-1-ylmethyl)(octyl)sulfane (**3ae**) 26
Cinnamyl(octyl)sulfane (**3af**) 26
(*E*)-Hex-2-en-1-yl(octyl)sulfane (**3ag**) 26
(*E*)-(1,3-Diphenylallyl)(octyl)sulfane (**3ah**) 27

Thiol Scope 27
(3-Methylbut-2-en-1-yl)(phenyl)sulfane (**3ba**) 27
(3-Methylbut-2-en-1-yl)(*p*-tolyl)sulfane (**3ca**) 28
(2,6-Dimethylphenyl)(3-methylbut-2-en-1-yl)sulfane (**3da**) 28
4-((3-Methylbut-2-en-1-yl)thio)phenol (**3ea**) 29
(4-Methoxyphenyl)(3-methylbut-2-en-1-yl)sulfane (**3fa**) 29
4-((3-Methylbut-2-en-1-yl)thio)aniline (**3ga**) 29
(4-Chlorophenyl)(3-methylbut-2-en-1-yl)sulfane (**3ha**) 30
(4-Fluorophenyl)(3-methylbut-2-en-1-yl)sulfane (**3ia**) 30
(3-Methylbut-2-en-1-yl)(4-(trifluoromethyl)phenyl)sulfane (**3ja**) 31
4-((3-Methylbut-2-en-1-yl)thio)benzoic acid (**3ka**) 31
2-Methyl-3-((3-methylbut-2-en-1-yl)thio)-1,2-dihydro-1,2,4-triazine-5,6-dione (**3la**) 32
2-((3-Methylbut-2-en-1-yl)thio)benzo[*d*]thiazole (**3ma**) 32
2-Methyl-5-((3-methylbut-2-en-1-yl)thio)-1,3,4-thiadiazole (**3na**) 33
1-Methyl-5-((3-methylbut-2-en-1-yl)thio)-1*H*-tetrazole (**3oa**) 33
Cbz-Cys(prenyl)-OMe (**3pa**) 34
Cbz-γ-Glu(OMe)-Cys(farnesyl)-Gly-OMe (**3qc**) 34
S-(3-Methylbut-2-en-1-yl)benzothioate (**3ra**) 35
(2-Methylbut-3-en-2-yl)(phenyl)sulfane (***i*-3ba**) 35
Allyl(*p*-tolyl)sulfane (**3ci**) 36

Natural Product *S*-Allylation 37
General Procedure for Cefalotin Derivatization 37
(6*R*,7*R*)-8-Oxo-7-(2-(thiophen-2-yl)acetamido)-3-((*p*-tolylthio)methyl)-5-thia-1-azabicyclo[4.2.0]oct-2-ene-2-carboxylic acid (**5**) 37
(6*R*,7*R*)-3-(((4-Fluorophenyl)thio)methyl)-8-oxo-7-(2-(thiophen-2-yl)acetamido)-5-thia-1-azabicyclo[4.2.0]oct-2-ene-2-carboxylic acid (**6**) 38
N-((6*R*,7*R*)-3-((Octylthio)methyl)-8-oxo-5-thia-1-azabicyclo[4.2.0]oct-2-en-7-yl)-2-(thiophen-2-yl)acetamide (**7**) 38

Mechanistic Experiments 40
Experiment A 40
Experiment B 41
Experiment C 41
Experiment D 42
Experiment E 43
Experiment F 43

References 44

NMR-Spectra 45

General Information

If reactions were performed under inert conditions, e.g. exclusion of water, oxygen or both, all experiments were carried out using established Schlenk techniques. Herein solvents were dried with common methods and afterwards stored under inert gas atmosphere (argon or nitrogen) over molecular sieves. In some cases, when explicitly mentioned, dry solvents were received from the mentioned suppliers. In general, when high vacuum (*in vacuo*) was stated in experimental procedures, typically a vacuum of 10^{-2}-10^{-3} mbar was applied. Degassing of solvents or reaction mixtures was performed by bubbling argon via cannula through the solvent or the reaction mixture during ultrasonication for about 20 min. All reagents were added in a counterstream of inert gas to keep the inert atmosphere. All reactions were stirred with Teflon-coated magnetic stirring bars.

Molecular sieves (Sigma-Aldrich, beads with 8-12 mesh) were activated in a round-bottom flask with a gas inlet adapter by heating them carefully in a heating mantle at level 1 at least for 24 h under high vacuum until complete dryness was obtained. These activated molecular sieves were stored at rt under argon atmosphere.

Temperatures were measured externally if not otherwise stated. When working at a temperature of 0 °C, an ice-water bath served as the cooling medium. Lower temperatures were achieved by using an acetone/dry ice cooling bath. Reactions, which were carried out at higher temperatures than rt, were heated in a silicon oil bath on a heating plate (RCT basic IKAMAG® safety control, 0-1500 rpm) equipped with an external temperature controller.

Chemicals

All commercially available chemicals and solvents were purchased from Acros Organics, Alfa Aesar, Fisher, Fluka, Honeywell, Merck, Roth, Sigma-Aldrich, TCI, VWR and used without further purification, unless otherwise stated.

<u>Acetonitrile:</u> Anhydrous acetonitrile was purchased from Alfa Aesar. It was transferred into an amber 1 L Schlenk bottle and stored over activated 3 Å MS under argon atmosphere.

<u>Dichloromethane:</u> Anhydrous dichloromethane was produced by pre-drying EtOH stabilized dichloromethane over P_4O_{10}, distilling it, and afterwards heating it under reflux over CaH_2 for 24 h under argon atmosphere. It was distilled into an amber 1 L Schlenk bottle over activated 4 Å MS and under argon atmosphere.

<u>*N*,*N*-Dimethylformamide:</u> *N*,*N*-Dimethylformamide was purchased in extra dry quality from Alfa Aesar. It was transferred into an amber 1 L Schlenk bottle and stored over activated 4 Å MS under argon atmosphere.

<u>Methanol:</u> Methanol was purchased from Fisher and heated under reflux over Mg and I_2 for 2 h. It was distilled into an amber 1 L Schlenk bottle and stored over activated 3 Å MS under argon atmosphere.

<u>THF:</u> THF was purchased from Fisher and heated under reflux over CaH_2 for 24 h under argon atmosphere. It was distilled into an amber 1 L Schlenk bottle and stored over activated 4 Å MS under argon atmosphere.

Thin Layer Chromatography

Analytical thin layer chromatography (TLC) was carried out on Merck TLC silica gel aluminum sheets (silica gel 60, F254, 20 x 20 cm). All separated compounds were visualized by UV light (λ = 254 nm and/or λ = 366 nm) and by the listed staining reagents followed by the development in the heat.

$KMnO_4$: 3.0 g $KMnO_4$ as well as 20 g K_2CO_3 were dissolved in 300 mL H_2O and afterwards 5.0 mL 5 % aq. NaOH were added.

CAM: 50 g $(NH_4)_6Mo_7O_{24}$, 2.0 g $Ce(SO_4)_2$ and 50 mL conc. H_2SO_4 were dissolved in 400 mL water.

Flash Column Chromatography

Flash column chromatography was performed on silica gel 60 from Acros Organics with particle sizes between 35 µm and 70 µm. Depending on the problem of separation, a 30 to 100 fold excess of silica gel was used with respect to the dry amount of crude material. The dimension of the column was adjusted to the required amount of silica gel and formed a pad between 10 cm and 30 cm. In general, the silica gel was mixed with the eluent and the column was equilibrated. Subsequently, the crude material was dissolved in the eluent and loaded onto the top of the silica gel and the mobile phase was forced through the column using a rubber bulb pump. The volume of each collected fraction was adjusted between 20 % and 40 % of the silica gel volume.

Gas Chromatography

GC-MS analyses were performed on an Agilent Technologies 7890A GC system equipped with a 5975C mass selective detector (inert MSD with Triple Axis Detector system) by electron-impact ionization (EI) with a potential of E = 70 eV. Herein, the samples were separated depending on their boiling point and polarity. The desired crude materials or pure compounds were dissolved and the solutions were injected by employing the autosampler 7683B in a split mode 1/20 (inlet temperature: 280 °C; injection volume: 0.2 µL). Separations were carried out on an Agilent Technologies J&W GC HP-5MS capillary column ((5 %-phenyl)methylpolysiloxane, 30 m x 0.2 mm x 0.25 µm) with a constant helium flow rate (He 5.0 (Air Liquide), 1.085 $mL \cdot min^{-1}$, average velocity: 41.6 $cm \cdot s^{-1}$). A general gradient temperature method was used:

50S: initial temperature: 50 °C for 1 min; linear increase to 300 °C (40 $°C \cdot min^{-1}$); hold for 5 min; 1 min post-run at 300 °C; detecting range: 50.0-550.0 amu; solvent delay: 2.60 min.

High Performance Liquid Chromatography

Analytical HPLC-MS measurements were performed on an Agilent Technologies 1200 Series system (G1379 Degasser, G1312 Binary Pump, G1367C HiP ALS SL Autosampler, G1330B FC/ALS Thermostat, G1316B TCC SL column compartment, G1365C MWD SL multiple wavelength detector (deuterium lamp, 190-400 nm)) equipped with a single quadrupole LCMS detector “6120 LC/MS” using electrospray ionization source (ESI in positive and negative mode). All separations were carried out on a reversed phase Agilent Poroshell 120 EC-C18 (100 x 3.0 mm, 2.7 µm) column equipped with a Merck LiChroCART® 4-4 pre-column. The following method was used:

2-100MeCN: 0.0 min: 98 % H_2O + 0.05 % TFA and 2 % CH_3CN; 0.0-6.0 min: linear gradient to 100 % CH_3CN; 6.0-8.0 min: 100 % CH_3CN; 8.0-8.5 min: linear gradient to 98 % H_2O + 0.05 % TFA and 2 % CH_3CN; 8.5-9.5 min: 98 % H_2O + 0.05 % TFA and 2 % CH_3CN; 0.700 $mL \cdot min^{-1}$; 35 °C.

Semi-Preparative High Performance Liquid Chromatography

Semi-preparative HPLC purifications were performed on a "Thermo Scientific Dionex UltiMate 3000" system with UltiMate 3000 pump, UltiMate 3000 autosampler, UltiMate 3000 column compartment, UltiMate 3000 diode array detector (deuterium lamp, λ = 190–380 nm) and an UltiMate 3000 automatic fraction collector. The components were separated on a RP Macherey-Nagel 125/21 Nucleodur® 100-5 C18ec column (21 × 125 mm, 5.0 µm). Signals were detected at 210 nm and 254 nm. As mobile phase acetonitrile (VWR HiPerSolv, HPLC grade) and water (Barnstead NANOpure®, ultrapure water system) with 0.05 % trifluoroacetic acid were used. The following method was used:

method: 0.0-5.0 min: 98 % H_2O + 0.05 % TFA and 2 % CH_3CN; 5.0-45.0 min: linear gradient to 100 % CH_3CN; 45.0-60.0 min: 100 % CH_3CN; 15.00 $mL \cdot min^{-1}$; 30 °C.

Nuclear Magnetic Resonance Spectroscopy

NMR spectra were recorded on a Bruker AVANCE III 300 spectrometer (^{1}H: 300.36 MHz; ^{13}C: 75.53 MHz) with autosampler. Chemical shifts δ are referenced to the residual proton and carbon signal of the deuterated solvent ($CDCl_3$: δ = 7.26 ppm (^{1}H), 77.16 ppm (^{13}C); DMSO-d_6: δ = 2.50 ppm (^{1}H), 39.52 ppm (^{13}C); D_2O: δ = 4.79 ppm (^{1}H)). Chemical shifts δ are given in ppm (parts per million) and coupling constants *J* in Hz (Hertz). If necessary, 1D spectra (APT and NOESY) as well as 2D spectra (H,H-COSY, HSQC, HMBC) were recorded for the identification and confirmation of the structure. Signal multiplicities are abbreviated as s (singlet), br s (broad singlet), d (doublet), dd (doublet of doublet), td (triplet of doublet), t (triplet), dt (doublet of triplet), q (quadruplet), p (pentet) and m (multiplet). Deuterated solvents for nuclear resonance spectroscopy were purchased from euriso-top®.

High Resolution Mass Spectrometry

High-resolution mass spectra were recorded on a Waters Micromass GCT Premier system. Ionization was realized by an electron impact source (EI ionization) at a constant potential of 70 eV. Herein, individual samples were either inserted directly (direct inlet electron impact ionization; DI-EI) or prior to this gas chromatographically separated on an Agilent 7890A system equipped with an Agilent Technologies J&W GC-column DB-5MS (length: 30 m; inner-diameter: 0.250 mm; film: 0.25 µm) at a constant helium flow. Molecule ions were analyzed by a time-of-flight (TOF) mass analyzer in the positive mode (TOF MS EI+).

Further high-resolution mass spectra were recorded using MALDI TOF on a Waters Micromass® MALDI micro MX Mass spectrometer. Dithranol (1,8-dihydroxy-9,10-dihydroanthracen-9-one) served as matrix and PEG as internal standard. Besides molecular formulas, calculated as well as determined *m/z* ratios of each molecule peak are denoted.

Determination of Melting Points

Melting points were determined on a Mel-Temp® melting point apparatus from Electrothermal with an integrated microscopical support. They were measured in open capillary tubes with a mercury-in-glass thermometer and were not corrected.

Determination of Optical Rotation

The specific optical rotation was determined on a Perkin Elmer Polarimeter 341 with an integrated sodium vapor lamp. All samples were measured at the D-line of the sodium light (λ = 589 nm) in a 10 cm cell. Concentrations are given in g/100 mL. Each optical rotation measurement was performed five times and the mean value is reported.

Experimental Procedures

Reaction Optimization

Ligand Screening

Table S1. Ligand screening for the optimization of the Pd-catalyzed allylation of 1-octanethiol.

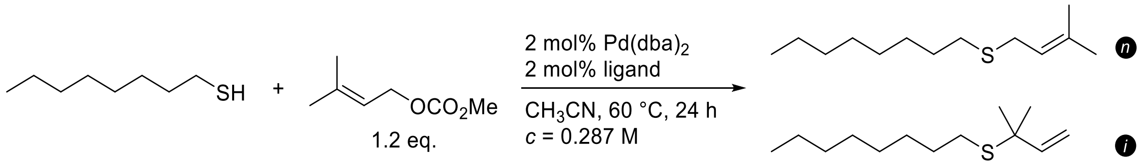

Ligand	conv. [%][a]	*n*/*i*[a]	Ligand	conv. [%][a]	*n*/*i*[a]
**L1**	0	n.d.	**L26**	0	n.d.
**L2**	0	n.d.	**L27**	13	24/76
**L3**	5	>99/1	**L28**	0	n.d.
**L4**	7	92/8	**L29**	42[b]	n.d.
**L5**	0	n.d.	**L30**	53[b]	n.d.
**L6**	2	n.d.	**L31**	0	n.d.
**L7**	6	89/11	**L32**	4	89/11
**L8**	42	62/38	**L33**	67[b]	n.d.
**L9**	99	92/8	**L34**	61[b]	n.d.
**L10**	89	77/23	**L35**	0	n.d.
**L11**	96	41/59	**L36**	5	80/20
**L12**	0	n.d.	**L37**	0	n.d.
**L13**	0	n.d.	**L38**	3	79/21
**L14**	0	n.d.	**L39**	0	n.d.
**L15**	45	51/49	**L40**	4	82/18
**L16**	0	n.d.	**L41**	64	67/33
**L17**	2	n.d.	**L42**	74	71/29
**L18**	0	n.d.	**L43**	81	70/30
**L19**	0	n.d.	**L44**	93	32/68
**L20**	13	83/17	**L45**	99	69/31
**L21**	0	n.d.	**L46**	99	58/42
**L22**	33	88/12	**L47**	98	57/43
**L23**	92	92/8	**L48**	97	35/65
**L24**	72	63/37	**L49**	0	n.d.
**L25**	80	18/82	**L50**	>99	>99/1

[a] Conversions as well as *n*/*i* ratios were determined by GC-MS with diglyme as internal standard. n.d. = not determined. [b] Only disulfide formed.

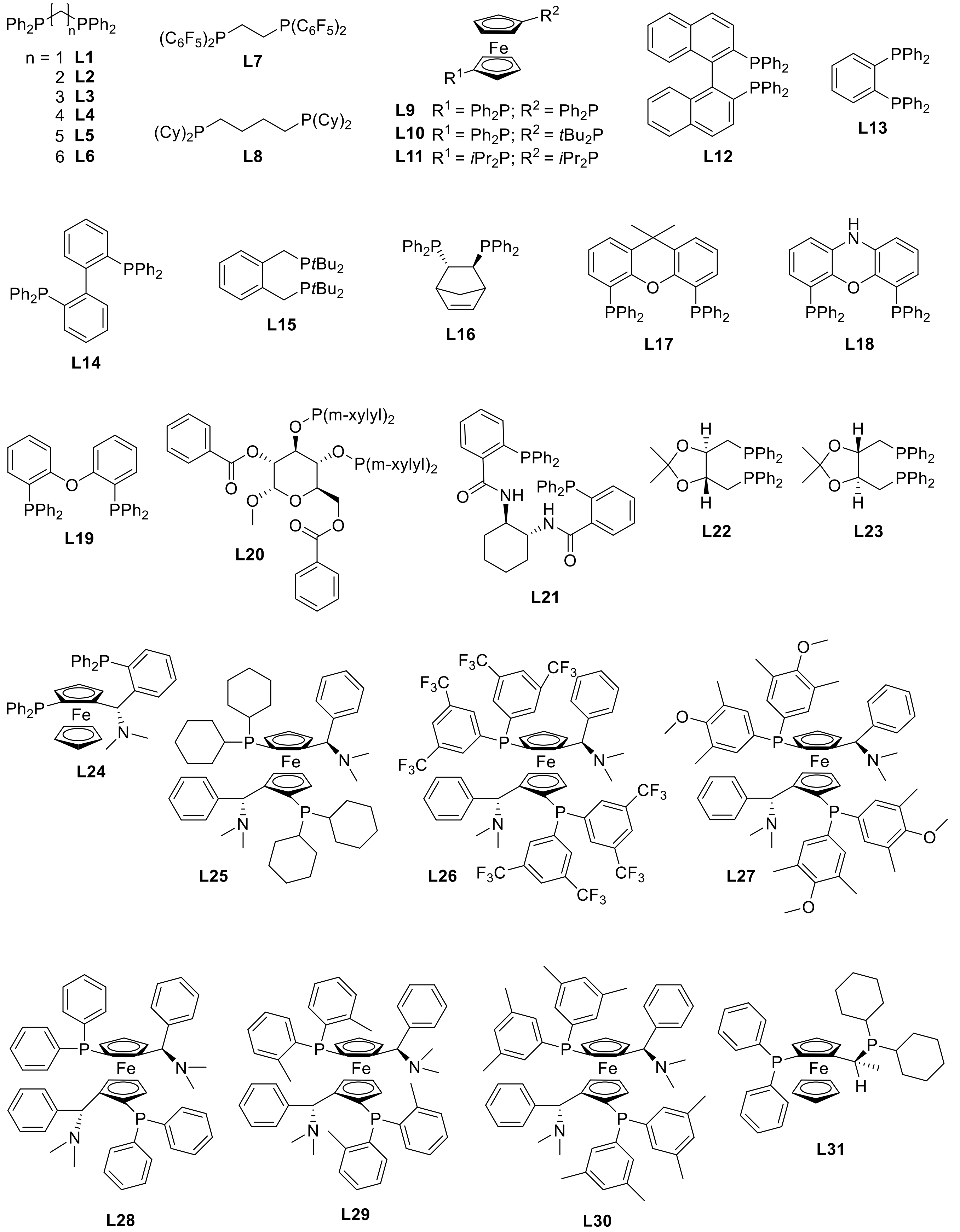

Figure S1. Structures of ligands **L1-L31**.

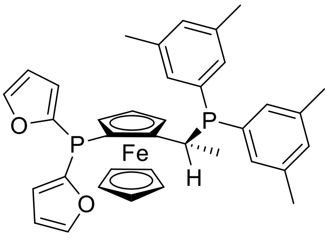

L32

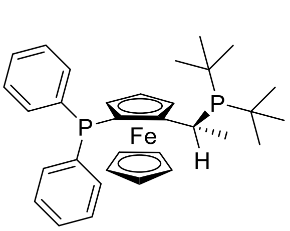

L33

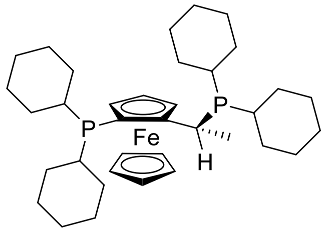

L34

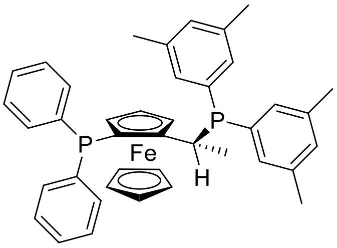

L35

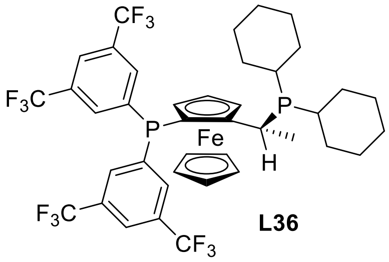

L36

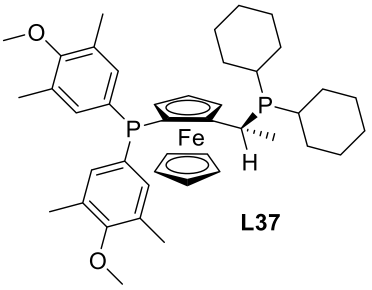

L37

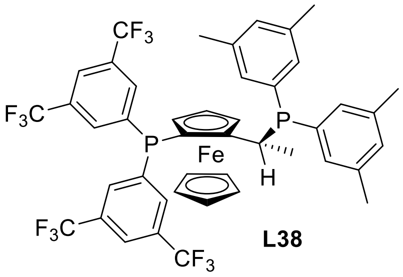

L38

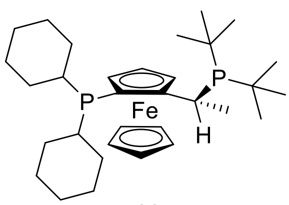

L39

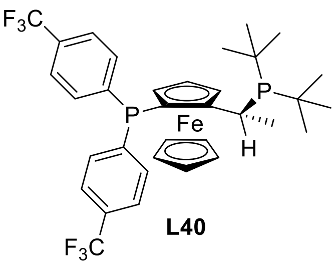

L40

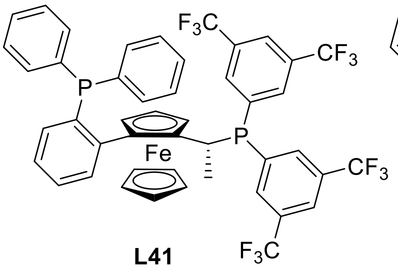

L41

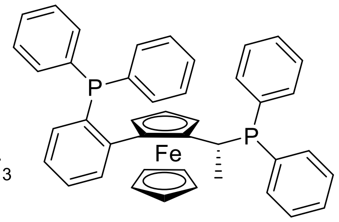

L42

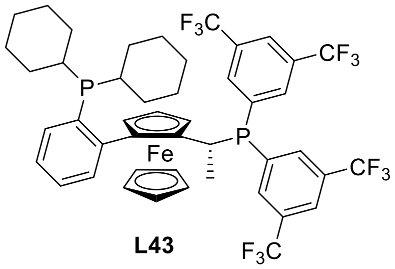

L43

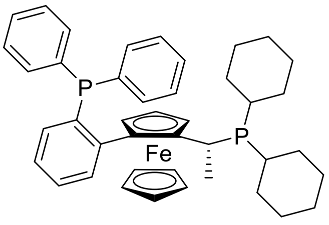

L44

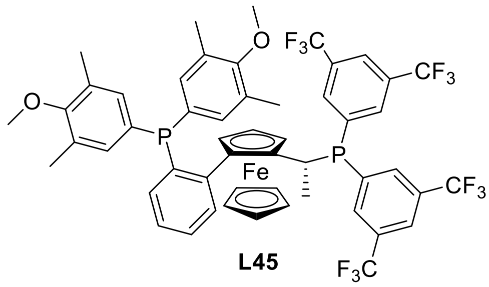

L45

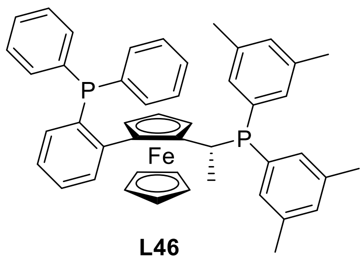

L46

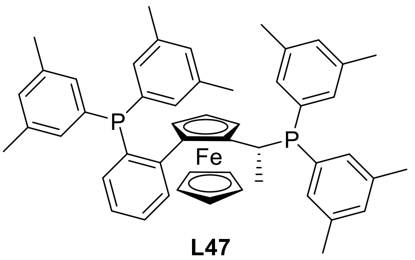

L47

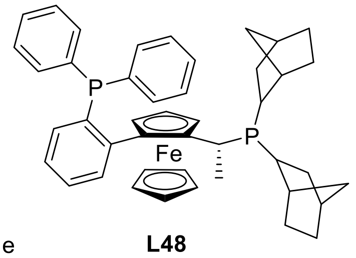

L48

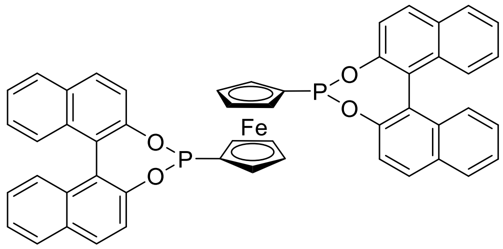

L49

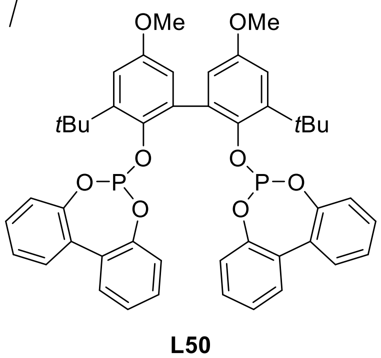

L50

Figure S2. Structures of ligands **L32**-**L50**.

Temperature Screening

Table S2. Temperature screening for the optimization of the Pd-catalyzed allylation of 1-octanethiol.

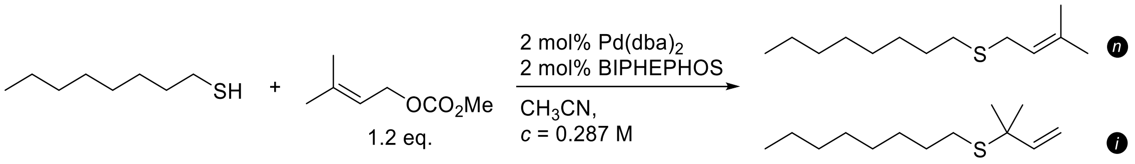

Entry	*T* [°C]	conv. after 30 min [%] (*n*/*i*-ratio)[a]	conv. after 2 h [%] (*n*/*i*-ratio)[a]	conv. after 24 h [%] (*n*/*i*-ratio)[a]
1	rt	>99 (87/13)	>99 (86/14)	>99 (90/10)
2	37	>99 (86/14)	>99 (87/13)	>99 (91/9)
3	60	>99 (89/11)	>99 (94/6)	>99 (>99/1)
4	reflux	>99 (90/10)	>99 (91/9)	>99 (98/2)

[a] Conversions as well as *n*/*i* ratios were determined by GC-MS without internal standard.

Catalyst Loading Screening

Table S3. Catalyst loading screening for the optimization of the Pd-catalyzed allylation of 1-octanethiol.

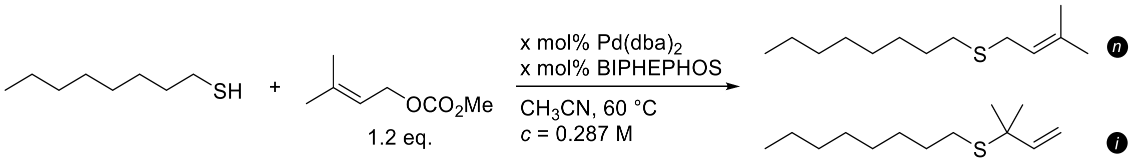

Entry	*x* [mol%]	conv. after 30 min [%] (*n*/*i*-ratio)[a]	conv. after 2 h [%] (*n*/*i*-ratio)[a]	conv. after 24 h [%] (*n*/*i*-ratio)[a]
1	2.0	>99 (90/10)	>99 (94/6)	>99 (>99/<1)
2	1.0	>99 (82/18)	>99 (85/15)	>99 (93/7)
3	0.5	>99 (87/13)	>99 (88/12)	>99 (92/8)
4	0.1	38 (85/15)	37 (87/13)	45 (89/11)
5	0.05	3 (83/17)	5 (83/17)	6 (n.d.)

[a] Conversions as well as *n*/*i* ratios were determined by GC-MS without internal standard. n.d. = not determined

Solvent Screening

Table S4. Solvent screening for the optimization of the Pd-catalyzed allylation of 1-octanethiol.

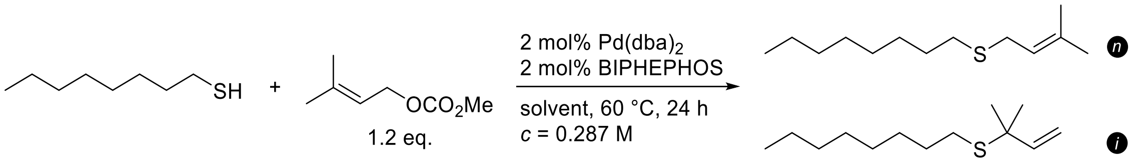

Entry	Solvent	30 min		24 h	
		conv. [%][a]	*n*/*i*[a]	conv. [%][a]	*n*/*i*[a]
1	acetonitrile	>99	90/10	>99	>99/1
2	propionitrile	>99	98/2	>99	>99/1
3	methanol	>99	95/5	>99	>99/1
4	ethanol	>99	95/5	>99	>99/1
5	ethanol/H_2O (5/1)	>99	93/7	>99	97/3
6	1-propanol	>99	98/2	>99	99/1
7	2-propanol	>99	91/9	>99	94/6
8	1-butanol	>99	97/3	>99	99/1
9	*tert*-butanol	99[d]	97/3	99[d]	96/4
10	*tert*-butanol/H_2O (3/1)	>99	89/11	>99	97/3
11	benzyl alcohol	>99[b]	87/13	>99[b]	98/2
12	2,2,2-trifluorethanol	>99[b]	93/7	>99[b]	>99/1
13	DCM[c]	89	86/14	92	96/4
14	chloroform	99[b]	94/6	>99[b]	>99/1
15	1,2-dichloroethane (DCE)	7	>99/1	22	90/10
16	toluene	73[b]	94/6	97[b]	94/6
17	ethyl acetate	98	94/6	>99	97/3
18	acetone	>99	91/9	>99	96/4
19	1,4-dioxane	75[d]	90/10	93[d]	94/6
20	THF	82	93/7	86	93/7
21	2-methyl-THF	82	92/8	92	94/6
22	MTBE[c]	89	95/5	90	91/9
23	NMP	>99	94/6	>99	>99/1
24	DMF	>99	81/19	>99	83/17
25	*N*,*N*-dimethylacetamide	>99	95/5	>99	99/1
26	DMSO	>99	87/13	>99	98/2
27	sulfolane	>99	93/7	>99	97/3

28	propylene carbonate	>99	94/6	>99	>99/1
29	diethylene glycol	99	92/8	99	92/8
30	DME	99	93/7	>99	98/2
31	acetic acid	87[b,d]	78/22	>99[b,d]	96/4

[a] Conversions as well as *n*/*i* ratios were determined by GC-MS without internal standard. [b] Unknown impurities relative to product and starting material monitored via GC-MS. [c] Reflux conditions due to a lower boiling point than 60 °C. [d] Disulfide formation detected.

Reversibility of the Pd-Catalyzed Allylation

Depending on the ligand used the Pd-catalyzed allylation can either be directed exclusively towards the *n*-thioether (**L50**) or with good selectivity towards the *i*-product (**L25**). It is noteworthy that the *n*/*i* selectivity increases even after full conversion is reached, indicating reversibility of the reaction.

Table S5. Time dependence of the *n*/*i* ratio during the Pd-catalyzed allylation of 1-octanethiol.

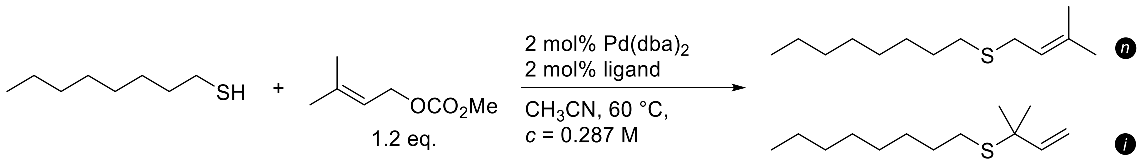

Entry	Time	BIPHEPHOS (**L50**)		**L25**	
		conv. [%][a]	*n*/*i* [a]	conv. [%][a]	*n*/*i* [a]
1	15 min	>99	87/13	n.d.	n.d.
2	30 min	>99	89/11	19	25/75
3	2 h	>99	94/6	79	20/80
4	5 h	>99	99/1	n.d.	n.d.
5	24 h	>99	>99/1	>99	18/82

[a] Conversions as well as *n*/*i* ratios were determined by GC-MS without internal standard. n.d. = not determined

Synthesis of Thiol Substrates

L-Cystine 1,1’-dimethyl ester hydrochloride (S1)

This compound was prepared according to a procedure described by Busnel et al.[1]

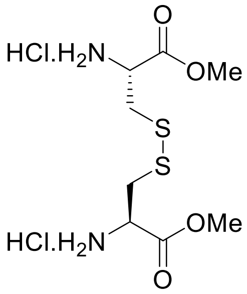

A 500 mL three-necked round-bottom flask, equipped with a Teflon-coated magnetic stirring bar, a dropping funnel, a reflux condenser and a bubbler, was charged with methanol (240 mL) and cooled to 0 °C in an ice-bath. Thionyl chloride (15 mL, 206 mmol) was added dropwise under vigorous stirring. Afterwards L-cystine (15.0 g, 62.4 mmol) was added in one portion and the resulting mixture was heated under reflux for 20 h. Removal of the volatiles under reduced pressure gave a crude product, which was recrystallized from MeOH/Et_2O (according to Zervas et al.[2]) to afford the desired compound as colorless solid (21.0 g, 98 %).

$C_8H_{18}Cl_2N_2O_4S_2$ [341.28 g·mol^{-1}]
m.p. = 166-170 °C
^{1}H NMR (300 MHz, D_2O): δ = 4.62-4.58 (m, 2H), 3.88 (s, 6H), 3.47-3.33 (m, 4H).
^{13}C NMR (76 MHz, D_2O): δ = 169.1, 54.0, 51.6, 35.7.
Analytical data are in accordance with the literature.[3]

L-Cystine *N*,*N’*-bis[(phenylmethoxy)carbonyl] 1,1’-dimethyl ester (S2)

This compound was prepared according to a procedure described by Carrasco et al.[4]

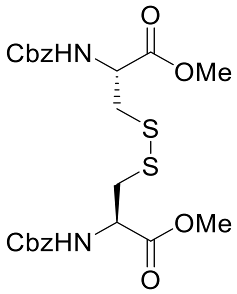

A 100 mL three-necked round-bottom flask, equipped with a Teflon-coated magnetic stirring bar, dropping funnel, was charged with L-cystine 1,1’-dimethyl ester hydrochloride (**S1**) (7.00 g, 20.5 mol), potassium bicarbonate (17.0 g, 123 mmol), H_2O (40 mL), Et_2O (35 mL) and the resulting solution was cooled to 0 °C (ice-bath). Benzyl chloroformate (6.41 mL, 45.1 mmol) was added dropwise within 30 min, the cooling bath was removed and the solution was stirred at rt for 5 h. Glycine (0.46 g, 6.2 mmol) was added (to scavenge excessive chloroformate) and the solution was stirred for an additional 18 h. The organic layer was separated and the aqueous layer was extracted with Et_2O (2 x 15 mL). The combined organic phases were washed with 0.01 M HCl (2 x 25 mL), H_2O

(2 x 25 mL), brine (25 mL), dried over Na_2SO_4, filtered and concentrated under reduced pressure. The resulting oil was further dried in a Kugelrohr oven (50 °C, 0.05 mbar, 5 h) to afford the desired compound as a clear oil that solidified upon cooling (7.90 g, 72 %).

$C_{24}H_{28}N_2O_8S_2$ [536.62 g·mol^{-1}]
R_f = 0.24 (cyclohexane:EtOAc = 2:1 (v/v), CAM)
^{1}H NMR (300 MHz, $CDCl_3$): δ = 7.35-7.30 (m, 10H), 5.72 (d, *J* = 7.7 Hz, 2H), 5.11 (s, 4H), 4.66 (q, *J* = 7.8 Hz, 5.1 Hz, 2H), 3.74 (s, 6H), 3.16 (d, *J* = 5.0 Hz, 4H).
^{13}C NMR (76 MHz, $CDCl_3$): δ = 170.9, 155.8, 136.2, 128.6, 128.4, 128.3, 67.3, 53.4, 52.9, 41.2.
Analytical data are in accordance with the literature.[5]

Cbz-Cys-OMe (1p)

This compound was prepared according to a procedure described by Busnel et al.[1]

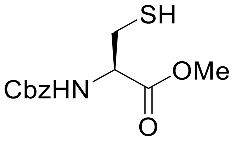

In a 100 mL round-bottom flask, equipped with a Teflon-coated magnetic stirring bar, L-Cystine *N*,*N*'-bis[(phenylmethoxy)carbonyl] 1,1'-dimethyl ester (**S2**) (4.65 g, 8.67 mmol), triphenylphosphine (2.39 g, 8.76 mmol) and sodium acetate (0.28 g, 3.46 mmol) were suspended in a mixture of methanol (32.5 mL) and water (16.25 mL). A colorless precipitate formed, which dissolved upon addition of glacial acetic acid (0.28 mL). Afterwards the solution was heated under reflux for 24 h. The mixture was diluted with CH_2Cl_2 (475 mL), washed with H_2O (2 x 200 mL), brine (2 × 200 mL), dried over $MgSO_4$, filtered and concentrated under reduced pressure. The residue was purified via flash column chromatography (625 g SiO_2, 25.0 x 8.0 cm, cyclohexane:EtOAc = 4:1 (v/v)) to give the desired compound as a colorless solid (3.88 g, 83 %).

$C_{12}H_{15}NO_4S$ [269.32 g·mol^{-1}]
R_f = 0.21 (cyclohexane:EtOAc = 4:1 (v/v), CAM)
m.p. = 20-25 °C
$[\alpha]_D^{20}$ = - 26.7 (0.5, DMSO)
^{1}H NMR (300 MHz, $CDCl_3$): δ = 7.36-7.32 (m, 5H), 5.71 (d, *J* = 6.9 Hz, 1H), 5.13 (s, 2H), 4.68 (dt, *J* = 7.8 Hz, 4.0 Hz, 1H), 3.78 (s, 3H), 3.02-2.98 (m, 2H), 1.39 (t, *J* = 9.0 Hz, 1H).
^{13}C NMR (76 MHz, $CDCl_3$): δ = 170.6, 155.8, 136.1, 128.7, 128.4, 128.3, 67.3, 55.4, 52.9, 27.3.
Analytical data are in accordance with the literature.[6]

Dimethyl 10,10'-(disulfanediylbis(methylene))(5*S*,5'*S*,10*R*,10'*R*)-bis(5-(methoxycarbonyl)-3,8,11-trioxo-1-phenyl-2-oxa-4,9,12-triazatetradecan-14-oate) (S3)

This compound was prepared according to a procedure described by Chalker et al.[7]

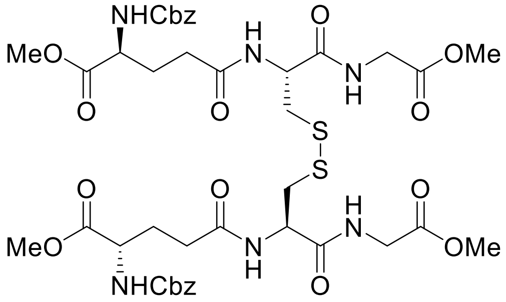

A flame-dried and argon-flushed 250 mL two-necked round-bottom flask, equipped with a Teflon-coated magnetic stirring bar, a dropping funnel and a bubbler, was charged with anhydrous methanol (40 mL) and cooled to 0 °C in an ice-bath. Acetyl chloride (9.29 mL, 130 mmol) was slowly added and the resulting solution was stirred for 15 min at 0 °C. Afterwards L-glutathione (4.0 g, 13.0 mmol) was added in one portion and the ice-bath was removed. The reaction was stirred for 6 h at rt. Then all volatiles were removed under reduced pressure.

The crude residue was dissolved in DMF (100 mL) and the solution was cooled to 0 °C before triethylamine (9.1 mL, 65.1 mmol) and iodine (1.65 g, 6.51 mmol) were successively added under vigorous stirring. After 15 min benzyl chloroformate (1.85 mL, 13.0 mmol) was added and the reaction mixture was stirred for an additional 2 h. Then the solution was diluted with EtOAc (320 mL) and washed with 1 M HCl (320 mL), H_2O (130 mL) and brine (130 mL). The organic layer was separated, dried over $MgSO_4$, filtered, and concentrated under reduced pressure. The crude product was purified via flash column chromatography (650 g SiO_2, 27. x 8.0 cm, CH_2Cl_2:CH_3OH = 19:1 (v/v)). Afterwards the isolated product was recrystallized from hot CH_2Cl_2/petroleum ether (100 mL/40 mL) and cooling to -18 °C overnight to give a pale yellow powder (567.7 mg, 9 %). The isolated product still was contaminated with Cbz-γ-Glu(OMe)-Cys(Cbz)-Gly-OMe and was used for the next step without further purification.

$C_{40}H_{52}N_6O_{16}S_2$ [937.00 g·mol^{-1}]
R_f = 0.20 (CH_2Cl_2:CH_3OH = 20:1 (v/v), CAM)
HRMS (MALDI-TOF): calcd. for $C_{40}H_{52}N_6O_{16}S_2Na^+$ $[M+Na]^+$: 959.2779; found: 959.2823.

Cbz-γ-Glu(OMe)-Cys-Gly-OMe (1q)

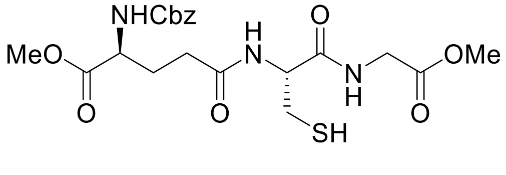

A flame-dried and argon-flushed 25 mL two-necked round-bottom flask, equipped with a Teflon-coated magnetic stirring bar and a bubbler, was charged with crude **S3** (542.4 mg, 0.58 mmol), degassed methanol (6.76 mL) and degassed H_2O (1.35 mL). Afterwards, PBu_3 (281 µL, 0.75 mmol), was added and the mixture was stirred vigorously for 90 min. The solution was concentrated under reduced pressure and the residue was purified via flash column chromatography (85 g SiO_2, 23.0 x 3.5 cm, CH_2Cl_2:CH_3OH = 19:1 (v/v)). Afterwards the isolated product was recrystallized twice from hot CH_2Cl_2/petroleum ether (68 mL/45 mL) and cooling to -18 °C overnight to give the desired compound as colorless powder (335.7 mg, 62 %).

$C_{20}H_{27}N_3O_8S$ [469.51 g·mol^{-1}]

R_f = 0.23 (CH_2Cl_2:CH_3OH = 19:1, CAM)

m.p. = 176 °C

$[\alpha]_D^{20}$ = - 35.0 (0.25, CH_3OH)

^{1}H NMR (300 MHz, $CDCl_3$): δ = 7.33 (m, 5H), 7.05 (m, 1H), 6.79 (d, *J* = 7.7 Hz, 1H), 5.71 (d, *J* = 7.8 Hz, 1H), 5.07 (s, 2H), 4.66 (m, 1H), 4.39 (m, 1H), 3.99 (m, 2H), 3.72 (s, 3H), 3.70 (s, 3H), 3.12-2.96 (m, 1H), 2.86-2.66 (m, 1H), 2.35 (t, *J* = 6.6 Hz, 2H), 2.29-2.11 (m, 1H), 2.09-1.85 (m, 1H), 1.77 (t, *J* = 8.5 Hz, 1H).

^{13}C NMR (76 MHz, $CDCl_3$): δ = 172.7, 172.4, 170.3, 170.3, 156.4, 136.3, 128.8, 128.5, 128.4, 67.4, 54.4, 53.4, 52.9, 52.6, 41.4, 32.2, 28.5, 26.5.

HRMS (MALDI-TOF): No HRMS data could be obtained due to disulfide formation during the measurement.

Synthesis of Allylic Alcohols

Cyclohex-1-en-1-ylmethanol (S4)

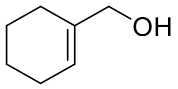

A flame-dried and argon-flushed 250 mL two-necked flask, equipped with a Teflon-coated magnetic stirring bar and a pressure compensator, was charged with DIBAL (1 M soln. in pentane, 33.0 mL, 32.8 mmol) and anhydrous CH_2Cl_2 (40 mL). This solution was cooled to -78 °C (dry ice / acetone) before methyl cyclohex-1-encarboxylate (1.95 mL, 14.3 mmol) was added dropwise. Afterwards this solution was stirred at -78 °C for additional 25 min. The excess of DIBAL was destroyed by carefully adding MeOH (20 mL) and 15 % NaOH (40 mL) at -78 °C. The two layers were separated and the aq. phase was extracted with CH_2Cl_2 (10 mL). The combined organic phases were dried over $MgSO_4$, filtered and concentrated under reduced pressure. The greenish crude product was purified via flash column chromatography (180 g SiO_2, 15.0 x 7.0 cm, cyclohexane:EtOAc = 5:1 (v/v)) affording the desired product as a pale yellow oil (1.40 g, 87 %).

$C_7H_{12}O$ [112.17 g·mol^{-1}]
R_f = 0.32 (cyclohexane:EtOAc = 5:1 (v/v), $KMnO_4$)
GC-MS (method: 50S): t_R = 3.99 min; *m/z* = 112 (46), 81 (66), 79 (100), 77 (29).
^{1}H NMR (300 MHz, $CDCl_3$): δ = 5.67 (m, 1H), 3.96 (s, 2H), 2.02-2.00 (m, 4H), 1.64-1.56 (m, 5H).
^{13}C NMR (76 MHz, $CDCl_3$): δ = 137.1, 122.5, 67.2, 25.1, 24.5, 22.1, 22.0.
Analytical data are in accordance with the literature.[8]

1-Phenylprop-2-en-1-ol (S5)

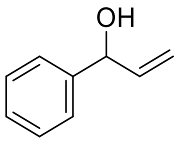

In a flame-dried and argon-flushed 250 mL Schlenk flask, equipped with a Teflon-coated magnetic stirring bar, benzaldehyde (2.0 mL, 19.7 mmol) was dissolved in anhydrous THF (20 mL) and cooled to -10 °C (ice/NaCl). Subsequently, a solution of vinylmagnesium bromide (1 M soln. in THF, 23.8 mL, 23.8 mmol) was added within 15 min. The resulting mixture was allowed to warm to rt and stirred for 2 h. After complete consumption of the starting material (according to TLC), the reaction was quenched by the addition of satd. NH_4Cl (30 mL) and diluted with H_2O (20 mL). The aqueous layer was back-extracted with EtOAc (3 x 50 mL) and the combined organic phases were dried over Na_2SO_4, filtered and concentrated under reduced pressure. The crude product was purified via Kugelrohr distillation (135 °C, 16 mbar) to give the desired compound as a colorless oil (2.02 g, 77 %).

$C_9H_{10}O$ [134.18 g·mol^{-1}]
R_f = 0.21 (cyclohexane:EtOAc = 10:1 (v/v), $KMnO_4$)
b.p. = approx. 135 °C (16 mbar)
^{1}H NMR (300 MHz, $CDCl_3$): δ = 7.48-7.16 (m, 5H), 6.11-5.87 (m, 1H), 5.28 (d, J = 17.1 Hz, 1H), 5.21-5.01 (m, 2H), 2.60 (br s, 1H).
^{13}C NMR (76 MHz, $CDCl_3$): δ = 142.7, 140.3, 128.6, 127.7, 126.4, 115.1, 75.3.
Analytical data are in accordance with the literature.[9]

Synthesis of Allylic Carbonates

General Procedure

In a round-bottom flask, equipped with a Teflon-coated magnetic stirring bar, methyl chloroformate was slowly added to an ice-cold solution of the corresponding allylic alcohol and pyridine in CH_2Cl_2. The resulting suspension was allowed to warm to rt. Upon complete consumption of the starting material (according to TLC), the reaction mixture was quenched by the addition of H_2O (1/3 of solvent volume) and stirred vigorously for at least 15 min. The organic layer was separated, washed twice with 1 M HCl (1/1 of solvent volume) and once with sat. $NaHCO_3$ (1/2 of the solvent volume), dried over Na_2SO_4, filtered, and concentrated under reduced pressure (in case of volatile products a minimal pressure of 40 mbar at 35 °C was applied).
Allylic carbonates **2a-2c**, **2e**, and **2g** were distilled to afford colorless products prior to use in allylation reactions, although crude products did not show any impurities according to ^{1}H-NMR-spectroscopy.

Methyl (3-methylbut-2-en-1-yl) carbonate (2a)

This compound was prepared according to a procedure described by Schlatzer et al.[10]

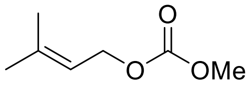

Following the general procedure, 3-methylbut-2-en-1-ol (6.94 g, 80.6 mmol) was reacted with pyridine (20.0 mL, 247 mmol) and methyl chloroformate (19.0 mL, 246 mmol) in CH_2Cl_2 (180 mL). General work up afforded the desired compound as yellowish oil (11.3 g, 97 %).

$C_7H_{12}O_3$ [144.17 g·mol^{-1}]
R_f = 0.51 (cyclohexane:EtOAc = 10:1 (v/v), $KMnO_4$)
GC-MS (method: 50S): t_R = 3.60 min; *m/z* (%) = 144 (1), 129 (1), 85 (42), 68 (100).
b.p. = 65-67 °C (19.5 mbar)
^{1}H NMR (300 MHz, $CDCl_3$): δ = 5.36 (t, *J* = 7.2 Hz, 1H), 4.62 (d, *J* = 7.2 Hz, 2H), 3.76 (s, 3H), 1.75 (s, 3H), 1.71 (s, 3H).
^{13}C NMR (76 MHz, $CDCl_3$): δ = 156.0, 140.1, 118.2, 64.8, 54.7, 25.9, 18.1.
Analytical data is in accordance with the literature.[11]

(*E*)-3,7-Dimethylocta-2,6-dien-1-yl methyl carbonate (2b)

This compound was prepared according to a procedure described by Schlatzer et al.[10]

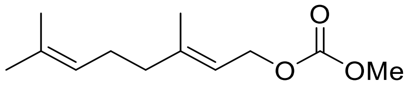

Following the general procedure, *trans*-geraniol (1.89 g, 12.2 mmol) was reacted with pyridine (3.2 mL, 40 mmol) and methyl chloroformate (3.0 mL, 39 mmol) in CH_2Cl_2 (30 mL). General work up afforded the desired compound as a brownish yellow oil (2.51 g, 96 %).

$C_{12}H_{20}O_3$ [212.29 g·mol^{-1}]

R_f = 0.46 (cyclohexane:EtOAc = 10:1 (v/v), $KMnO_4$)
b.p. = approx. 100 °C (0.12 mbar)
^{1}H NMR (300 MHz, $CDCl_3$): δ = 5.37 (t, *J* = 6.7 Hz, 1H), 5.12-5.03 (m, 1H), 4.65 (d, *J* = 7.1 Hz, 2H), 3.77 (s, 3H), 2.15-2.00 (m, 4H), 1.71 (s, 3H), 1.67 (s, 3H), 1.59 (s, 3H).
^{13}C NMR (76 MHz, $CDCl_3$): δ = 156.0, 143.3, 132.0, 123.8, 117.9, 64.9, 54.8, 39.6, 26.4, 25.8, 17.8, 16.6.
Analytical data are in accordance with the literature.[12]

((2*E*,6*E*)-3,7,11-Trimethyldodeca-2,6,10-trien-1-yl) methyl carbonate (2c)

This compound was prepared according to a procedure described by Schlatzer et al.[10]

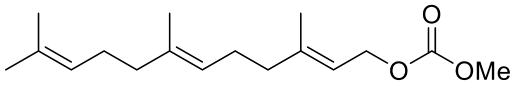

Following the general procedure, *trans,trans*-farnesol (0.968 g, 4.35 mmol) was reacted with pyridine (1.1 mL, 14 mmol) and methyl chloroformate (1.0 mL, 13 mmol) in CH_2Cl_2 (10 mL). General work up afforded the desired compound as yellow oil (1.11 g, 91 %).

$C_{17}H_{28}O_3$ [280.41 g·mol^{-1}]
R_f = 0.48 (cyclohexane:EtOAc = 10:1 (v/v), $KMnO_4$)
b.p. = approx. 145 °C (0.025 mbar)
^{1}H NMR (300 MHz, $CDCl_3$): δ = 5.37 (t, *J* = 6.8 Hz, 1H), 5.13-5.04 (m, 2H), 4.65 (d, *J* = 7.1 Hz, 2H), 3.77 (s, 3H), 2.20-1.87 (m, 8H), 1.72 (s, 3H), 1.67 (s, 3H), 1.59 (s, 6H).
^{13}C NMR (76 MHz, $CDCl_3$): δ = 156.0, 143.4, 135.7, 131.5, 124.5, 123.7, 117.9, 64.9, 54.8, 39.8, 39.7, 26.9, 26.3, 25.8, 17.8, 16.7, 16.1.
HRMS (EI-DI): calcd. for $C_{17}H_{28}O_3^+$ $[M]^+$: 280.2039; found: 280.2043.

((1*R*,5*S*)-6,6-Dimethylbicyclo[3.1.1]hept-2-en-2-yl)methyl methyl carbonate (2d)

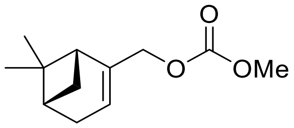

Following the general procedure, ((1*R*,5*S*)-6,6-dimethylbicyclo[3.1.1]hept-2-en-2-yl)methanol (5.10 mL, 32.9 mmol) was reacted with pyridine (8.00 mL, 98.7 mmol) and methyl chloroformate (7.60 mL, 98.7 mmol) in CH_2Cl_2 (70 mL). General work up afforded the desired compound as pale yellow oil (6.56 g, 96 %).

$C_{12}H_{18}O_3$ [210.27 g·mol^{-1}]
GC-MS (method: 50S): t_R = 5.53 min; *m/z* = 119 (44), 105 (14), 91 (100), 79 (28).
$[\alpha]_D^{20}$ = - 48.4 (1.0, CH_3CN)
^{1}H NMR (300 MHz, $CDCl_3$): δ = 5.60 (m, 1H), 4.49 (m, 2H), 3.76 (s, 3H), 2.43-2.09 (m, 5H), 1.28 (s, 3H), 1.18 (d, *J* = 8.7 Hz, 1H), 0.81 (s, 3H).
^{13}C NMR (76 MHz, $CDCl_3$): δ = 155.8, 142.4, 122.4, 70.5, 54.6, 43.4, 40.6, 38.0, 31.5, 31.3, 26.0, 20.9.
Analytical data are in accordance with the literature.[13]

Cyclohex-1-en-1-ylmethyl methyl carbonate (2e)

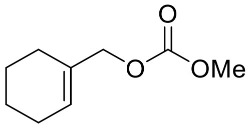

Following the general procedure, cyclohex-1-en-1-ylmethanol (1.43 mL, 12.3 mmol) was reacted with pyridine (2.98 mL, 37.0 mmol) and methyl chloroformate (2.86 mL, 37.0 mmol) in CH_2Cl_2 (25 mL). General work up and vacuum distillation afforded the desired compound as a colorless liquid (1.83 g, 97 %).

$C_9H_{14}O_3$ [170.21 g·mol^{-1}]
GC-MS (method: 50S): t_R = 4.98 min; m/z = 94 (38), 79 (100), 77 (20), 67 (15).
b.p. = 90 °C (15 mbar)
^{1}H NMR (300 MHz, $CDCl_3$): δ = 5.76 (s, 1H), 4.48 (s, 2H), 3.77 (s, 3H), 2.09-1.93 (m, 4H), 1.72-1.50 (m, 4H).
^{13}C NMR (76 MHz, $CDCl_3$): δ = 156.0, 132.5, 127.4, 72.6, 54.8, 25.9, 25.1, 22.4, 22.1.
HRMS (EI-DI): calcd. for $C_9H_{14}O_3^+$ [M]$^+$: 170.0943; found: 170.0938.

Methyl (*E*)-3-phenyl-2-propenyl carbonate (2f)

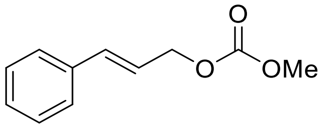

Following the general procedure, (*E*)-3-phenylprop-2-en-1-ol (5.00 g, 37.3 mmol) was reacted with pyridine (8.6 mL, 112 mmol) and methyl chloroformate (9.0 mL, 112 mmol) in CH_2Cl_2 (80 mL). General work up afforded the desired compound as a pale yellow oil (7.16 g, 99 %).

$C_{11}H_{12}O_3$ [192.21 g·mol^{-1}]
GC-MS (method: 50S): t_R = 5.13 min; *m/z* = 192 (16), 117 (76), 115 (100), 105 (35).
^{1}H NMR (300 MHz, $CDCl_3$): δ = 7.28-7.45 (m, 5H), 6.73 (d, *J* = 15.6 Hz, 1H), 6.34 (td, *J* = 15.9 Hz, 6.3 Hz, 1H), 4.83 (dd, *J* = 12.6 Hz, 1.2 Hz, 2H), 3.84 (s, 3H).
^{13}C NMR (76 MHz, $CDCl_3$): δ = 155.2, 135.6, 134.3, 128.1, 127.7, 126.2, 122.0, 67.9, 54.4.
Analytical data are in accordance with the literature.[14]

Methyl (1-phenylallyl) carbonate (2f*)

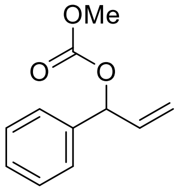

Following the general procedure, 1-phenylprop-2-en-1-ol (0.975 g, 7.27 mmol) was reacted with pyridine (1.8 mL, 22 mmol), DMAP (92.3 mg, 0.756 mmol) and methyl chloroformate (1.8 mL, 23 mmol) in CH_2Cl_2 (15 mL). General work up afforded the desired compound as a colorless oil (1.27 g, 91 %).

$C_{11}H_{12}O_3$ [192.21 g·mol^{-1}]

R_f = 0.51 (cyclohexane:EtOAc = 10:1 (v/v), $KMnO_4$)
^1H NMR (300 MHz, $CDCl_3$): δ = 7.37 (s, 5H), 6.20-5.92 (m, 2H), 5.47-5.09 (m, 2H), 3.78 (s, 3H).
^{13}C NMR (76 MHz, $CDCl_3$): δ = 155.2, 138.4, 135.9, 128.8, 128.6, 127.2, 117.6, 80.3, 54.9.
Analytical data are in accordance with the literature.[15]

(*E*)-Hex-2-en-1-yl methyl carbonate (2g)

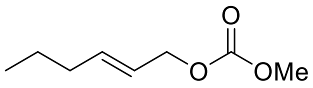

Following the general procedure, (*E*)-hex-2-en-1-ol (21.0 mL, 177 mmol) was reacted with pyridine (43 mL, 530 mmol) and methyl chloroformate (40 mL, 530 mmol) in CH_2Cl_2 (400 mL). General work up and vacuum distillation afforded the desired compound as a colorless oil (24.4 g, 87 %).

$C_8H_{14}O_3$ [158.20 g·mol^{-1}]
b.p. = 65 °C (15 mbar)
GC-MS (method: 50S): t_R = 4.26 min; *m/z* = 158 (2), 82 (50), 71 (62), 67 (100).
^1H NMR (300 MHz, $CDCl_3$): δ = 5.80 (dt, *J* = 14.4 Hz, 6.7 Hz, 1H), 5.57 (dtt, *J* = 15.4 Hz, 6.5 Hz, 1.3 Hz, 1H), 4.48 (dd, *J* = 6.4 Hz, 0.9 Hz, 2H), 3.75 (s, 3H), 2.02 (q, *J* = 7.1 Hz, 2H), 1.40 (sext, *J* = 7.5 Hz, 2H), 0.89 (t, *J* = 7.4 Hz, 3H).
^{13}C NMR (76 MHz, $CDCl_3$): δ = 155.8, 137.4, 123.5, 68.8, 54.8, 34.4, 22.1, 13.7.
Analytical data are in accordance with the literature.[16]

(*E*)-1,3-Diphenylallyl acetate (2h)

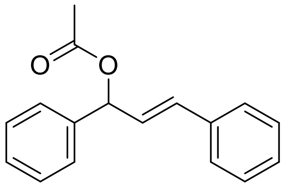

In a 25 mL round-bottom flask, equipped with a Teflon-coated magnetic stirring bar, acetyl chloride (0.32 mL, 4.5 mmol) was slowly added to a solution of (*E*)-1,3-diphenylprop-2-en-1-ol (0.630 g, 3.00 mmol), pyridine (0.49 mL, 6.1 mmol) and DMAP (37.8 mg, 0.31 mmol) in CH_2Cl_2 (5 mL). The resulting mixture was stirred at rt until complete consumption of the starting material (according to TLC). The reaction was quenched by the addition of H_2O (5 mL) and stirred vigorously for at least 15 min. The organic layer was separated, dried over Na_2SO_4, filtered, and concentrated under reduced pressure. Purification via flash column chromatography (125 g SiO_2, 13.5 x 5.0 cm, cyclohexane:EtOAc = 30:1 (v/v)) afforded the desired compound as a colorless oil (655.8 mg, 87 %).

$C_{17}H_{16}O_2$ [252.31 g·mol^{-1}]
R_f = 0.31 (cyclohexane:EtOAc = 30:1 (v/v), $KMnO_4$)
^1H NMR (300 MHz, $CDCl_3$): δ = 7.60-7.08 (m, 10H), 6.63 (d, J = 15.7 Hz, 1H), 6.53-6.22 (m, 2H), 2.13 (s, 3H).
^{13}C NMR (76 MHz, $CDCl_3$): δ = 170.2, 139.4, 136.3, 132.7, 128.8, 128.7, 128.3, 128.2, 127.7, 127.2, 126.8, 76.3, 21.5.
No HRMS data could be obtained due to intense fragmentation.

General Procedure for the Pd-Catalyzed *S*-Allylation

In a flame-dried and argon-flushed Schlenk flask, equipped with a Teflon-coated magnetic stirring bar, Pd(dba)$_2$ and BIPHEPHOS were suspended in anhydrous CH_3CN and stirred in a pre-heated oil bath at 60 °C for 30 min to obtain a bright yellow solution. Then allylic carbonate and thiol were added and the resulting mixture was stirred at 60 °C (except for **3la**-**3oa)** until complete consumption of the starting material (according to GC-MS or TLC). The reaction mixture was cooled to rt and concentrated under reduced pressure. The crude product was purified via flash column chromatography to afford the desired compound.

Allylic Carbonate Scope

(3-Methylbut-2-en-1-yl)(octyl)sulfane (3aa)

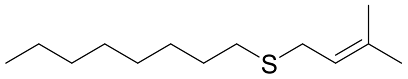

Following the general procedure, methyl prenyl carbonate (**2a**) (100 µL, 0.689 mmol) and 1-octanethiol (100 µL, 0.574 mmol) were reacted in the presence of Pd(dba)$_2$ (6.6 mg, 11.5 µmol) and BIPHEPHOS (9.0 mg, 11.5 µmol) in 2.0 mL anhydrous CH_3CN. Purification via flash column chromatography (18 g SiO_2, 20.0 x 1.5 cm, cyclohexane) afforded the desired compound as a yellow oil (112 mg, 91 %).

$C_{13}H_{26}S$ [214.41 g·mol^{-1}]
R_f = 0.32 (cyclohexane, CAM)
GC-MS (method: 50S): t_R = 6.05 min; *m/z* (%) = 214 (63), 144 (21), 101 (10), 68 (100).
^{1}H NMR (300 MHz, $CDCl_3$): δ = 5.23 (t, *J* = 7.5 Hz, 1H), 3.12 (d, *J* = 7.8 Hz, 2H), 2.45 (t, *J* = 7.5 Hz, 2H), 1.73 (s, 3H), 1.65 (s, 3H), 1.61-1.50 (m, 2H), 1.42-1.27 (m, 10H), 0.87 (t, *J* = 6.6 Hz, 3H).
^{13}C NMR (76 MHz, $CDCl_3$): δ = 134.8, 120.9, 31.9, 31.3, 29.9, 29.5, 29.2, 29.1, 27.0, 25.7, 22.7, 17.7, 14.1.
Analytical data are in accordance with the literature.[17]

(*E*)-(3,7-Dimethylocta-2,6-dien-1-yl)(octyl)sulfane (3ab)

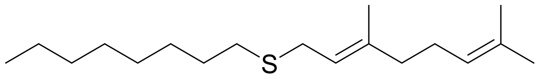

Following the general procedure, (*E*)-3,7-dimethylocta-2,6-dien-1-yl methyl carbonate (**2b**) (155 µL, 0.689 mmol) and 1-octanethiol (100 µL, 0.574 mmol) were reacted in the presence of Pd(dba)$_2$ (6.6 mg, 11.5 µmol) and BIPHEPHOS (9.0 mg, 11.5 µmol) in 2.0 mL anhydrous CH_3CN. Purification via flash column chromatography (20 g SiO_2, 14.0 x 2.0 cm, cyclohexane) afforded the desired compound as a yellow oil (154 mg, 95 %).

$C_{18}H_{34}S$ [284.52 g·mol^{-1}]
R_f = 0.17 (cyclohexane, CAM)
GC-MS (method: 50S): t_R = 7.33 min; *m/z* (%) = 282 (1), 121 (11), 93 (41), 81 (20), 69 (100).
^{1}H NMR (300 MHz, $CDCl_3$): δ = 5.23 (t, *J* = 7.8 Hz, 1H), 5.10-5.06 (m, 1H), 3.13 (d, *J* = 7.7 Hz, 2H), 2.44 (t, *J* = 7.2 Hz, 2H), 2.10-2.04 (m, 4H), 1.67-1.51 (m, 11H), 1.37-1.26 (m, 10H), 0.87 (t, *J* = 6.6 Hz, 3H).

^{13}C NMR (76 MHz, $CDCl_3$): δ = 138.4, 131.6, 124.0, 120.8, 39.8, 32.0, 31.2, 29.9, 29.4, 29.4, 29.3, 29.2, 26.6, 25.8, 22.8, 17.8, 16.1, 14.2.
HRMS (EI-DI): calcd. for $C_{18}H_{34}S^+$ $[M]^+$: 282.2381; found: 282.2390.

((2*E*,6*E*)-3,7,11-Trimethyldodeca-2,6,10-trien-1-yl)(octyl)sulfane (3ac)

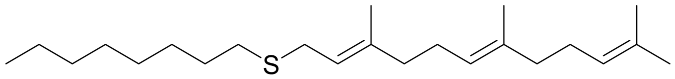

Following the general procedure, ((2*E*,6*E*)-3,7,11-trimethyldodeca-2,6,10-trien-1-yl) methyl carbonate (**2c**) (51 µL, 0.172 mmol) and 1-octanethiol (25 µL, 0.144 mmol) were reacted in the presence of $Pd(dba)_2$ (1.7 mg, 2.9 µmol) and BIPHEPHOS (2.3 mg, 2.9 µmol) in 0.5 mL anhydrous CH_3CN. Purification via flash column chromatography (6.5 g SiO_2, 35.0 x 0.5 cm, cyclohexane) afforded the desired compound as a yellow oil (49.9 mg, 99 %).

$C_{23}H_{42}S$ [350.64 g·mol^{-1}]
R_f = 0.20 (cyclohexane, CAM)
GC-MS (method: 50S): t_R = 8.44 min; *m/z* (%) = 350 (2), 119 (29), 93 (72), 69 (100).
^1H NMR (300 MHz, $CDCl_3$): δ = 5.24 (t, *J* = 7.7 Hz, 1 H), 5.11-5.07 (m, 2H), 3.14 (d, *J* = 7.7 Hz, 2H), 2.45 (t, *J* = 7.2 Hz, 2H), 2.11-1.95 (m, 8 H), 1.74-1.52 (m, 14 H), 1.38-1.27 (m, 10 H,), 0.88 (t, *J* = 6.7 Hz, 3H).
^{13}C NMR (76 MHz, $CDCl_3$): δ = 138.6, 135.4, 131.5, 124.5, 124.0, 120.9, 39.9, 39.8, 32.0, 31.3, 29.9, 29.5, 29.4, 29.4, 29.2, 26.9, 26.6, 25.8, 22.8, 17.8, 16.2, 16.2, 14.2.
HRMS (EI-DI): calcd. for $C_{23}H_{42}S^+$ $[M]^+$: 350.3007; found: 350.3057.

(((1*R*,5*S*)-6,6-Dimethylbicyclo[3.1.1]hept-2-en-2-yl)methyl)(octyl)sulfane (3ad)

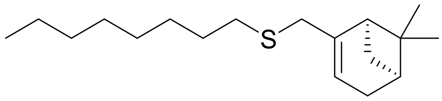

Following the general procedure, ((1*R*,5*S*)-6,6-dimethylbicyclo[3.1.1]hept-2-en-2-yl)methyl methyl carbonate (**2d**) (140 µL, 0.689 mmol) and 1-octanethiol (100 µL, 0.574 mmol) were reacted in the presence of $Pd(dba)_2$ (6.6 mg, 11.5 µmol) and BIPHEPHOS (9.0 mg, 11.5 µmol) in 2.0 mL anhydrous CH_3CN. Purification via flash column chromatography (20 g SiO_2, 14.0 x 2.0 cm, cyclohexane) afforded the desired compound as a yellow oil (158 mg, 98 %).

$C_{18}H_{32}S$ [280.51 g·mol^{-1}]
R_f = 0.50 (cyclohexane, CAM)
GC-MS (method: 50S): t_R = 7.32 min; *m/z* (%) = 280 (10), 134 (53), 119 (100), 91 (79).
$[\alpha]_D^{20}$ = - 6.3 (1.0, *n*-heptane)
^1H NMR (300 MHz, $CDCl_3$): δ = 5.34 (s, 1H), 3.05 (m, 2H), 2.44-2.37 (m, 3H), 2.27-2.19 (m, 3H), 2.11-2.09 (m, 1H), 1.55 (p, *J* = 7.5 Hz, 2H), 1.37-1.27 (m, 13H), 1.14 (d, *J* = 8.6 Hz, 1H), 0.90-0.83 (m, 6H).
^{13}C NMR (76 MHz, $CDCl_3$): δ = 143.8, 119.5, 45.1, 40.6, 38.2, 38.1, 31.8, 31.8, 31.3, 31.1, 29.3, 29.2, 29.2, 29.0, 26.2, 22.7, 21.1, 14.1.
HRMS (EI-DI): calcd. for $C_{18}H_{32}S^+$ $[M]^+$: 280.2225; found: 280.2222.

(Cyclohex-1-en-1-ylmethyl)(octyl)sulfane (3ae)

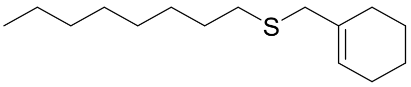

Following the general procedure, cyclohex-1-en-1-ylmethyl methyl carbonate (**2e**) (55 µL, 0.344 mmol) and 1-octanethiol (50 µL, 0.287 mmol) were reacted in the presence of Pd(dba)$_2$ (3.3 mg, 5.7 µmol) and BIPHEPHOS (4.5 mg, 5.7 µmol) in 1.0 mL anhydrous CH_3CN. Purification via flash column chromatography (6.5 g SiO_2, 15.5 x 1.0 cm, cyclohexane) afforded the desired compound as a yellow oil (59.2 mg, 86 %).

$C_{15}H_{28}S$ [240.45 g·mol^{-1}]
R_f = 0.43 (cyclohexane, CAM)
GC-MS (method: 50S): t_R = 6.86 min; *m/z* (%) = 240 (23), 145 (21), 95 (100), 79 (63).
^{1}H NMR (300 MHz, $CDCl_3$): δ = 5.54 (s, 1H), 3.05 (s, 2H), 2.39 (t, *J* = 7.4 Hz, 2H), 2.13-1.92 (m, 4H), 1.71-1.45 (m, 6H), 1.41-1.16 (m, 10H), 0.87 (t, *J* = 6.6 Hz, 3H).
^{13}C NMR (76 MHz, $CDCl_3$): δ = 134.1, 124.8, 39.9, 32.0, 31.2, 29.6, 29.4, 29.4, 29.1, 27.0, 25.5, 22.9, 22.8, 22.5, 14.2.
HRMS (EI-DI): calcd. for $C_{15}H_{28}S^+$ $[M]^+$: 240.1912; found: 240.1913.

Cinnamyl(octyl)sulfane (3af)

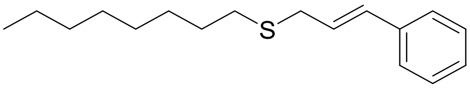

Following the general procedure, cinnamyl methyl carbonate (**2f**) (120 µL, 0.689 mmol) and 1-octanethiol (100 µL, 0.574 mmol) were reacted in the presence of Pd(dba)$_2$ (6.6 mg, 11.5 µmol) and BIPHEPHOS (9.0 mg, 11.5 µmol) in 2.0 mL anhydrous CH_3CN. Purification via flash column chromatography (20 g SiO_2, 14.0 x 2.0 cm, cyclohexane) afforded the desired compound as a yellow oil (141 mg, 94 %).

$C_{17}H_{28}S$ [178.29 g·mol^{-1}]
R_f = 0.13 (cyclohexane, CAM)
GC-MS (method: 50S): t_R = 7.61 min; *m/z* (%) = 262 (7), 117 (100), 105 (35), 91 (17).
^{1}H NMR (300 MHz, $CDCl_3$): δ = 7.38-7.19 (m, 5H), 6.42 (d, *J* = 15.9 Hz, 1H), 6.23-6.13 (m, 1H), 3.29 (d, *J* = 7.2 Hz, 2H), 2.48 (t, *J* = 7.2 Hz, 2H), 1.64-1.52 (m, 2H), 1.35-1.25 (m, 12H), 0.86 (t, *J* = 6.8 Hz, 3H).
^{13}C NMR (76 MHz, $CDCl_3$): δ = 136.8, 131.9, 128.5, 127.4, 126.3 (2x C), 34.3, 31.8, 30.8, 29.4, 29.2, 29.2, 28.9, 22.6, 14.1.
HRMS (EI-DI): calcd. for $C_{11}H_{14}S^+$ $[M]^+$: 262.1755; found: 262.1755.

(*E*)-Hex-2-en-1-yl(octyl)sulfane (3ag)

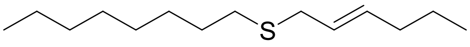

Following the general procedure, (*E*)-hex-2-en-1-yl methyl carbonate (**2g**) (175 µL, 1.03 mmol) and 1-octanethiol (150 µL, 0.861 mmol) were reacted in the presence of Pd(dba)$_2$ (9.9 mg, 17.2 µmol) and BIPHEPHOS (13.5 mg,

17.2 µmol) in 3.0 mL anhydrous CH_3CN. Purification via flash column chromatography (25 g SiO_2, 18.0 x 2.0 cm, cyclohexane) afforded the desired compound as a yellow oil (195 mg, 99 %).

$C_{14}H_{28}S$ [228.43 g·mol^{-1}]
R_f = 0.46 (cyclohexane, CAM)
GC-MS (method: 50S): t_R = 6.32 min; *m/z* (%) = 228 (7), 82 (100), 67 (49), 55 (75).
^{1}H NMR (300 MHz, $CDCl_3$): δ = 5.53-5.37 (m, 2H), 3.08 (d, *J* = 6.6 Hz, 2H), 2.43 (t, *J* = 7.4 Hz, 2H), 2.05-1.99 (m, 2H), 1.62-1.27 (m, 14H), 0.93-0.85 (m, 6H).
^{13}C NMR (76 MHz, $CDCl_3$): δ = 133.3, 126.3, 34.3, 33.9, 31.8, 30.6, 29.4, 29.2, 29.2, 28.9, 22.6, 22.5, 14.0, 13.6.
HRMS (EI-DI): calcd. for $C_{14}H_{28}S^+$ [M]$^+$: 228.1912; found: 228.1920.

(*E*)-(1,3-Diphenylallyl)(octyl)sulfane (3ah)

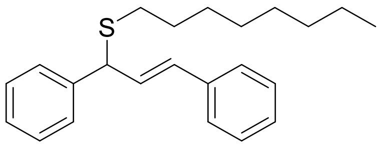

Following the general procedure, (*E*)-1,3-diphenylallyl methyl carbonate (**2h**) (90.2 mg, 0.36 mmol) and 1-octanethiol (43.7 mg, 0.30 mmol) were reacted in the presence of $Pd(dba)_2$ (3.7 mg, 6.4 µmol) and BIPHEPHOS (4.8 mg, 6.1 µmol) in 1.0 mL anhydrous CH_3CN. Purification via flash column chromatography (10 g SiO_2, 11.5 x 2.0 cm, cyclohexane) afforded the desired compound as a yellow oil (52 mg, 51 %).

$C_{23}H_{30}S$ [338.55 g·mol^{-1}]
R_f = 0.19 (cyclohexane, $KMnO_4$)
^{1}H NMR (300 MHz, $CDCl_3$): δ = 7.65-7.03 (m, 10H), 6.56-6.27 (m, 2H), 4.58 (d, *J* = 8.1 Hz, 1H), 2.59-2.36 (m, 2H), 1.73-1.50 (m, 2H), 1.47-1.03 (m, 10H), 0.86 (t, *J* = 5.4 Hz, 3H).
^{13}C NMR (76 MHz, $CDCl_3$): δ = 141.0, 136.8, 130.9, 130.0, 128.8, 128.7, 128.0, 127.7, 127.5, 126.6, 52.4, 31.9, 31.9, 29.5, 29.3, 29.3, 29.1, 22.8, 14.2.
HRMS (EI-DI): calcd. for $C_{23}H_{30}S^+$ [M]$^+$: 338.2068; found: 338.2066.

Thiol Scope

(3-Methylbut-2-en-1-yl)(phenyl)sulfane (3ba)

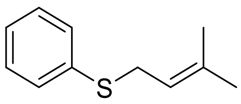

Following the general procedure, methyl prenyl carbonate (**2a**) (252 mg, 1.75 mmol) and benzenethiol (150 µL, 1.46 mmol) were reacted in the presence of $Pd(dba)_2$ (16.8 mg, 29.2 µmol) and BIPHEPHOS (23.0 mg, 29.2 µmol) in 5.0 mL anhydrous CH_3CN. Purification via flash column chromatography (35 g SiO_2, 23.0 x 2.5 cm, *n*-pentane) afforded the desired compound as a colorless oil (247 mg, 95 %).

$C_{11}H_{14}S$ [178.29 g·mol^{-1}]
R_f = 0.34 (cyclohexane, CAM)

GC-MS (method: 50S): t_R = 5.23 min; *m/z* (%) = 178 (38), 110 (100), 69 (66), 65 (17).

^{1}H NMR (300 MHz, $CDCl_3$): δ = 7.35 (d, *J* = 7.2 Hz, 2H), 7.27 (t, *J* = 7.4 Hz, 2H), 7.18 (t, *J* = 7.1 Hz, 1H), 5.32 (t, *J* = 7.6 Hz, 1H), 3.55 (d, *J* = 7.6 Hz, 2H), 1.73 (s, 3H), 1.60 (s, 3H).

^{13}C NMR (76 MHz, $CDCl_3$): δ = 137.0, 136.4, 129.8, 128.8, 126.0, 119.4, 32.3, 25.7, 17.8.

HRMS (EI-DI): calcd. for $C_{11}H_{14}S^+$ $[M]^+$: 178.0816; found: 178.0817.

(3-Methylbut-2-en-1-yl)(*p*-tolyl)sulfane (3ca)

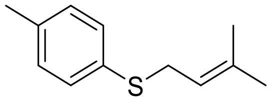

Following the general procedure, methyl prenyl carbonate (**2a**) (104 mg, 0.72 mmol) and 4-methylbenzenethiol (74.1 mg, 0.60 mmol) were reacted in the presence of $Pd(dba)_2$ (7.1 mg, 12 µmol) and BIPHEPHOS (9.4 mg, 12 µmol) in 2.0 mL anhydrous CH_3CN. Purification via flash column chromatography (5.5 g SiO_2, 12.0 x 1.0 cm, cyclohexane) afforded the desired compound as a colorless oil (114.2 mg, 99 %).

$C_{12}H_{16}S$ [192.32 g·mol^{-1}]

R_f = 0.23 (cyclohexane, CAM)

GC-MS (method: 50S): t_R = 5.54 min; *m/z* (%) = 192 (27), 124 (100), 91 (29), 69 (35).

^{1}H NMR (300 MHz, $CDCl_3$): δ = 7.23 (d, *J* = 8.0 Hz, 2H), 7.05 (d, *J* = 7.8 Hz, 2H), 5.27 (t, *J* = 7.1 Hz, 1H), 3.47 (d, *J* = 7.6 Hz, 2H), 2.29 (s, 3H), 1.68 (s, 3H), 1.53 (s, 3H).

^{13}C NMR (76 MHz, $CDCl_3$): δ = 136.2, 136.1, 133.1, 130.6, 129.6, 119.7, 33.0, 25.7, 21.1, 17.7.

HRMS (EI-DI): calcd. for $C_{12}H_{16}S^+$ $[M]^+$: 192.0973; found: 192.0967.

(2,6-Dimethylphenyl)(3-methylbut-2-en-1-yl)sulfane (3da)

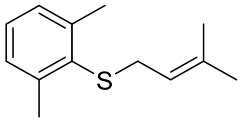

Following the general procedure, methyl prenyl carbonate (**2a**) (104 mg, 0.72 mmol) and 2,6-dimethylbenzenethiol (83.6 mg, 0.61 mmol) were reacted in the presence of $Pd(dba)_2$ (6.9 mg, 12 µmol) and BIPHEPHOS (9.4 mg, 12 µmol) in 2.0 mL anhydrous CH_3CN. Purification via flash column chromatography (5 g SiO_2, 16.5 x 1.0 cm, cyclohexane) afforded the desired compound as a colorless oil (114.2 mg, 92 %).

$C_{13}H_{18}S$ [206.35 g·mol^{-1}]

R_f = 0.57 (cyclohexane, CAM)

GC-MS (method: 50S): t_R = 5.67 min; *m/z* (%) = 206 (28), 138 (100), 105 (32), 69 (58).

^{1}H NMR (300 MHz, $CDCl_3$): δ = 7.26-7.03 (m, 3H), 5.33 (t, *J* = 8.1 Hz, 1H), 3.33 (d, *J* = 8.0 Hz, 2H), 2.62 (s, 6H), 1.72 (s, 3H), 1.44 (s, 3H).

^{13}C NMR (76 MHz, $CDCl_3$): δ = 143.6, 135.6, 133.5, 128.3, 128.0, 120.0, 33.3, 25.7, 22.2, 17.2.

HRMS (EI-DI): calcd. for $C_{13}H_{18}S^+$ $[M]^+$: 206.1129; found: 206.1130.

4-((3-Methylbut-2-en-1-yl)thio)phenol (3ea)

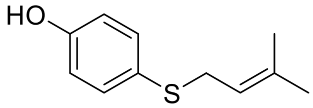

Following the general procedure, methyl prenyl carbonate (**2a**) (104 mg, 0.72 mmol) and 4-mercaptophenol (76.8 mg, 0.61 mmol) were reacted in the presence of Pd(dba)$_2$ (6.8 mg, 12 µmol) and BIPHEPHOS (9.3 mg, 12 µmol) in 2.0 mL anhydrous CH_3CN. Purification via flash column chromatography (20 g SiO_2, 19.0 x 2.0 cm, cyclohexane:EtOAc = 20:1 (v/v)) afforded the desired compound as a slightly yellowish oil (101.9 mg, 88 %).

$C_{11}H_{14}OS$ [194.29 g·mol^{-1}]
R_f = 0.09 (cyclohexane:EtOAc = 20:1 (v/v), CAM)
GC-MS (method: 50S): t_R = 6.15 min; *m/z* (%) = 194 (26), 126 (100), 97 (16), 69 (32).
^{1}H NMR (300 MHz, $CDCl_3$): δ = 7.20 (d, *J* = 8.5 Hz, 2H), 6.68 (d, *J* = 8.5 Hz, 2H), 5.94 (br s, 1H), 5.17 (t, *J* = 7.7 Hz, 1H), 3.33 (d, *J* = 7.8 Hz, 2H), 1.59 (s, 3H), 1.36 (s, 3H).
^{13}C NMR (76 MHz, $CDCl_3$): δ = 155.2, 136.2, 134.4, 126.4, 119.7, 116.0, 34.5, 25.7, 17.6.
HRMS (EI-DI): calcd. for $C_{11}H_{14}OS^+$ [M]$^+$: 194.0765; found: 194.0762.

(4-Methoxyphenyl)(3-methylbut-2-en-1-yl)sulfane (3fa)

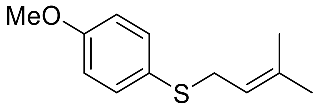

Following the general procedure, methyl prenyl carbonate (**2a**) (103.2 mg, 0.72 mmol) and 4-methoxybenzenethiol (84.3 mg, 0.60 mmol) were reacted in the presence of Pd(dba)$_2$ (6.7 mg, 12 µmol) and BIPHEPHOS (9.5 mg, 12 µmol) in 2.0 mL anhydrous CH_3CN. Purification via flash column chromatography (15 g SiO_2, 21.0 x 1.5 cm, cyclohexane to cyclohexane:EtOAc = 80:1 (v/v)) afforded the desired compound as a colorless oil (112.9 mg, 90 %).

$C_{12}H_{16}OS$ [208.32 g·mol^{-1}]
R_f = 0.09 (cyclohexane, CAM)
GC-MS (method: 50S): t_R = 5.97 min; *m/z* (%) = 208 (24), 140 (100), 125 (22), 69 (20).
^{1}H NMR (300 MHz, $CDCl_3$): δ = 7.32 (d, *J* = 8.7 Hz, 2H), 6.80 (d, *J* = 8.7 Hz, 2H), 5.25 (t, *J* = 7.7 Hz, 1H), 3.75 (s, 3H), 3.40 (d, *J* = 7.7 Hz, 2H), 1.67 (s, 3H), 1.44 (s, 3H).
^{13}C NMR (76 MHz, $CDCl_3$): δ = 159.1, 135.8, 134.0, 126.7, 120.0, 114.4, 55.3, 34.4, 25.7, 17.6.
HRMS (EI-DI): calcd. for $C_{12}H_{16}OS^+$ [M]$^+$: 208.0922; found: 208.0928.

4-((3-Methylbut-2-en-1-yl)thio)aniline (3ga)

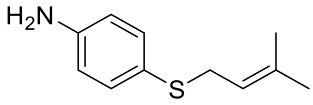

Following the general procedure, methyl prenyl carbonate (**2a**) (102.9 mg, 0.71 mmol) and 4-aminobenzenethiol (75.0 mg, 0.60 mmol) were reacted in the presence of Pd(dba)$_2$ (6.9 mg, 12 µmol) and BIPHEPHOS (9.5 mg,

12 µmol) in 2.0 mL anhydrous CH_3CN. Purification via flash column chromatography (15 g SiO_2, 21.0 x 1.5 cm, cyclohexane:EtOAc = 10:1 (v/v) + 1 vol% NEt_3) afforded the desired compound as a pale yellow oil (77.8 mg, 67 %).

$C_{11}H_{15}NS$ [193.31 g·mol^{-1}]
R_f = 0.19 (cyclohexane:EtOAc = 10:1 (v/v) + 1 vol% NEt_3, CAM)
GC-MS (method: 50S): t_R = 6.26 min; *m/z* (%) = 193 (27), 125 (100), 80 (14), 69 (10).
^{1}H NMR (300 MHz, $CDCl_3$): δ = 7.23 (d, *J* = 8.4 Hz, 2H), 6.60 (d, *J* = 8.4 Hz, 2H), 5.26 (t, *J* = 7.8 Hz, 1H), 3.69 (br s, 2H), 3.38 (d, *J* = 7.8 Hz, 2H), 1.69 (s, 3H), 1.44 (s, 3H).
^{13}C NMR (76 MHz, $CDCl_3$): δ = 146.1, 135.7, 134.8, 123.6, 120.2, 115.5, 35.0, 25.8, 17.6.
HRMS (EI-DI): calcd. for $C_{11}H_{15}NS^+$ [M]$^+$: 193.0925; found: 193.0916.

(4-Chlorophenyl)(3-methylbut-2-en-1-yl)sulfane (3ha)

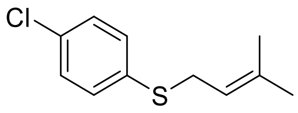

Following the general procedure, methyl prenyl carbonate (**2a**) (105 mg, 0.73 mmol) and 4-chlorobenzenethiol (86.7 mg, 0.60 mmol) were reacted in the presence of $Pd(dba)_2$ (6.9 mg, 12 µmol) and BIPHEPHOS (9.5 mg, 12 µmol) in 2.0 mL anhydrous CH_3CN. Purification via flash column chromatography (10 g SiO_2, 12.0 x 2.0 cm, cyclohexane) afforded the desired compound as a colorless oil (104 mg, 82 %).

$C_{11}H_{13}ClS$ [212.74 g·mol^{-1}]
R_f = 0.34 (cyclohexane, CAM)
GC-MS (method: 50S): t_R = 5.80 min; *m/z* (%) = 212 (33), 144 (81), 108 (36), 69 (100).
^{1}H NMR (300 MHz, $CDCl_3$): δ = 7.32-7.10 (m, 4H), 5.25 (t, *J* = 7.7 Hz, 1H), 3.49 (d, *J* = 7.6 Hz, 2H), 1.69 (s, 3H), 1.55 (s, 3H).
^{13}C NMR (76 MHz, $CDCl_3$): δ = 136.8, 135.4, 132.1, 131.3, 128.9, 119.2, 32.6, 25.7, 17.8.
HRMS (EI-DI): calcd. for $C_{11}H_{13}ClS^+$ [M]$^+$: 212.0426; found: 212.0432.

(4-Fluorophenyl)(3-methylbut-2-en-1-yl)sulfane (3ia)

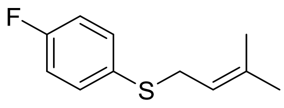

Following the general procedure, methyl prenyl carbonate (**2a**) (104 mg, 0.72 mmol) and 4-fluorobenzenethiol (77.0 mg, 0.60 mmol) were reacted in the presence of $Pd(dba)_2$ (6.8 mg, 12 µmol) and BIPHEPHOS (9.4 mg, 12 µmol) in 2.0 mL anhydrous CH_3CN. Purification via flash column chromatography (17.5 g SiO_2, 17.5 x 2.0 cm, cyclohexane) afforded the desired compound as a colorless oil (117 mg, 99 %).

$C_{11}H_{13}FS$ [196.28 g·mol^{-1}]
R_f = 0.40 (cyclohexane, CAM)
GC-MS (method: 50S): t_R = 5.13 min; *m/z* (%) = 196 (44), 128 (97), 83 (41), 69 (100).
^{1}H NMR (300 MHz, $CDCl_3$): δ = 7.45-7.20 (m, 2H), 6.97 (t, *J* = 8.6 Hz, 2H), 5.26 (t, *J* = 7.7 Hz, 1H), 3.47 (d, *J* = 7.7 Hz, 2H), 1.70 (s, 3H), 1.49 (s, 3H).

^{13}C NMR (76 MHz, $CDCl_3$): δ = 162.1 (d, *J* = 246.3 Hz), 136.5, 133.4 (d, *J* = 8.0 Hz), 131.4 (d, *J* = 3.3 Hz), 119.5, 115.8 (d, *J* = 21.8 Hz), 33.7, 25.7, 17.7.
^{19}F NMR (470 MHz, $CDCl_3$): δ = -115.6 (m).
HRMS (EI-DI): calcd. for $C_{11}H_{13}FS^+$ $[M]^+$: 196.0722; found: 196.0723.

(3-Methylbut-2-en-1-yl)(4-(trifluoromethyl)phenyl)sulfane (3ja)

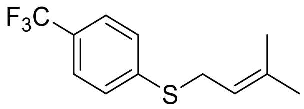

Following the general procedure, methyl prenyl carbonate (**2a**) (104 mg, 0.72 mmol) and 4-(trifluoromethyl)benzenethiol (108.3 mg, 0.61 mmol) were reacted in the presence of $Pd(dba)_2$ (7.3 mg, 12 µmol) and BIPHEPHOS (9.5 mg, 12 µmol) in 2.0 mL anhydrous CH_3CN. Purification via flash column chromatography (22.5 g SiO_2, 20.5 x 2.0 cm, cyclohexane) afforded the desired compound as a colorless oil (127.9 mg, 85 %).

$C_{12}H_{13}F_3S$ [246.29 g·mol^{-1}]
R_f = 0.38 (cyclohexane, CAM)
GC-MS (method: 50S): t_R = 5.20 min; *m/z* (%) = 246 (33), 177 (22), 157 (23), 69 (100).
^{1}H NMR (300 MHz, $CDCl_3$): δ = 7.50 (d, *J* = 8.2 Hz, 2H), 7.35 (d, *J* = 8.2 Hz, 2H), 5.30 (t, *J* = 7.5 Hz, 1H), 3.60 (d, *J* = 7.5 Hz, 2H), 1.74 (s, 3H), 1.67 (s, 3H).
^{13}C NMR (126 MHz, $CDCl_3$): δ = 142.9 (q, *J* = 1.2 Hz), 137.4, 128.0, 127.6 (q, *J* = 32.7 Hz), 125.6 (q, *J* = 3.8 Hz), 124.4 (q, *J* = 271.6 Hz), 118.6, 31.2, 25.8, 18.0.
^{19}F NMR (470 MHz, $CDCl_3$): δ = -62.4.
HRMS (EI-DI): calcd. for $C_{12}H_{13}F_3S^+$ $[M]^+$: 246.0690; found: 246.0690.

4-((3-Methylbut-2-en-1-yl)thio)benzoic acid (3ka)

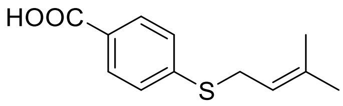

Following the general procedure, methyl prenyl carbonate (**2a**) (104 mg, 0.72 mmol) and 4-mercaptobenzoic acid (92.5 mg, 0.60 mmol) were reacted in the presence of $Pd(dba)_2$ (7.0 mg, 12 µmol) and BIPHEPHOS (9.5 mg, 12 µmol) in 2.0 mL anhydrous CH_3CN. Purification via flash column chromatography (20 g SiO_2, 21.0 x 2.0 cm, cyclohexane:EtOAc = 8:1 (v/v) + 1 vol% AcOH to 5:1 (v/v) + 1 vol% AcOH) afforded the desired compound as slightly yellowish solid (102.1 mg, 77 %).

$C_{12}H_{14}O_2S$ [222.30 g·mol^{-1}]
R_f = 0.16 (cyclohexane:EtOAc = 8:1 (v/v) + 1 vol% AcOH, CAM)
m.p. = 130 °C
^{1}H NMR (300 MHz, DMSO-*d*$_6$): δ = 12.86 (br s, 1H), 7.83 (d, *J* = 8.3 Hz, 2H), 7.36 (d, *J* = 8.3 Hz, 2H), 5.27 (t, *J* = 6.9 Hz, 1H), 3.69 (d, *J* = 7.4 Hz, 2H), 1.67 (s, 6H).
^{13}C NMR (76 MHz, DMSO-*d*$_6$): δ = 167.0, 143.6, 136.6, 129.7, 127.2, 126.4, 118.5, 29.5, 25.3, 17.7.
HRMS (EI-DI): calcd. for $C_{12}H_{14}O_2S^+$ $[M]^+$: 222.0715; found: 222.0708.

2-Methyl-3-((3-methylbut-2-en-1-yl)thio)-1,2-dihydro-1,2,4-triazine-5,6-dione (3la)

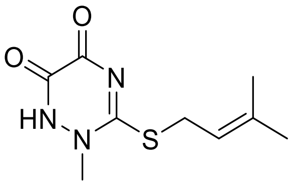

Following the general procedure, methyl prenyl carbonate (**2a**) (104 mg, 0.72 mmol) and 3-mercapto-2-methyl-1,2-dihydro-1,2,4-triazine-5,6-dione (95.5 mg, 0.60 mmol) were reacted in the presence of $Pd(dba)_2$ (6.7 mg, 12 µmol) and BIPHEPHOS (9.5 mg, 12 µmol) in 2.0 mL anhydrous CH_3CN **at 35 °C**. Purification via flash column chromatography (8 g SiO_2, 11.5 x 1.0 cm, CH_2Cl_2:CH_3OH = 20:1 (v/v)) afforded the desired compound as a red solid (93.1 mg, 68 %).

$C_9H_{13}N_3O_2S$ [227.28 g·mol^{-1}]
R_f = 0.16 (CH_2Cl_2:CH_3OH = 20:1 (v/v), CAM)
m.p. = 176 °C
^{1}H NMR (300 MHz, $CDCl_3$): δ = 6.55 (s, 1H), 5.31 (s, 1H), 3.89 (d, *J* = 7.3 Hz, 2H), 3.70 (s, 3H), 1.73 (s, 6H).
^{13}C NMR (76 MHz, $CDCl_3$): δ = 164.1, 157.5, 153.1, 139.7, 116.5, 43.1, 30.8, 25.8, 18.1.
HRMS (EI-DI): calcd. for $C_9H_{13}N_3O_2S^+$ [M]$^+$: 227.0728; found: 227.0720.

2-((3-Methylbut-2-en-1-yl)thio)benzo[*d*]thiazole (3ma)

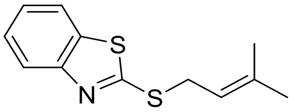

Following the general procedure, methyl prenyl carbonate (**2a**) (105.8 mg, 0.73 mmol) and benzo[*d*]thiazole-2-thiol (100.5 mg, 0.60 mmol) were reacted in the presence of $Pd(dba)_2$ (6.8 mg, 12 µmol) and BIPHEPHOS (9.5 mg, 12 µmol) in 2.0 mL anhydrous CH_3CN **at 35 °C**. Purification via flash column chromatography (20 g SiO_2, 24.5 x 2.0 cm, cyclohexane:EtOAc = 100:1 (v/v)) afforded the desired compound as a colorless oil (94.7 mg, 67 %).

$C_{12}H_{13}NS_2$ [235.36 g·mol^{-1}]
R_f = 0.18 (cyclohexane:EtOAc = 100:1 (v/v), $KMnO_4$)
GC-MS (method: 50S): t_R = 6.37 min; *m/z* (%) = 235 (22), 202 (31), 167 (100), 69 (27).
^{1}H NMR (300 MHz, $CDCl_3$): δ = 7.84 (d, *J* = 8.1 Hz, 1H), 7.70 (d, *J* = 7.9 Hz, 1H), 7.37 (t, *J* = 7.6 Hz, 1H), 7.24 (t, *J* = 7.6 Hz, 1H), 5.39 (t, *J* = 7.8 Hz, 1H), 3.97 (d, *J* = 7.8 Hz, 2H), 1.73 (s, 6H).
^{13}C NMR (76 MHz, $CDCl_3$): δ = 167.1, 153.4, 138.5, 135.4, 126.0, 124.2, 121.5, 121.0, 117.9, 31.9, 25.8, 18.0.
HRMS (EI-DI): calcd. for $C_{12}H_{13}NS_2^+$ [M]$^+$: 235.0490; found: 235.0484.

2-Methyl-5-((3-methylbut-2-en-1-yl)thio)-1,3,4-thiadiazole (3na)

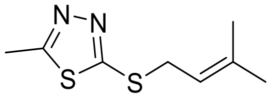

Following the general procedure, methyl prenyl carbonate (**2a**) (104 mg, 0.72 mmol) and 5-methyl-1,3,4-thiadiazole-2-thiol (79.3 mg, 0.60 mmol) were reacted in the presence of $Pd(dba)_2$ (6.8 mg, 12 µmol) and BIPHEPHOS (9.3 mg, 12 µmol) in 2.0 mL anhydrous CH_3CN **at 35 °C**. Purification via flash column chromatography (17.5 g SiO_2, 15.5 x 2.0 cm, cyclohexane:EtOAc = 10:1 (v/v)) afforded the desired compound as a colorless oil (97.4 mg, 81 %).

$C_8H_{12}N_2S_2$ [200.32 g·mol^{-1}]
R_f = 0.16 (cyclohexane:EtOAc = 10:1 (v/v), CAM)
GC-MS (method: 50S): t_R = 5.96 min; *m/z* (%) = 200 (18), 167 (31), 132 (100), 69 (44).
^{1}H NMR (300 MHz, $CDCl_3$): δ = 5.33 (t, *J* = 7.4 Hz, 1H), 3.87 (d, *J* = 7.8 Hz, 2H), 2.67 (s, 3H), 1.70 (s, 3H), 1.67 (s, 3H).
^{13}C NMR (76 MHz, $CDCl_3$): δ = 165.5, 164.9, 138.8, 117.7, 32.5, 25.7, 17.9, 15.6.
HRMS (Ei-DI): calcd. for $C_8H_{12}N_2S_2^+$ [M]$^+$: 200.0442; found: 200.0440.

1-Methyl-5-((3-methylbut-2-en-1-yl)thio)-1*H*-tetrazole (3oa)

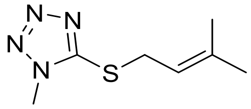

Following the general procedure, methyl prenyl carbonate (**2a**) (104 mg, 0.72 mmol) and 1-methyl-1*H*-tetrazole-5-thiol (69.7 mg, 0.60 mmol) were reacted in the presence of $Pd(dba)_2$ (6.8 mg, 12 µmol) and BIPHEPHOS (9.4 mg, 12 µmol) in 2.0 mL anhydrous CH_3CN **at 35 °C**. Purification via flash column chromatography (15 g SiO_2, 19.5 x 1.5 cm, cyclohexane:EtOAc = 8:1 (v/v)) afforded the desired compound as a colorless oil (88.7 mg, 80 %).

$C_7H_{12}N_4S$ [184.26 g·mol^{-1}]
R_f = 0.12 (cyclohexane:EtOAc = 8:1 (v/v), CAM)
GC-MS (method: 50S): t_R = 5.90 min; *m/z* (%) = 184 (21), 123 (70), 116 (76), 69 (100).
^{1}H NMR (300 MHz, $CDCl_3$): δ = 5.33 (t, *J* = 7.9 Hz, 1H), 3.91 (d, *J* = 7.9 Hz, 2H), 3.86 (s, 3H), 1.69 (s, 3H), 1.65 (s, 3H).
^{13}C NMR (76 MHz, $CDCl_3$): δ = 154.2, 139.5, 117.3, 33.5, 31.8, 25.7, 17.8.
HRMS (EI-DI): calcd. for $C_7H_{12}N_4S^+$ [M]$^+$: 184.0783; found: 184.0778.

Cbz-Cys(prenyl)-OMe (3pa)

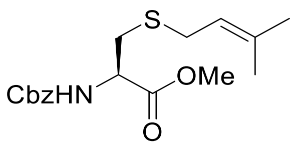

Following the general procedure, methyl prenyl carbonate (**2a**) (85 µL, 0.668 mmol) and Cbz-Cys-OMe (**1p**) (150 mg, 0.557 mmol) were reacted in the presence of Pd(dba)$_2$ (6.4 mg, 11 µmol) and BIPHEPHOS (8.8 mg, 11 µmol) in 2.0 mL anhydrous CH_3CN. Purification via flash column chromatography (20 g SiO_2, 14.0 x 2.0 cm, cyclohexane:EtOAc = 6:1 (v/v)) afforded the desired compound as a yellow oil (151 mg, 80 %).

$C_{17}H_{23}NO_4S$ [337.43 g·mol^{-1}]
R_f = 0.30 (cyclohexane:EtOAc = 6:1, CAM)
GC-MS (method: 50S): t_R = 8.51 min; *m/z* (%) = 202 (34), 146 (14), 91 (100), 69 (32).
$[\alpha]_D^{20}$ = - 38.3 (1.0, CH_3CN)
^{1}H NMR (300 MHz, $CDCl_3$): δ = 7.35-7.33 (m, 5H), 5.58 (d, *J* = 7.8 Hz, 1H), 5.18 (t, *J* = 7.8 Hz, 1H), 5.12 (s, 2H), 4.59 (dt, *J* = 7.8 Hz, 5.3 Hz, 1H), 3.76 (s, 3H), 3.14 (t, *J* = 7.5 Hz, 2H), 2.91 (t, *J* = 5.4 Hz, 2H), 1.72 (s, 3H), 1.64 (s, 3H).
^{13}C NMR (76 MHz, $CDCl_3$): δ = 171.6, 156.0, 136.6, 136.4, 128.8, 128.4, 128.3, 120.0, 67.3, 53.9, 52.8, 33.9, 30.4, 25.9, 18.0.
HRMS (EI-DI): calcd. for $C_{17}H_{23}NO_4S^+$ [M]$^+$: 337.1348; found: 337.1368.

Cbz-γ-Glu(OMe)-Cys(farnesyl)-Gly-OMe (3qc)

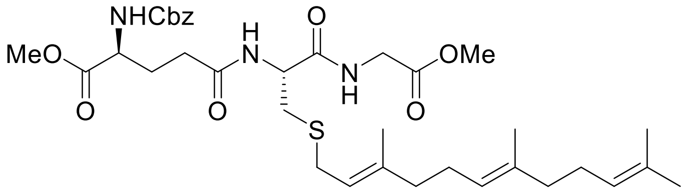

Following the general procedure, methyl ((2*E*,6*E*)-3,7,11-trimethyldodeca-2,6,10-trien-1-yl) carbonate (**2c**) (40 µL, 0.134 mmol) and Cbz-γ-Glu(OMe)-Cys-Gly-OMe (**1q**) (52.3 mg, 0.111 mmol) were reacted in the presence of Pd(dba)$_2$ (1.3 mg, 2.2 µmol) and BIPHEPHOS (1.8 mg, 2.2 µmol) in 5.4 mL anhydrous CH_3CN. Purification via flash column chromatography (10 g SiO_2, 20.0 x 1.5 cm, CH_2Cl_2:CH_3OH = 29:1 (v/v)) afforded the desired compound as an off-white solid (66.0 mg, 88 %).

$C_{35}H_{51}N_3O_8S$ [673.86 g·mol^{-1}]
R_f = 0.19 (CH_2Cl_2:CH_3OH = 29:1 (v/v), CAM)
m.p. = 96 °C
$[\alpha]_D^{20}$ = - 29.0 (0.25, CH_3OH)
^{1}H NMR (300 MHz, $CDCl_3$): δ = 7.45-7.29 (m, 5H), 7.02 (t, *J* = 5.1 Hz, 1H), 6.66 (d, *J* = 7.1 Hz, 1H), 5.71 (d, *J* = 7.6 Hz, 1H), 5.24 (t, *J* = 7.4 Hz, 1H), 5.17-5.03 (m, 4H), 4.57 (q, *J* = 6.7 Hz, 1H), 4.50-4.37 (m, 1H), 4.11-3.91 (m, 2H), 3.74 (s, 3H), 3.73 (s, 3H), 3.29-3.13 (m, 2H), 2.95-2.77 (m, 2H), 2.41-2.30 (m, 2H), 2.30-2.15 (m, 1H), 2.15-1.90 (m, 9H), 1.67 (s, 6H), 1.59 (s, 6H).

^{13}C NMR (76 MHz, $CDCl_3$): δ = 172.6, 172.2, 170.7, 170.1, 156.4, 140.2, 136.3, 135.5, 131.5, 128.7, 128.4, 128.3, 124.5, 123.9, 119.7, 67.3, 53.4, 52.7, 52.6, 52.5, 41.4, 39.8, 39.8, 33.1, 32.2, 30.1, 28.4, 26.9, 26.6, 25.8, 17.8, 16.3, 16.2.
HRMS (MALDI-TOF): calcd. for $C_{35}H_{51}N_3O_8SNa^+$ $[M+Na]^+$: 696.2395; found: 669.2359.

_S_-(3-Methylbut-2-en-1-yl)benzothioate (3ra)

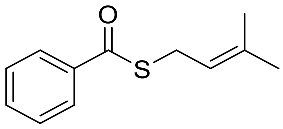

Following the general procedure, methyl prenyl carbonate (**2a**) (102.8 mg, 0.71 mmol) and thiobenzoic acid (82.5 mg, 0.60 mmol) were reacted in the presence of $Pd(dba)_2$ (7.1 mg, 12 µmol) and BIPHEPHOS (9.2 mg, 12 µmol) in 2.0 mL anhydrous CH_3CN. Purification via flash column chromatography (10 g SiO_2, 17.0 x 1.5 cm, cyclohexane:toluene = 200:1 (v/v) to 50:1 (v/v)) afforded the desired compound as a colorless oil (41.0 mg, 33 %).

$C_{12}H_{14}OS$ [206.30 g·mol^{-1}]
R_f = 0.18 (cyclohexane:toluene = 200:1 (v/v), $KMnO_4$)
GC-MS (method: 50S): t_R = 6.06 min; *m/z* (%) = 206 (11), 105 (100), 77 (31), 69 (12), 51 (9).
^1H NMR (300 MHz, $CDCl_3$): δ = 7.96 (d, *J* = 7.2 Hz, 2H), 7.56 (t, *J* = 7.3 Hz, 1H), 7.44 (t, *J* = 7.5 Hz, 2H), 5.31 (t, *J* = 7.9 Hz, 1H), 3.73 (d, *J* = 7.8 Hz, 2H), 1.75 (s, 6H).
^{13}C NMR (76 MHz, $CDCl_3$): δ = 192.3, 137.3, 137.1, 133.4, 128.7, 127.3, 118.7, 27.5, 25.8, 18.0.
HRMS (EI-DI): calcd. for $C_{12}H_{14}OS^+$ $[M]^+$: 206.0765; found: 206.0760.

(2-Methylbut-3-en-2-yl)(phenyl)sulfane (_i_-3ba)

This compound was prepared according to a procedure described by Zaitsev et al.[18]

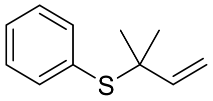

In a flame-dried and argon-flushed Schlenk flask, equipped with a Teflon-coated magnetic stirring bar, $[RuCp^*(CH_3CN)_3]PF_6$ (37.8 mg, 74.9 µmol) and (1*S*)-(+)-10-camphorsulfonic acid (17.4 mg, 74.9 µmol) were dissolved in anhydrous CH_3CN (10 mL). Subsequently, 2-methylbut-3-en-2-ol (165 µL, 1.58 mmol) and benzenethiol (154 µL, 1.50 mmol) were added and the resulting mixture was stirred at rt until complete consumption of the starting material (according to GC-MS, 1.5 h). The reaction mixture was cooled to rt and concentrated under reduced pressure. The crude product was dissolved in CH_2Cl_2 and filtered through a pad of Celite (2.0 cm x 3.5 cm) and afterwards through basic SiO_2 (2.0 cm x 3.5 cm). The filtrate was concentrated under reduced pressure to afford the desired product as a yellow-orange oil (178.2 mg, 67 %).

$C_{11}H_{14}S$ [178.29 g·mol^{-1}]
GC-MS (method: 50S): t_R = 5.03 min; *m/z* (%) = 178 (16), 110 (100), 69 (86).
^1H NMR (300 MHz, $CDCl_3$): δ = 7.58-7.41 (m, 2H), 7.41-7.11 (m, 3H), 5.96 (dd, *J* = 17.3, 10.6 Hz, 1H), 4.91 (d, *J* = 10.5 Hz, 1H), 4.72 (d, *J* = 17.4 Hz, 1H), 1.36 (s, 6H).
^{13}C NMR (76 MHz, $CDCl_3$): δ = 144.9, 137.3, 132.6, 128.8, 128.4, 111.7, 50.1, 27.6.
HRMS (EI-DI): calcd. for $C_{11}H_{14}S^+$ $[M]^+$: 178.0816; found: 178.0816.

Allyl(*p*-tolyl)sulfane (3ci)

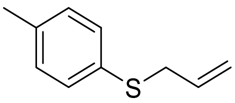

In a 10 mL round-bottom flask, equipped with a Teflon-coated magnetic stirring bar, 4-methylbenzenethiol (186.6 mg, 1.50 mmol) was dissolved in THF (5 mL). Subsequently, KO*t*Bu (185.2 mg, 1.65 mmol) and allyl bromide (130 µL, 1.54 mmol) were added. The resulting suspension was stirred at rt until complete consumption of the starting material (according to GC-MS, 1 h) and concentrated under reduced pressure. The crude product was suspended in CH_3OH (5 mL) and adsorbed on 1.5 g SiO_2. Purification via flash column chromatography (25 g SiO_2, 8.5 x 3.0 cm, cyclohexane) afforded the desired compound as a colorless oil (176.6 mg, 72 %).

$C_{10}H_{12}S$ [164.27 g·mol^{-1}]
R_f = 0.35 (cyclohexane, $KMnO_4$)
GC-MS (method: 50S): t_R = 5.12 min; *m/z* (%) = 164 (100), 149 (47), 131 (47), 123 (69), 91 (31).
^{1}H NMR (300 MHz, $CDCl_3$): δ = 7.25 (d, *J* = 8.0 Hz, 2H), 7.09 (d, *J* = 7.9 Hz, 2H), 5.97-5.74 (m, 1H), 5.21-4.91 (m, 2H), 3.50 (d, *J* = 6.9 Hz, 2H), 2.31 (s, 3H).
^{13}C NMR (76 MHz, $CDCl_3$): δ = 136.6, 134.0, 132.2, 130.8, 129.7, 117.5, 38.0, 21.2.
HRMS (EI-DI): calcd. for $C_{10}H_{12}S^+$ [M]$^+$: 164.0660; found: 164.0662.

Natural Product *S*-Allylation

General Procedure for Cefalotin Derivatization

In a flame-dried and argon-flushed 10 mL Schlenk flask, equipped with a Teflon-coated magnetic stirring bar, $Pd(dba)_2$ and BIPHEPHOS were suspended in 2.0 mL anhydrous CH_3CN and stirred in a pre-heated oil bath at 60 °C for 30 min to obtain a bright yellow solution. Then cefalotin and thiol were added and the resulting mixture was stirred at the indicated reaction temperature until complete consumption of the starting material (according to HPLC-MS). The reaction mixture was cooled to rt and concentrated under reduced pressure. The crude product was purified via semi-preparative HPLC to afford the desired compound.

(6*R*,7*R*)-8-Oxo-7-(2-(thiophen-2-yl)acetamido)-3-((*p*-tolylthio)methyl)-5-thia-1-azabicyclo[4.2.0]oct-2-ene-2-carboxylic acid (5)

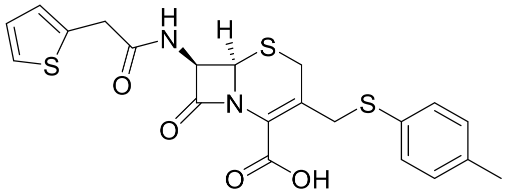

Following the general procedure, cefalotin (79.8 mg, 0.20 mmol) and 4-methylbenzenethiol (29.9 mg, 0.24 mmol) were reacted in the presence of $Pd(dba)_2$ (2.5 mg, 4 µmol) and BIPHEPHOS (3.4 mg, 4 µmol) in 2.0 mL anhydrous CH_3CN **at 40 °C**. Purification via semi-preparative HPLC afforded the desired compound as a colorless powder (37.9 mg, 41 %).

$C_{21}H_{20}N_2O_4S_3$ [460.58 g·mol^{-1}]
HPLC-MS (method: 2-100MeCN): t_R = 5.14 min; *m/z* (ESI+) = 483 [M+Na]$^+$.
m.p. = 98 °C
$[\alpha]_D^{20}$ = - 62.9 (0.24, $CHCl_3$)
^{1}H NMR (300 MHz, $CDCl_3$): δ = 7.38-7.15 (m, 3H), 7.06 (d, *J* = 7.8 Hz, 2H), 7.01-6.86 (m, 2H), 6.69 (d, *J* = 8.5 Hz, 1H), 6.17 (br s, 1H), 5.70 (dd, *J* = 8.5, 4.6 Hz, 1H), 4.87 (d, *J* = 4.6 Hz, 1H), 4.17 (d, *J* = 13.3 Hz, 1H), 3.90-3.73 (m, 3H), 3.60 (d, *J* = 18.1 Hz, 1H), 3.33 (d, *J* = 18.0 Hz, 1H), 2.28 (s, 3H).
^{13}C NMR (76 MHz, $CDCl_3$): δ = 170.8, 165.1, 164.0, 138.4, 134.9, 132.9, 132.4, 130.4, 130.1, 128.0, 127.6, 126.1, 124.1, 59.1, 58.0, 38.1, 37.1, 28.7, 21.3.
HRMS (MALDI-TOF): calcd. for $C_{21}H_{20}N_2O_4S_3Na^+$ [M+Na]$^+$: 483.0483; found: 483.0485.

(6*R*,7*R*)-3-(((4-Fluorophenyl)thio)methyl)-8-oxo-7-(2-(thiophen-2-yl)acetamido)-5-thia-1-azabicyclo[4.2.0]oct-2-ene-2-carboxylic acid (6)

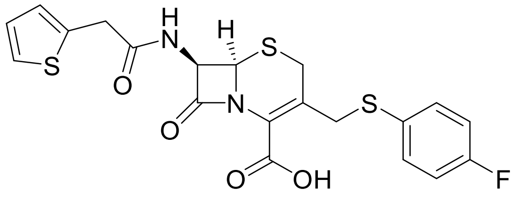

Following the general procedure, cefalotin (79.4 mg, 0.20 mmol) and 4-fluorobenzenethiol (31.0 mg, 0.24 mmol) were reacted in the presence of $Pd(dba)_2$ (2.3 mg, 4 µmol) and BIPHEPHOS (3.2 mg, 4 µmol) in 2.0 mL anhydrous CH_3CN **at 40 °C**. Purification via semi-preparative HPLC afforded the desired compound as a colorless powder (53.8 mg, 58 %).

$C_{20}H_{17}FN_2O_4S_3$ [464.54 g·mol^{-1}]
HPLC-MS (method: 2-100MeCN): t_R = 4.97 min; *m/z* (ESI+) = 487 [M+Na]$^+$.
m.p. = 85 °C
$[\alpha]_D^{20}$ = - 1.0 (0.99, CH_3OH)
^{1}H NMR (300 MHz, $CDCl_3$): δ = 7.35 (dd, *J* = 8.2, 5.3 Hz, 2H), 7.29-7.18 (m, 1H), 7.05-6.86 (m, 4H), 6.76 (d, *J* = 8.1 Hz, 1H), 6.63 (br s, 1H), 5.69 (dd, *J* = 8.3, 4.5 Hz, 1H), 4.88 (d, *J* = 4.6 Hz, 1H), 4.10 (d, *J* = 13.3 Hz, 1H), 3.97-3.70 (m, 3H), 3.60 (d, *J* = 18.0 Hz, 1H), 3.35 (d, *J* = 17.9 Hz, 1H).
^{13}C NMR (76 MHz, $CDCl_3$): δ = 170.9, 165.1, 163.9, 162.9 (d, *J* = 248.7 Hz), 135.3 (d, *J* = 8.3 Hz), 135.0, 131.6, 129.0 (d, *J* = 3.5 Hz), 128.0, 127.6, 126.1, 124.5, 116.5 (d, *J* = 21.9 Hz), 59.1, 58.0, 38.4, 37.1, 28.6.
^{19}F NMR (470 MHz, $CDCl_3$): δ = - 112.5.
HRMS (MALDI-TOF): calcd. for $C_{20}H_{17}FN_2O_4S_3Na^+$ [M+Na]$^+$: 487.0232; found: 487.0265.

***N*-((6*R*,7*R*)-3-((Octylthio)methyl)-8-oxo-5-thia-1-azabicyclo[4.2.0]oct-2-en-7-yl)-2-(thiophen-2-yl)acetamide (7)**

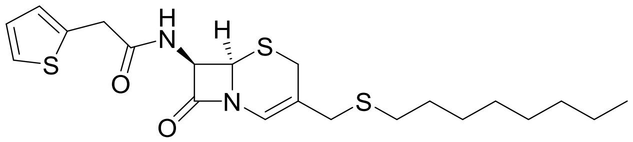

Following the general procedure, cefalotin (79.7 mg, 0.20 mmol) and 1-octanethiol (35.8 mg, 0.24 mmol) were reacted in the presence of $Pd(dba)_2$ (2.4 mg, 4 µmol) and BIPHEPHOS (3.3 mg, 4 µmol) in 2.0 mL anhydrous CH_3CN **at 60 °C**. Purification via flash column chromatography (3 g SiO_2, 8.0 x 1.0 cm, cyclohexane:EtOAc = 5:1 (v/v) + 3 vol% AcOH) afforded the desired compound as a yellowish solid (39.5 mg, 39 %).

$C_{21}H_{30}N_2O_2S_3$ [438.66 g·mol^{-1}]
R_f = 0.23 (cyclohexane:EtOAc = 5:1 (v/v) + 3 vol% AcOH, $KMnO_4$)
HPLC-MS (method: 2-100MeCN): t_R = 6.67 min; *m/z* (ESI+) = 461 [M+Na]$^+$.
m.p. = 128 °C
$[\alpha]_D^{20}$ = - 81.2 (0.99, $CHCl_3$)
^{1}H NMR (300 MHz, $CDCl_3$): δ = 7.26 (d, *J* = 3.4 Hz, 1H), 7.11-6.89 (m, 2H), 6.54 (s, 1H), 6.38 (d, *J* = 8.8 Hz, 1H), 5.76 (dd, *J* = 9.1, 4.7 Hz, 1H), 4.96 (d, *J* = 4.7 Hz, 1H), 3.85 (s, 2H), 3.50 (d, *J* = 17.2 Hz, 1H), 3.34 (d, *J* = 17.2 Hz,

1H), 3.16 (d, *J* = 21.1 Hz, 1H), 3.11 (d, *J* = 21.2 Hz, 1H), 2.37 (t, *J* = 7.2 Hz, 2H), 1.60-1.45 (m, 2H), 1.43-1.13 (m, 10H), 1.00-0.75 (m, 3H).

^{13}C NMR (76 MHz, $CDCl_3$): δ = 170.1, 163.7, 134.9, 127.9, 127.6, 126.1, 118.3, 118.2, 59.3, 57.8, 37.2 (2x C), 31.9, 31.3, 29.3, 29.3, 29.3, 28.9, 26.5, 22.7, 14.2.

HRMS (MALDI-TOF): calcd. for $C_{21}H_{30}N_2O_2S_3Na^+$ $[M+Na]^+$: 461.1367; found: 461.1385.

Mechanistic Experiments

Experiment A

In a flame-dried and argon-flushed Schlenk flask, equipped with a Teflon-coated magnetic stirring bar, $Pd(dba)_2$ (7.5 mg, 13 µmol) and BIPHEPHOS (9.6 mg, 12 µmol) were suspended in 2.0 mL anhydrous CH_3CN and stirred in a pre-heated oil bath at 60 °C for 30 min to obtain a bright yellow solution. Then **2f** (138.2 mg, 0.72 mmol) and 1-octanethiol (86.6 mg, 0.59 mmol) were added and the resulting mixture was stirred at 60 °C for 2 h.

GC-MS chromatogram:

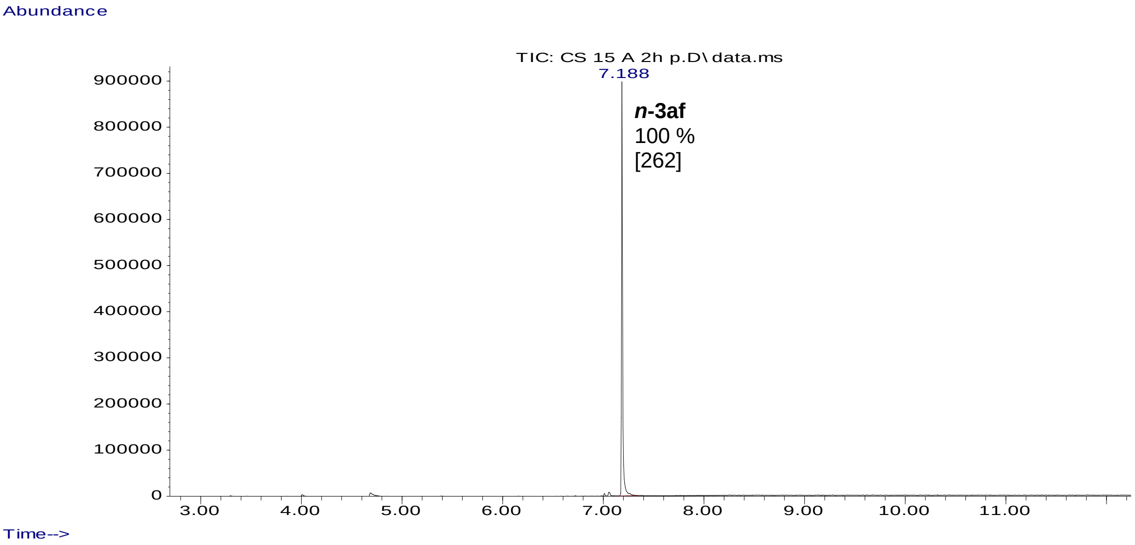

In a flame-dried and argon-flushed Schlenk flask, equipped with a Teflon-coated magnetic stirring bar, $Pd(dba)_2$ (7.4 mg, 13 µmol) and BIPHEPHOS (9.4 mg, 12 µmol) were suspended in 2.0 mL anhydrous CH_3CN and stirred in a pre-heated oil bath at 60 °C for 30 min to obtain a bright yellow solution. Then **2f*** (140.1 mg, 0.73 mmol) and 1-octanethiol (87.5 mg, 0.60 mmol) were added and the resulting mixture was stirred at 60 °C for 2 h.

GC-MS chromatogram:

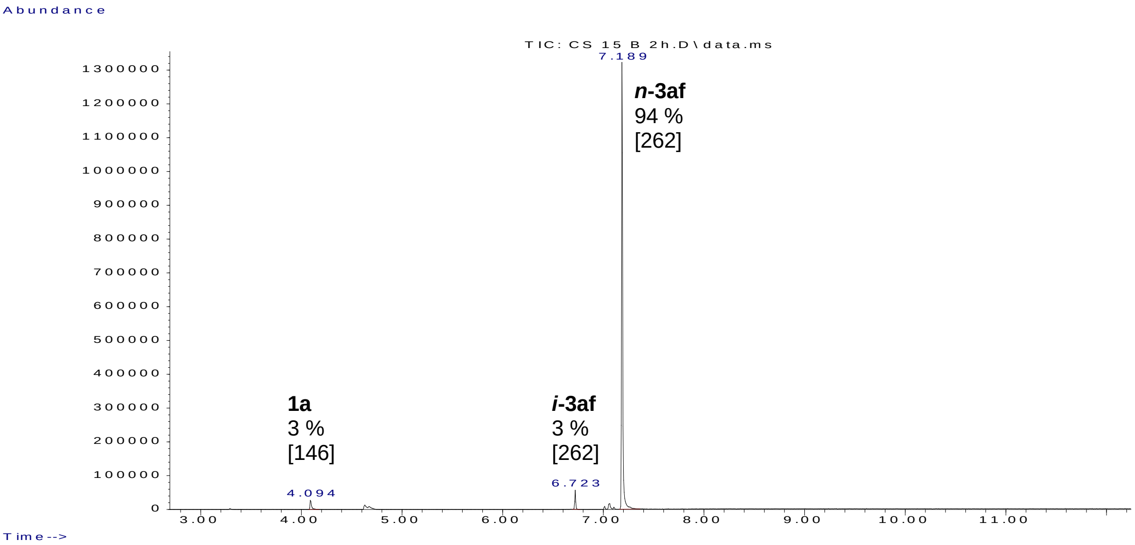

Experiment B

In a flame-dried and argon-flushed Schlenk flask, equipped with a Teflon-coated magnetic stirring bar, $Pd(dba)_2$ (7.0 mg, 12 µmol) and BIPHEPHOS (9.4 mg, 12 µmol) were suspended in 2.0 mL anhydrous CH_3CN and stirred in a pre-heated oil bath at 60 °C for 30 min to obtain a bright yellow solution. Then **2f*** (138.3 mg, 0.72 mmol) was added and the resulting mixture was stirred at 60 °C for 2 h.

<u>^{1}H NMR (300 MHz, $CDCl_3$):</u>

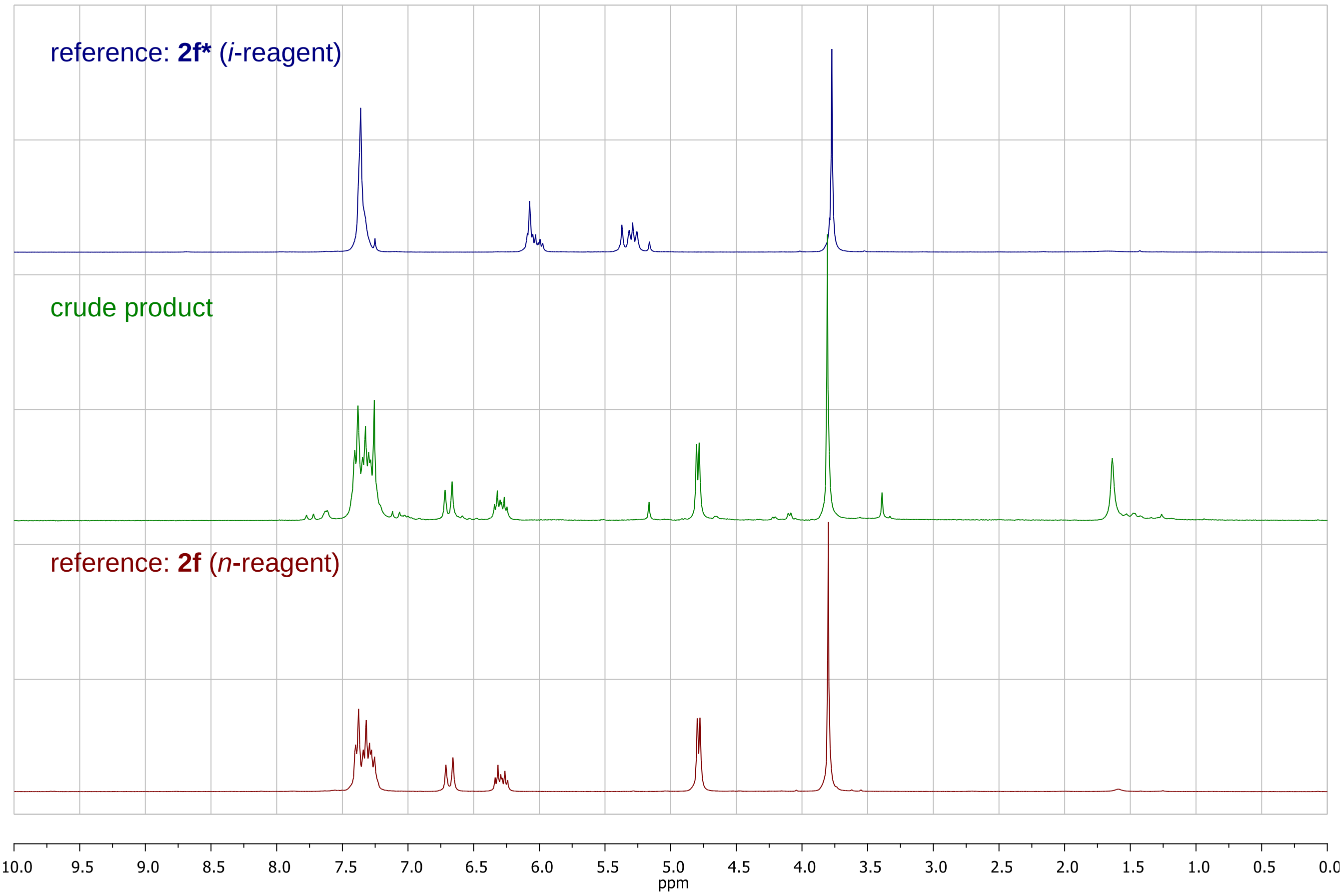

Experiment C

In a flame-dried and argon-flushed Schlenk flask, equipped with a Teflon-coated magnetic stirring bar, $Pd(dba)_2$ (6.6 mg, 11.5 µmol) and BIPHEPHOS (9.0 mg, 11.5 µmol) were suspended in 2.0 mL anhydrous CH_3CN and stirred in a pre-heated oil bath at 60 °C for 30 min to obtain a bright yellow solution. Then **2a** (100 µL, 0.689 mmol) and 1-octanethiol (100 µL, 0.574 mmol) were added and the resulting mixture was stirred at 60 °C.

Experiment D

In a flame-dried and argon-flushed Schlenk flask, equipped with a Teflon-coated magnetic stirring bar, $Pd(dba)_2$ (1.1 mg, 1.9 µmol) and BIPHEPHOS (1.5 mg, 1.9 µmol) were suspended in 0.3 mL anhydrous CH_3CN and stirred in a pre-heated oil bath at 60 °C for 30 min to obtain a bright yellow solution. Then ***i*-3ba** (17.8 mg, 99.8 µmol) was added and the resulting mixture was stirred at 60 °C for 24 h.

GC-MS chromatogram:

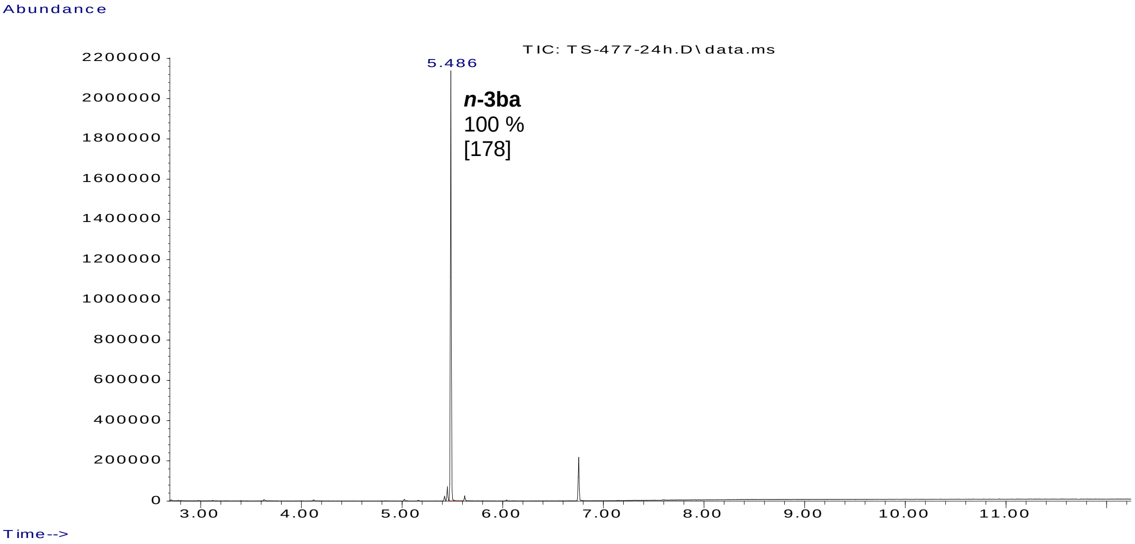

In a flame-dried and argon-flushed Schlenk flask, equipped with a Teflon-coated magnetic stirring bar, NaH (4.0 mg, 60 % dispersion in mineral oil, 100 µmol) and 4-methylbenzenethiol (12.8 mg, 103 µmol) were suspended in 0.7 mL anhydrous CH_3CN and stirred for 30 min. Then ***i*-3ba** (18.7 mg, 105 µmol) was added and the resulting mixture was stirred at 60 °C for 24 h.

GC-MS chromatogram:

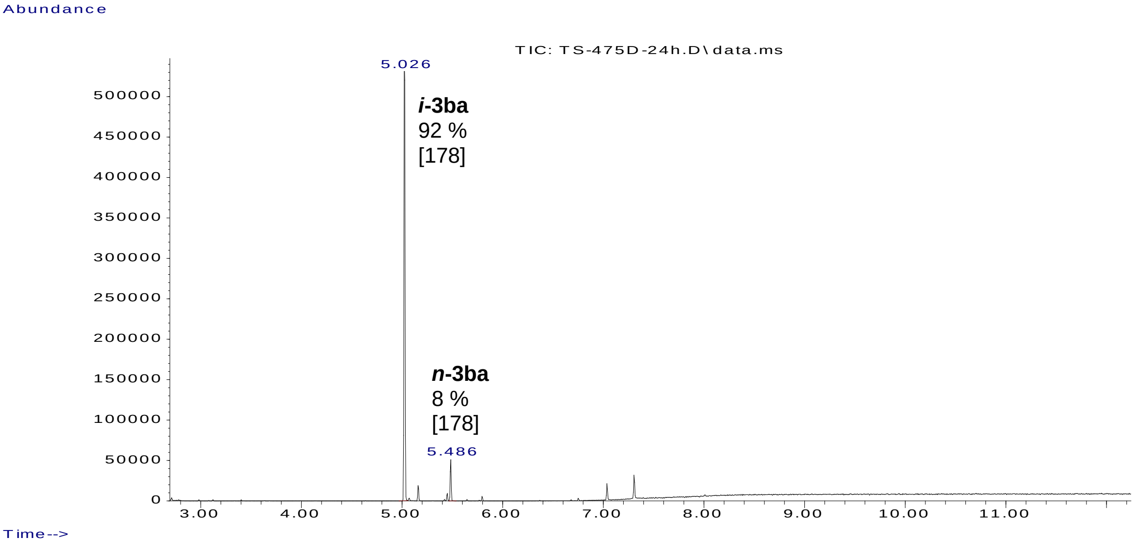

(The starting material ***i*-3ba** exhibited an inital ratio of *i*/n = 93/7.)

Experiment E

In a flame-dried and argon-flushed Schlenk flask, equipped with a Teflon-coated magnetic stirring bar, $Pd(dba)_2$ (2.3 mg, 4.0 µmol) and BIPHEPHOS (3.1 mg, 3.9 µmol) were suspended in 0.7 mL anhydrous CH_3CN and stirred in a pre-heated oil bath at 60 °C for 30 min to obtain a bright yellow solution. Then ***n*-3ba** (18.7 mg, 105 µmol) and **3ci** (17.2 mg, 105 µmol) were added and the resulting mixture was stirred at 60 °C for 24 h.

GC-MS chromatogram:

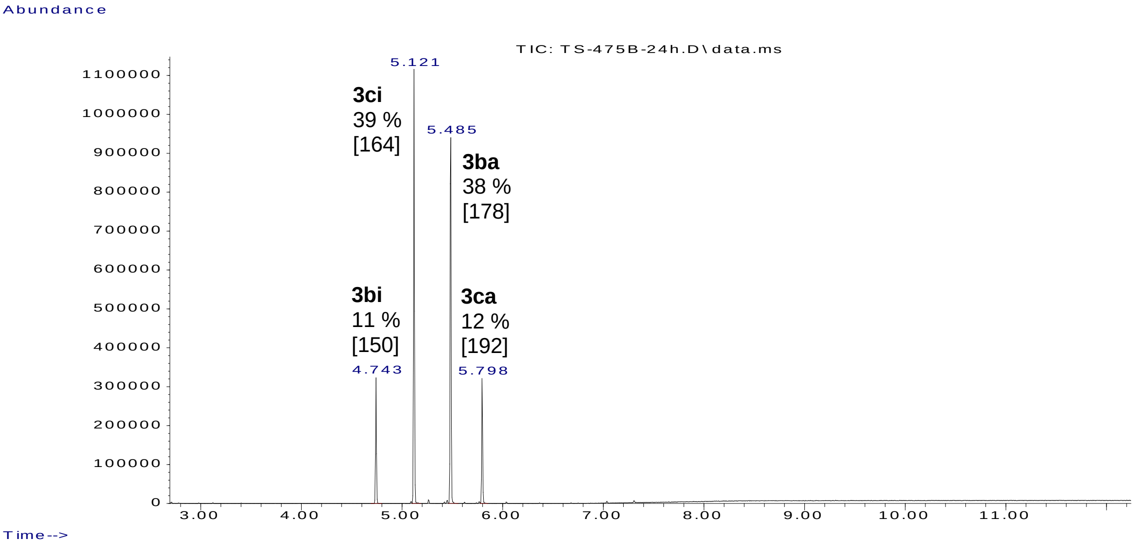

Experiment F

In a flame-dried and argon-flushed Schlenk flask, equipped with a Teflon-coated magnetic stirring bar, $Pd(dba)_2$ (2.3 mg, 4.0 µmol) and BIPHEPHOS (3.0 mg, 3.8 µmol) were suspended in 0.7 mL anhydrous CH_3CN and stirred in a pre-heated oil bath at 60 °C for 30 min to obtain a bright yellow solution. Then ***i*-3ba** (17.7 mg, 99.3 µmol) and **3ci** (16.4 mg, 99.8 µmol) were added and the resulting mixture was stirred at 60 °C for 24 h.

GC-MS chromatogram:

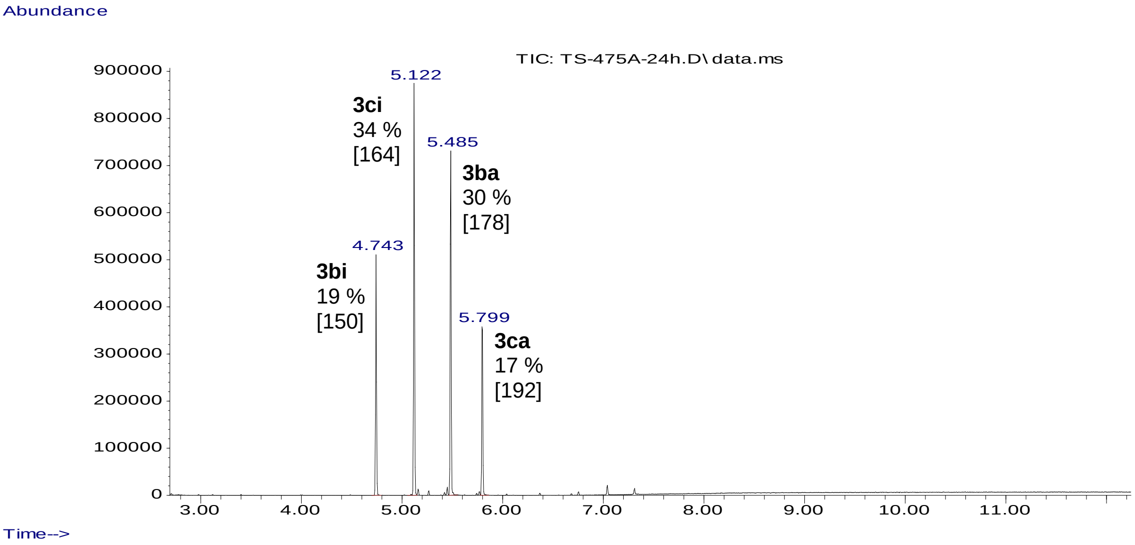

References

[1] O. Busnel, F. Carreaux, B. Carboni, S. Pethe, S. V.-L. Goff, D. Mansuy, J.-L. Boucher, *Bioorg. Med. Chem.* **2005**, *13*, 2373–2379.

[2] L. Zervas, I. Photaki, *J. Am. Chem. Soc.* **1962**, *84*, 3887–3897.

[3] S. Ranganathan, P. Venkateshwarlu, S. M. Babu, N. S. Reddy, S. J. Basha, A.V.S. Sarma, D. Vijay, G. N. Sastry, *Tetrahedron* **2010**, *66*, 3923–3929.

[4] M. Carrasco, R. J. Jones, S. Kamel, H. Rapoport, T. Truong, *Org. Synth.* **1992**, *70*, 29.

[5] L. Wang, D. L. J. Clive, *Org. Lett.* **2011**, *13*, 1734–1737.

[6] C. K. Jin, H. J. Jeong, M. K. Kim, J. Y. Kim, Y.-J. Yoon, S.-G. Lee, *Synlett* **2001**, *2001*, 1956–1958.

[7] J. M. Chalker, C. S. C. Wood, B. G. Davis, *J. Am. Chem. Soc.* **2009**, *131*, 16346–16347.

[8] A. S. Dreiding, J. A. Hartman, *J. Am. Chem. Soc.* **1953**, *75*, 939–943.

[9] N. Marion, R. Gealageas, S. P. Nolan, *Org. Lett.* **2007**, *9*, 2653–2656.

[10] T. Schlatzer, J. Kriegesmann, H. Schröder, M. Trobe, C. Lembacher-Fadum, S. Santner, A. V. Kravchuk, C. F. W. Becker, R. Breinbauer, *J. Am. Chem. Soc.* **2019**, *141*, 14931–14937.

[11] Y. Dai, F. Wu, Z. Zang, H. You, H. Gong, *Chem. Eur. J.* **2012**, *18*, 808–812.

[12] A. Guzman-Martinez, A. H. Hoveyda, *J. Am. Chem. Soc.* **2010**, *132*, 10634–10637.

[13] T. Mitsudo, M. Kadokura, Y. Watanabe, *J. Org. Chem.* **1987**, *52*, 1695–1699.

[14] J. Tsuji, I. Shimizu, I. Minami, Y. Ohashi, *Tetrahedron Lett.* **1982**, *23*, 4809–4812.

[15] D. C. Vrieze, G. S. Hoge, P. Z. Hoerter, J. T. van Haitsma, B. M. Samas, *Org. Lett.* **2009**, *11*, 3140–3142.

[16] P. Tosatti, J. Horn, A. J. Campbell, D. House, A. Nelson, S. P. Marsden, *Adv. Synth. Catal.* **2010**, *352*, 3153–3157.

[17] W.-J. Xiao, G. Vasapollo, H. Alper, *J. Org. Chem.* **2000**, *65*, 4138–4144.

[18] A. B. Zaitsev, H. F. Caldwell, P. S. Pregosin, L. F. Veiros, *Chem. Eur. J.* **2009**, *15*, 6468–6477.

NMR-Spectra

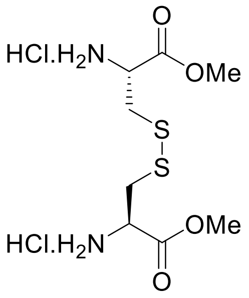

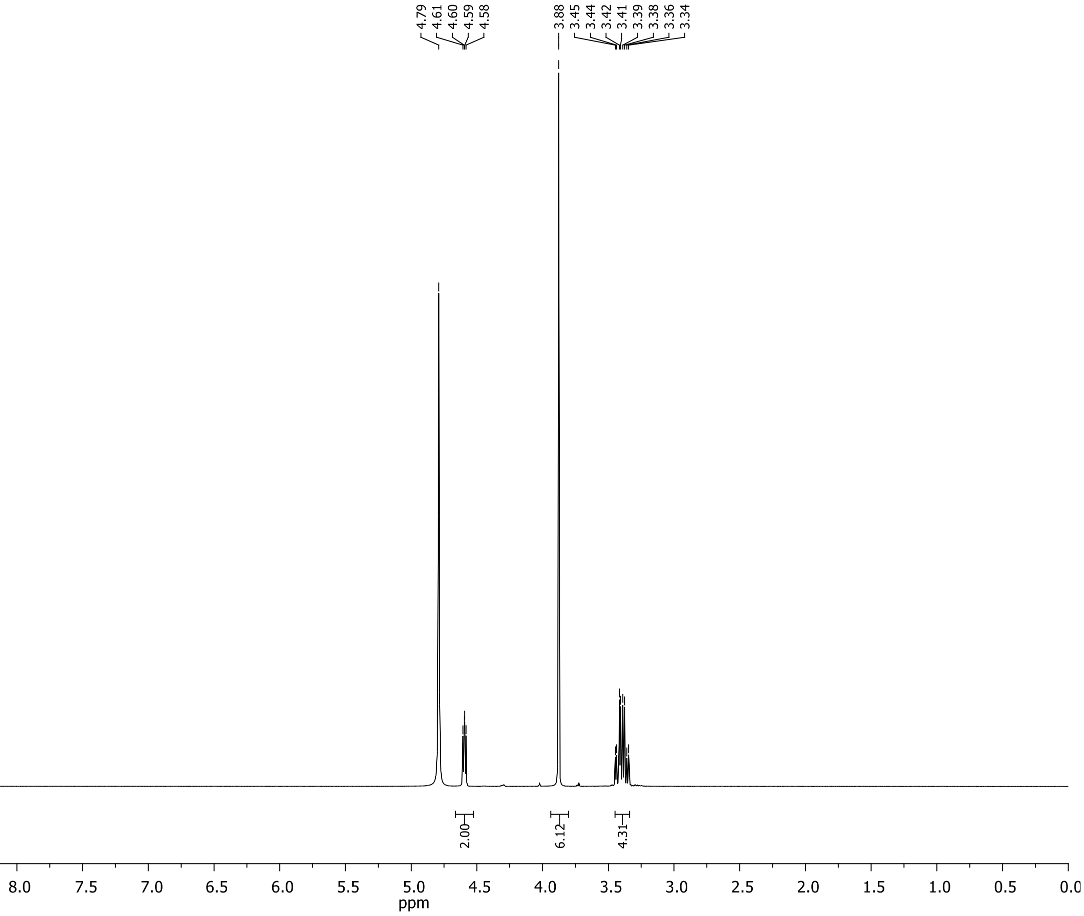

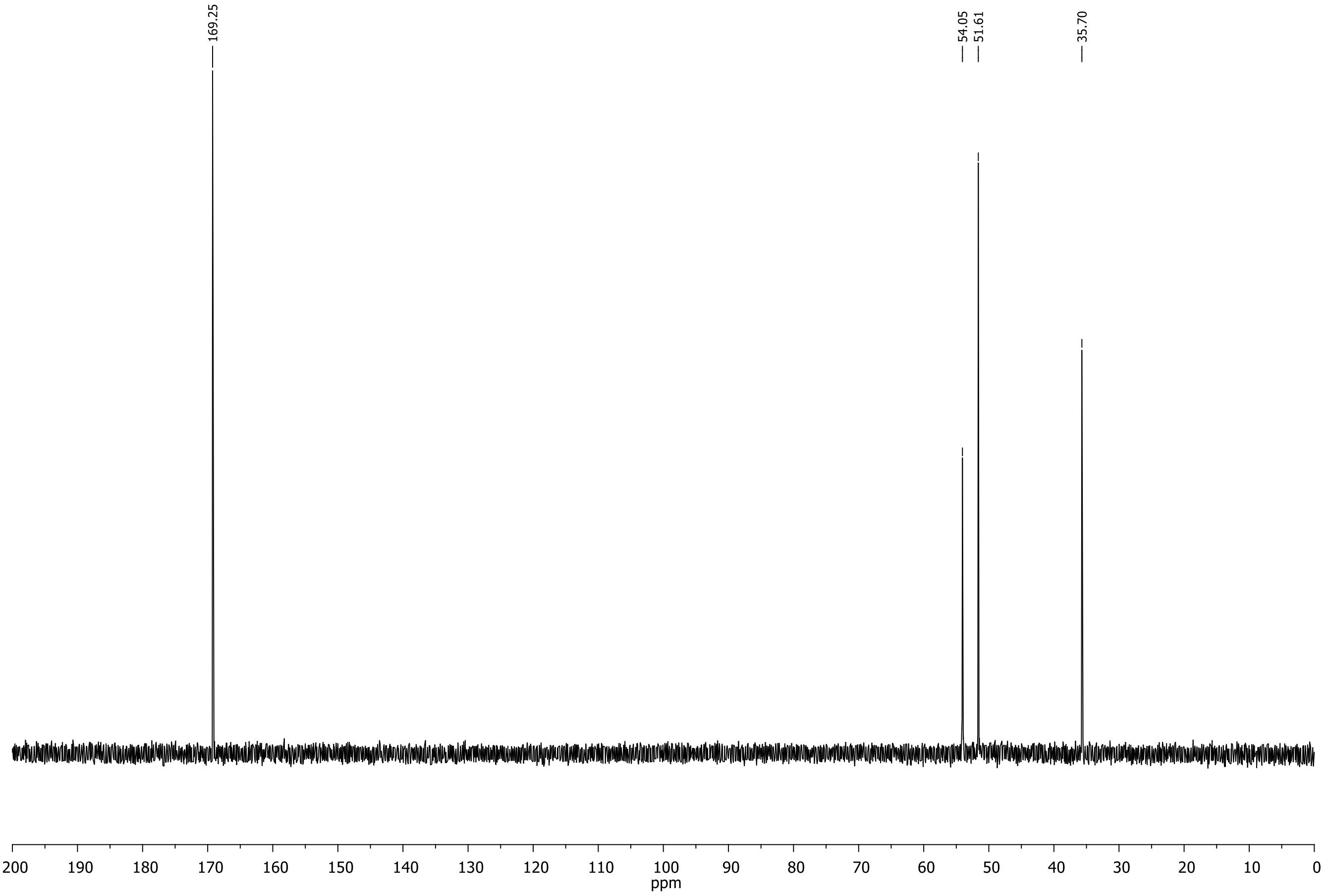

^{1}H-NMR (499.87 MHz, D_2O); ^{13}C-NMR: (125.69 MHz, D_2O)

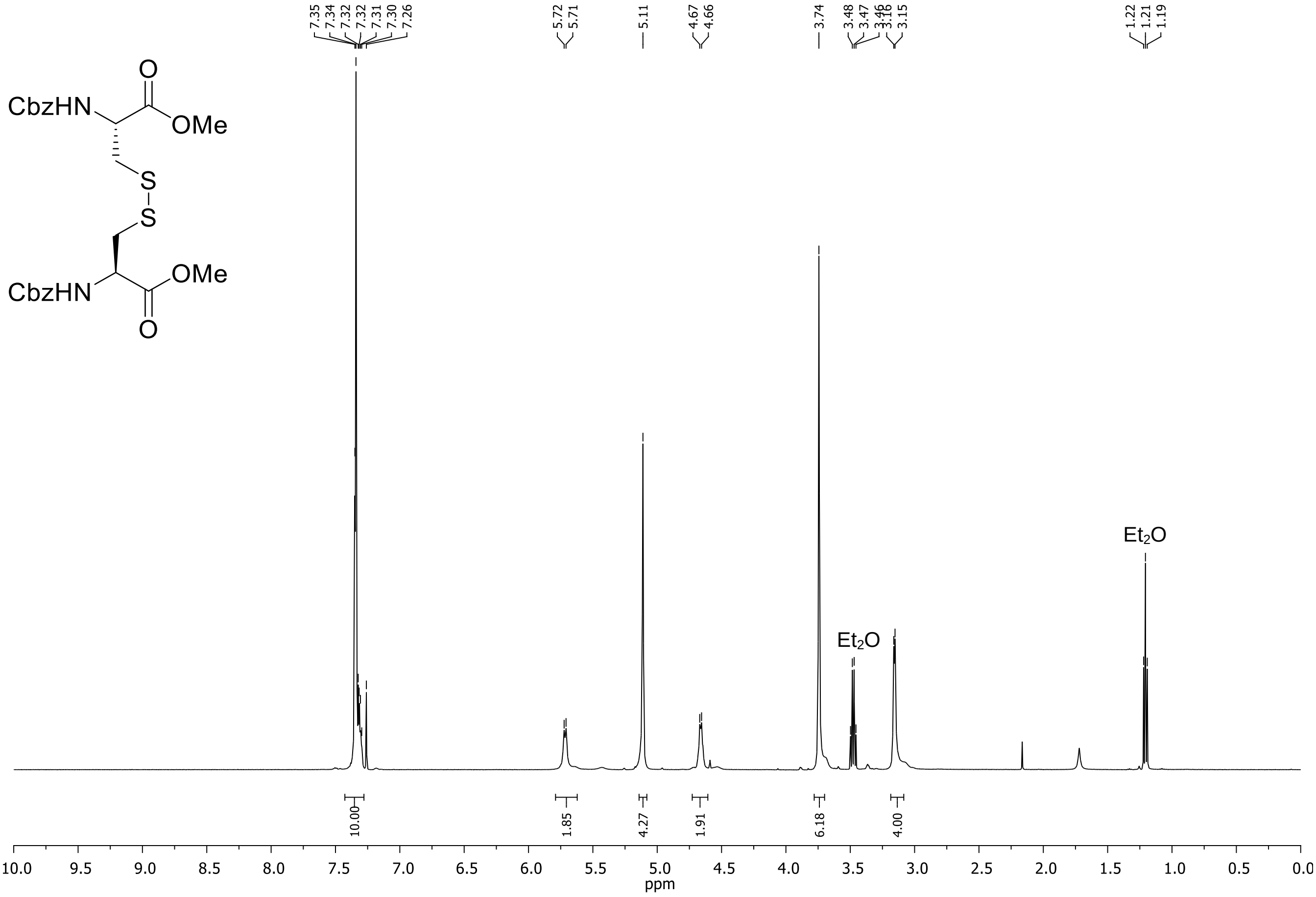

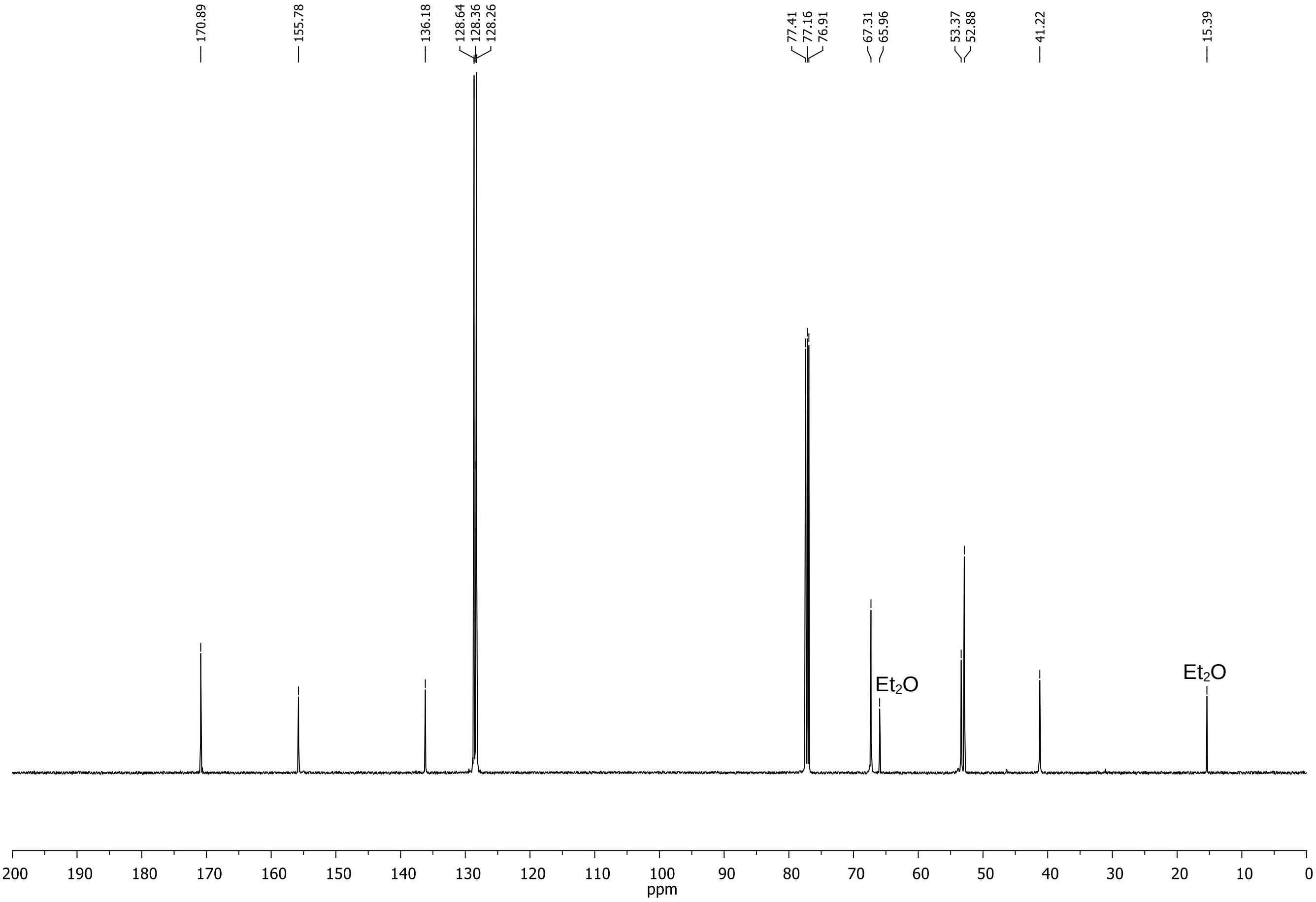

^{1}H-NMR (499.87 MHz, $CDCl_3$); ^{13}C-NMR: (125.69 MHz, $CDCl_3$)

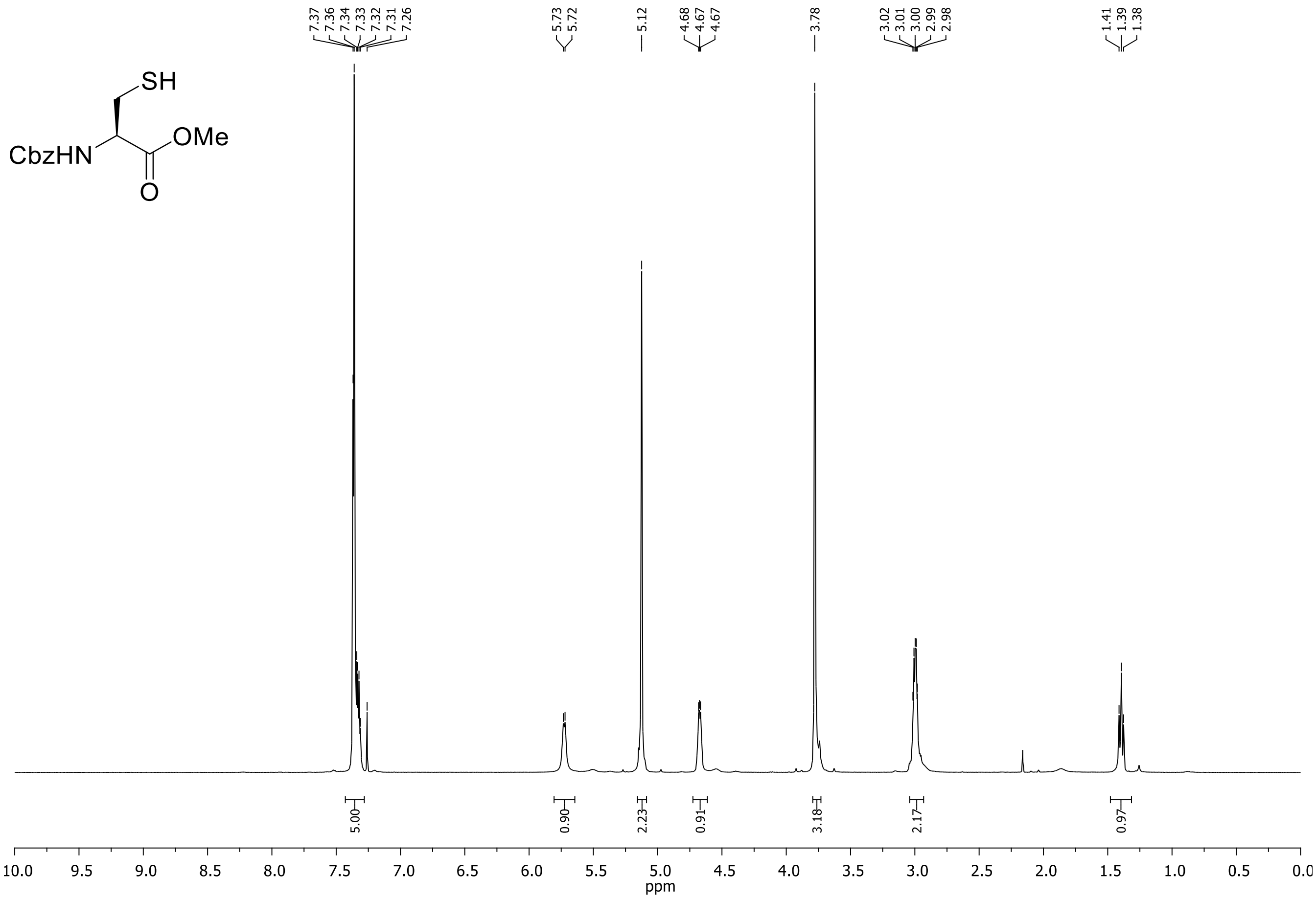

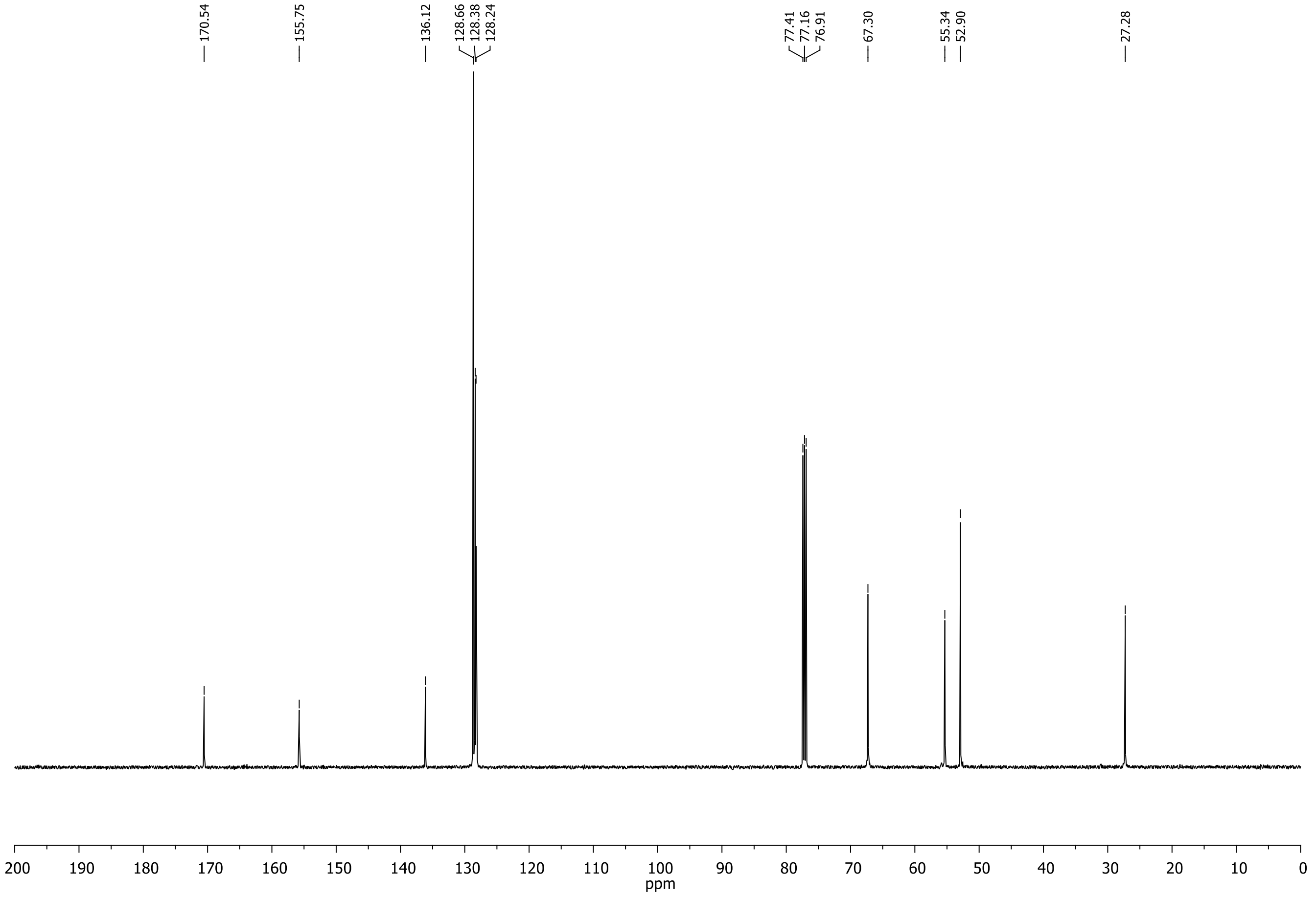

^{1}H-NMR (499.87 MHz, $CDCl_3$); ^{13}C-NMR: (125.69 MHz, $CDCl_3$)

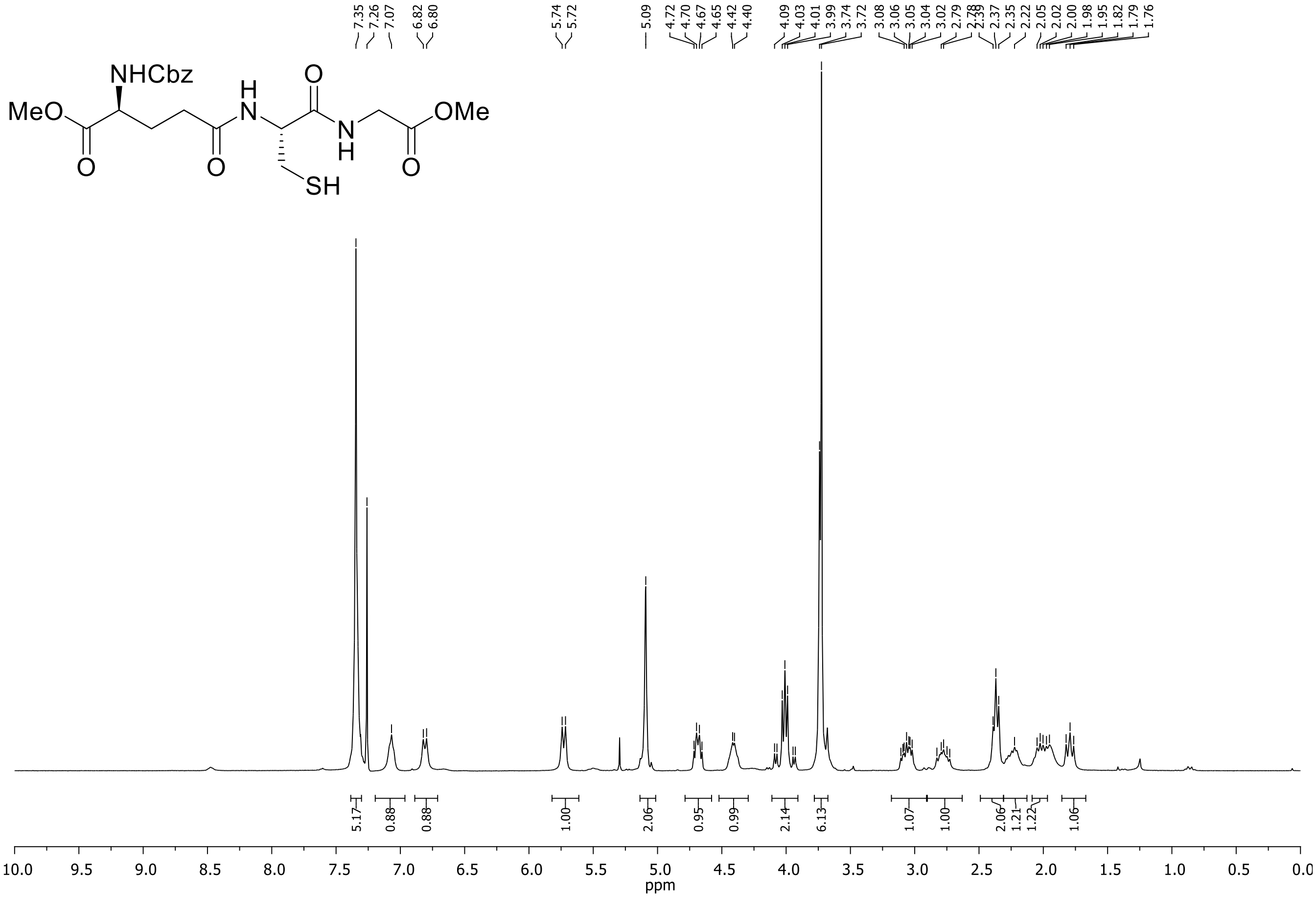

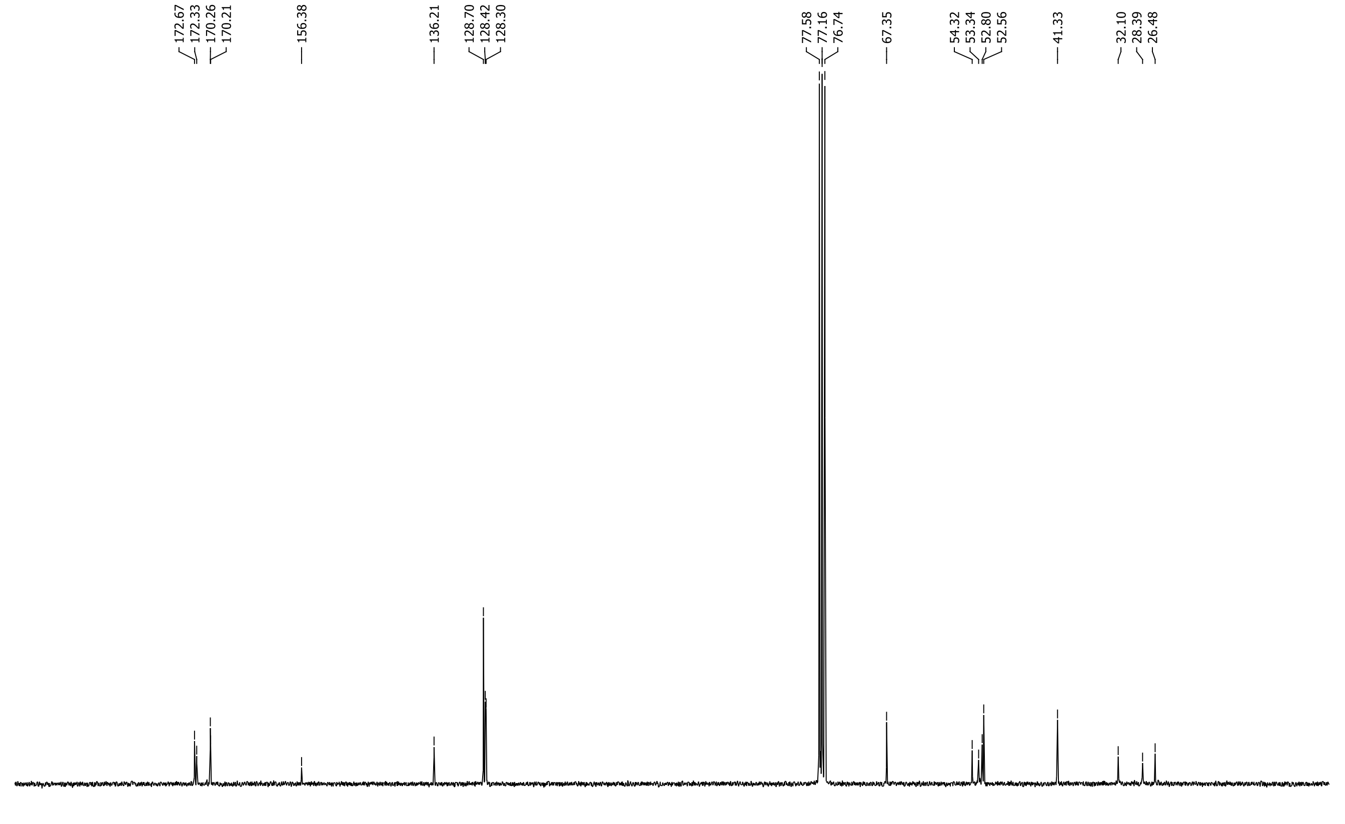

^{1}H-NMR (300.36 MHz, $CDCl_3$); ^{13}C-NMR: (75.53 MHz, $CDCl_3$)

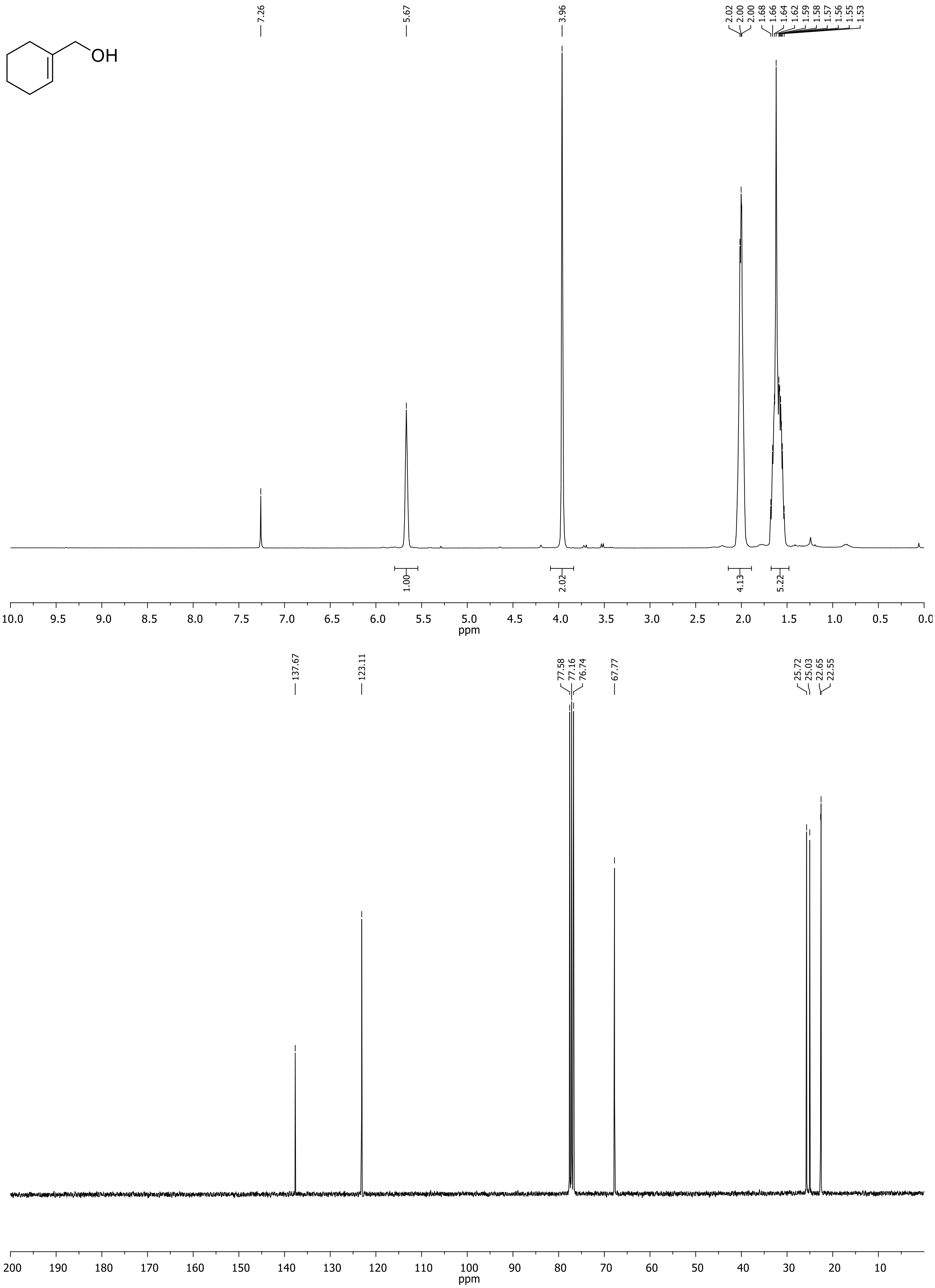

^{1}H-NMR (300.36 MHz, $CDCl_3$); ^{13}C-NMR: (75.53 MHz, $CDCl_3$)

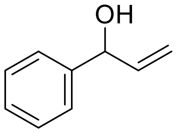

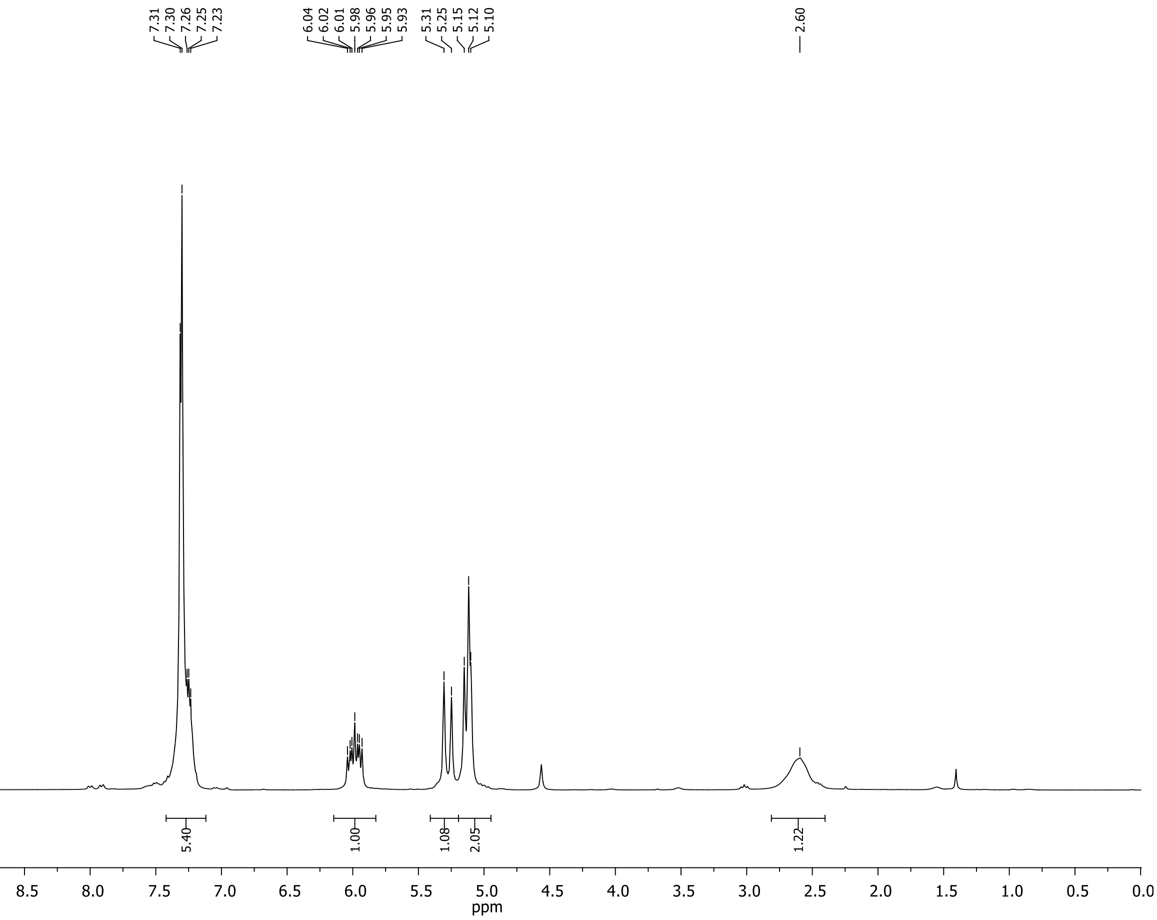

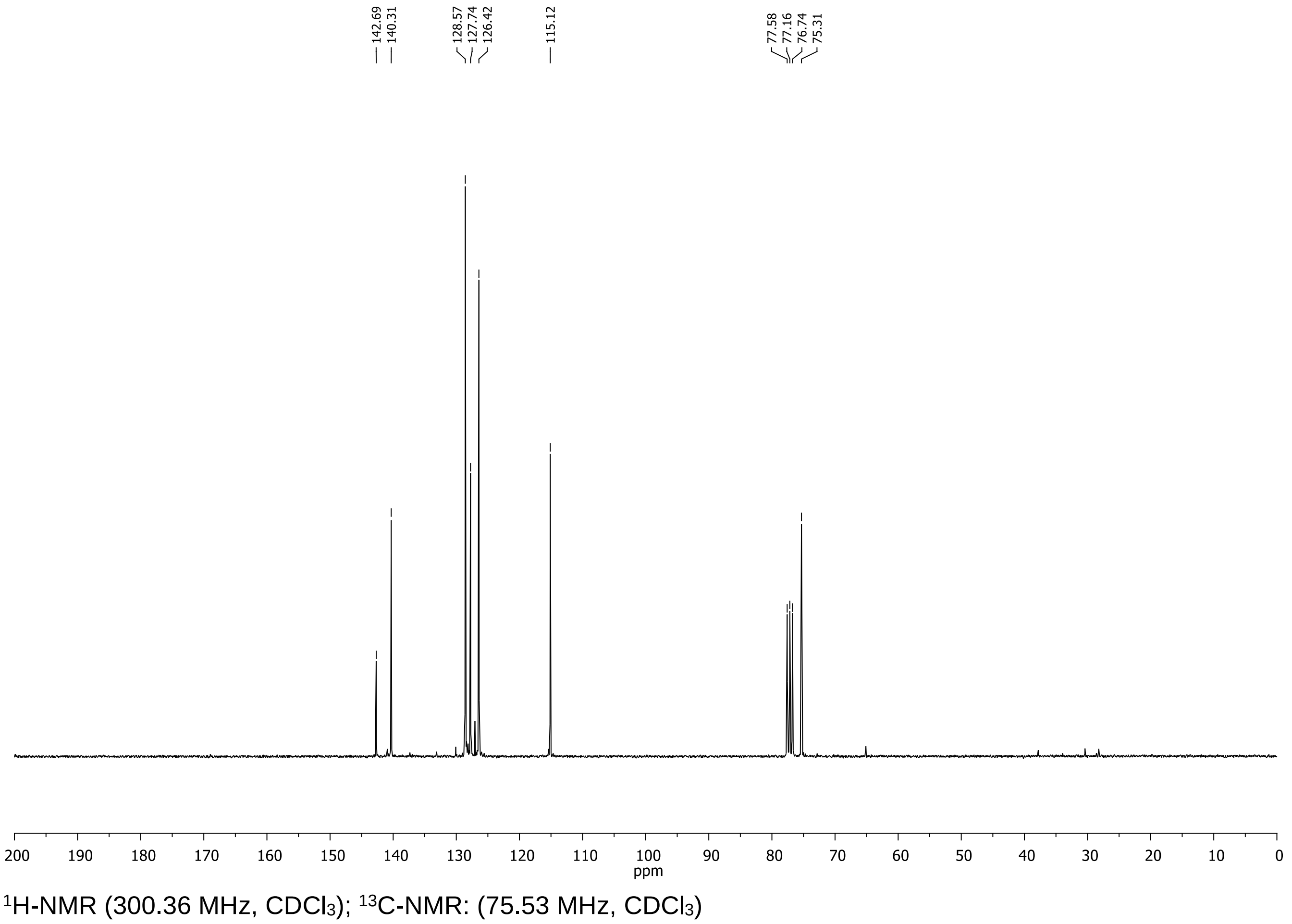

^{1}H-NMR (300.36 MHz, $CDCl_3$); ^{13}C-NMR: (75.53 MHz, $CDCl_3$)

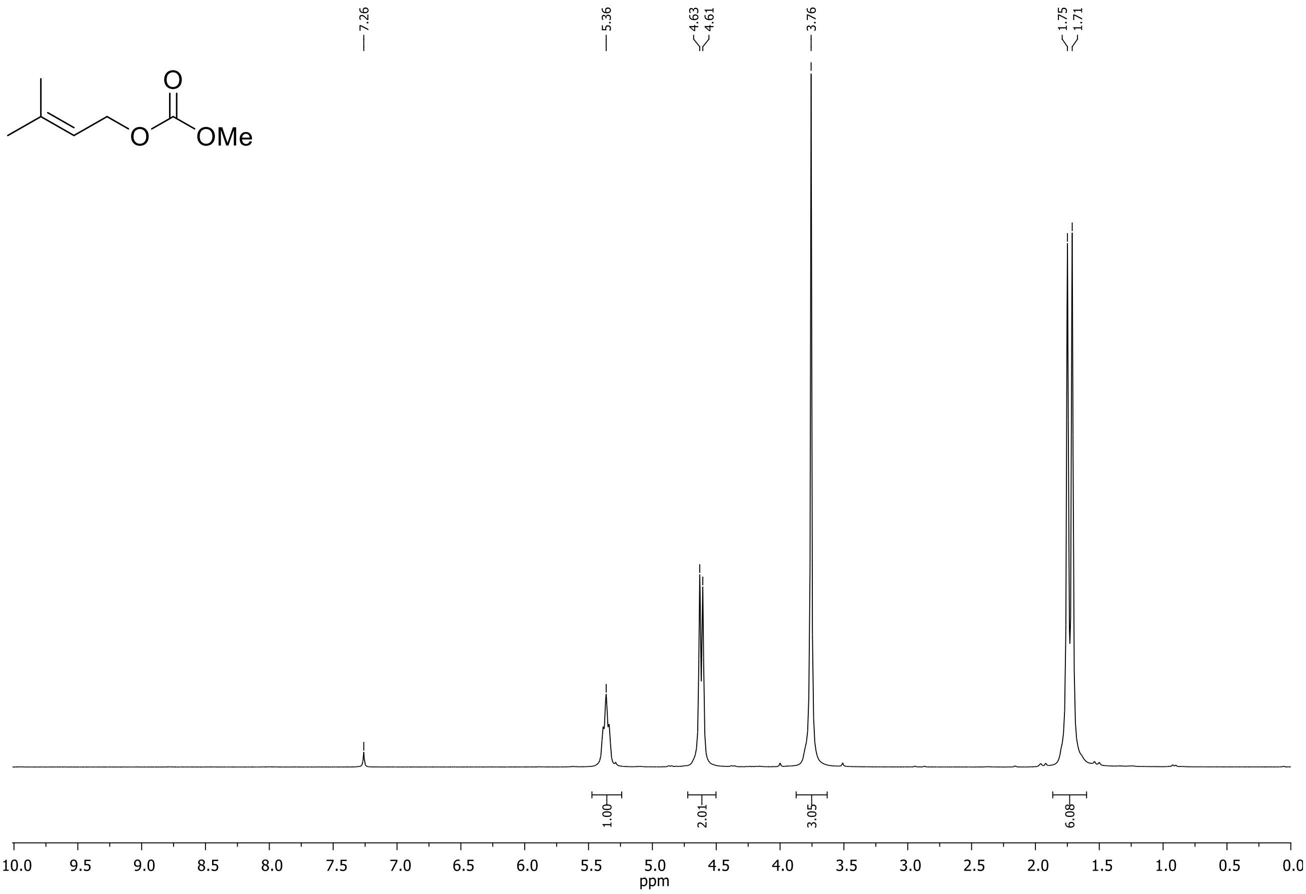

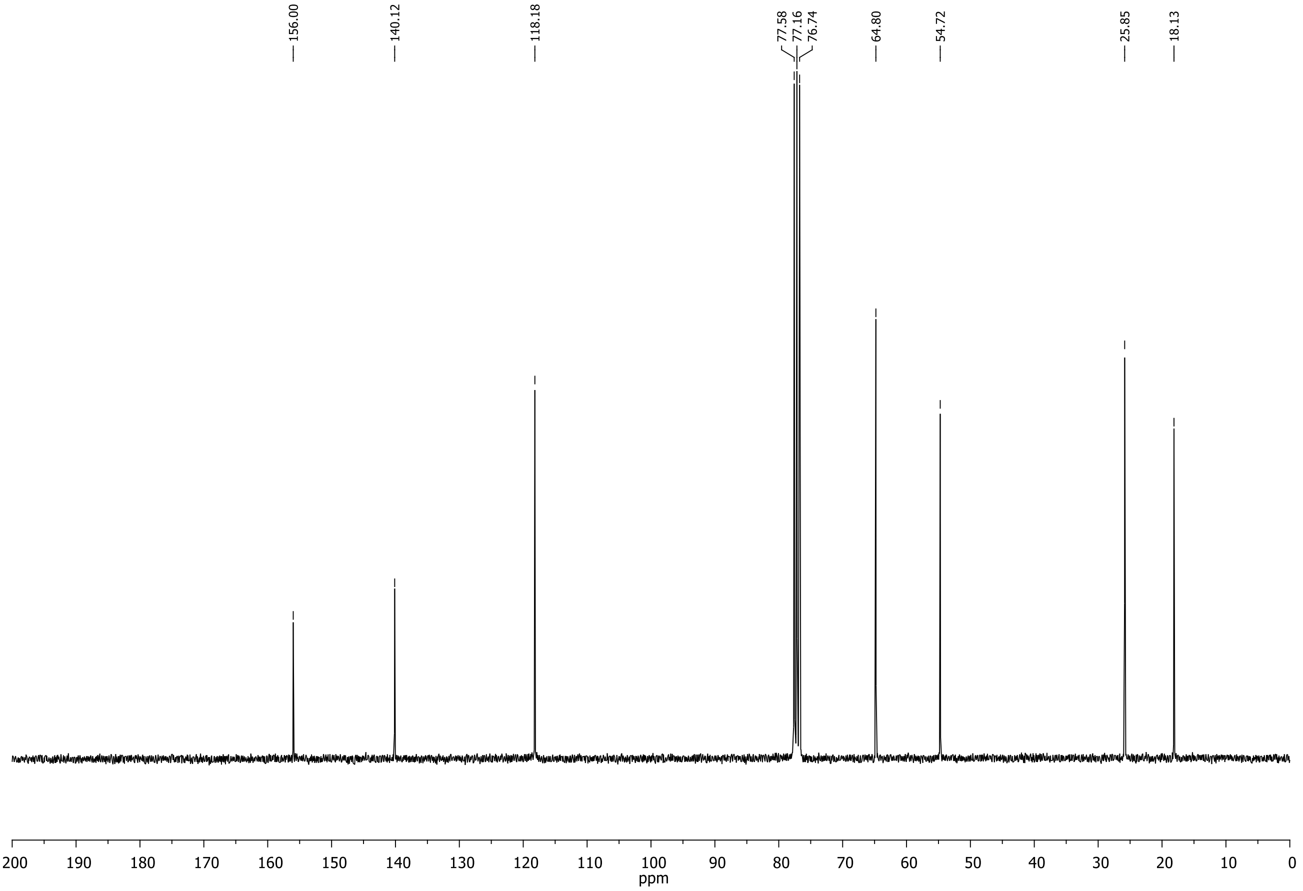

^{1}H-NMR (300.36 MHz, $CDCl_3$); ^{13}C-NMR: (75.53 MHz, $CDCl_3$)

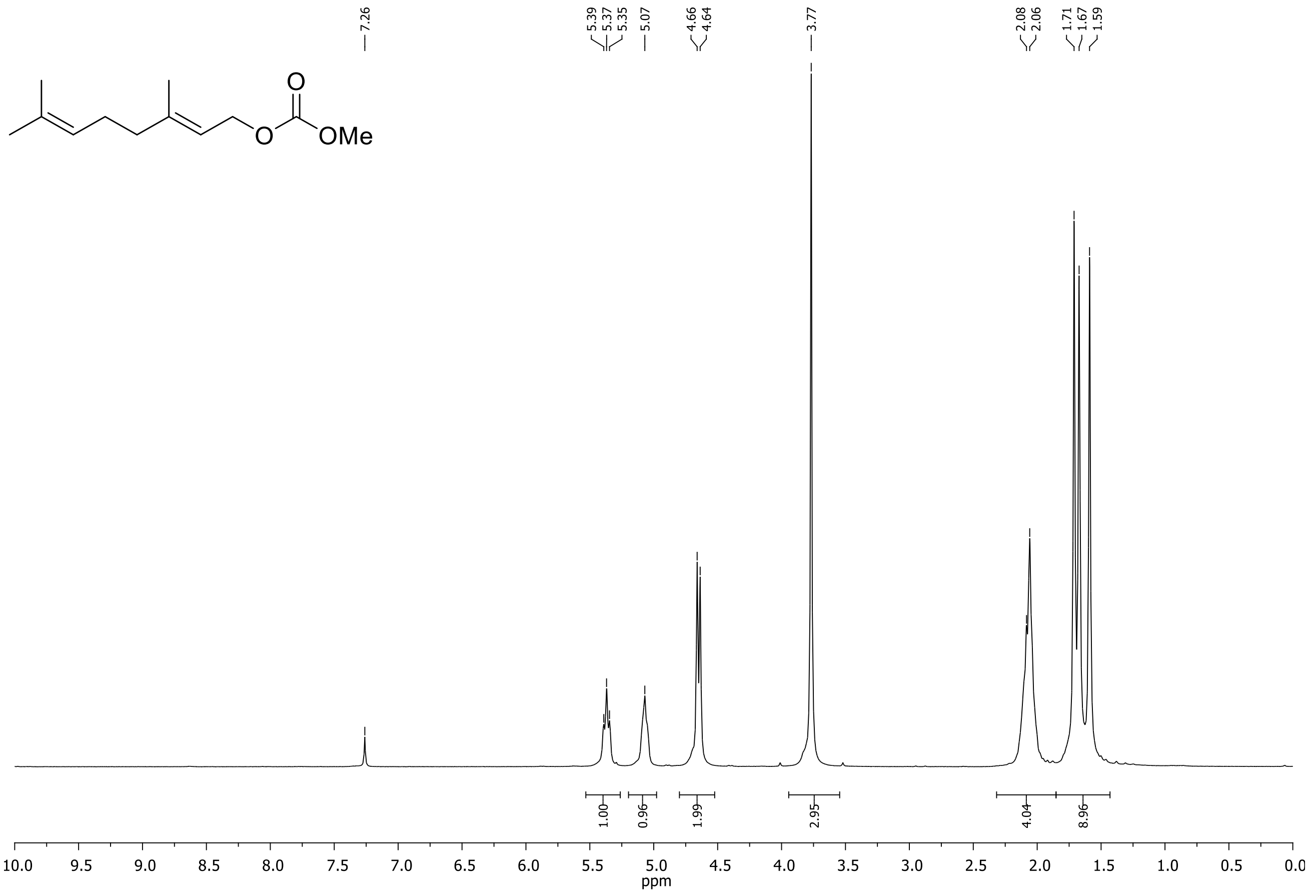

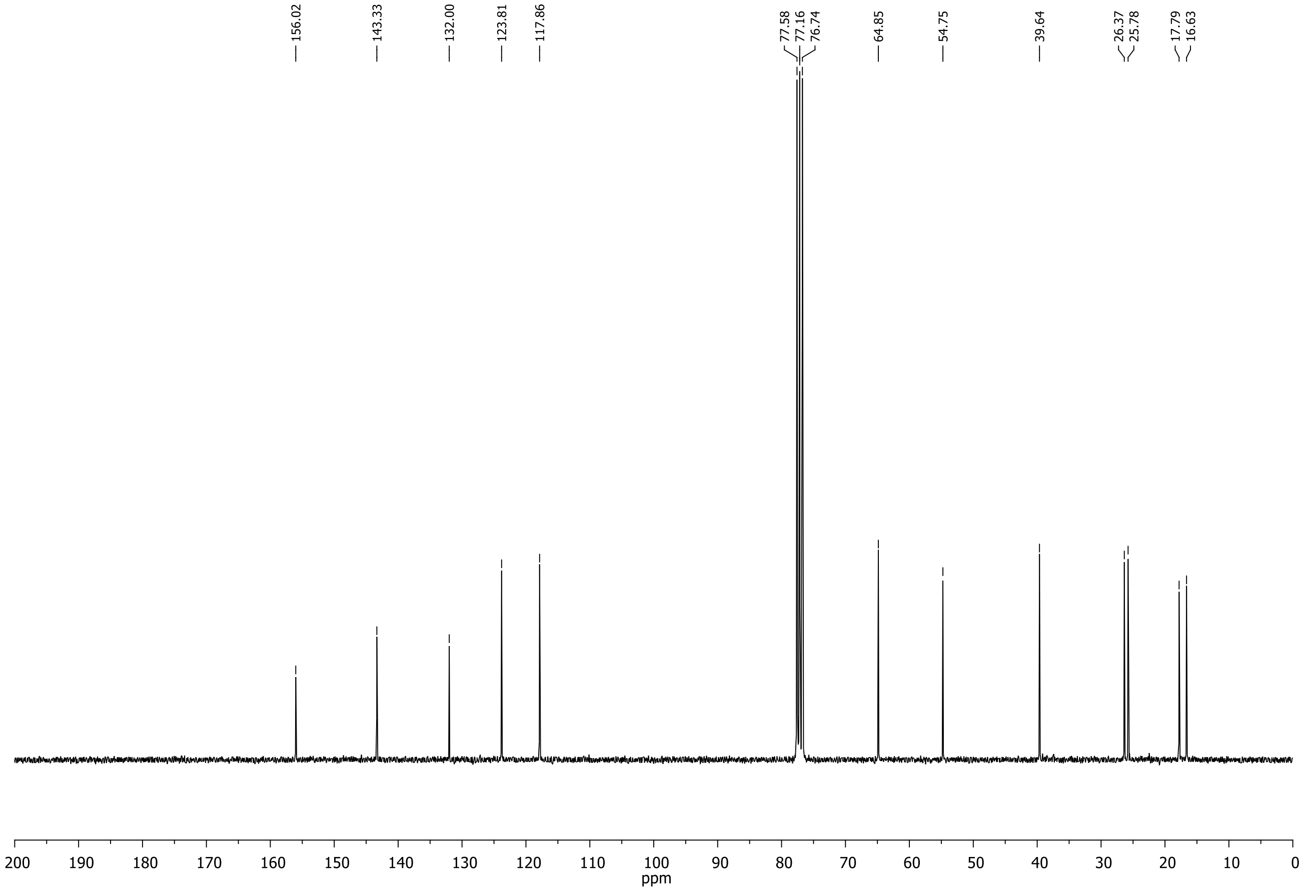

^{1}H-NMR (300.36 MHz, $CDCl_3$); ^{13}C-NMR: (75.53 MHz, $CDCl_3$)

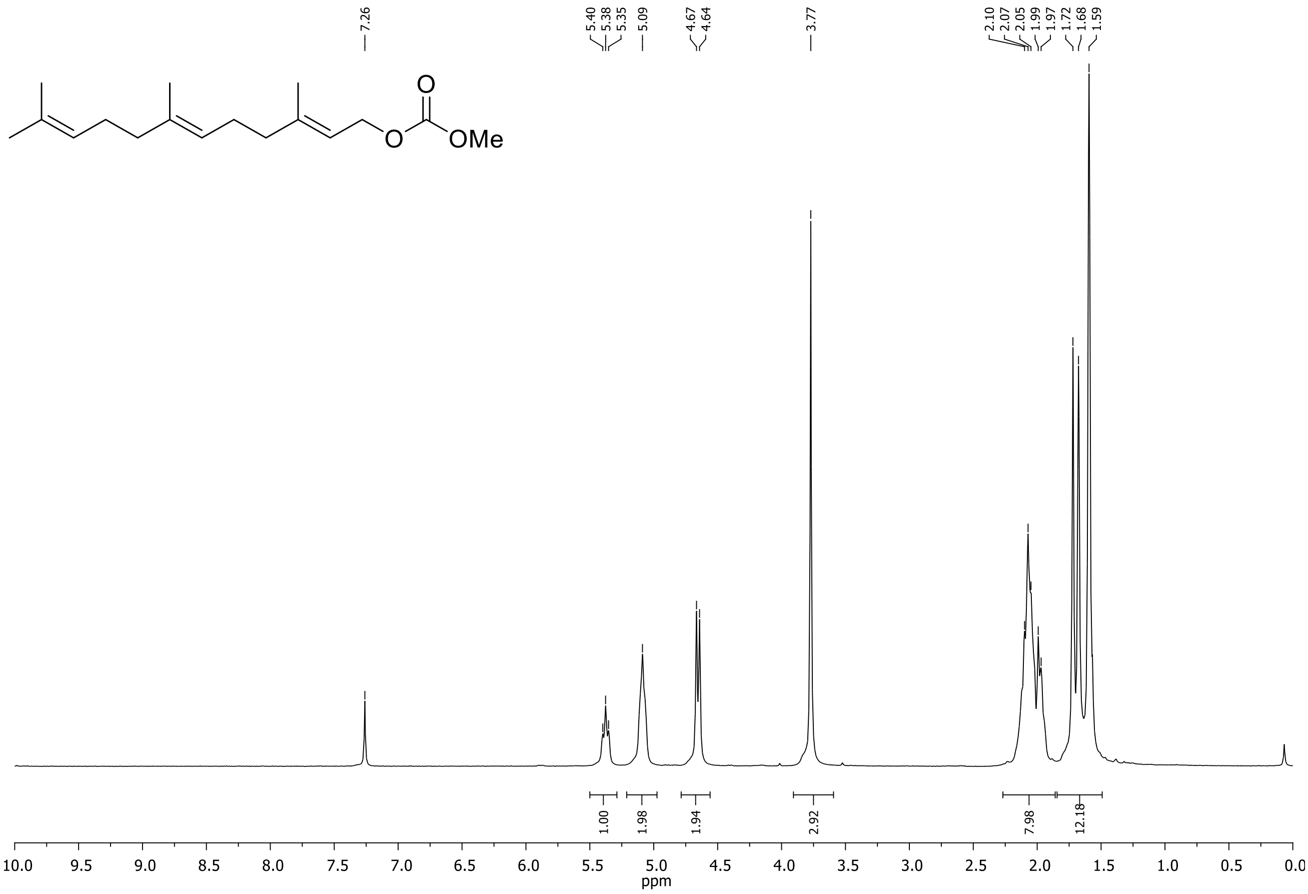

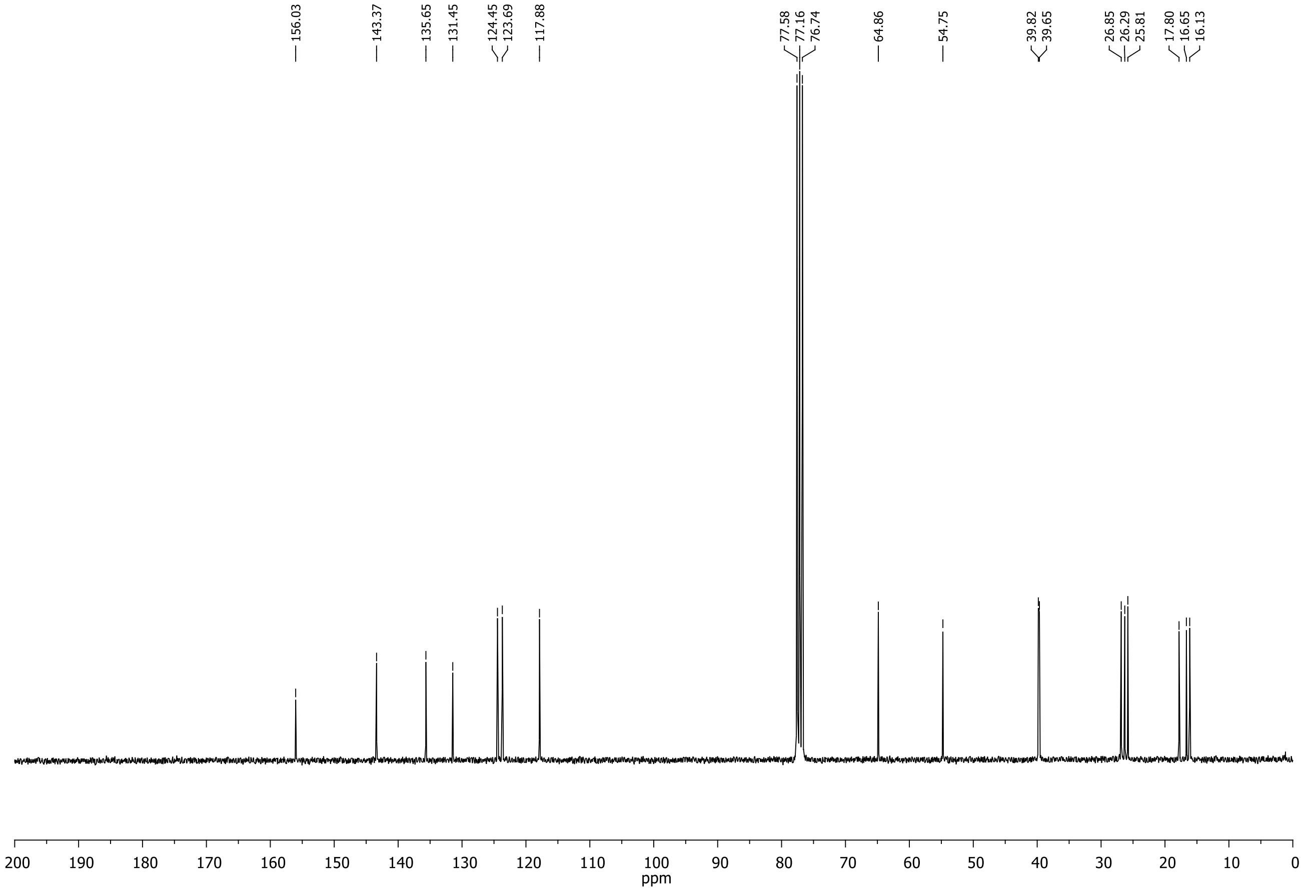

^{1}H-NMR (300.36 MHz, $CDCl_3$); ^{13}C-NMR: (75.53 MHz, $CDCl_3$)

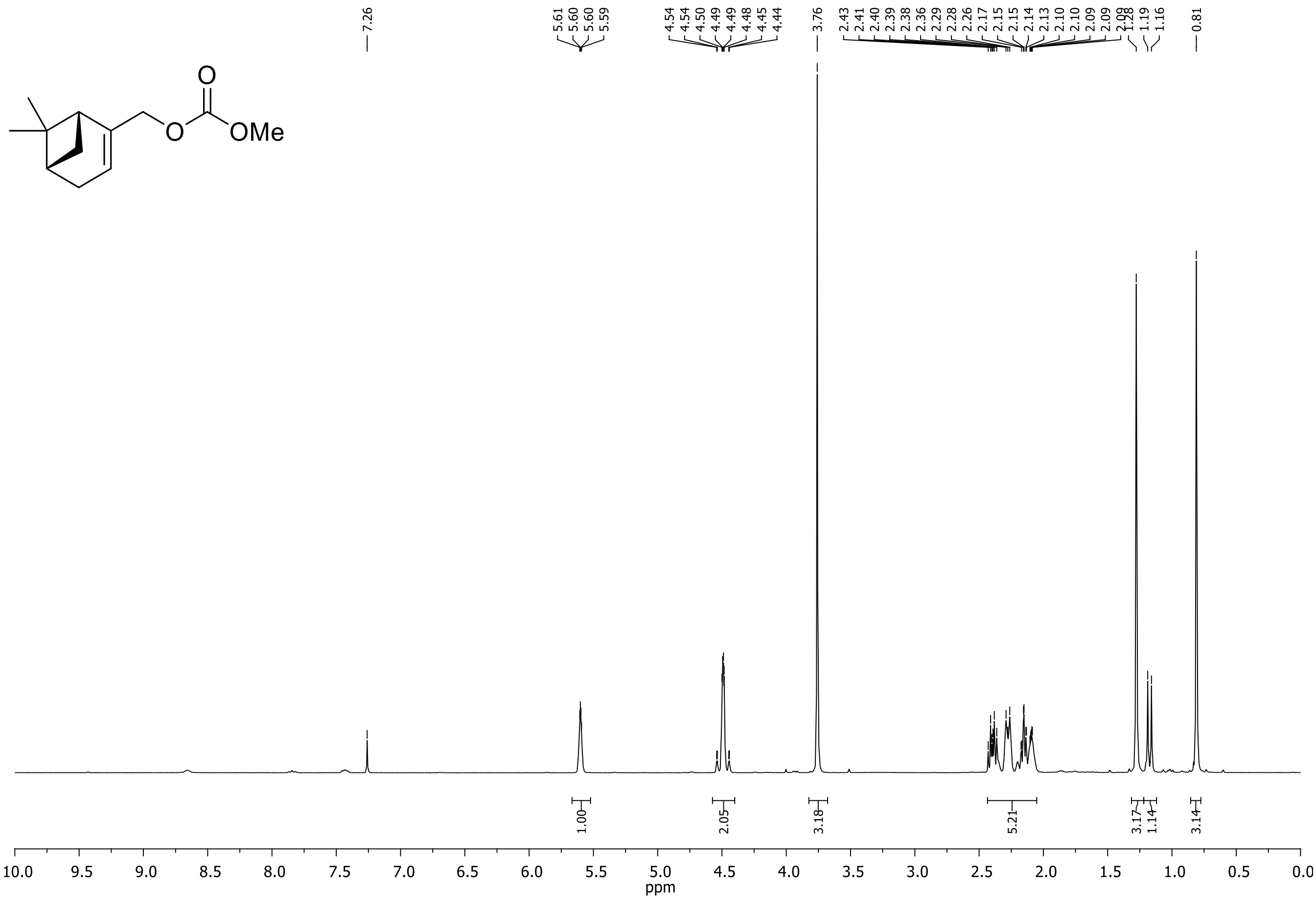

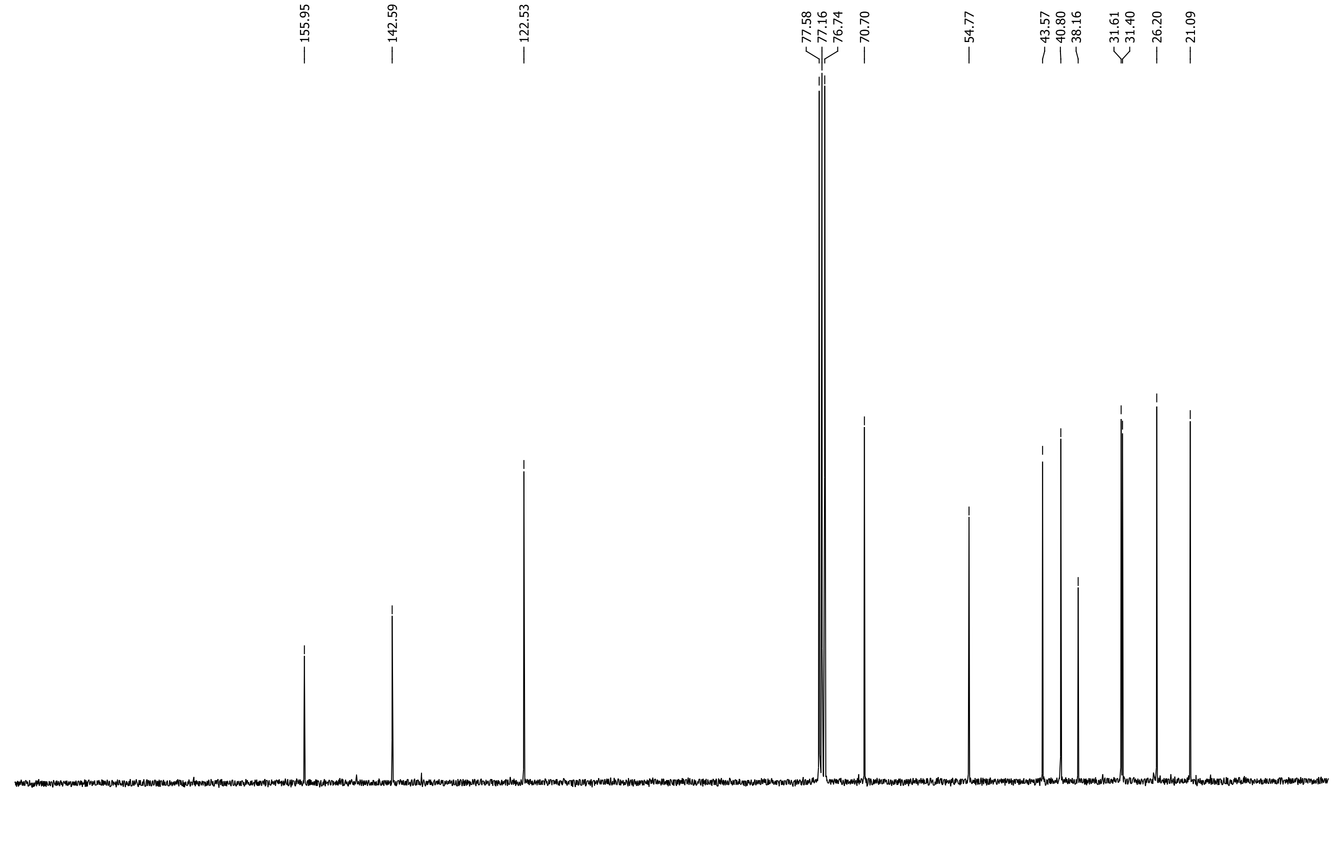

^{1}H-NMR (300.36 MHz, $CDCl_3$); ^{13}C-NMR: (75.53 MHz, $CDCl_3$)

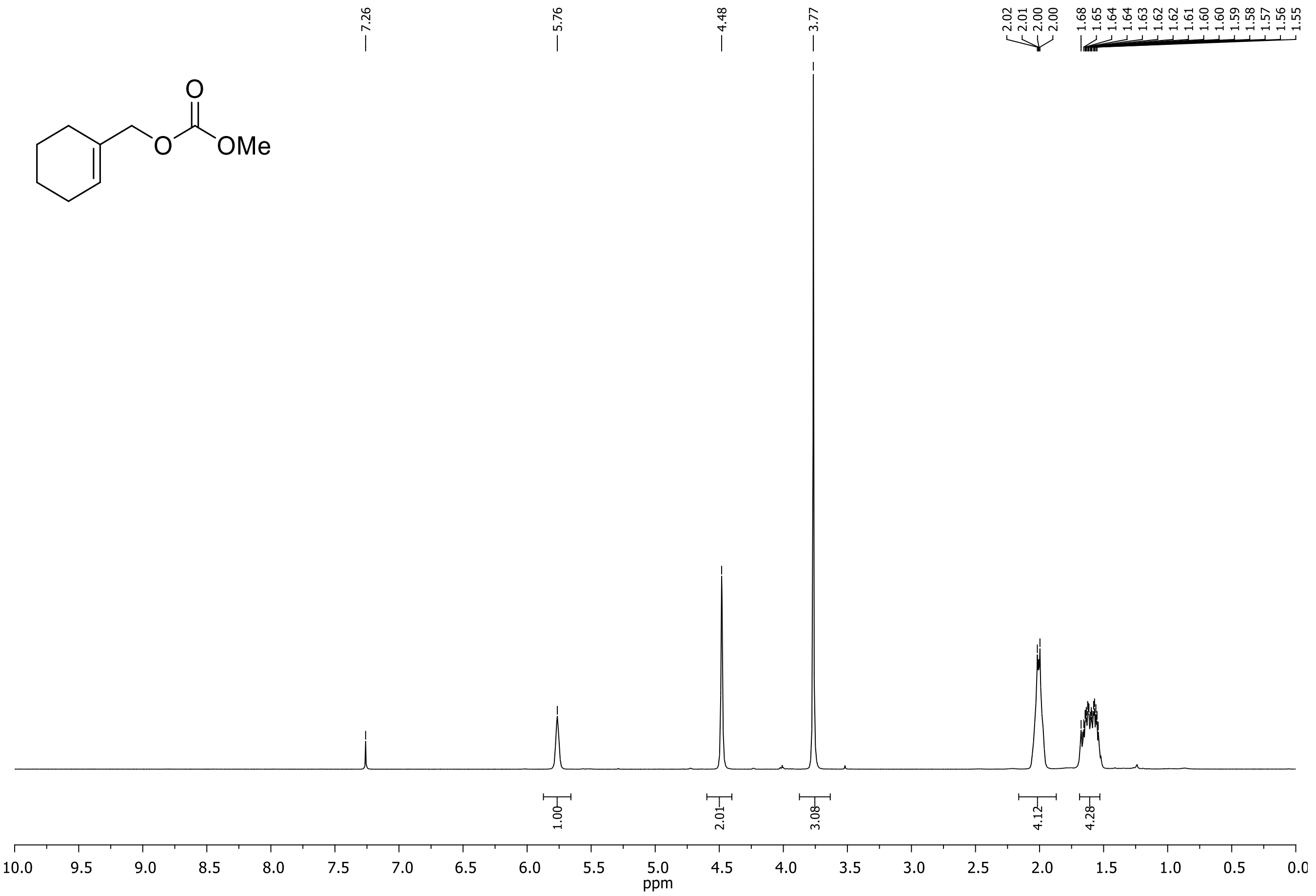

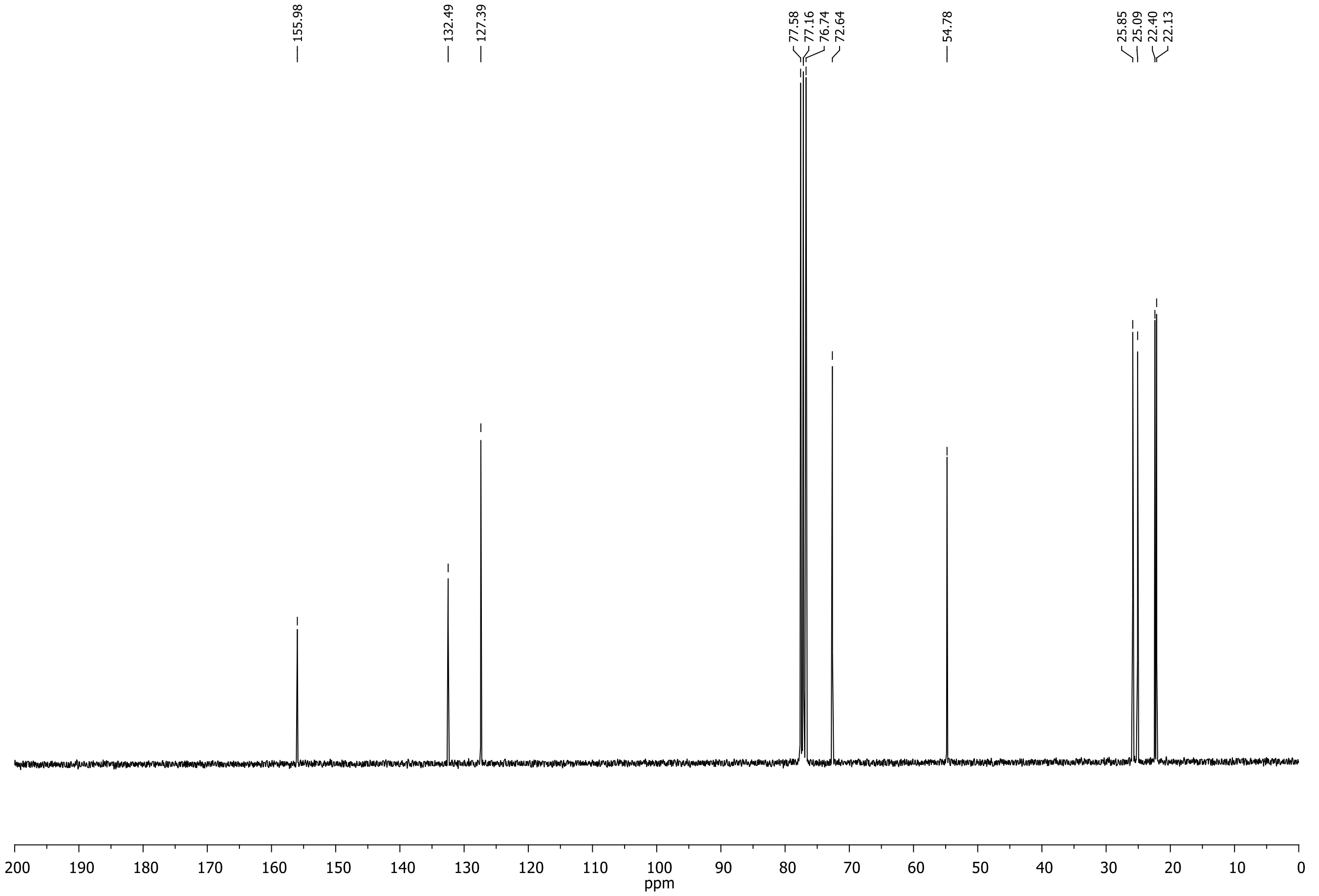

^{1}H-NMR (300.36 MHz, $CDCl_3$); ^{13}C-NMR: (75.53 MHz, $CDCl_3$)

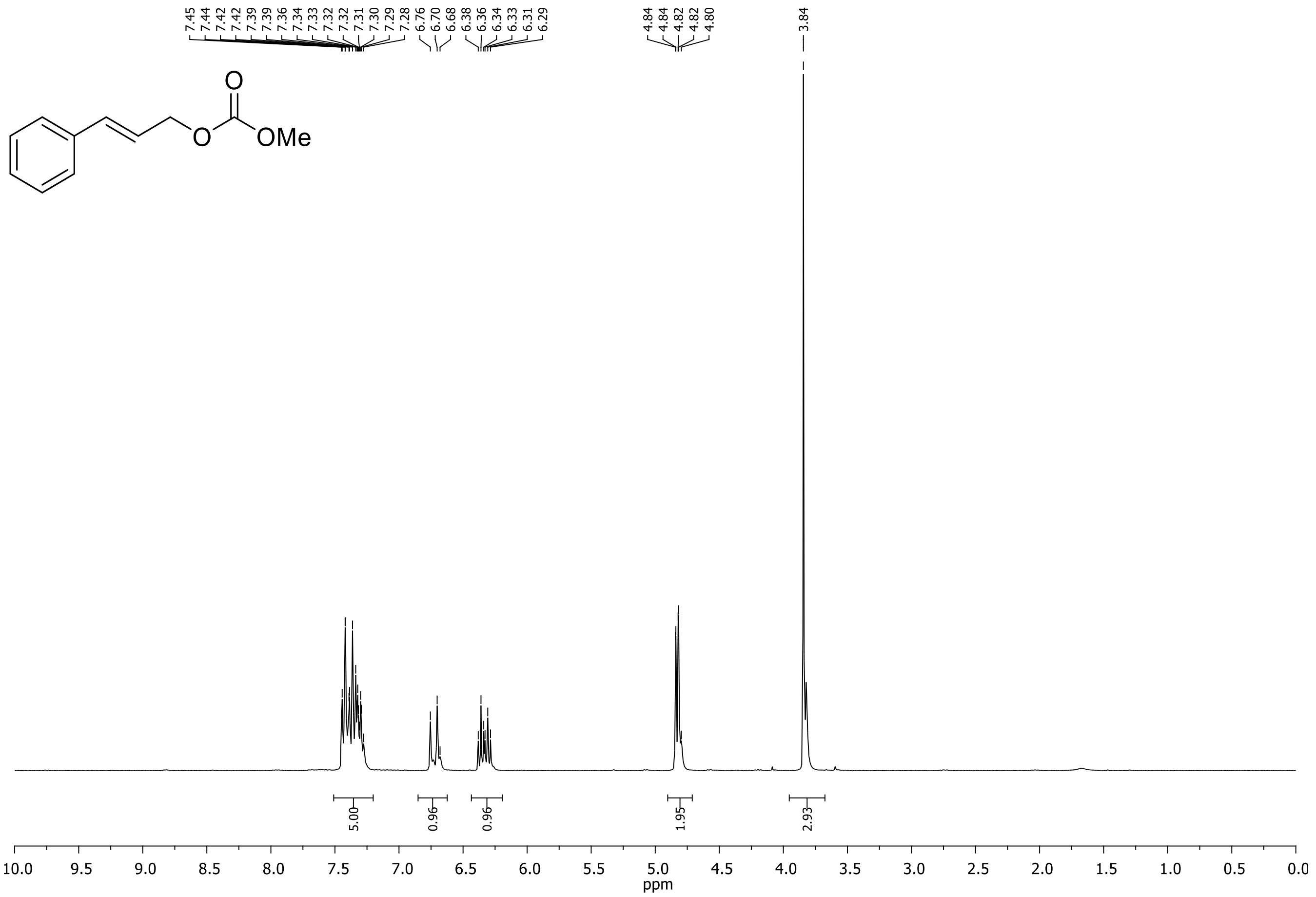

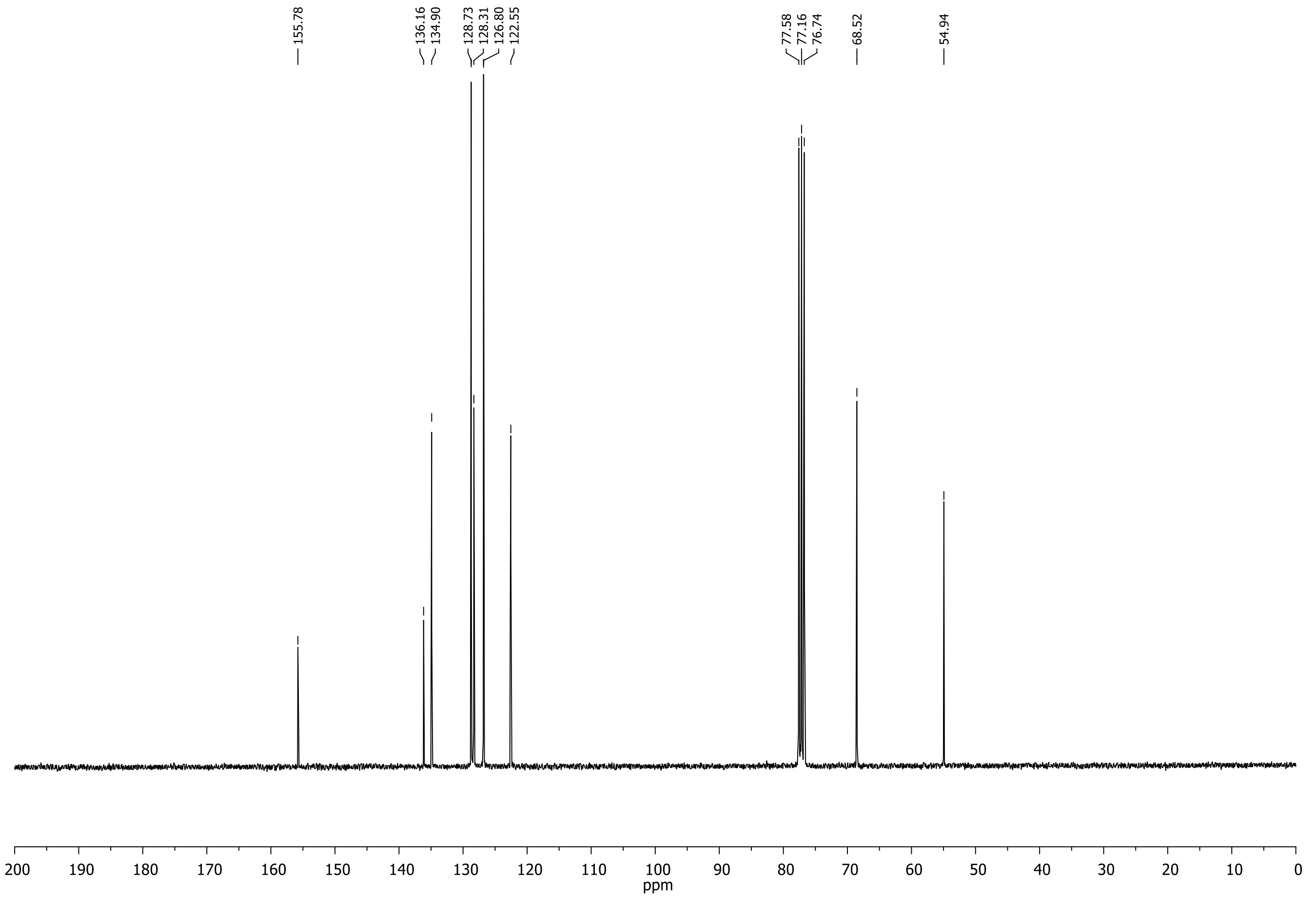

^1H-NMR (300.36 MHz, $CDCl_3$); ^{13}C-NMR: (75.53 MHz, $CDCl_3$)

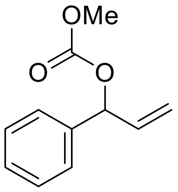

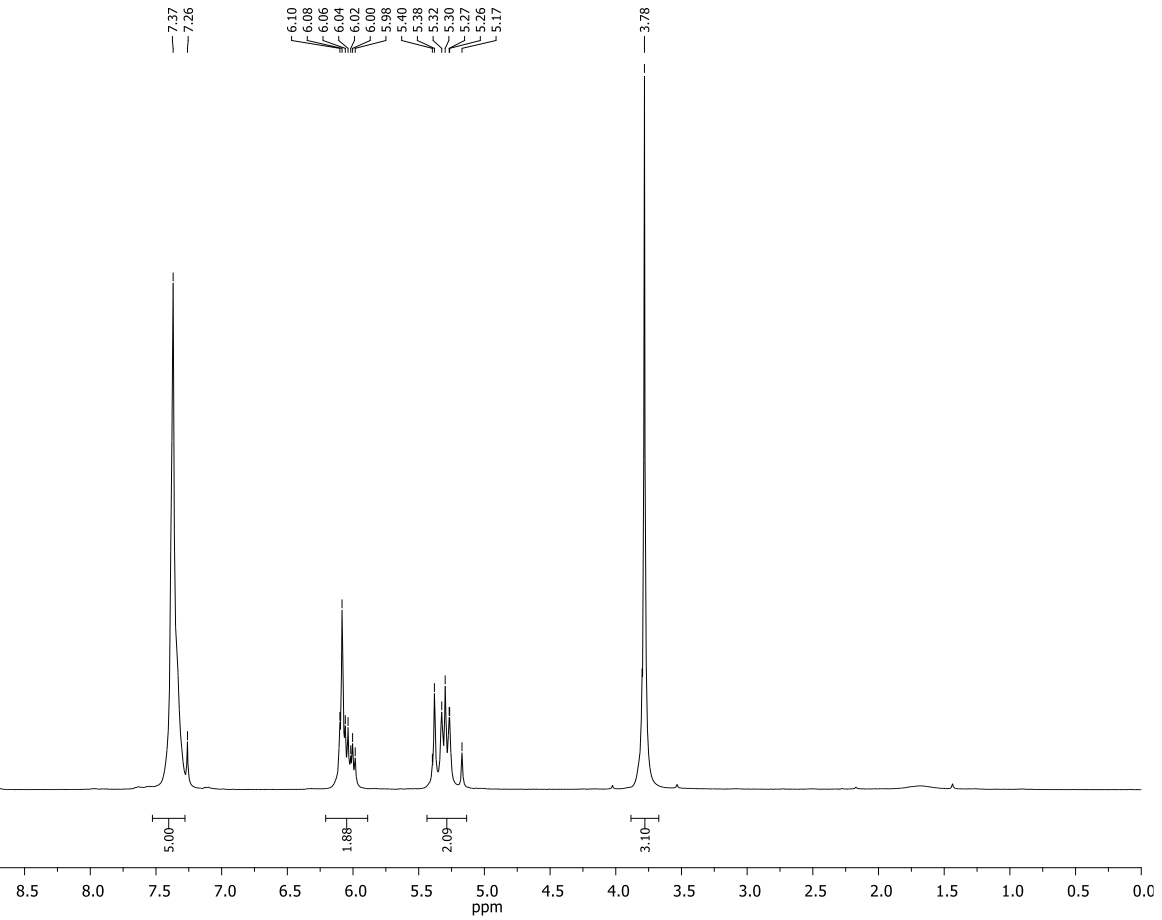

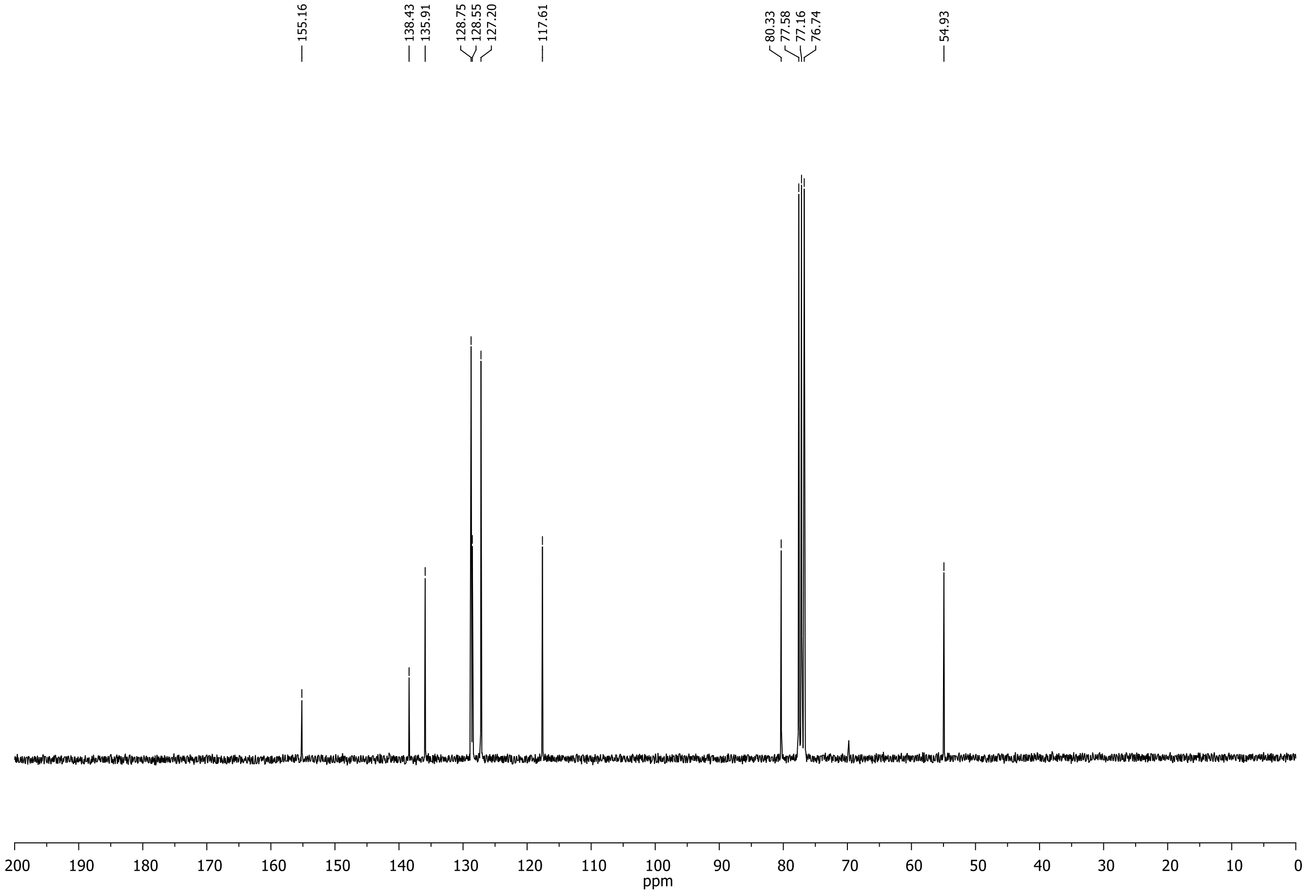

^{1}H-NMR (300.36 MHz, $CDCl_3$); ^{13}C-NMR: (75.53 MHz, $CDCl_3$)

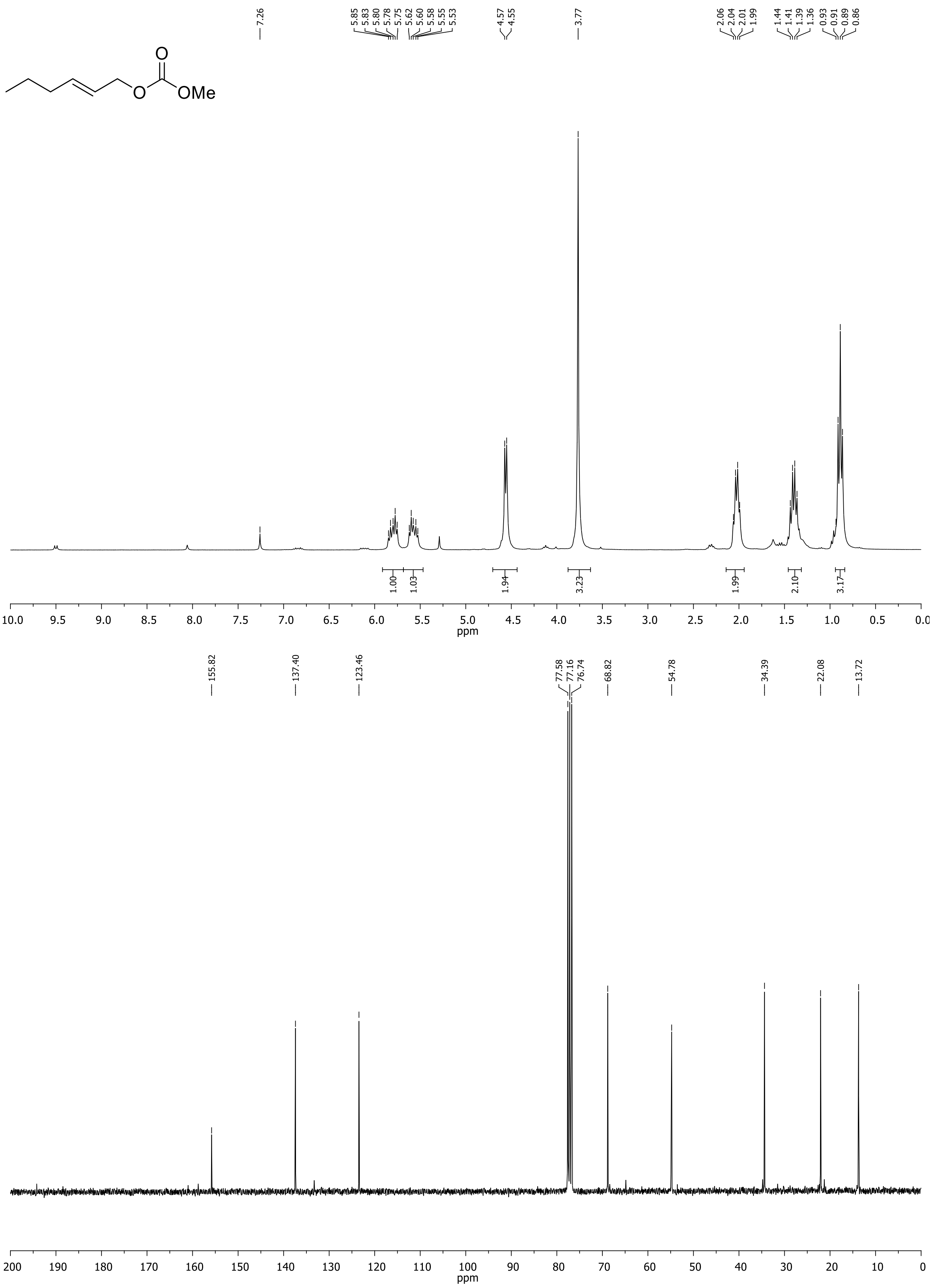

^{1}H-NMR (300.36 MHz, $CDCl_3$); ^{13}C-NMR: (75.53 MHz, $CDCl_3$)

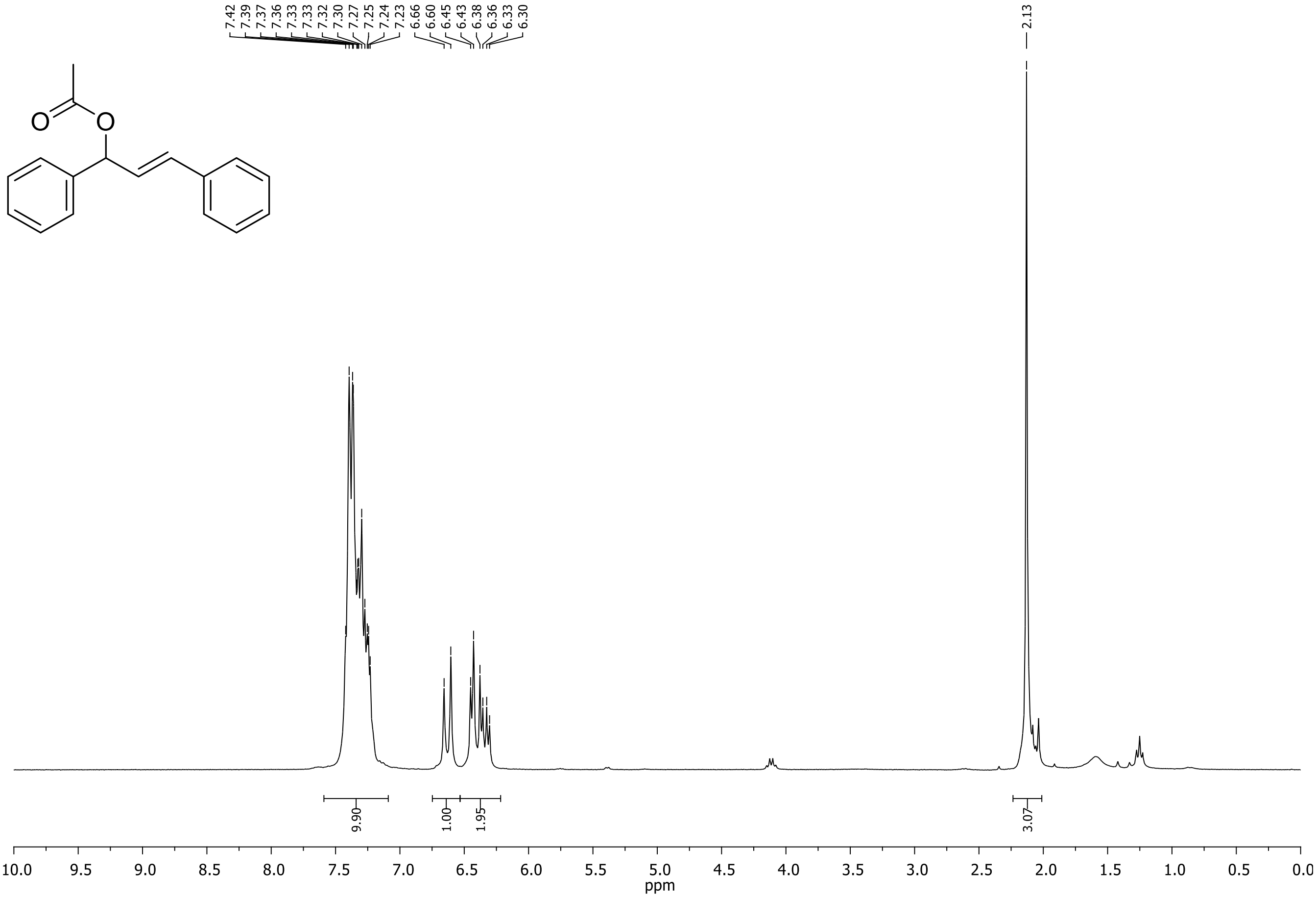

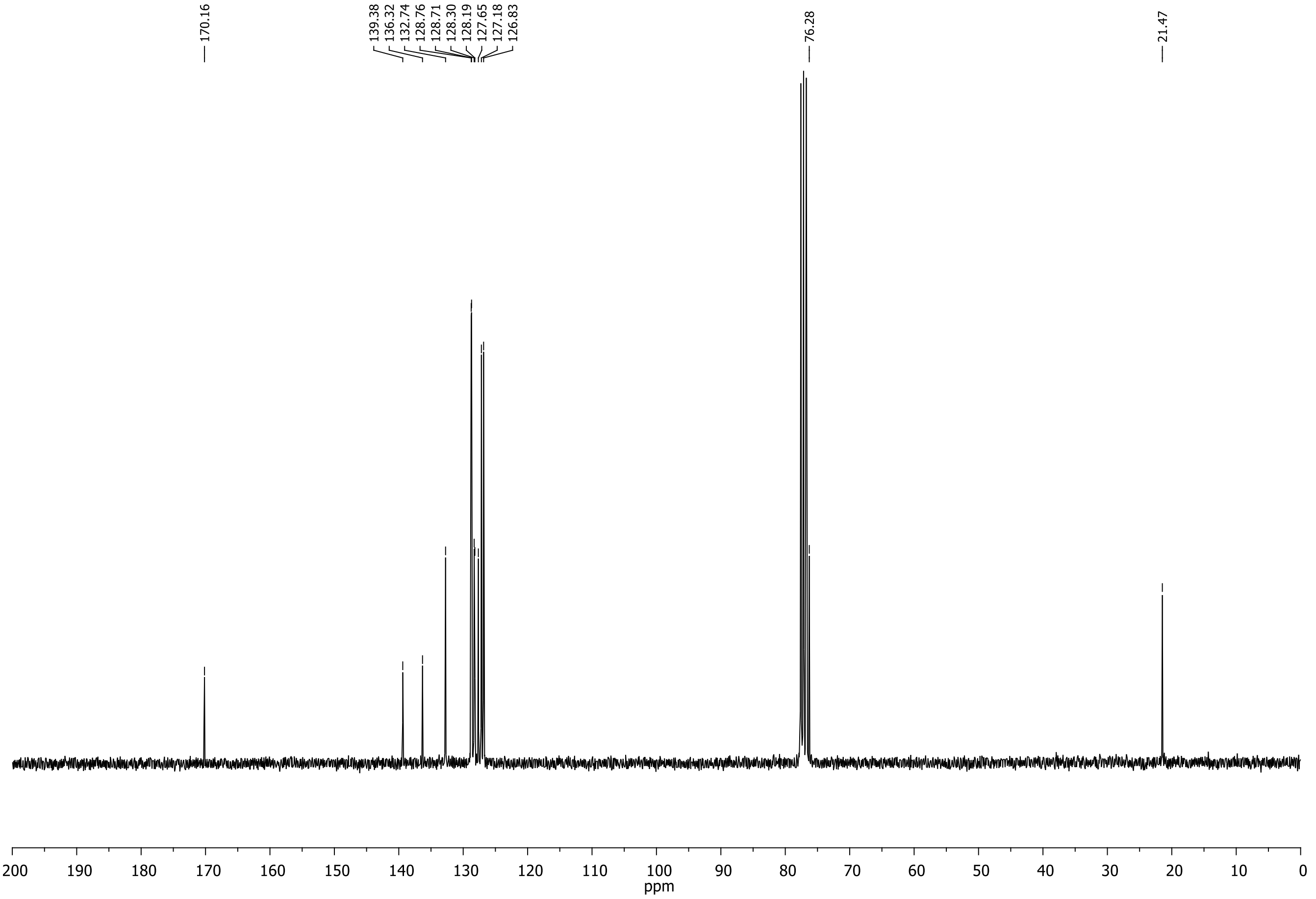

^{1}H-NMR (300.36 MHz, $CDCl_3$); ^{13}C-NMR: (75.53 MHz, $CDCl_3$)

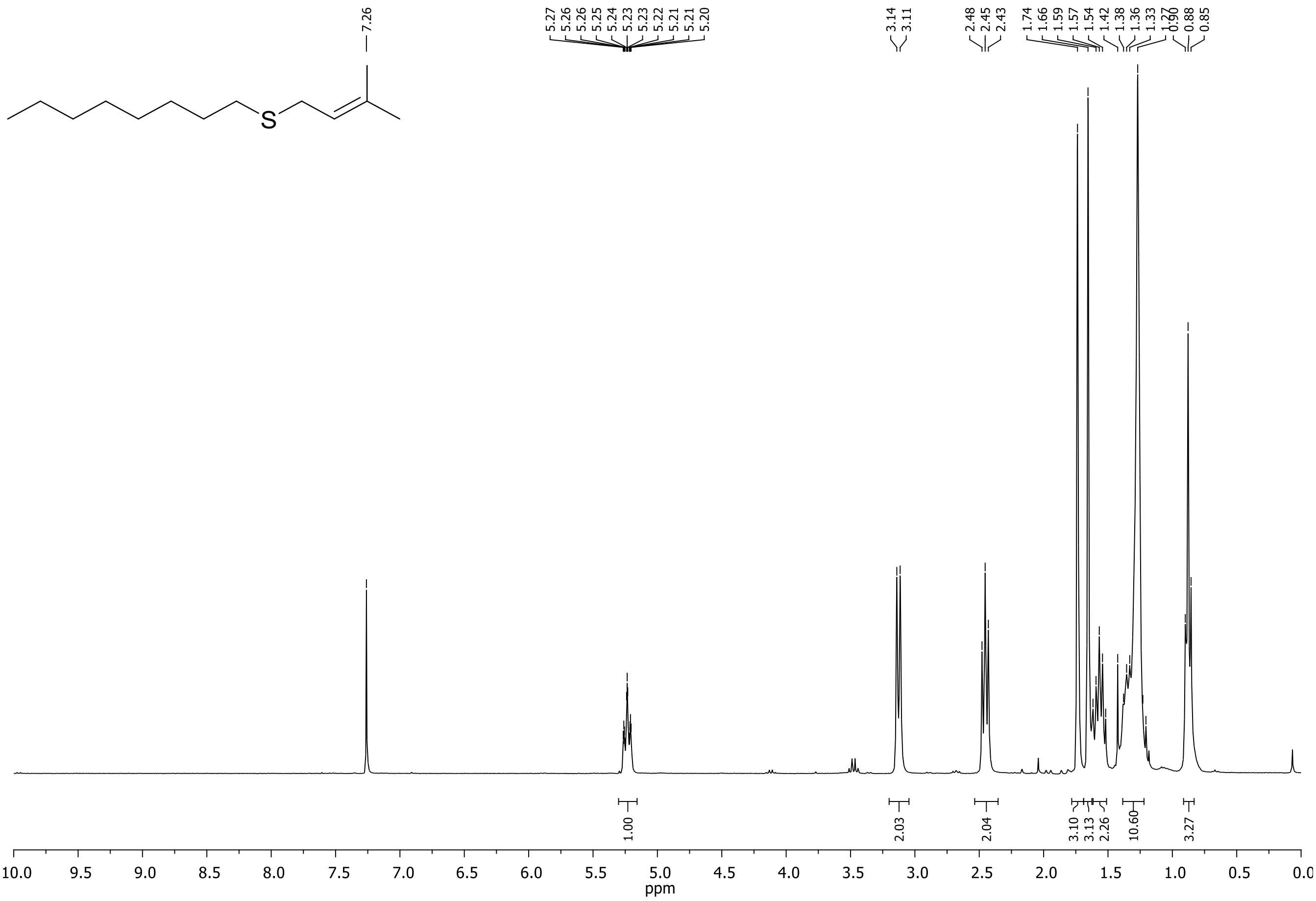

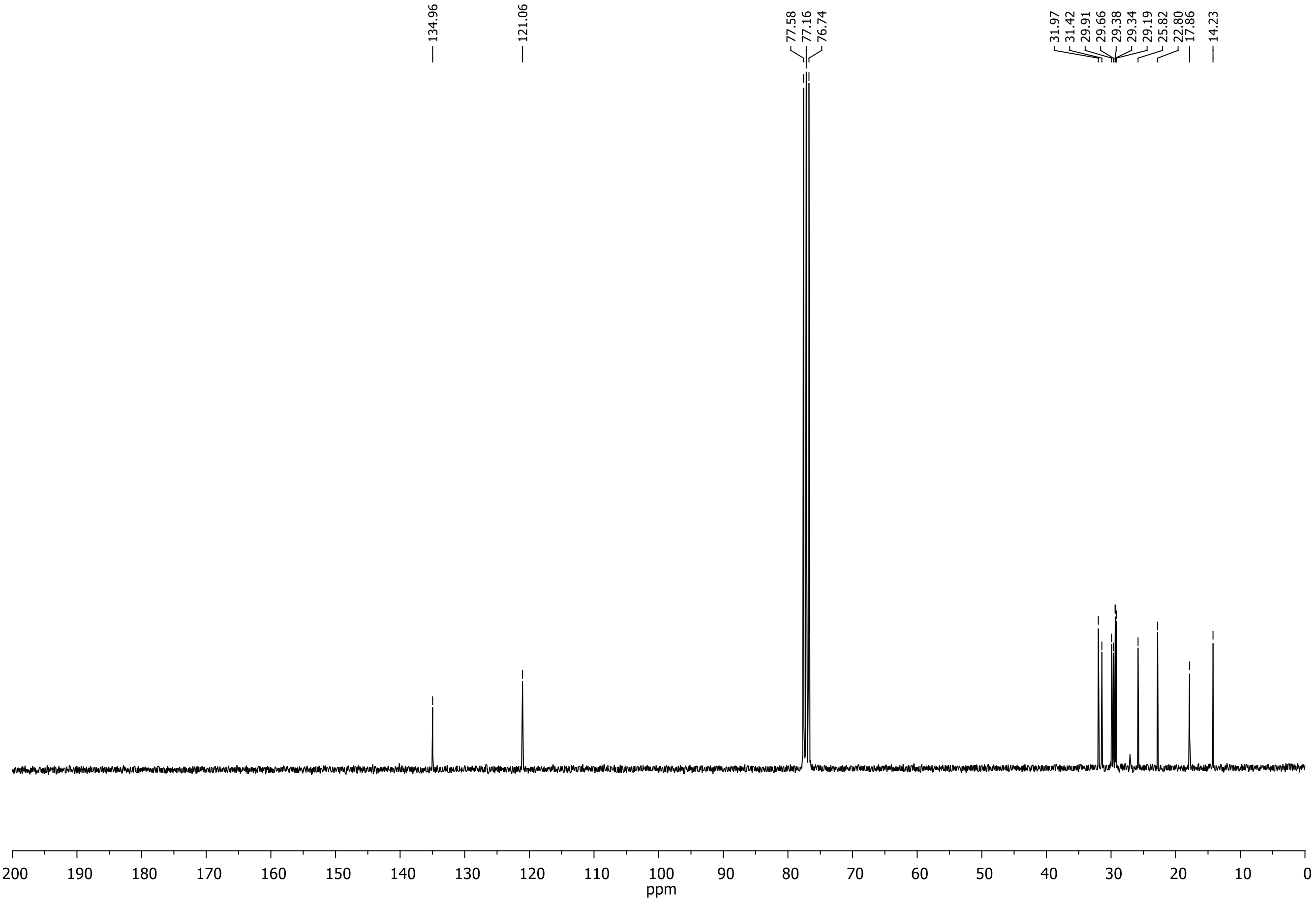

^{1}H-NMR (300.36 MHz, $CDCl_3$); ^{13}C-NMR: (75.53 MHz, $CDCl_3$)

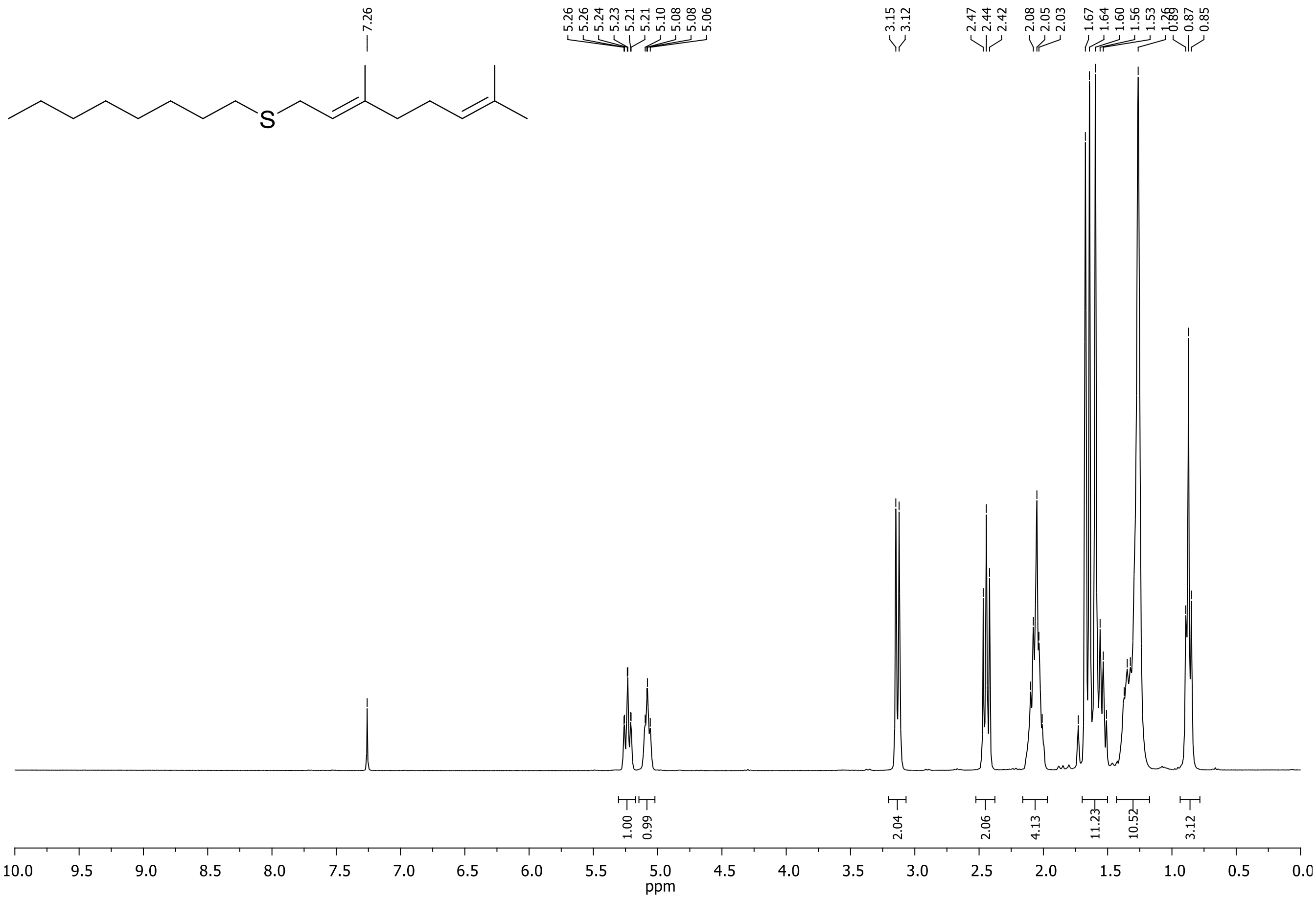

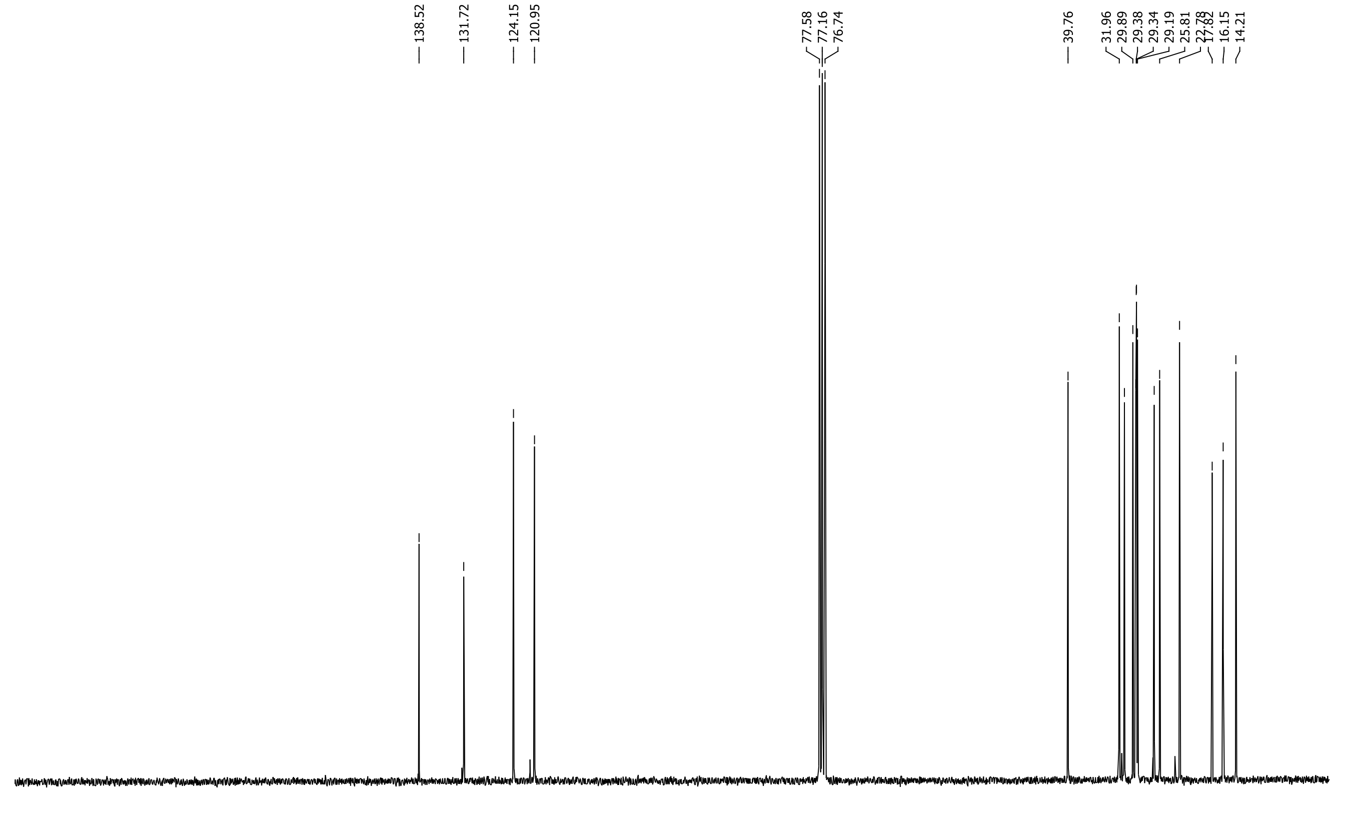

^{1}H-NMR (300.36 MHz, $CDCl_3$); ^{13}C-NMR: (75.53 MHz, $CDCl_3$)

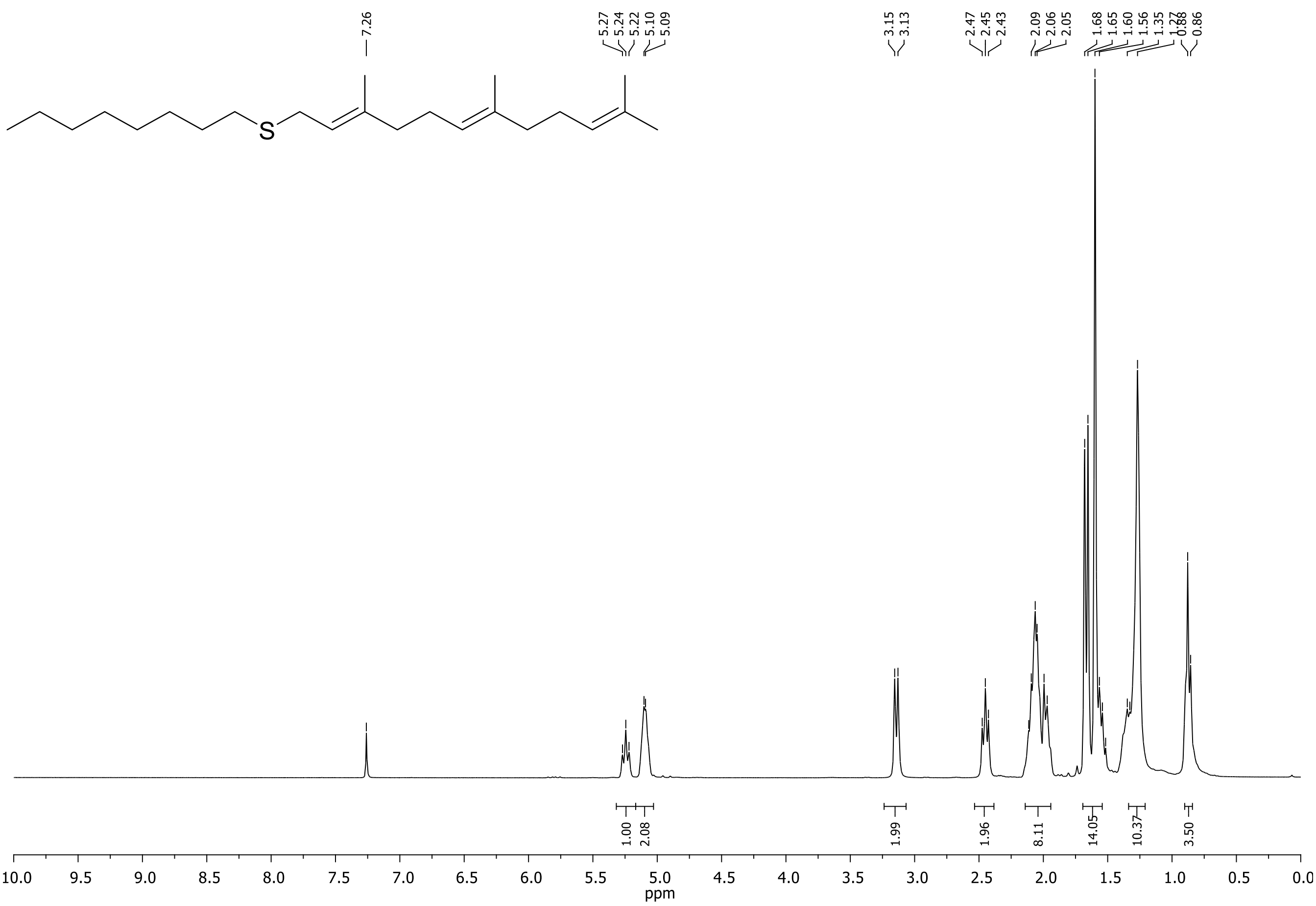

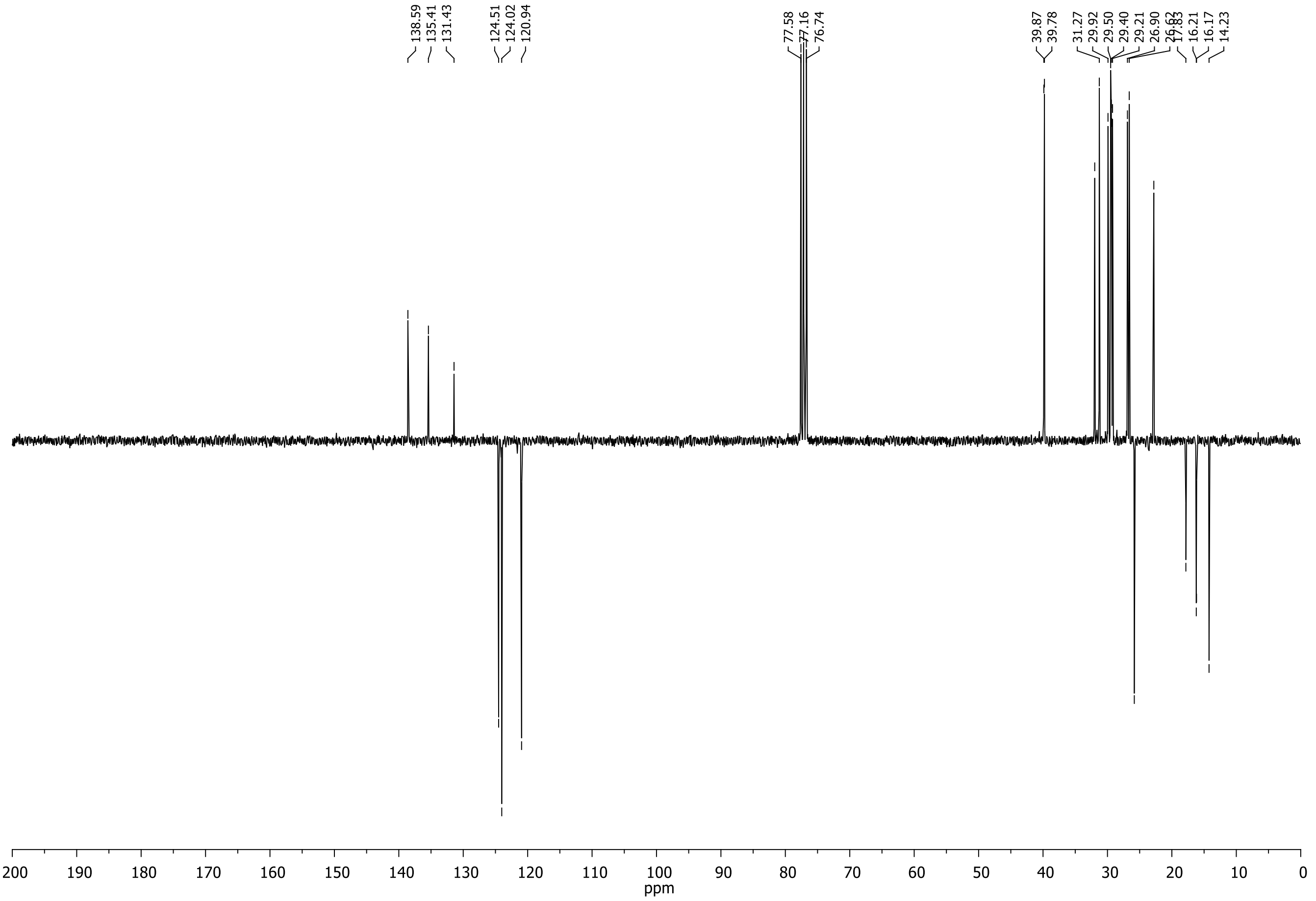

^{1}H-NMR (300.36 MHz, $CDCl_3$); ^{13}C-NMR: (75.53 MHz, $CDCl_3$)

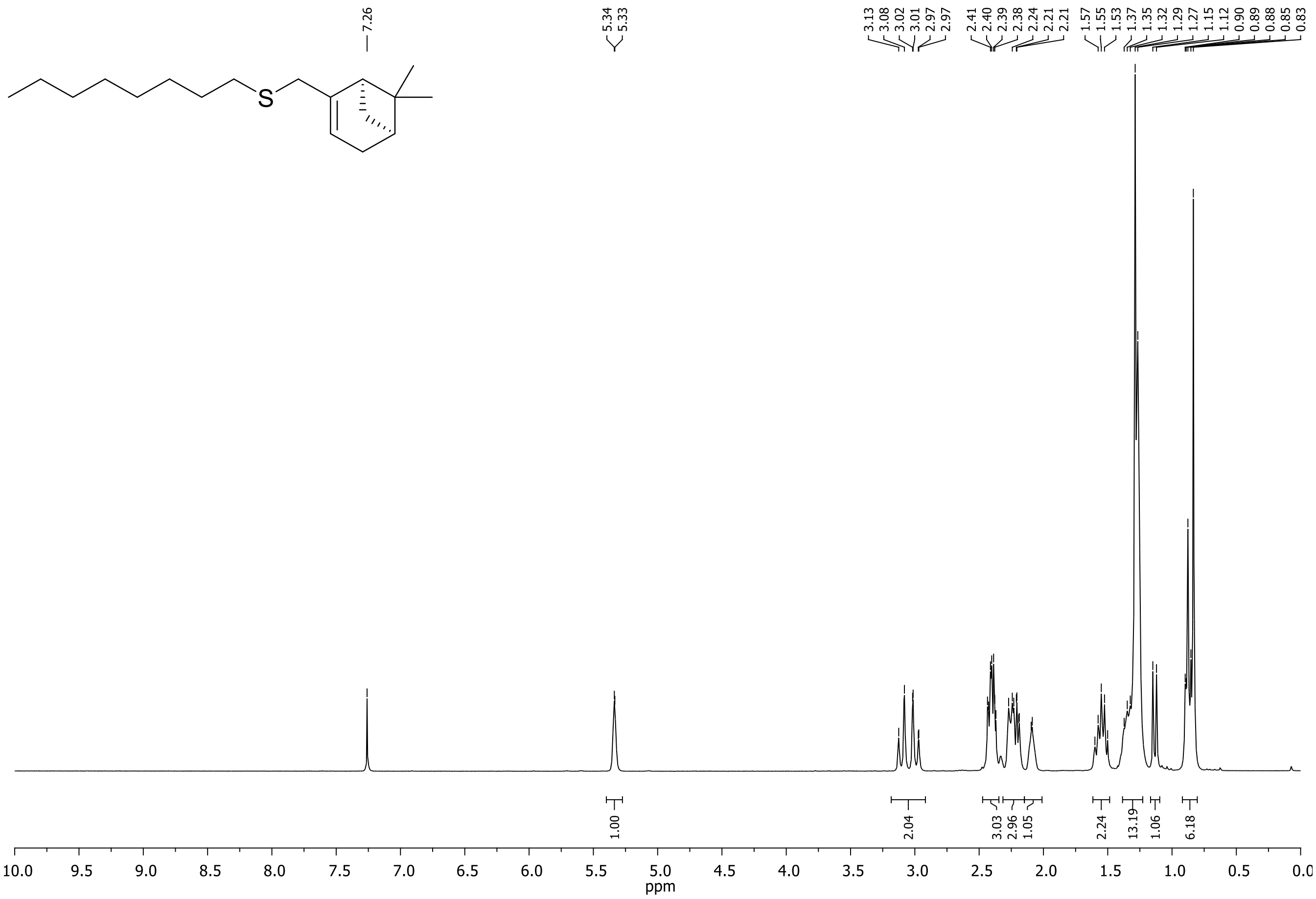

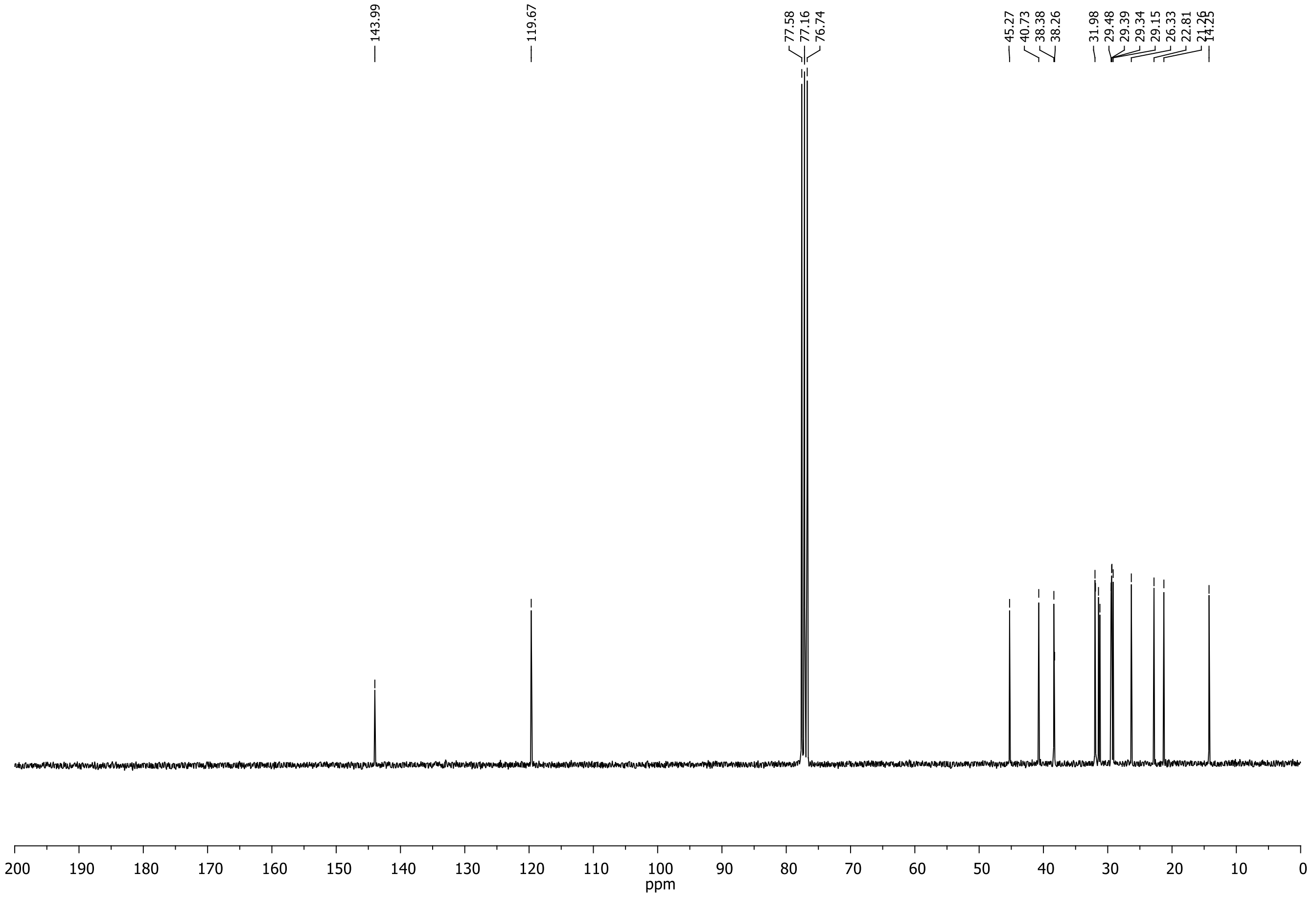

^{1}H-NMR (300.36 MHz, $CDCl_3$); ^{13}C-NMR: (75.53 MHz, $CDCl_3$)

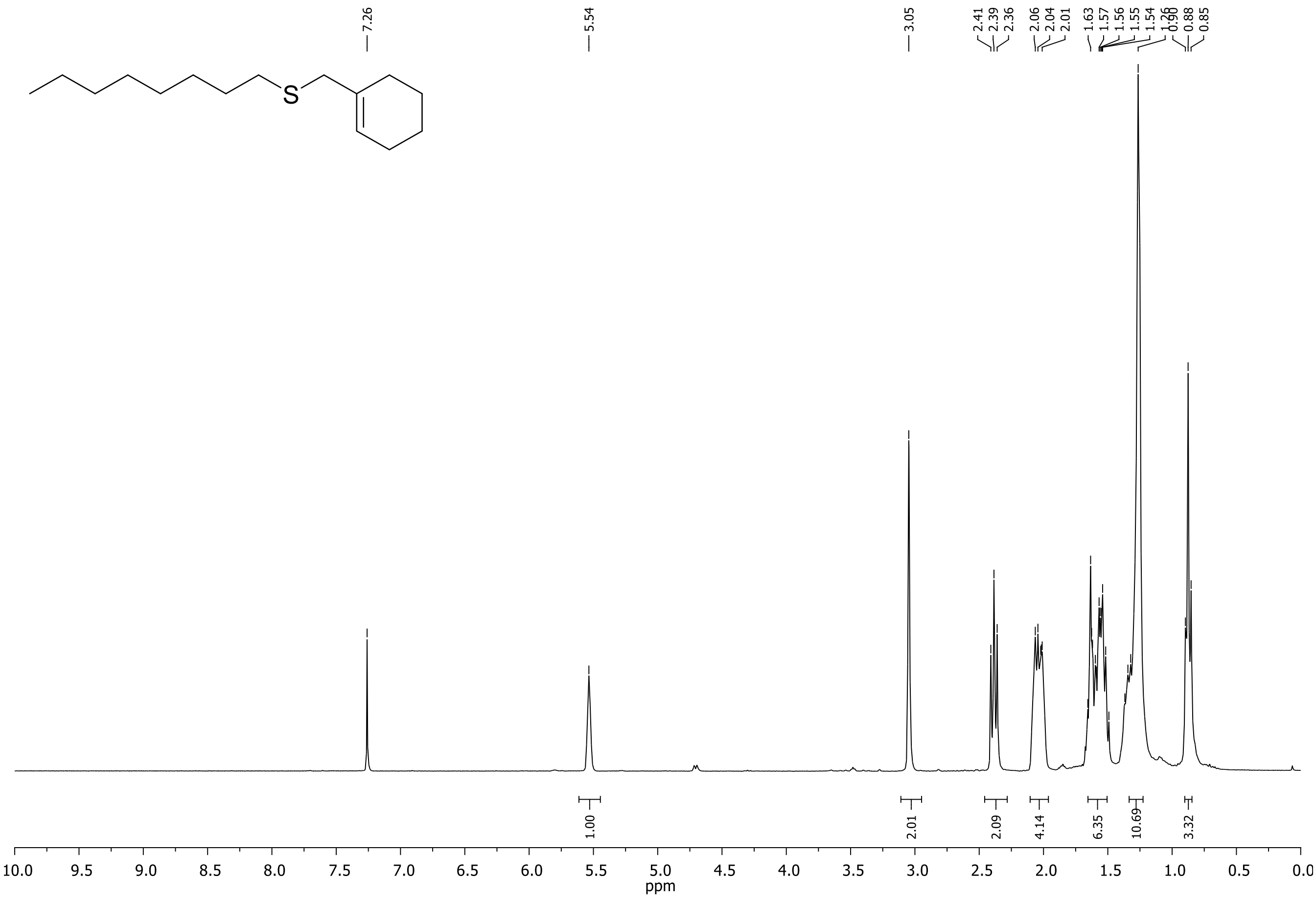

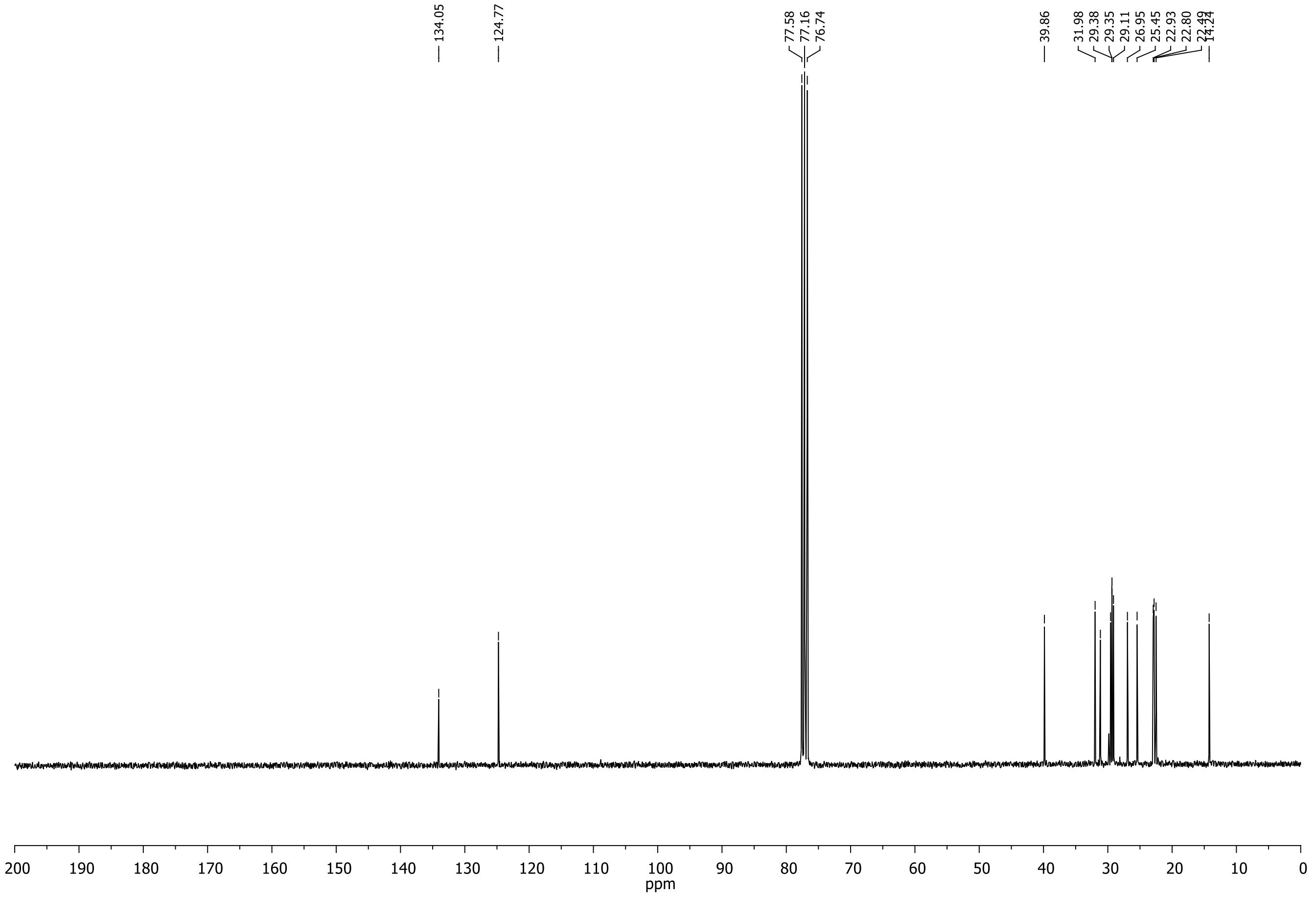

^{1}H-NMR (300.36 MHz, $CDCl_3$); ^{13}C-NMR: (75.53 MHz, $CDCl_3$)

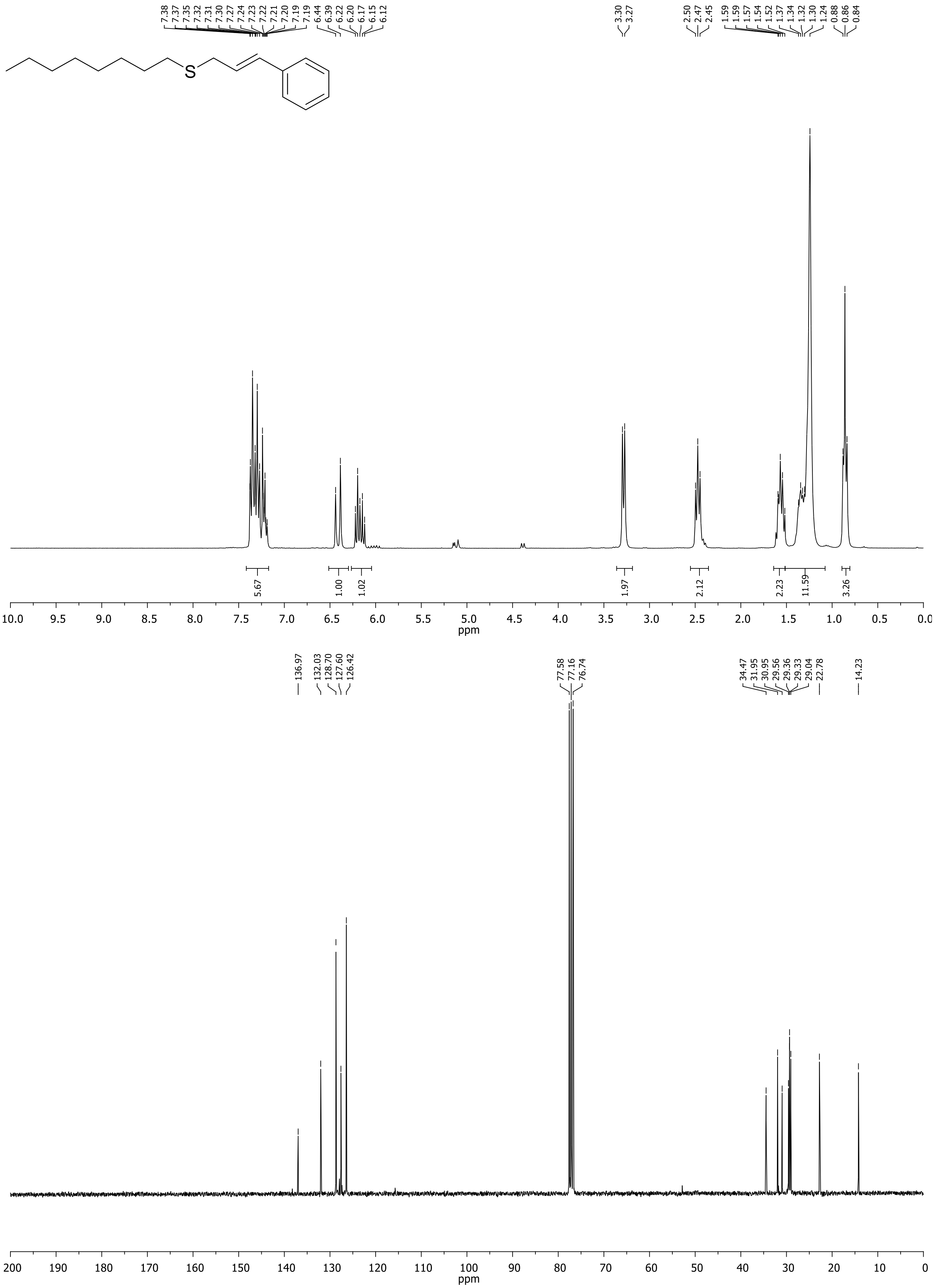

^{1}H-NMR (300.36 MHz, $CDCl_3$); ^{13}C-NMR: (75.53 MHz, $CDCl_3$)

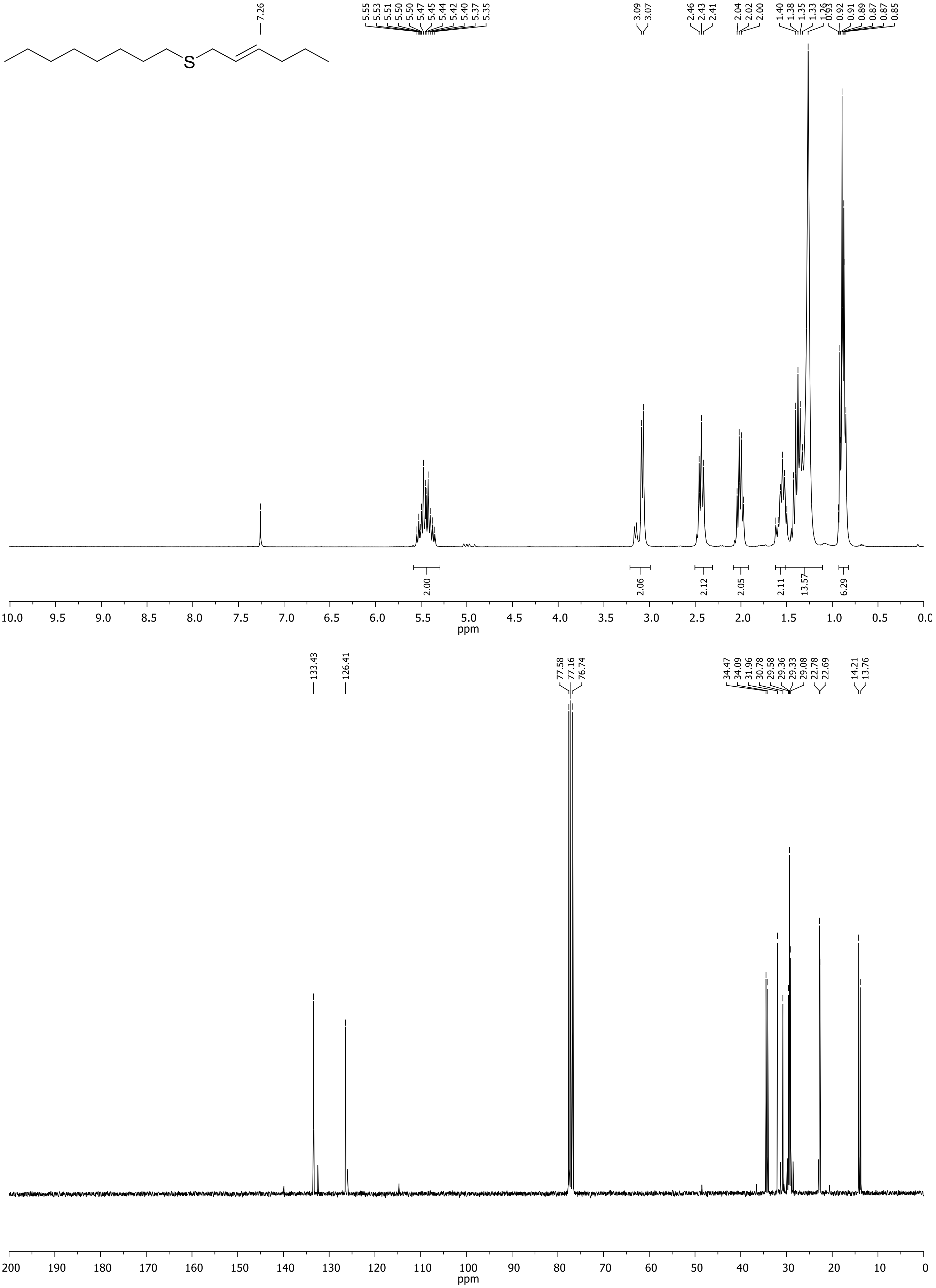

^{1}H-NMR (300.36 MHz, $CDCl_3$); ^{13}C-NMR: (75.53 MHz, $CDCl_3$)

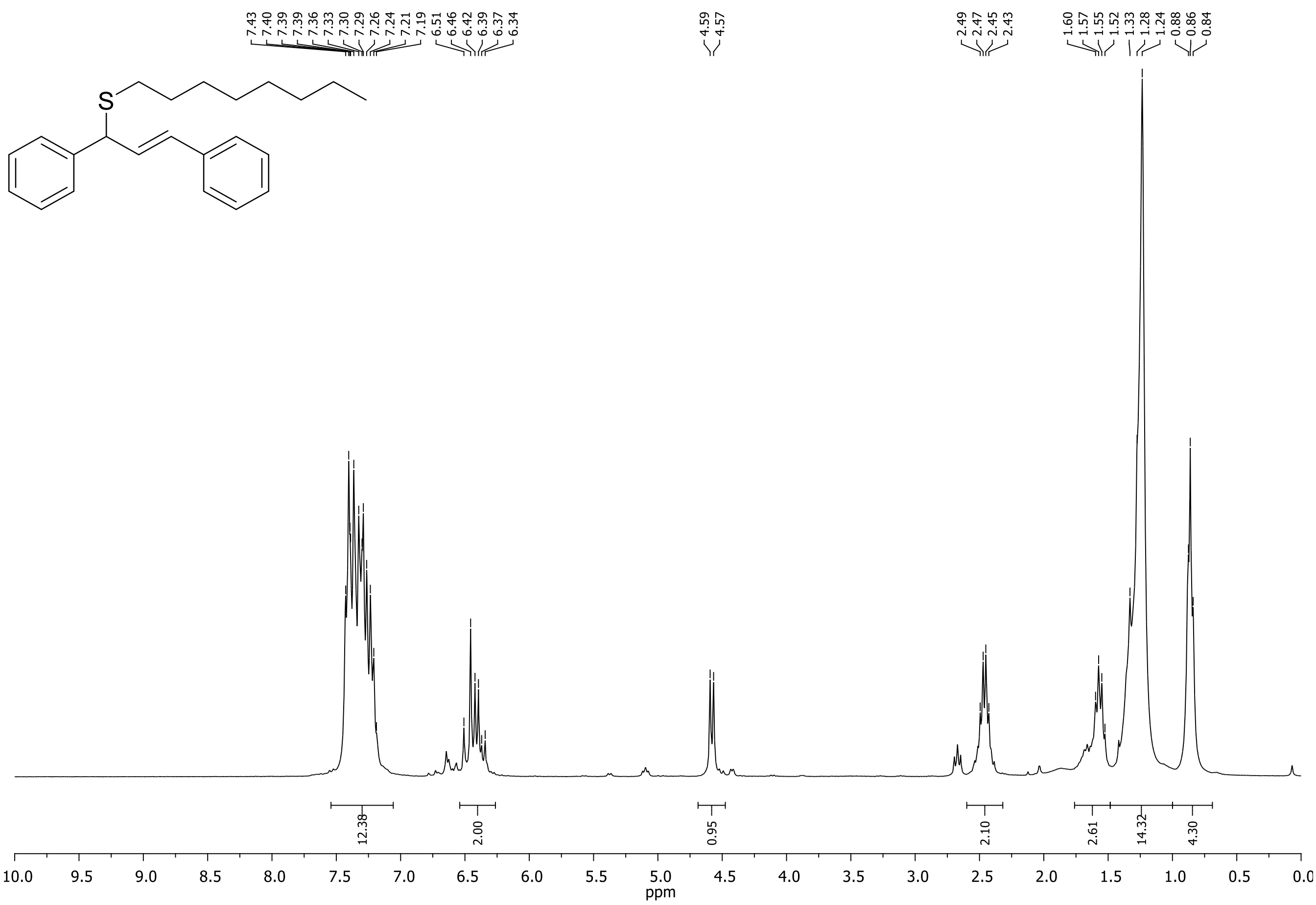

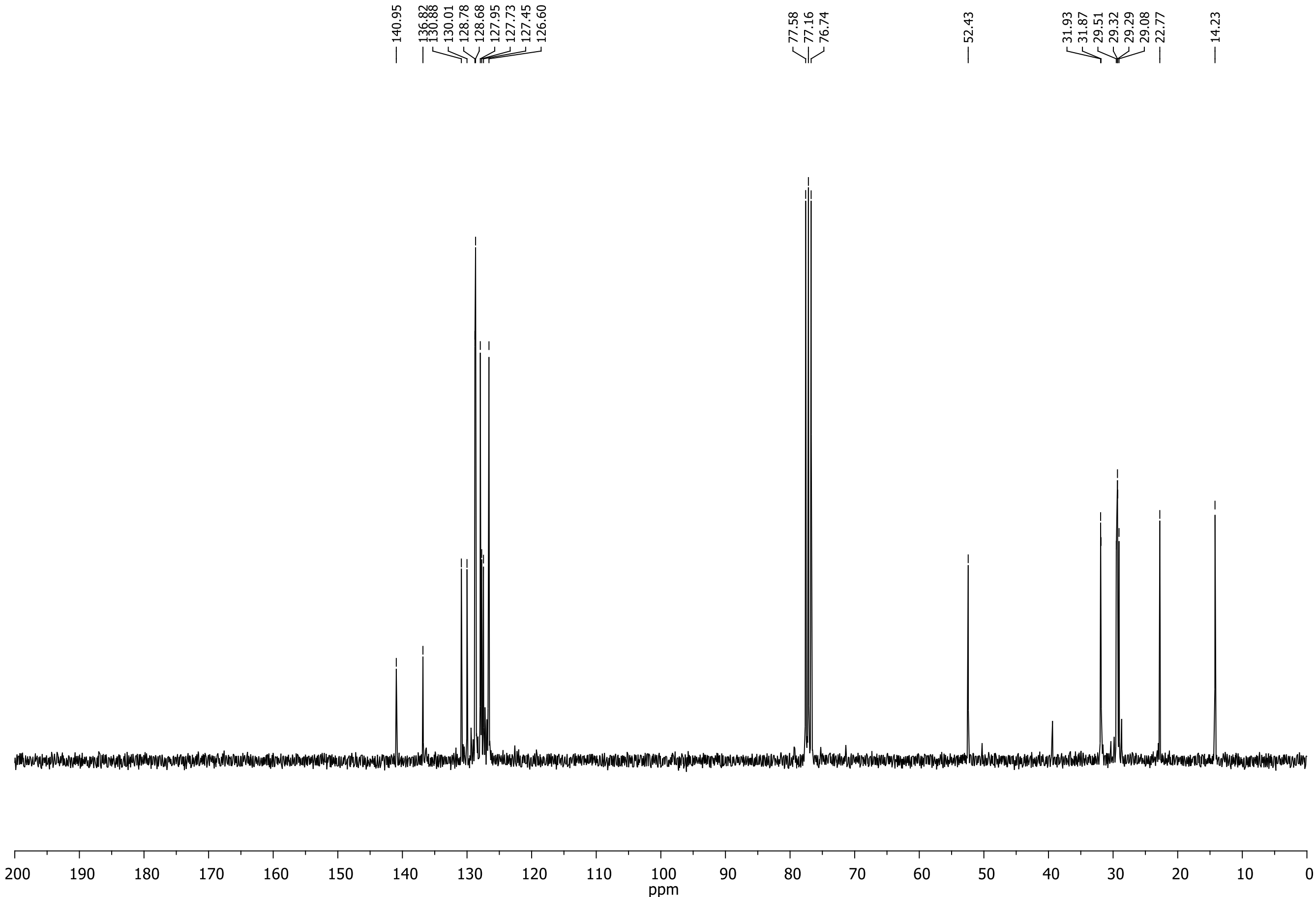

^{1}H-NMR (300.36 MHz, $CDCl_3$); ^{13}C-NMR: (75.53 MHz, $CDCl_3$)

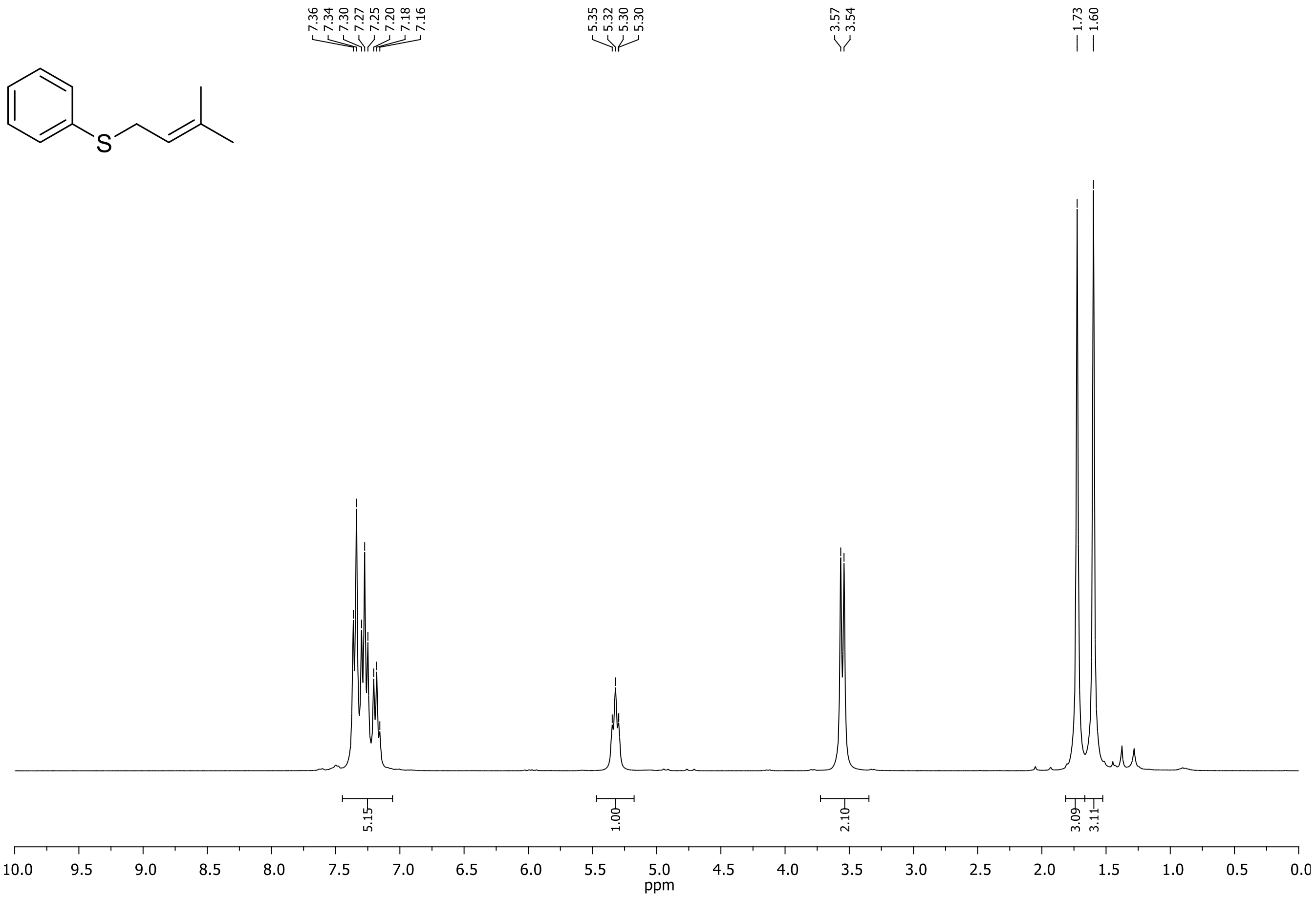

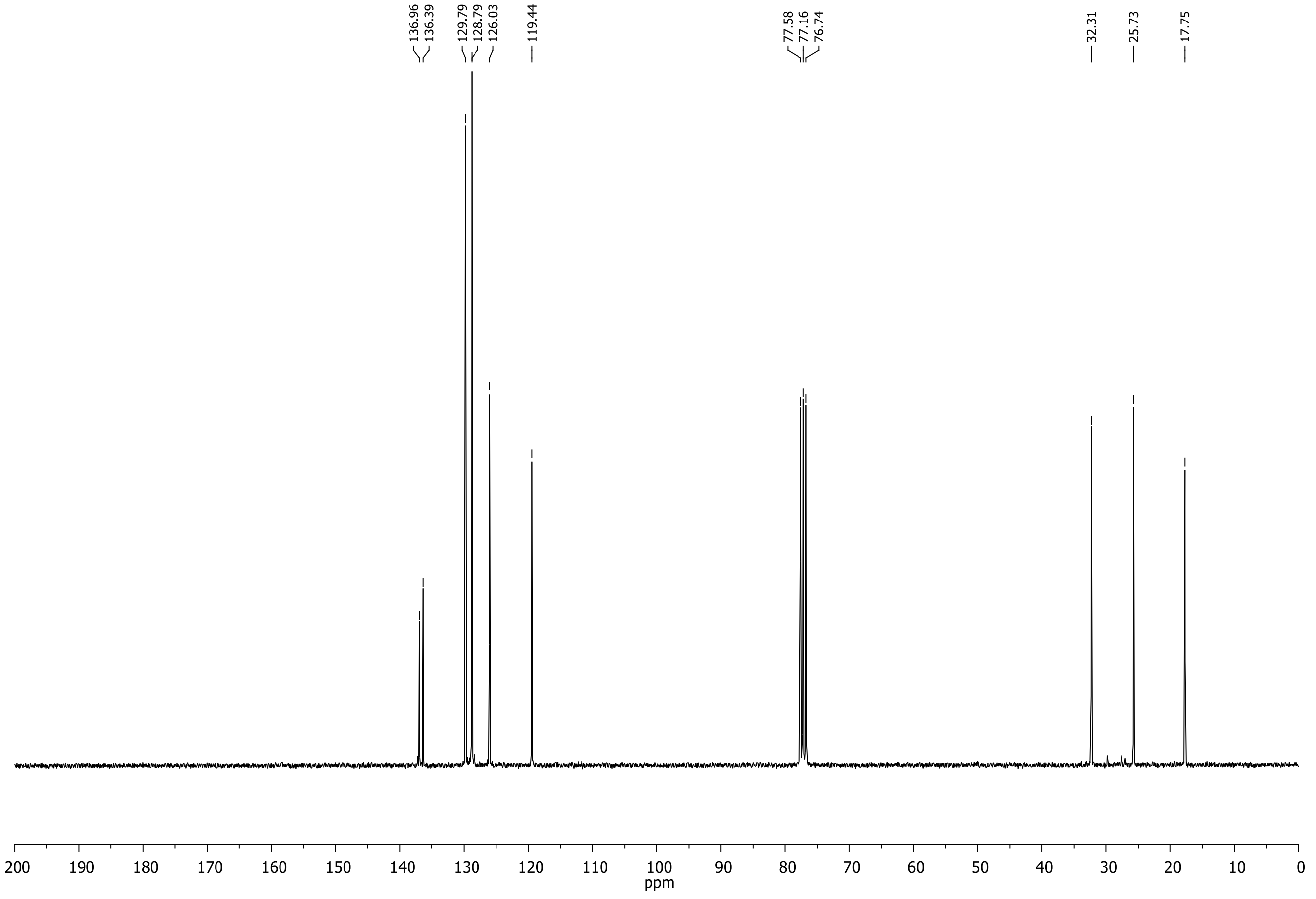

^{1}H-NMR (300.36 MHz, $CDCl_3$); ^{13}C-NMR: (75.53 MHz, $CDCl_3$)

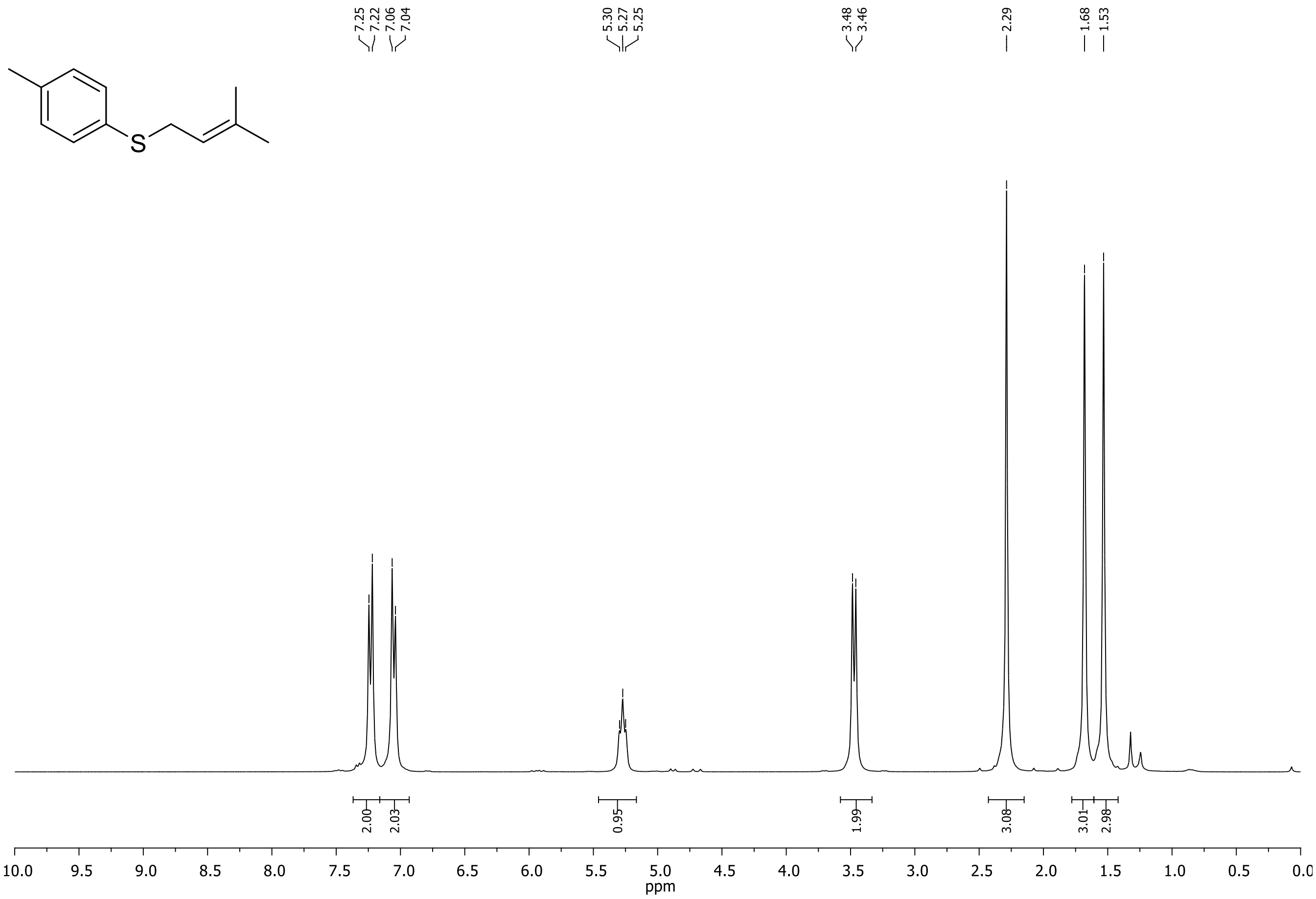

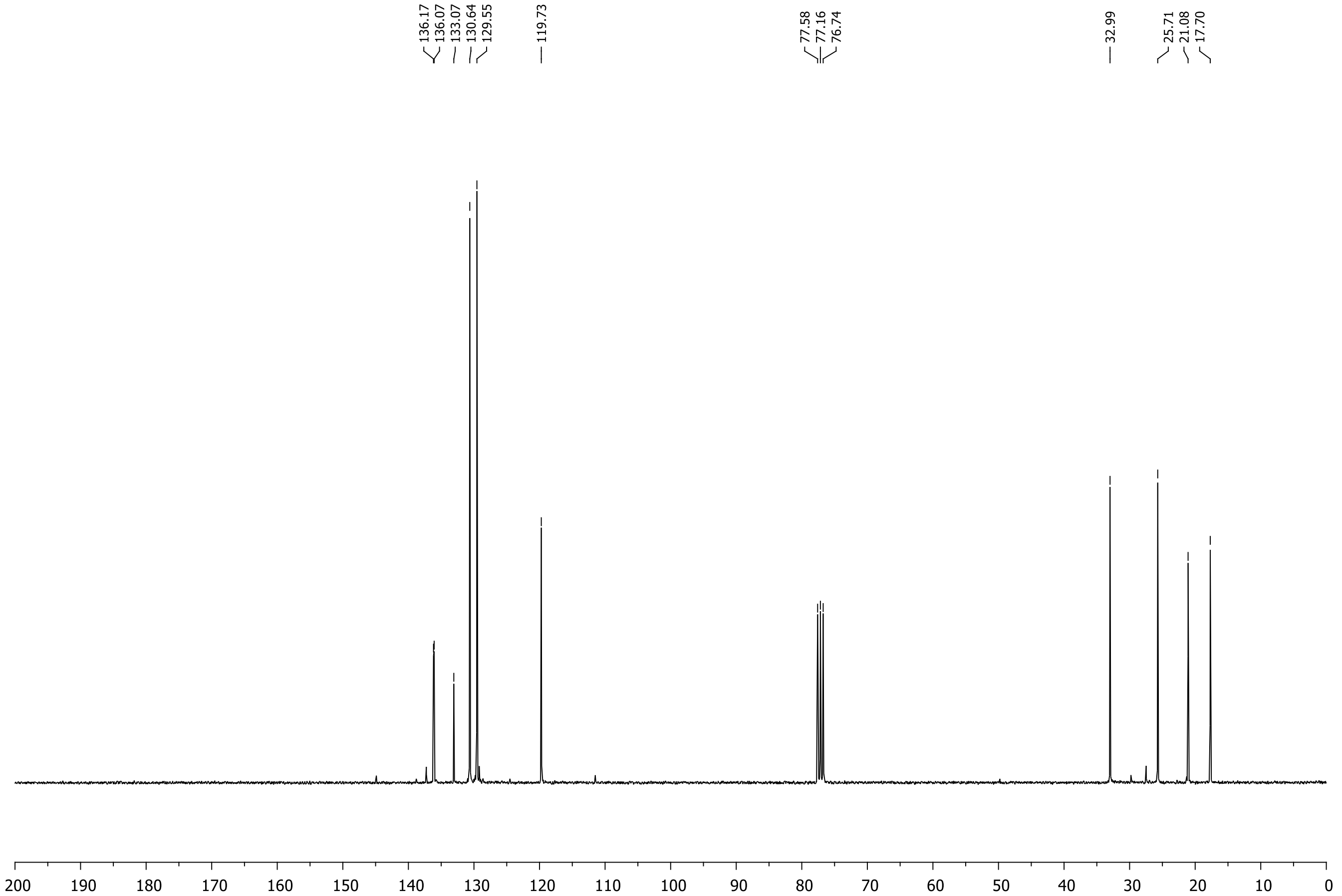

^{1}H-NMR (300.36 MHz, $CDCl_3$); ^{13}C-NMR: (75.53 MHz, $CDCl_3$)

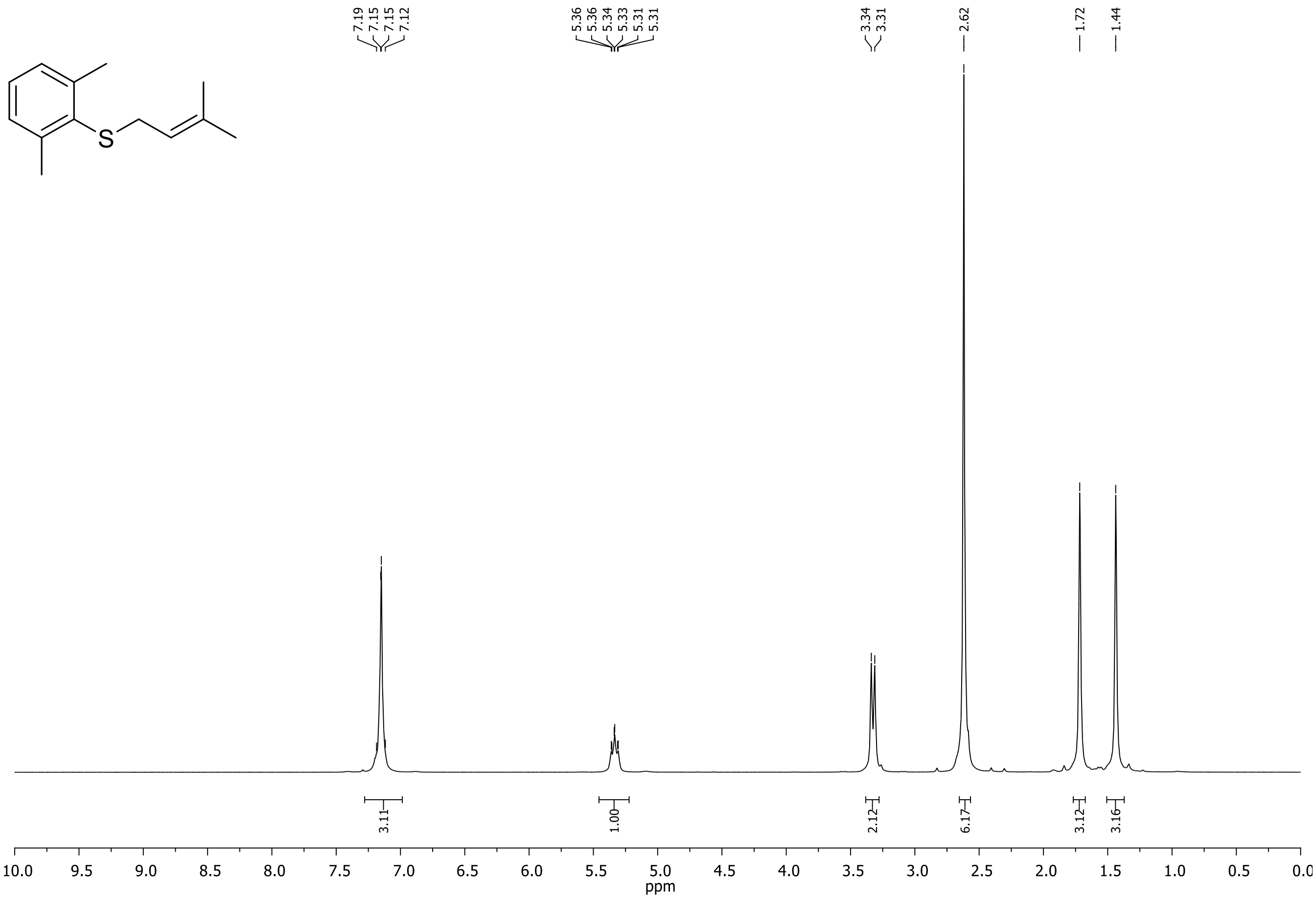

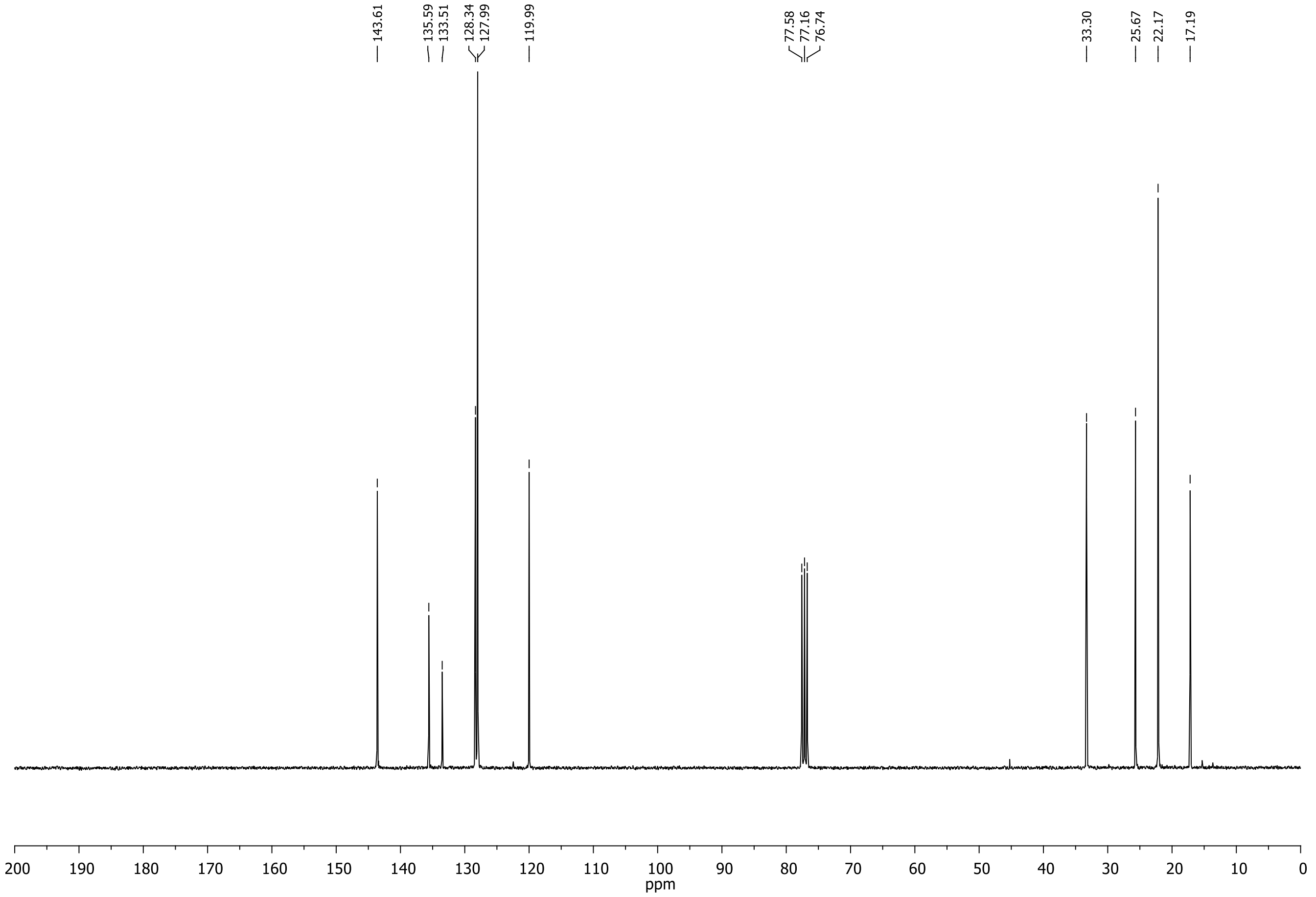

^{1}H-NMR (300.36 MHz, $CDCl_3$); ^{13}C-NMR: (75.53 MHz, $CDCl_3$)

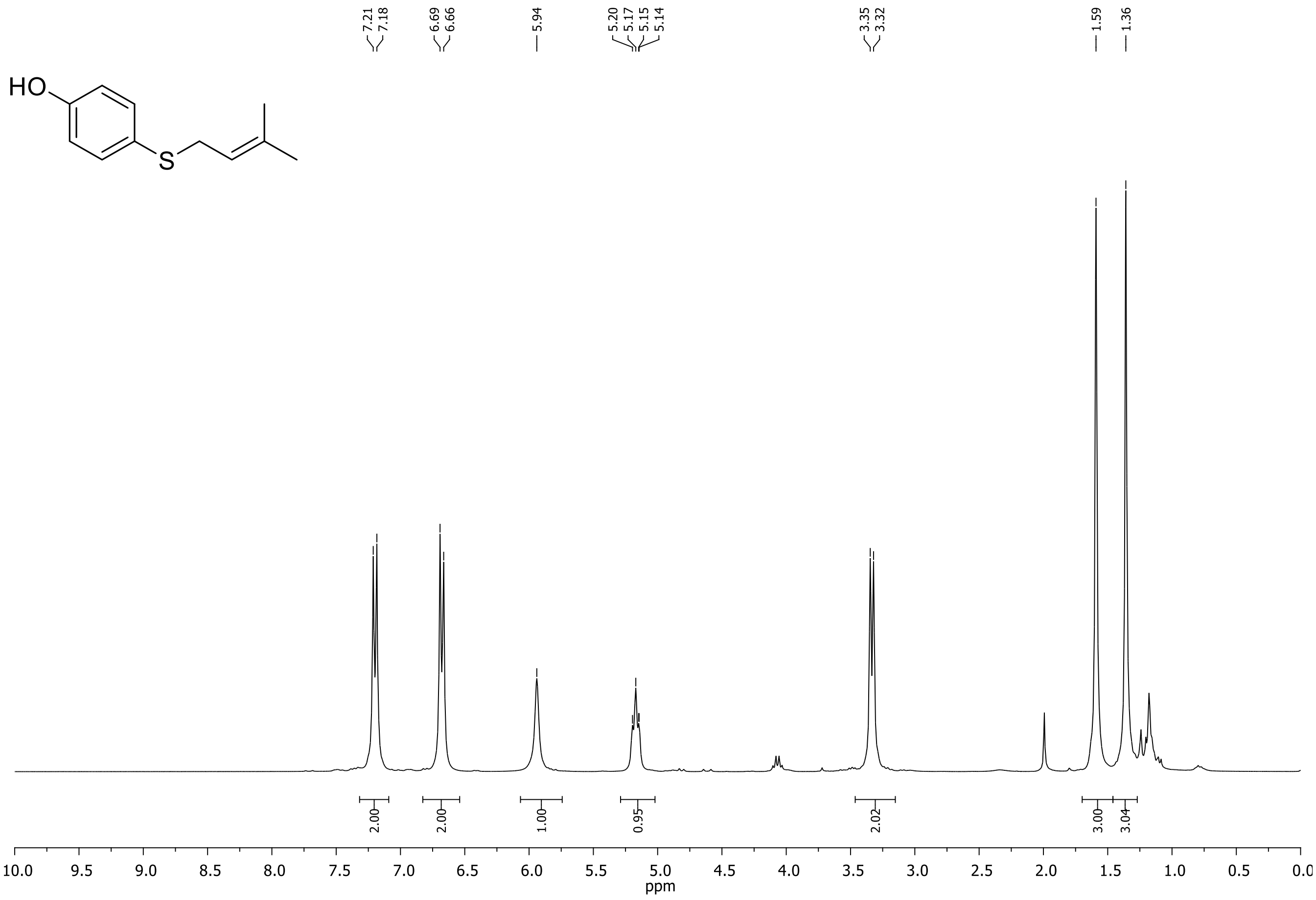

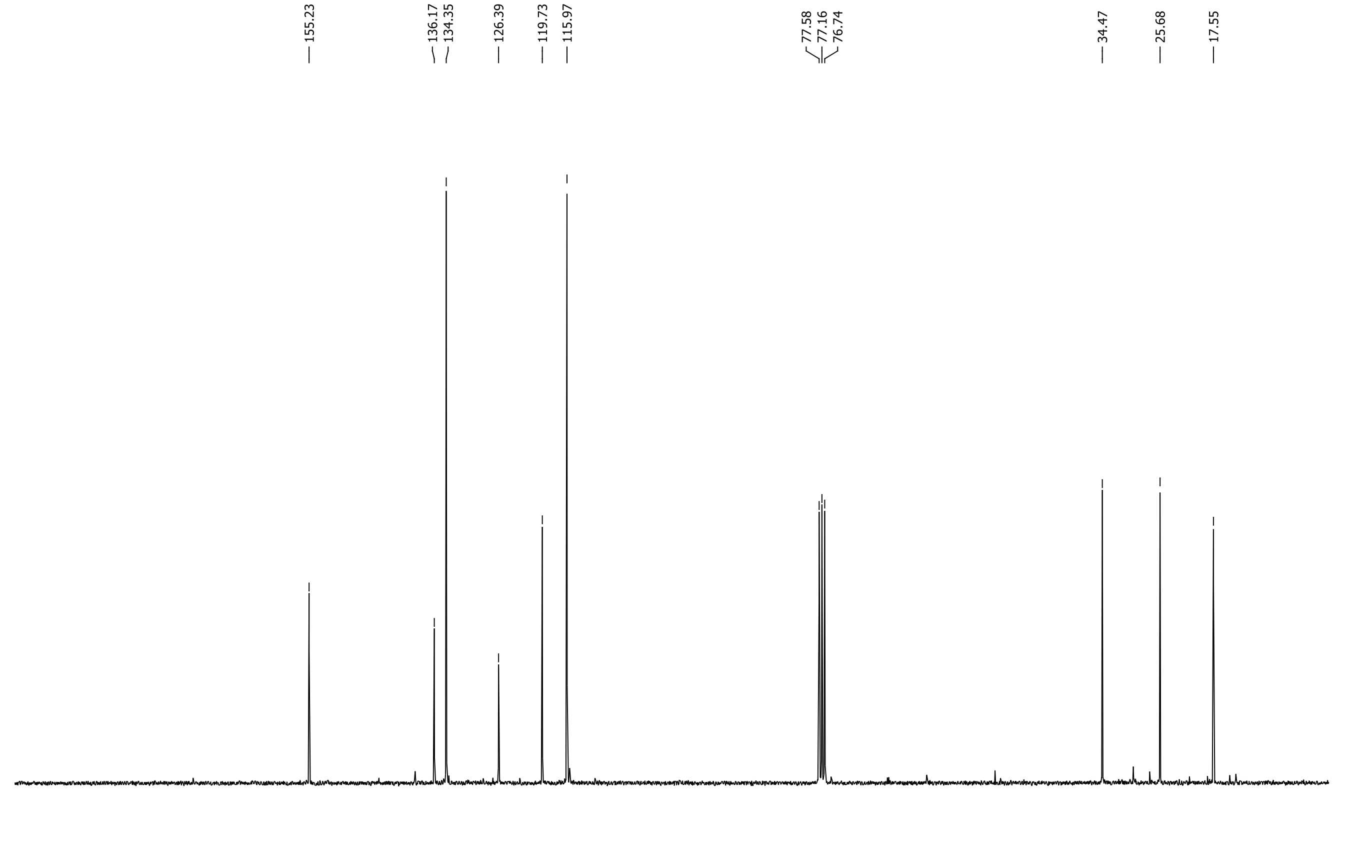

^{1}H-NMR (300.36 MHz, $CDCl_3$); ^{13}C-NMR: (75.53 MHz, $CDCl_3$)

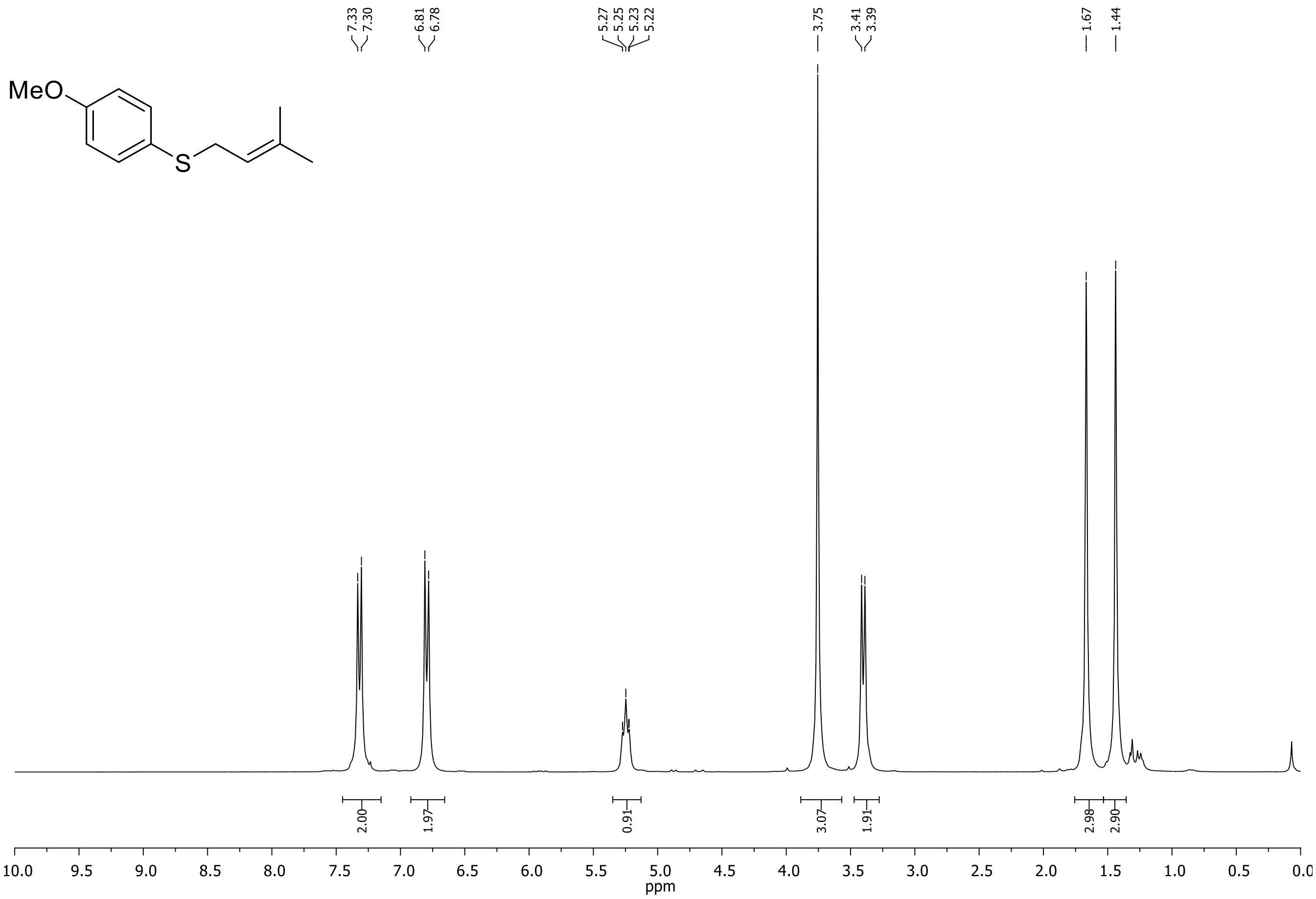

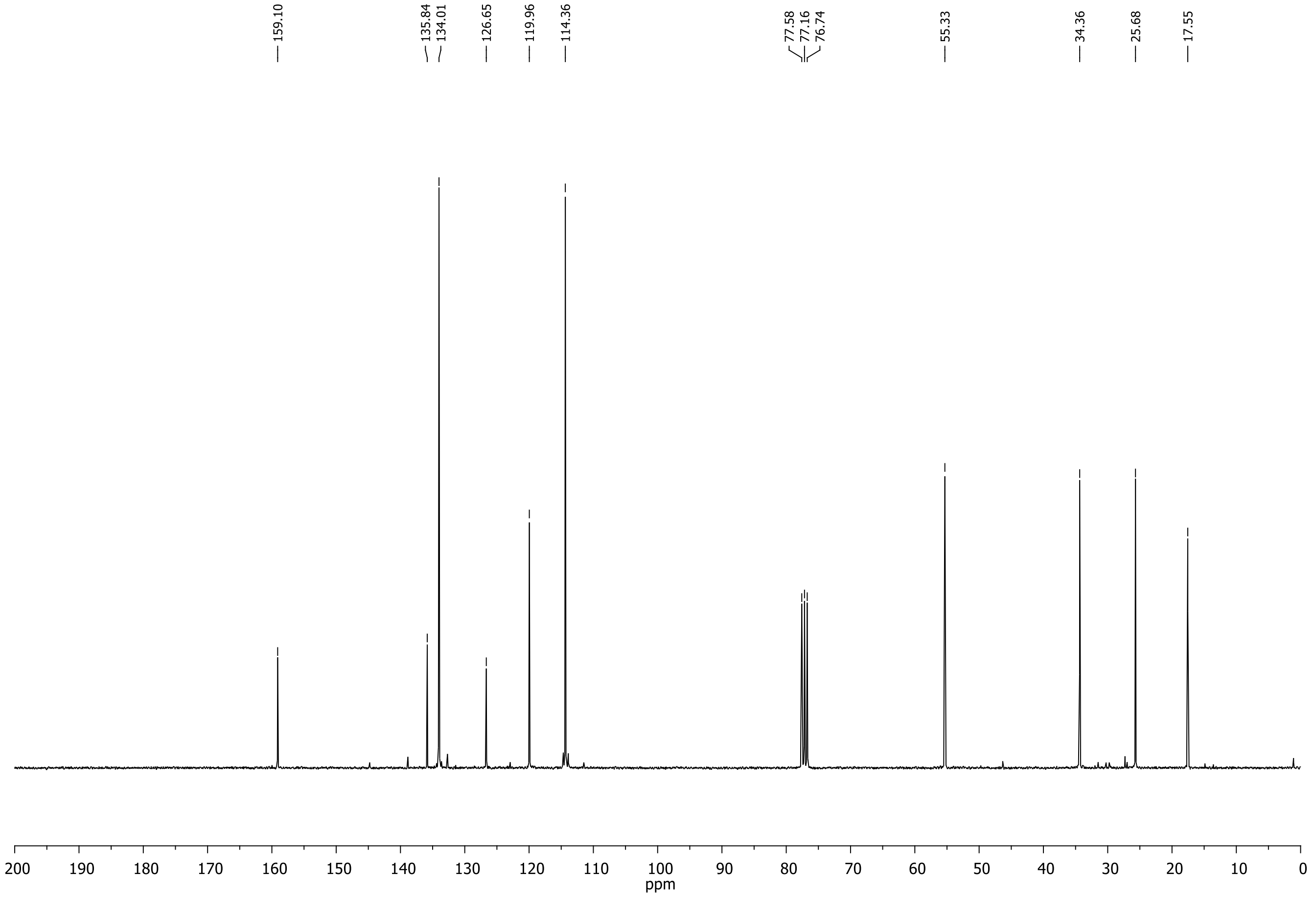

^{1}H-NMR (300.36 MHz, $CDCl_3$); ^{13}C-NMR: (75.53 MHz, $CDCl_3$)

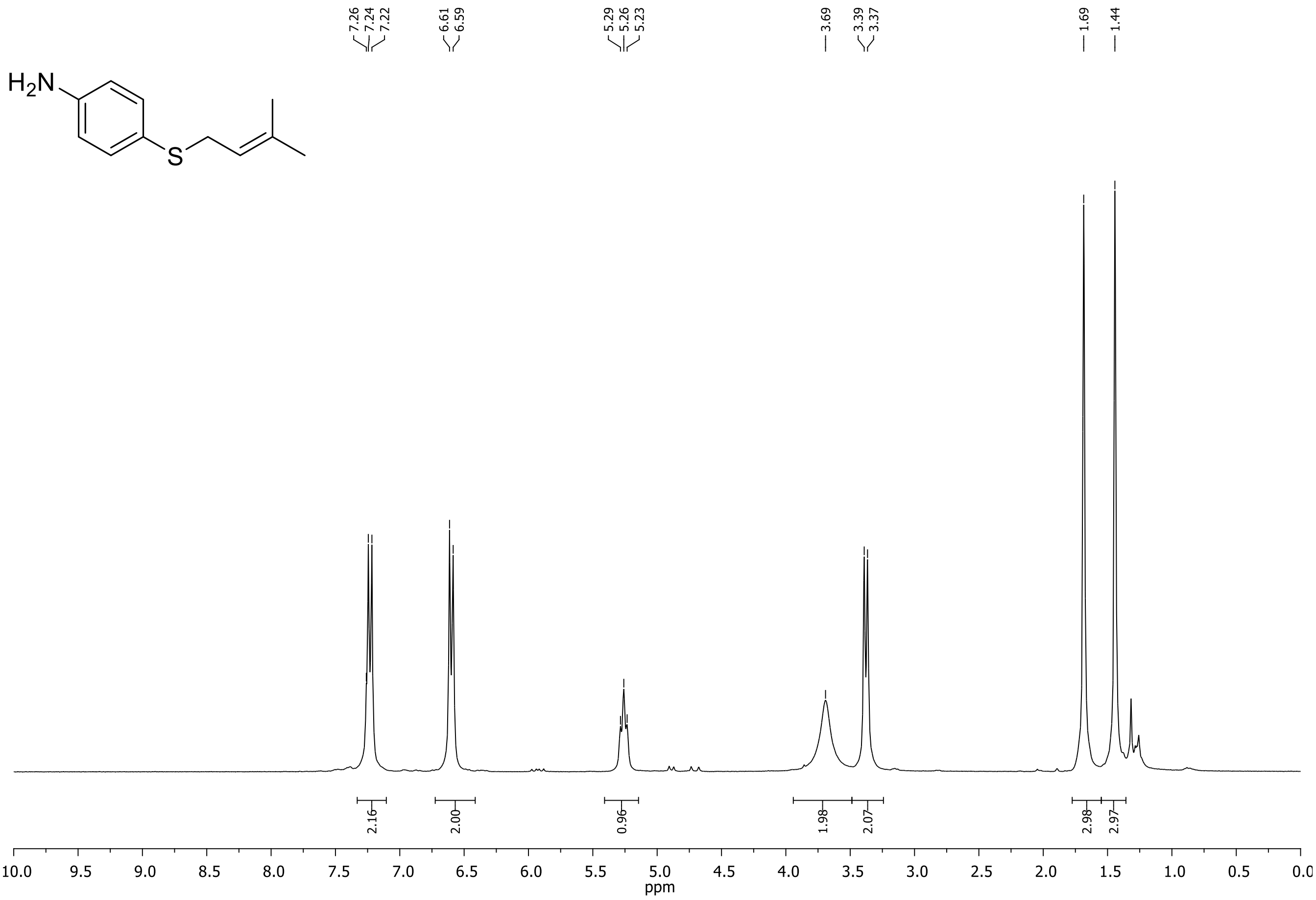

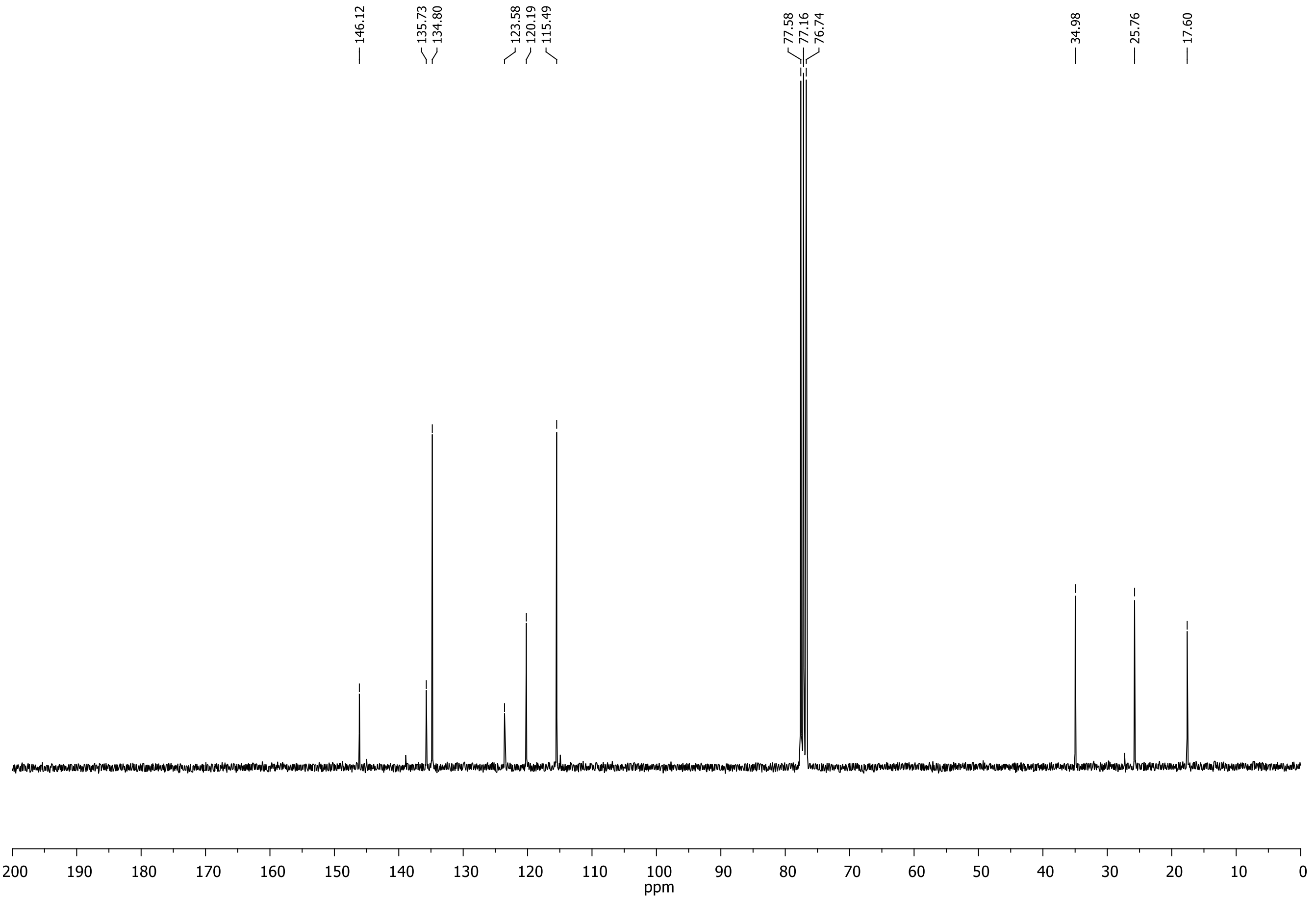

^{1}H-NMR (300.36 MHz, $CDCl_3$); ^{13}C-NMR: (75.53 MHz, $CDCl_3$)

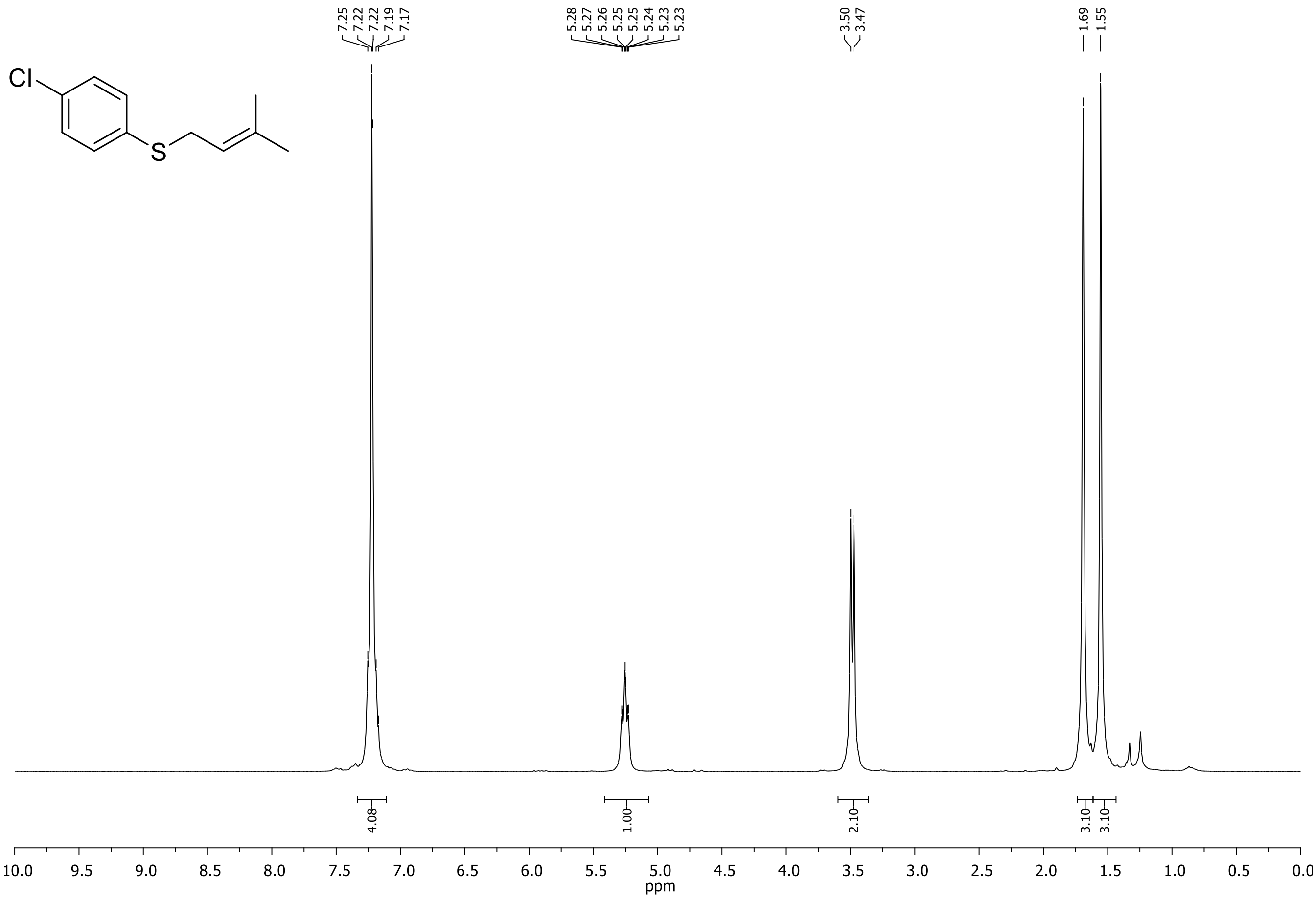

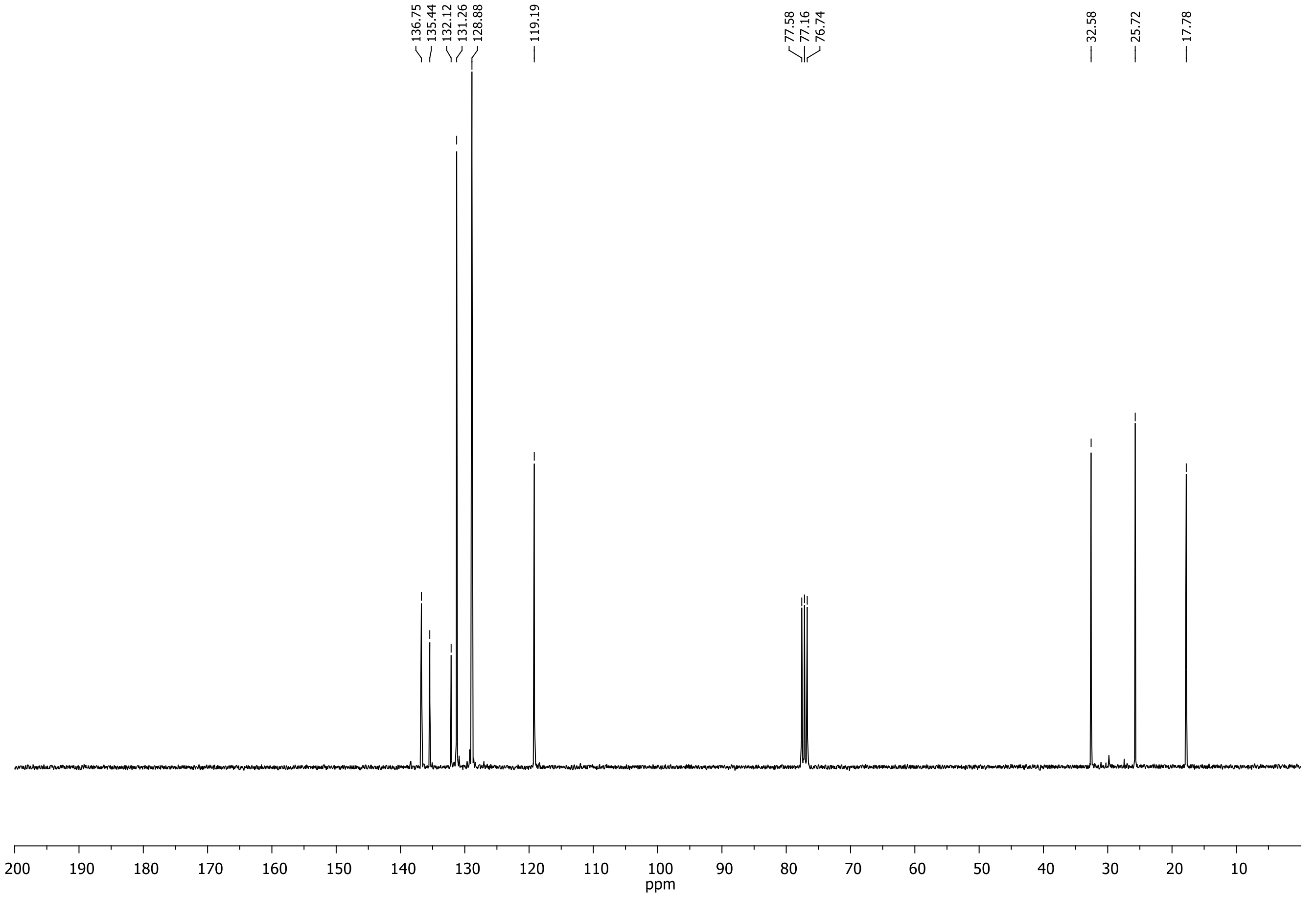

^{1}H-NMR (300.36 MHz, $CDCl_3$); ^{13}C-NMR: (75.53 MHz, $CDCl_3$)

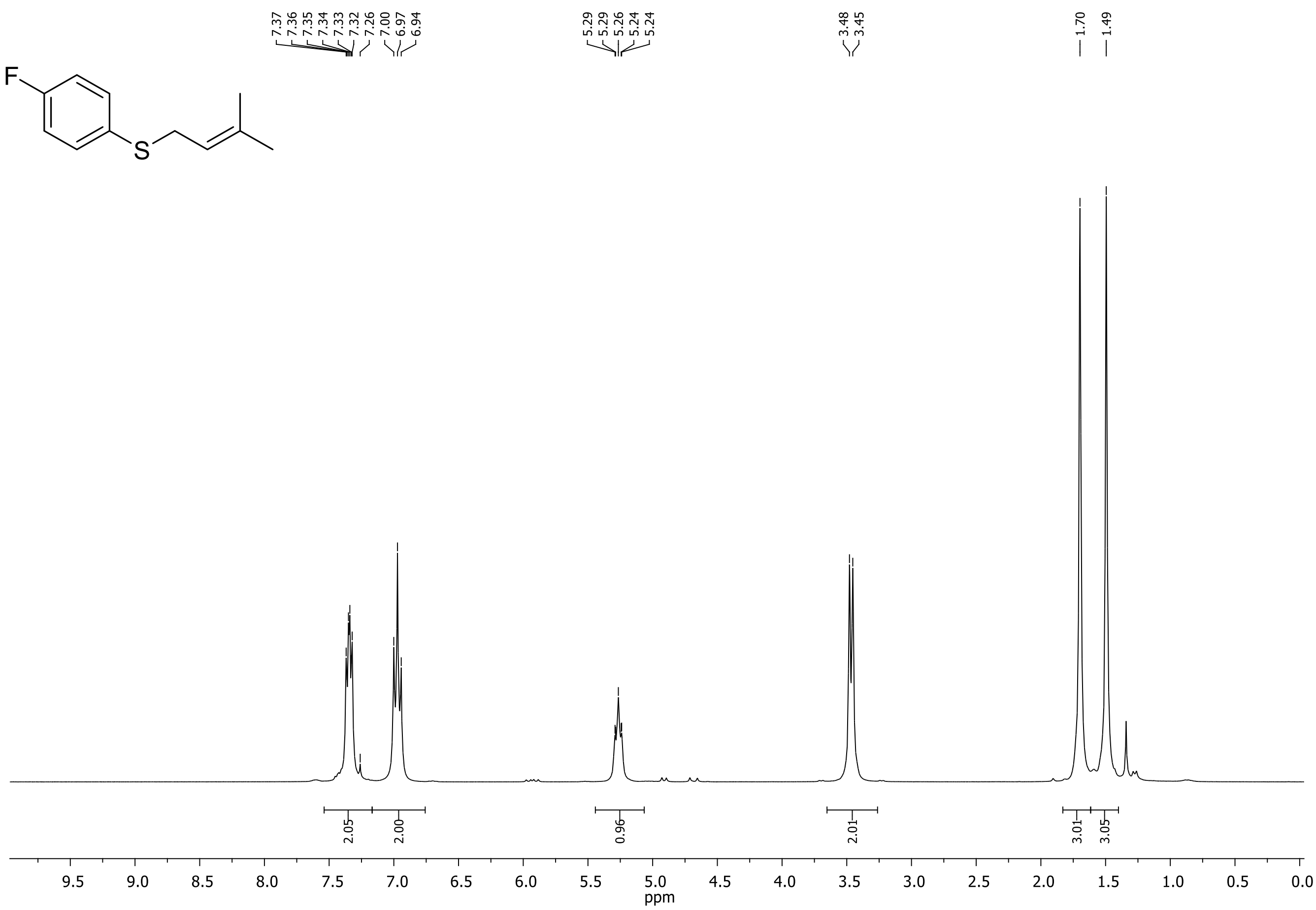

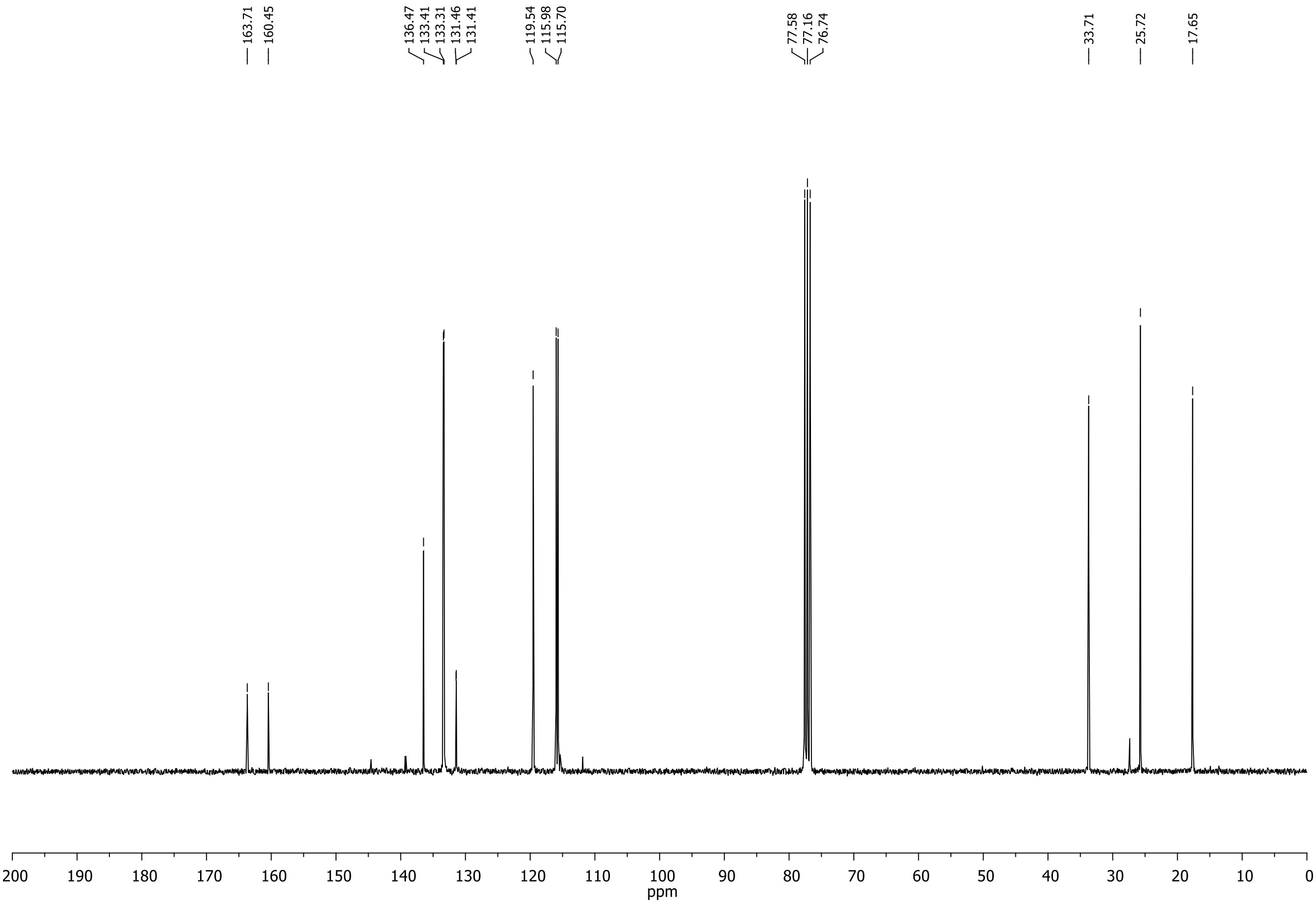

^{1}H-NMR (300.36 MHz, $CDCl_3$); ^{13}C-NMR: (75.53 MHz, $CDCl_3$)

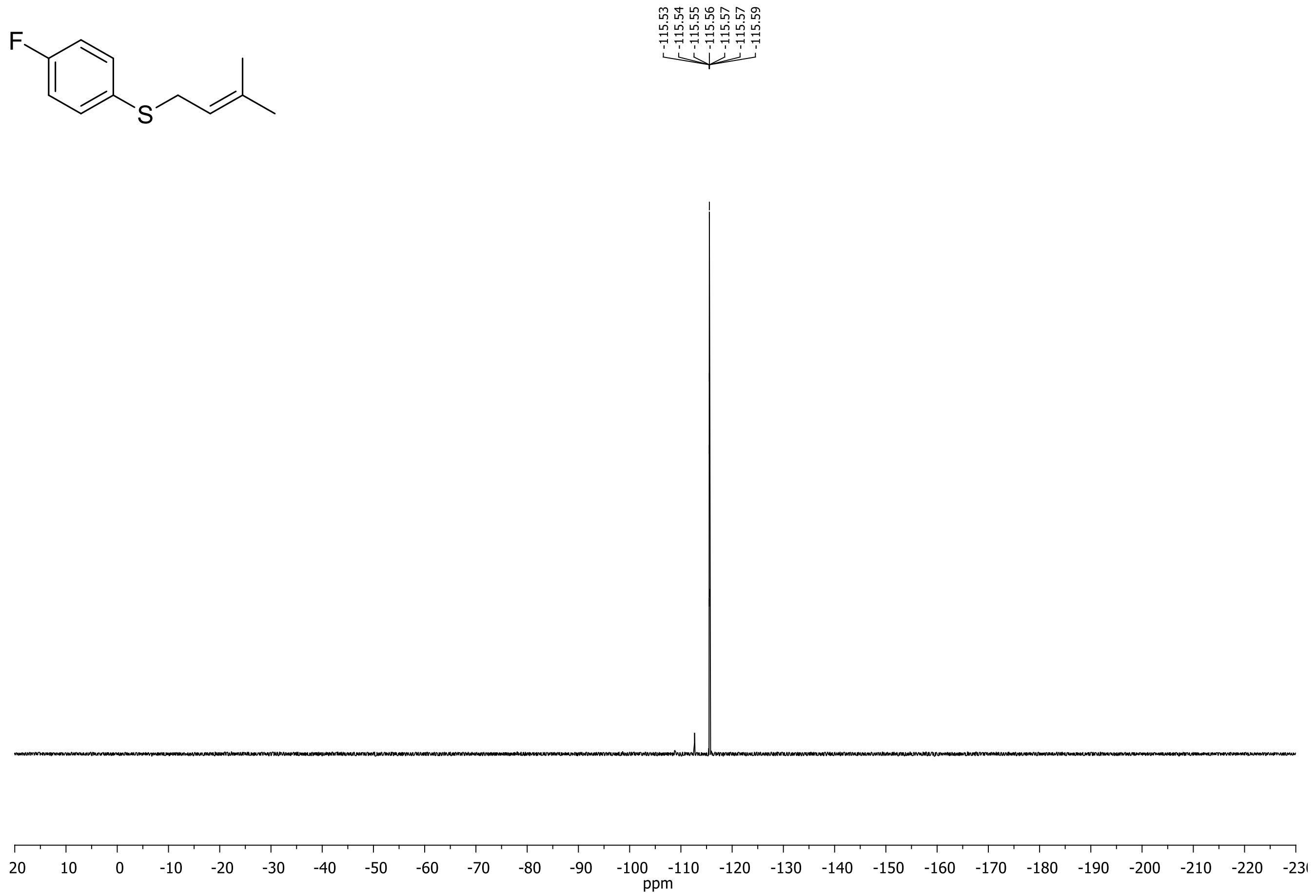

^{19}F-NMR (470 MHz, $CDCl_3$)

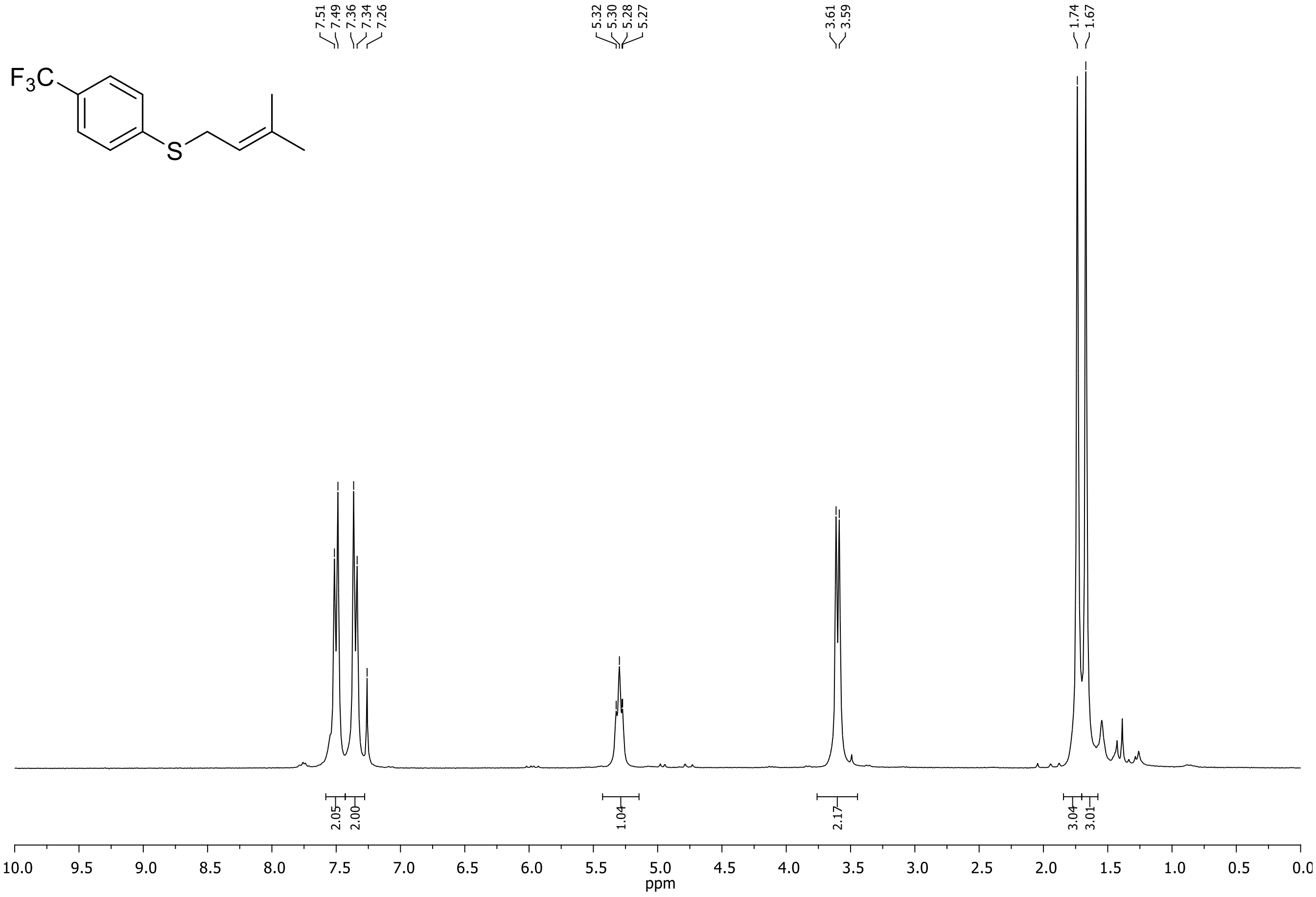

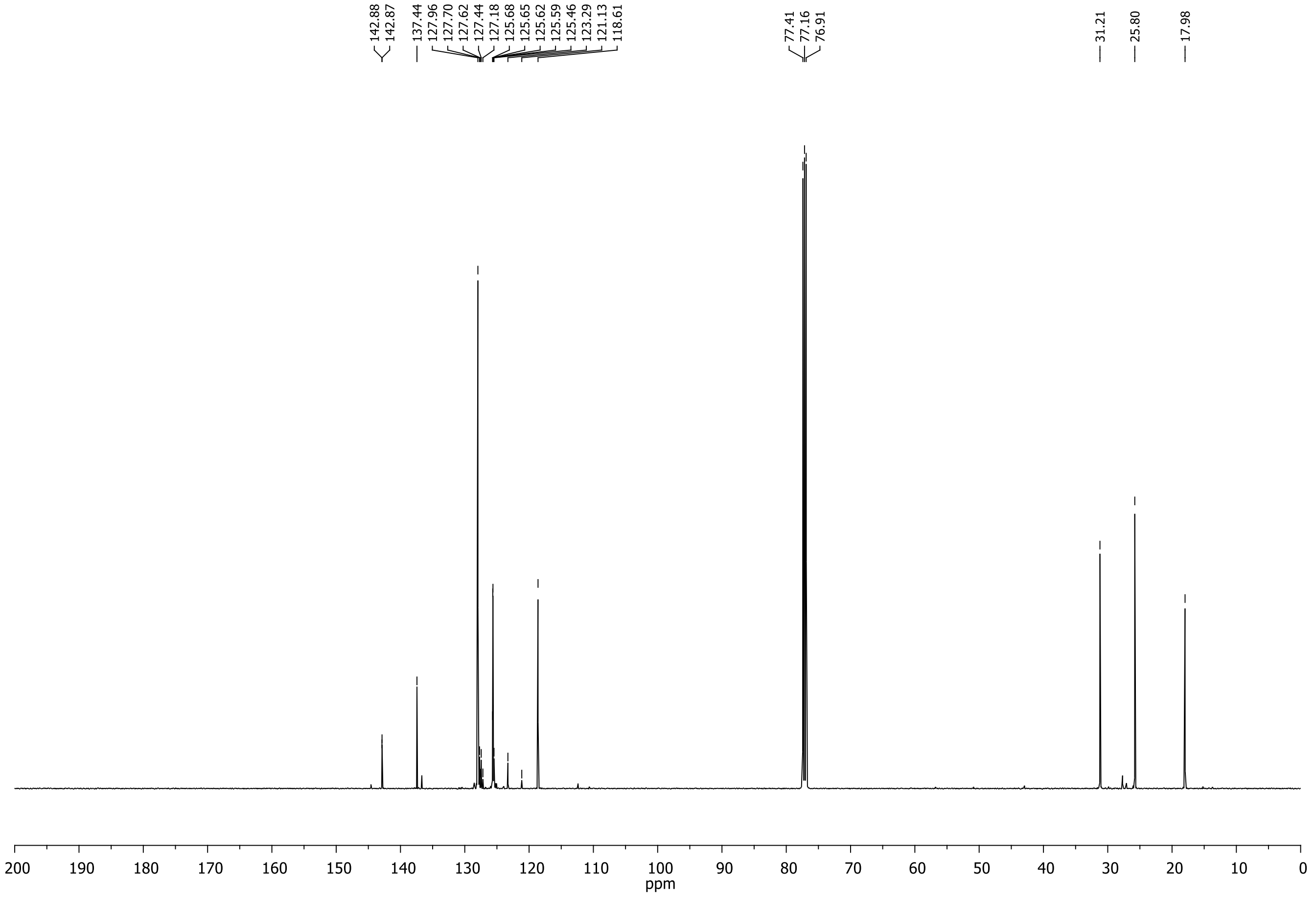

^{1}H-NMR (300 MHz, $CDCl_3$); ^{13}C-NMR: (126 MHz, $CDCl_3$)

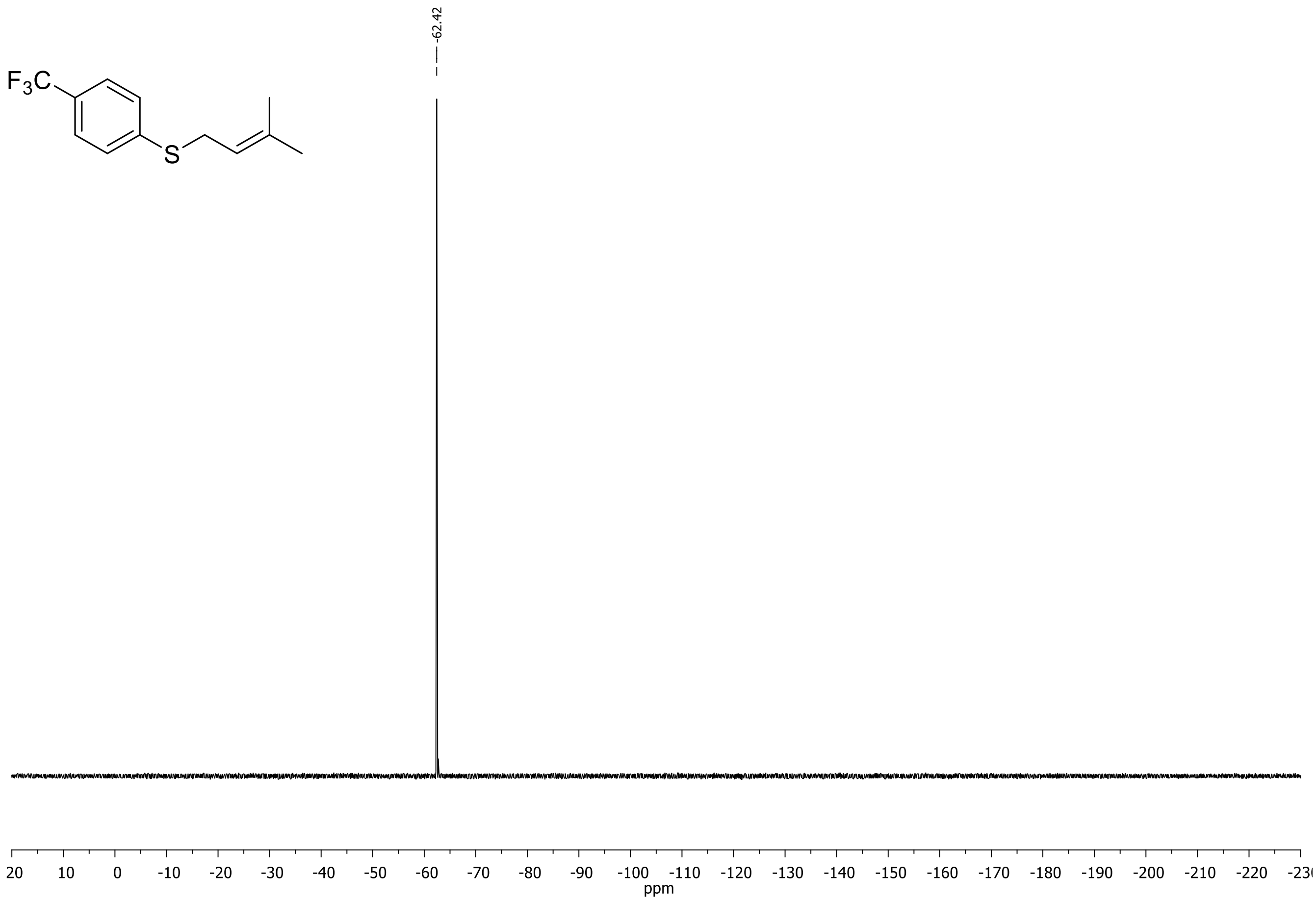

^{19}F-NMR (470 MHz, $CDCl_3$)

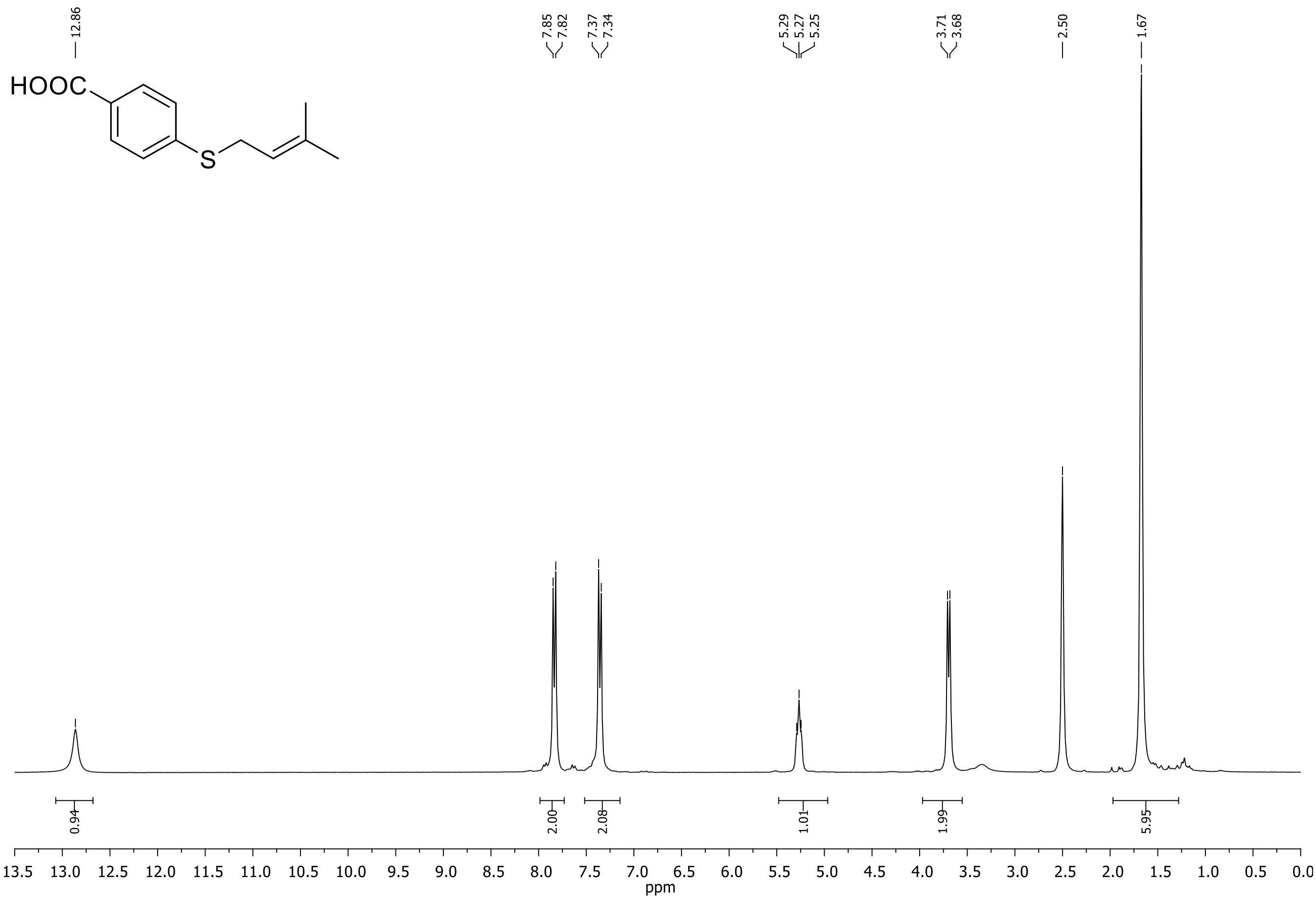

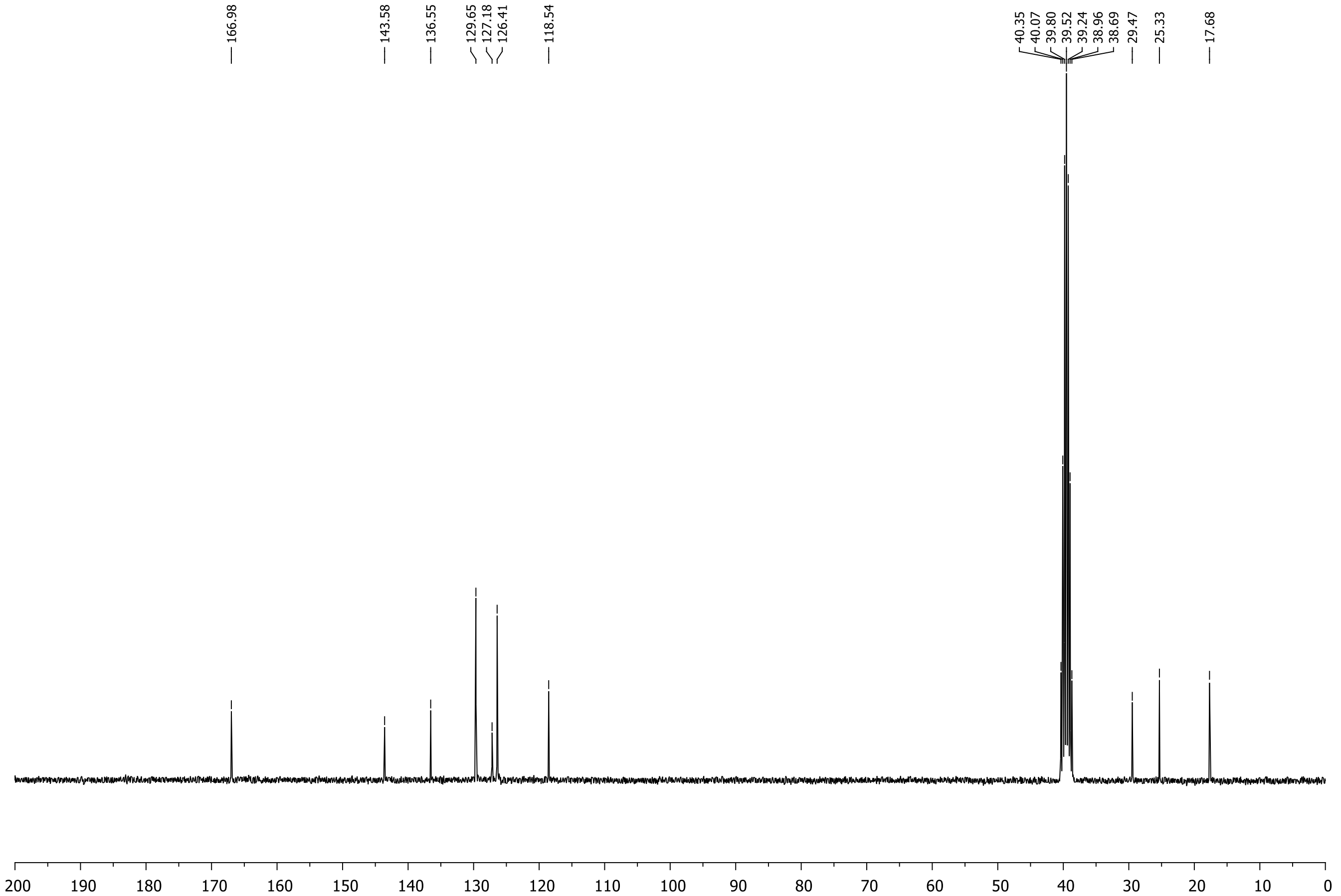

^{1}H-NMR (300.36 MHz, DMSO-d_6); ^{13}C-NMR: (75.53 MHz, DMSO-d_6)

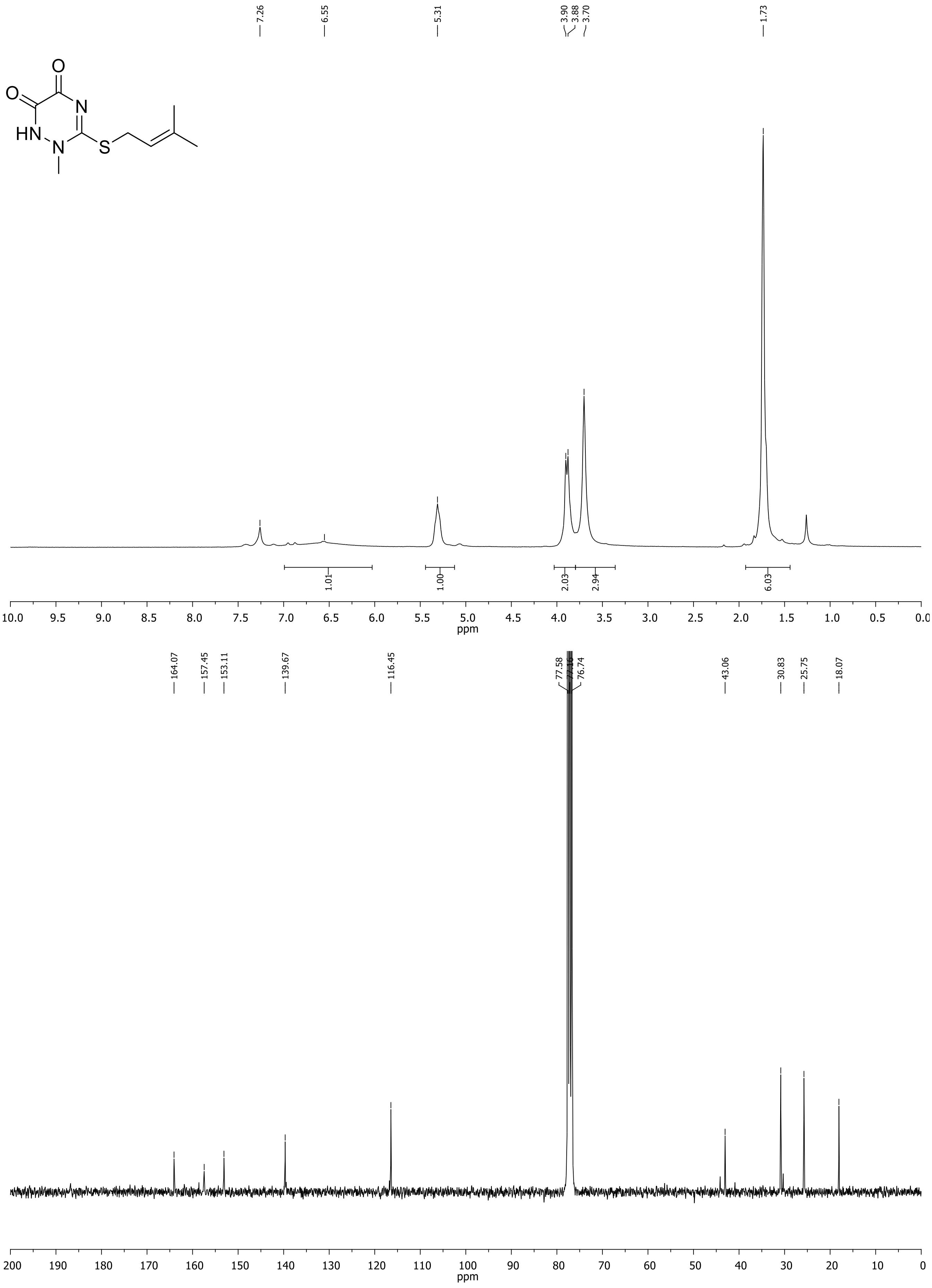

^{1}H-NMR (300.36 MHz, $CDCl_3$); ^{13}C-NMR: (75.53 MHz, $CDCl_3$)

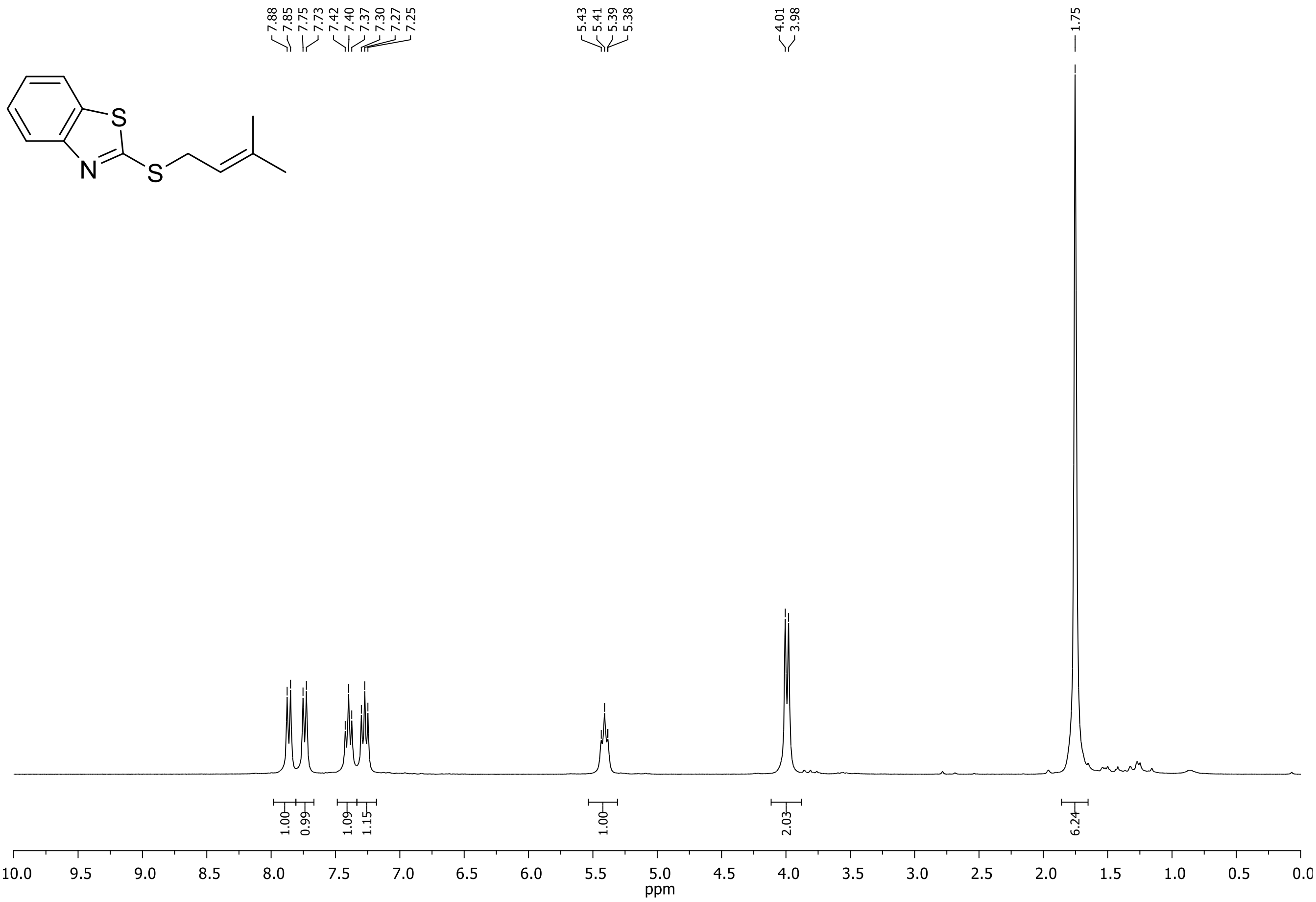

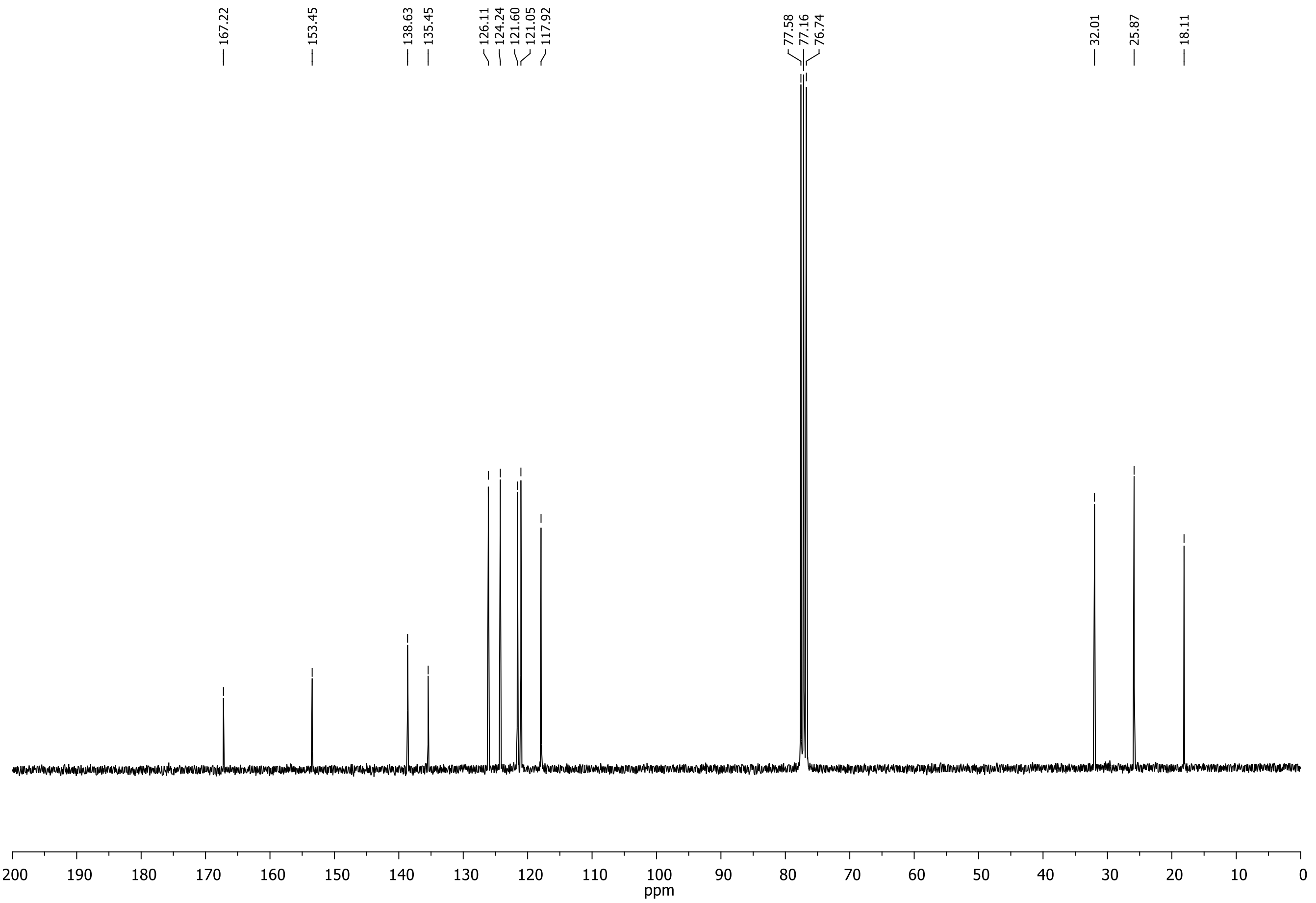

^{1}H-NMR (300.36 MHz, $CDCl_3$); ^{13}C-NMR: (75.53 MHz, $CDCl_3$)

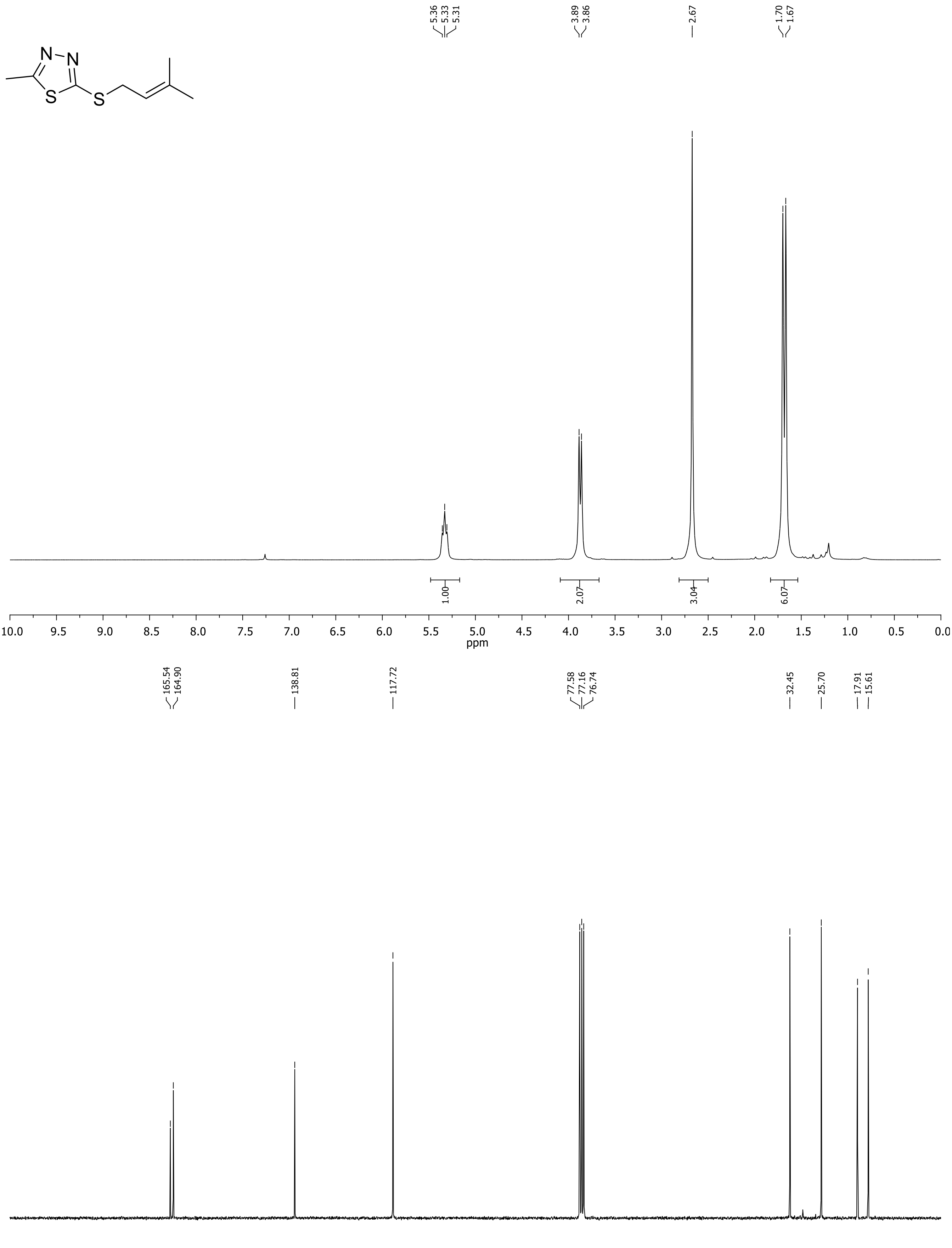

^{1}H-NMR (300.36 MHz, $CDCl_3$); ^{13}C-NMR: (75.53 MHz, $CDCl_3$)

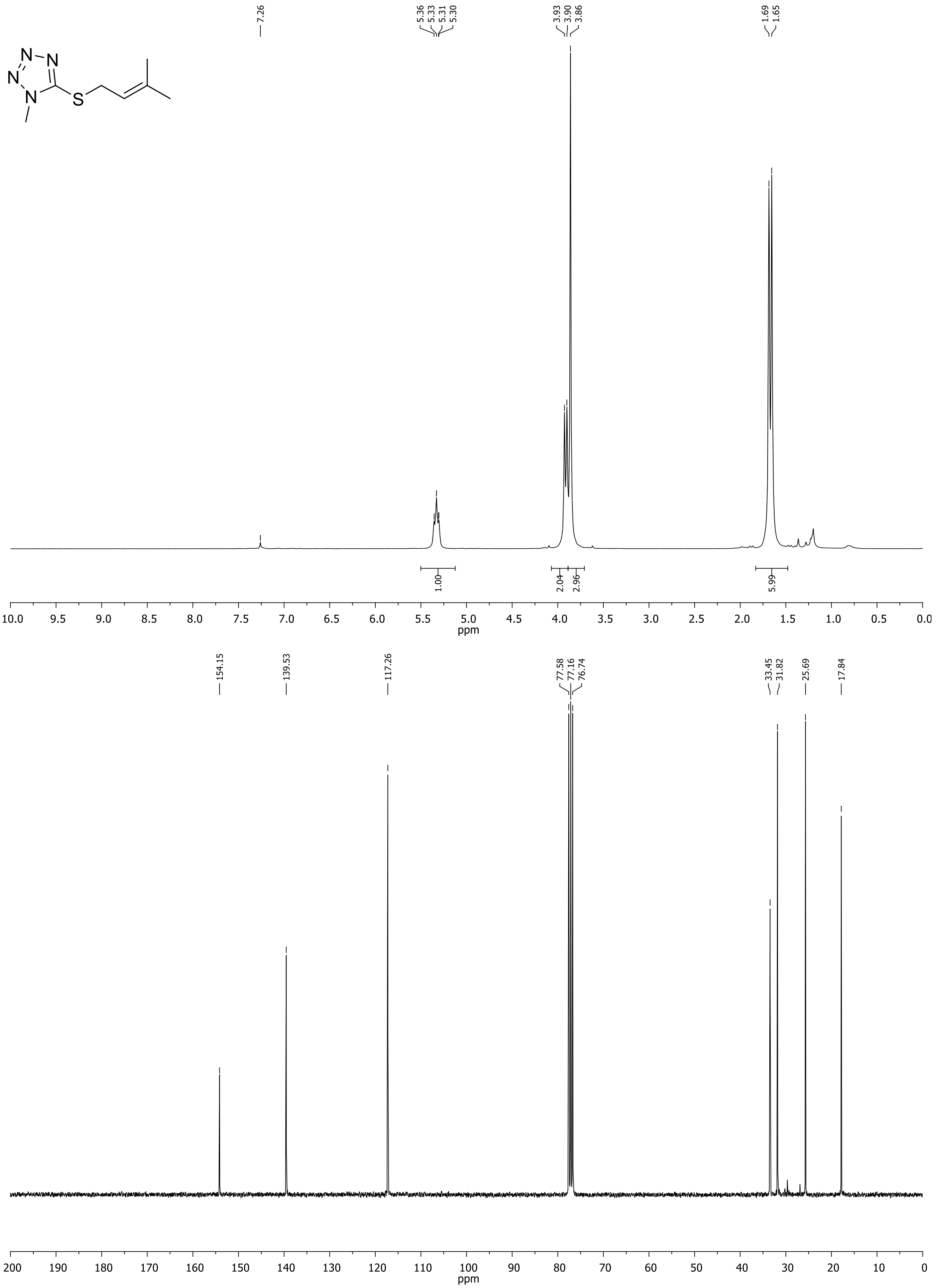

^{1}H-NMR (300.36 MHz, $CDCl_3$); ^{13}C-NMR: (75.53 MHz, $CDCl_3$)

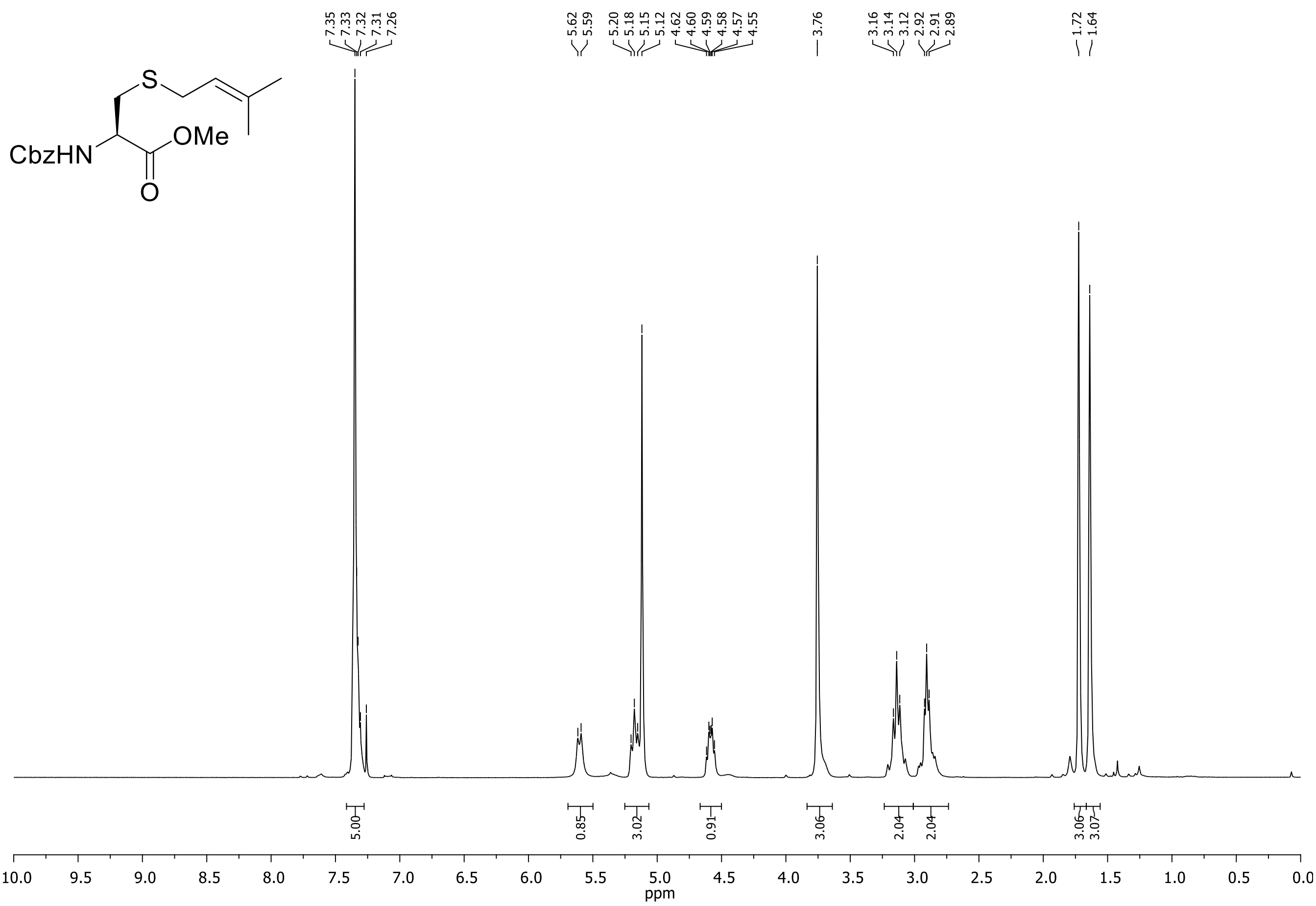

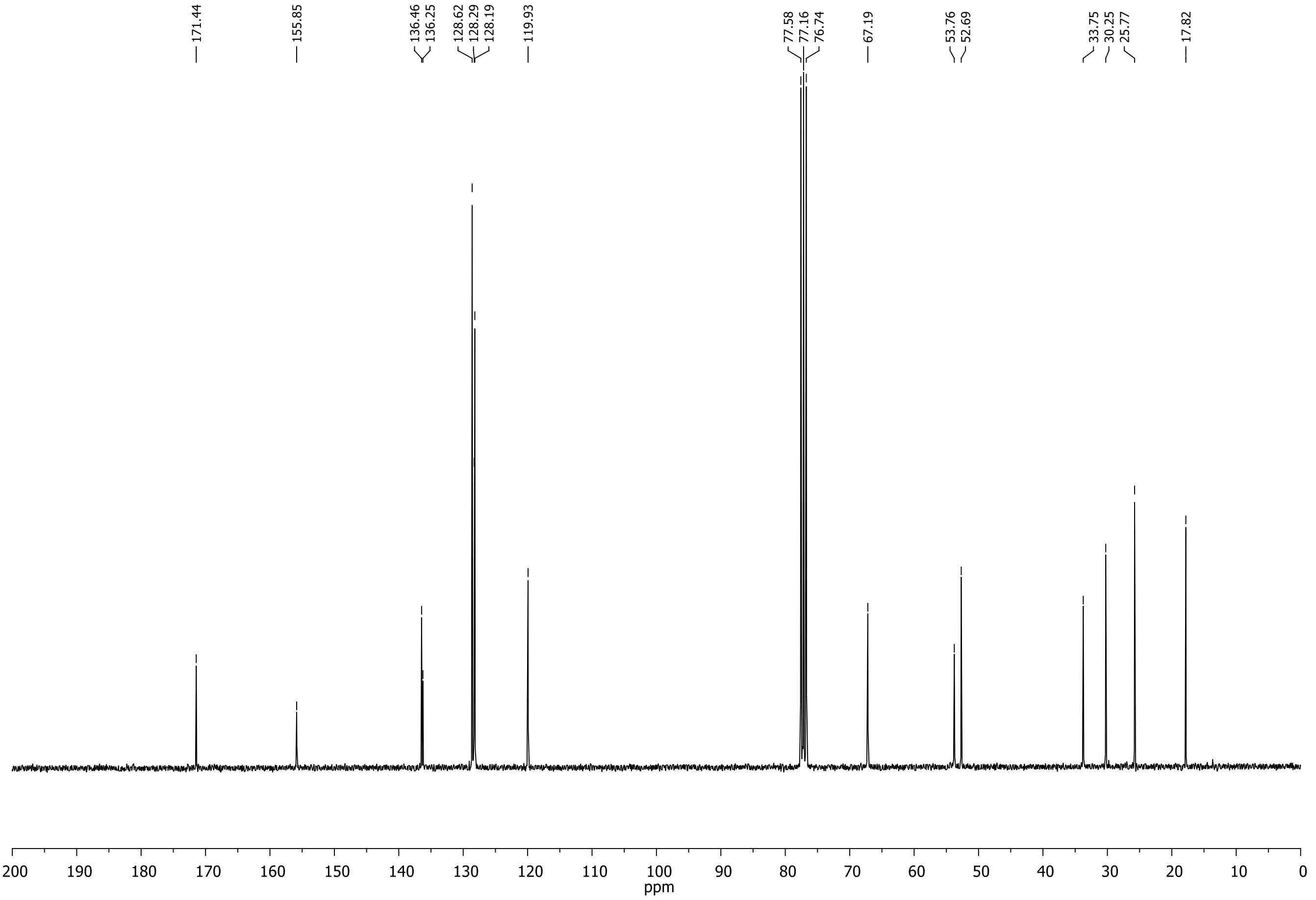

^{1}H-NMR (300.36 MHz, $CDCl_3$); ^{13}C-NMR: (75.53 MHz, $CDCl_3$)

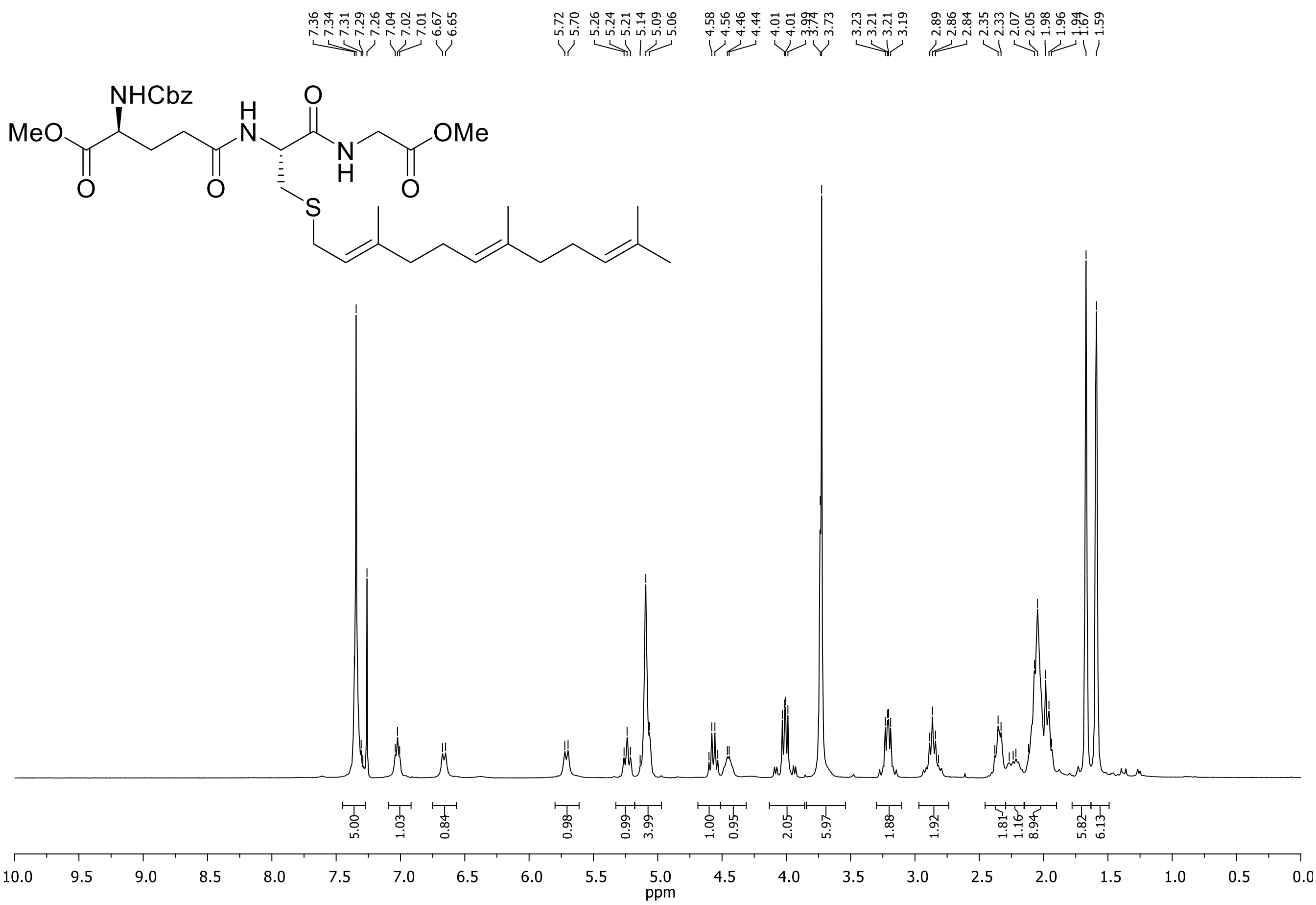

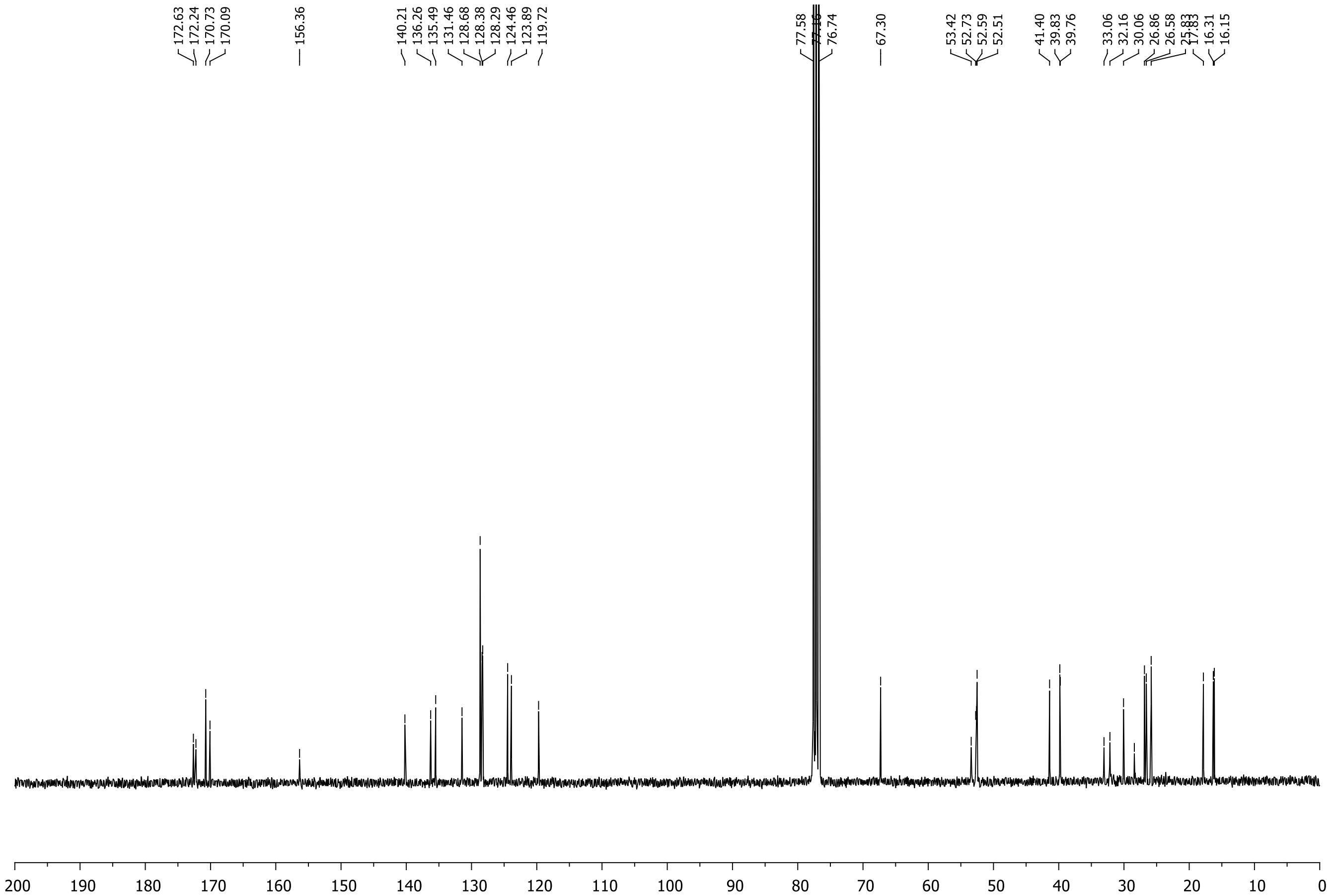

^{1}H-NMR (300.36 MHz, $CDCl_3$); ^{13}C-NMR: (75.53 MHz, $CDCl_3$)

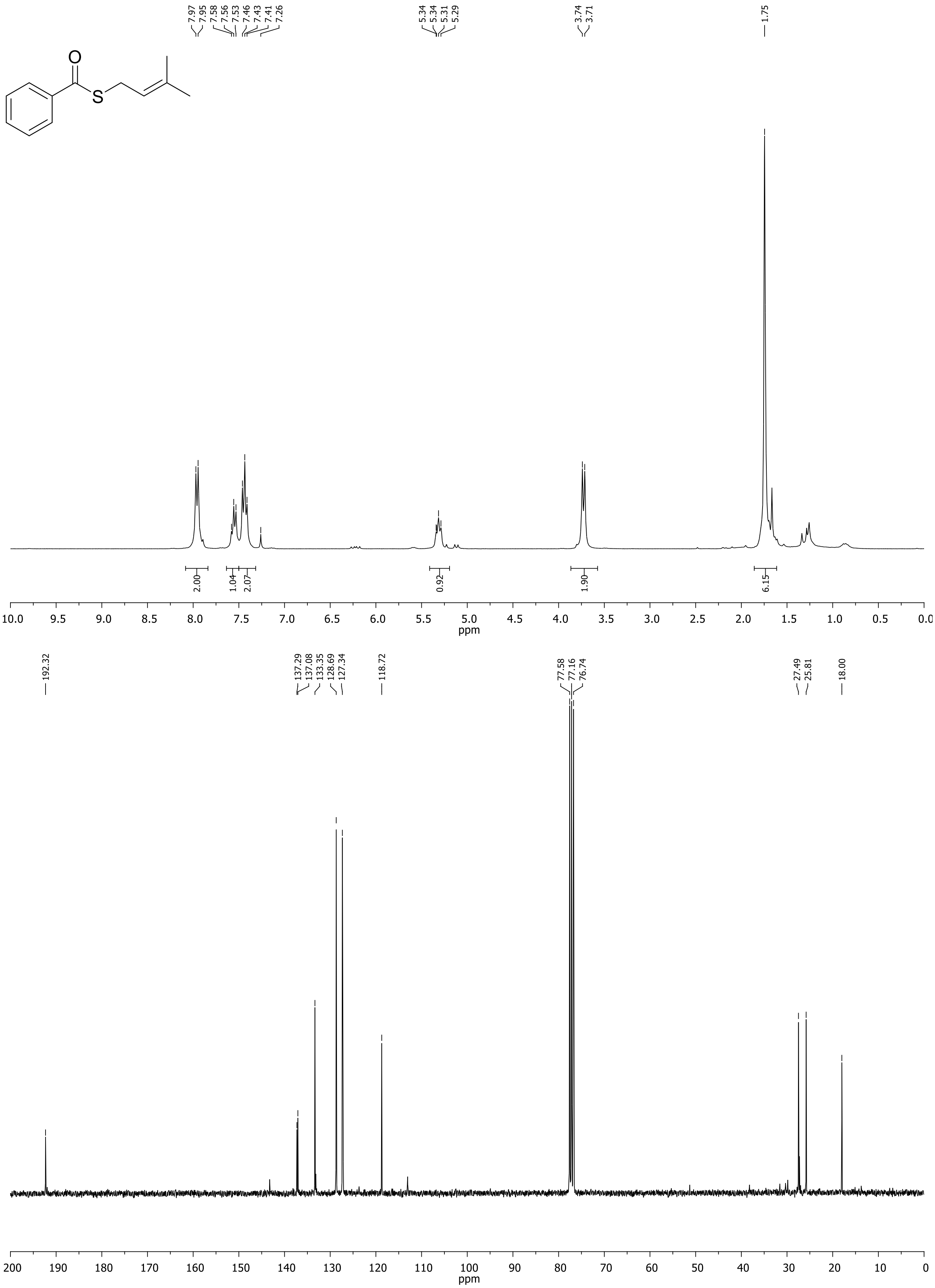

^{1}H-NMR (300.36 MHz, $CDCl_3$); ^{13}C-NMR: (75.53 MHz, $CDCl_3$)

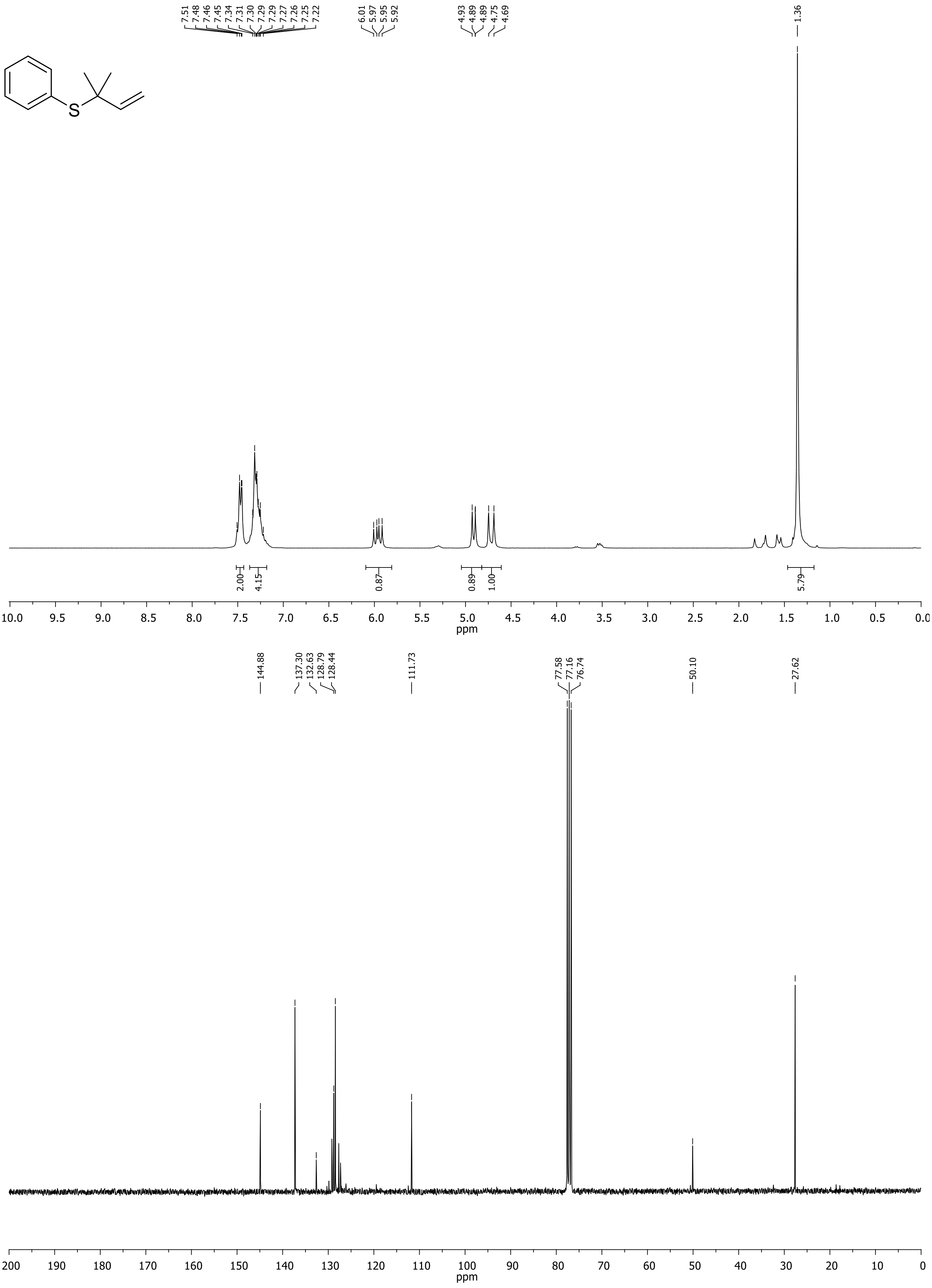

^{1}H-NMR (300.36 MHz, $CDCl_3$); ^{13}C-NMR: (75.53 MHz, $CDCl_3$)

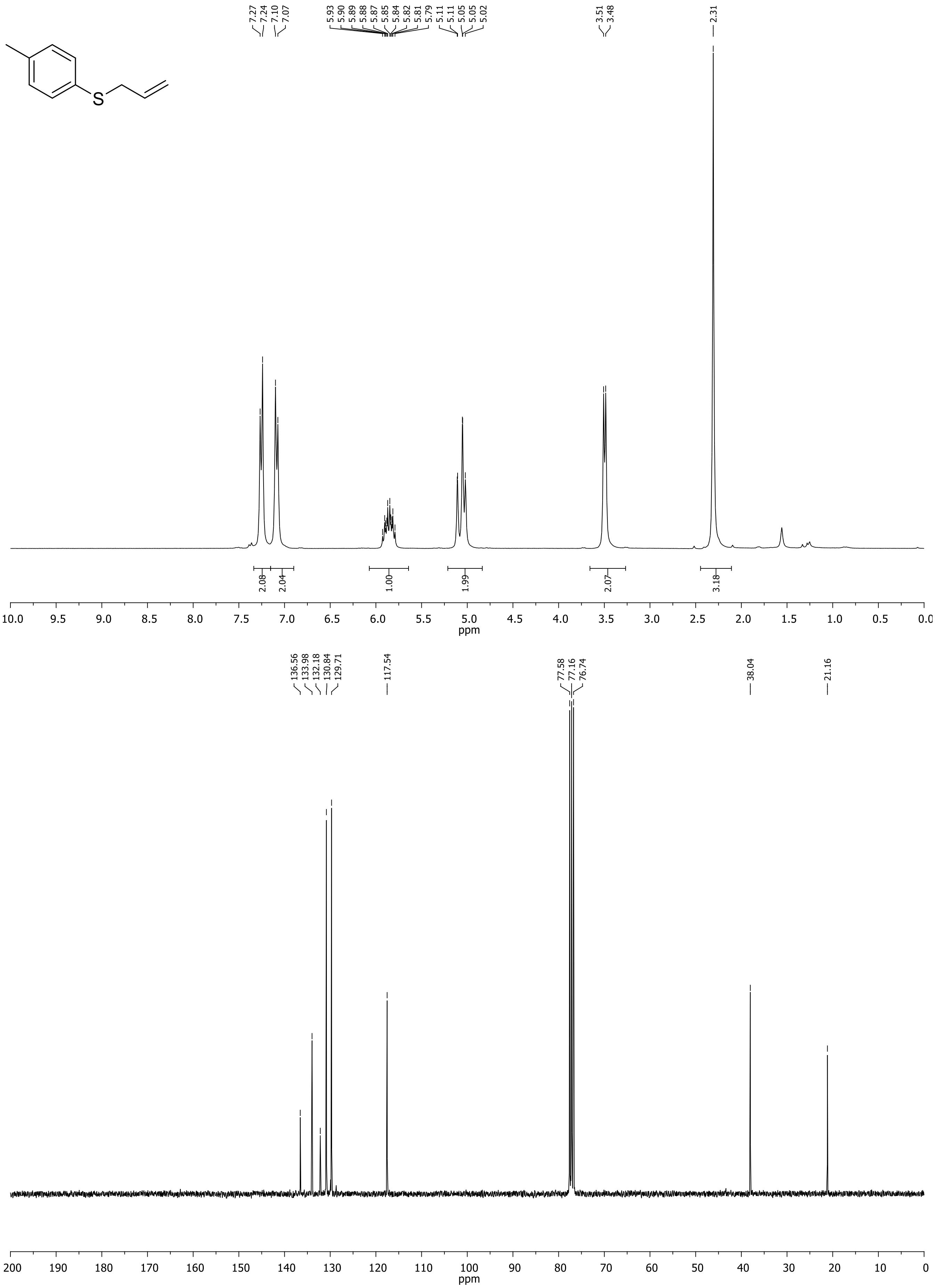

^{1}H-NMR (300.36 MHz, $CDCl_3$); ^{13}C-NMR: (75.53 MHz, $CDCl_3$)

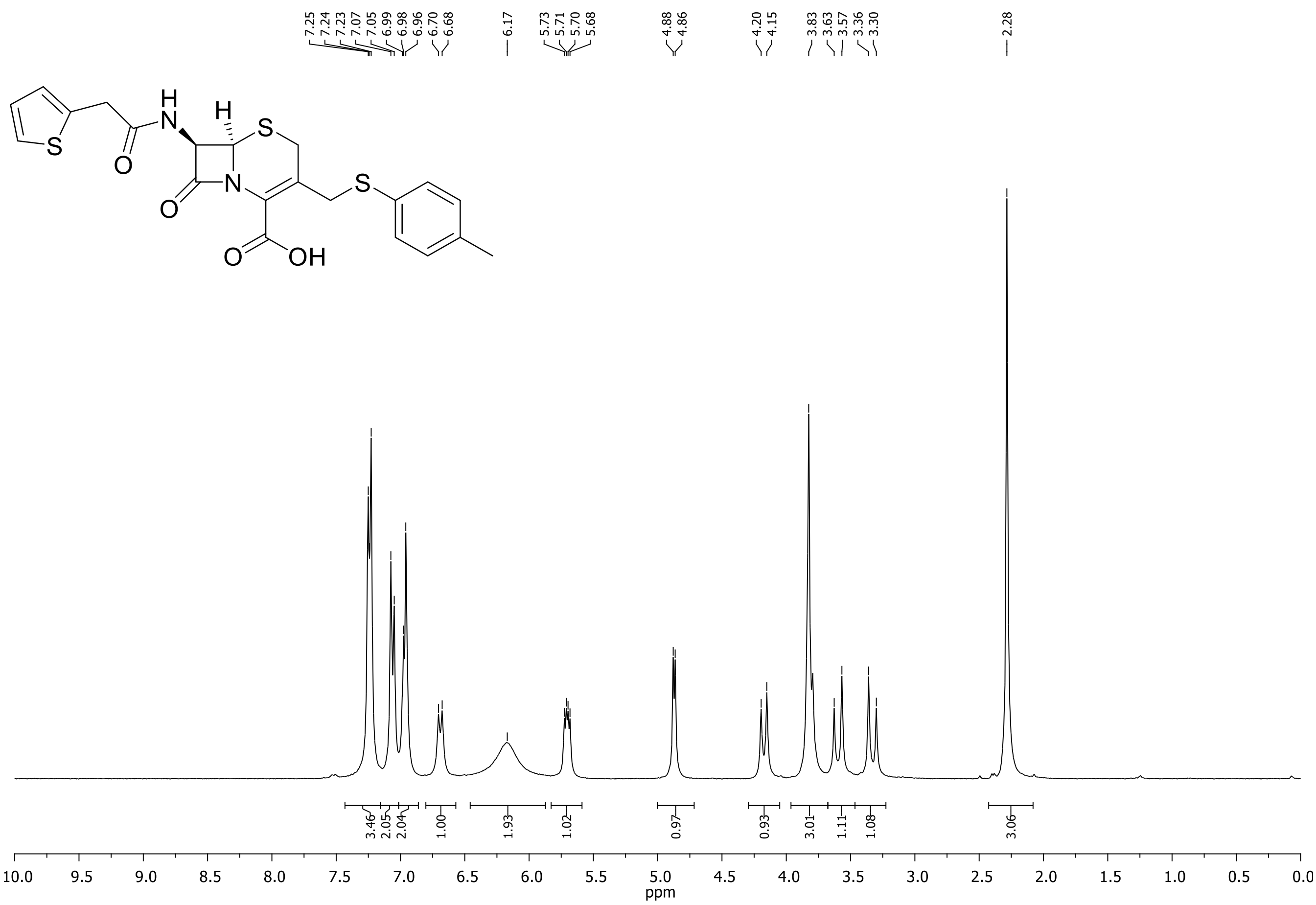

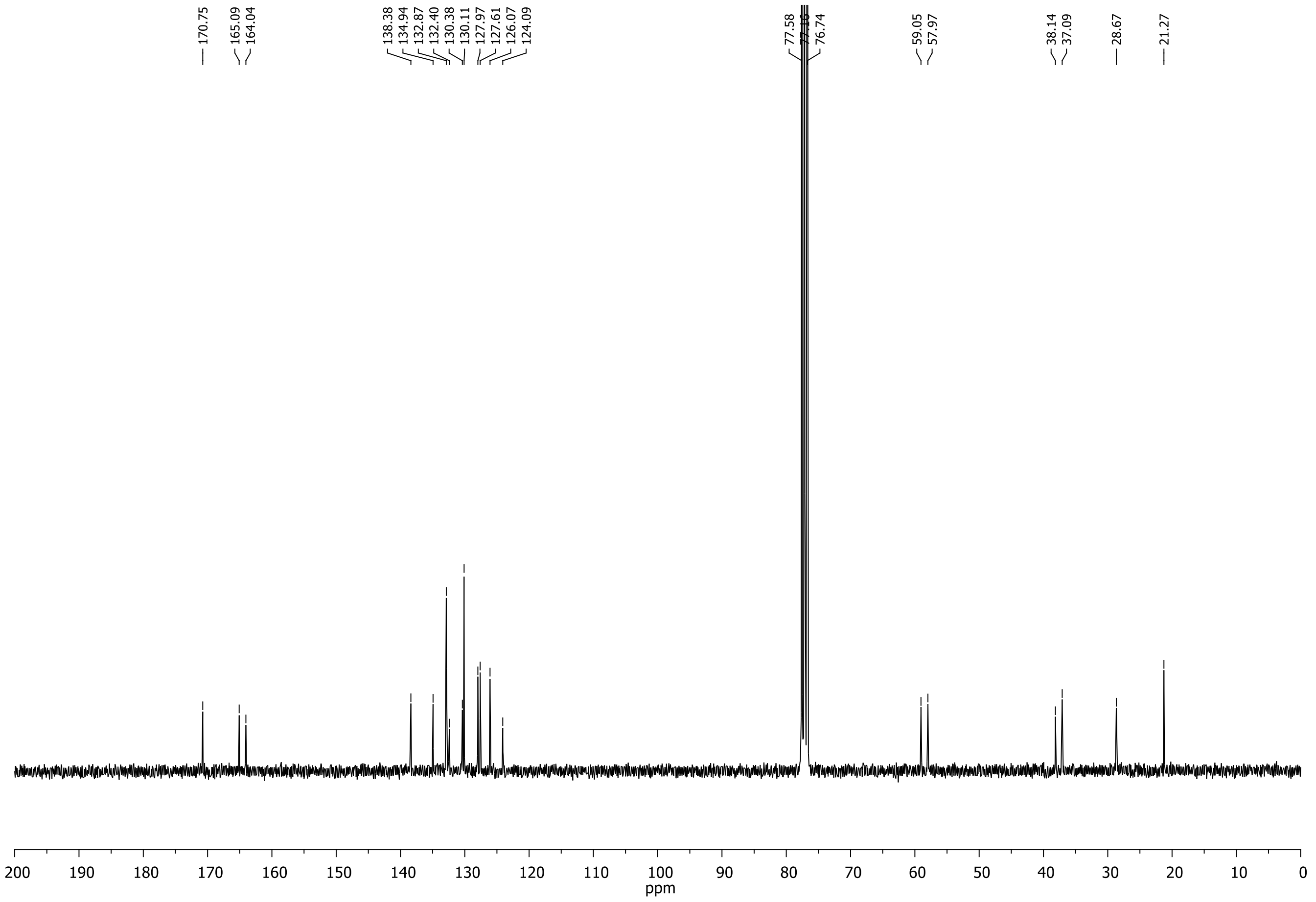

^{1}H-NMR (300.36 MHz, $CDCl_3$); ^{13}C-NMR: (75.53 MHz, $CDCl_3$)

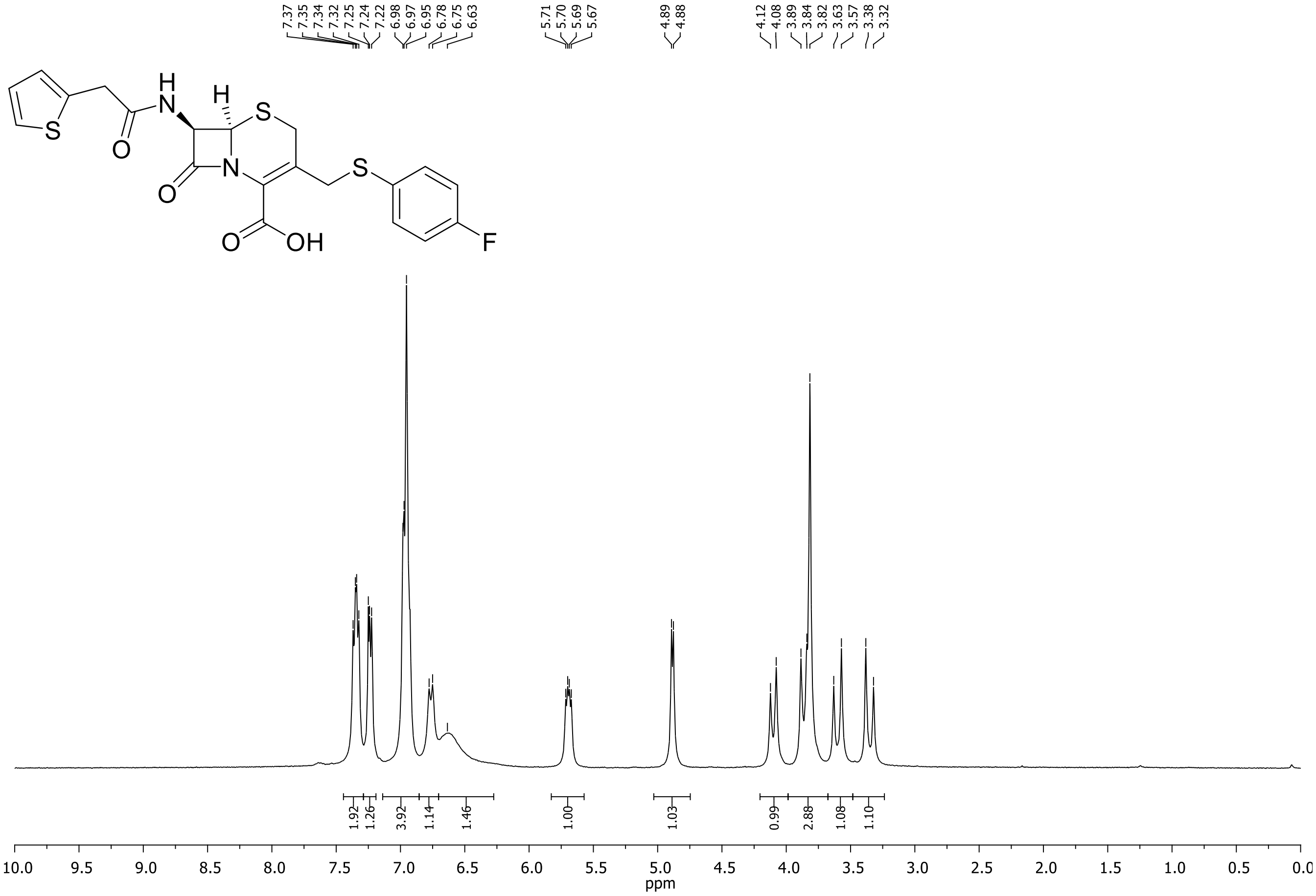

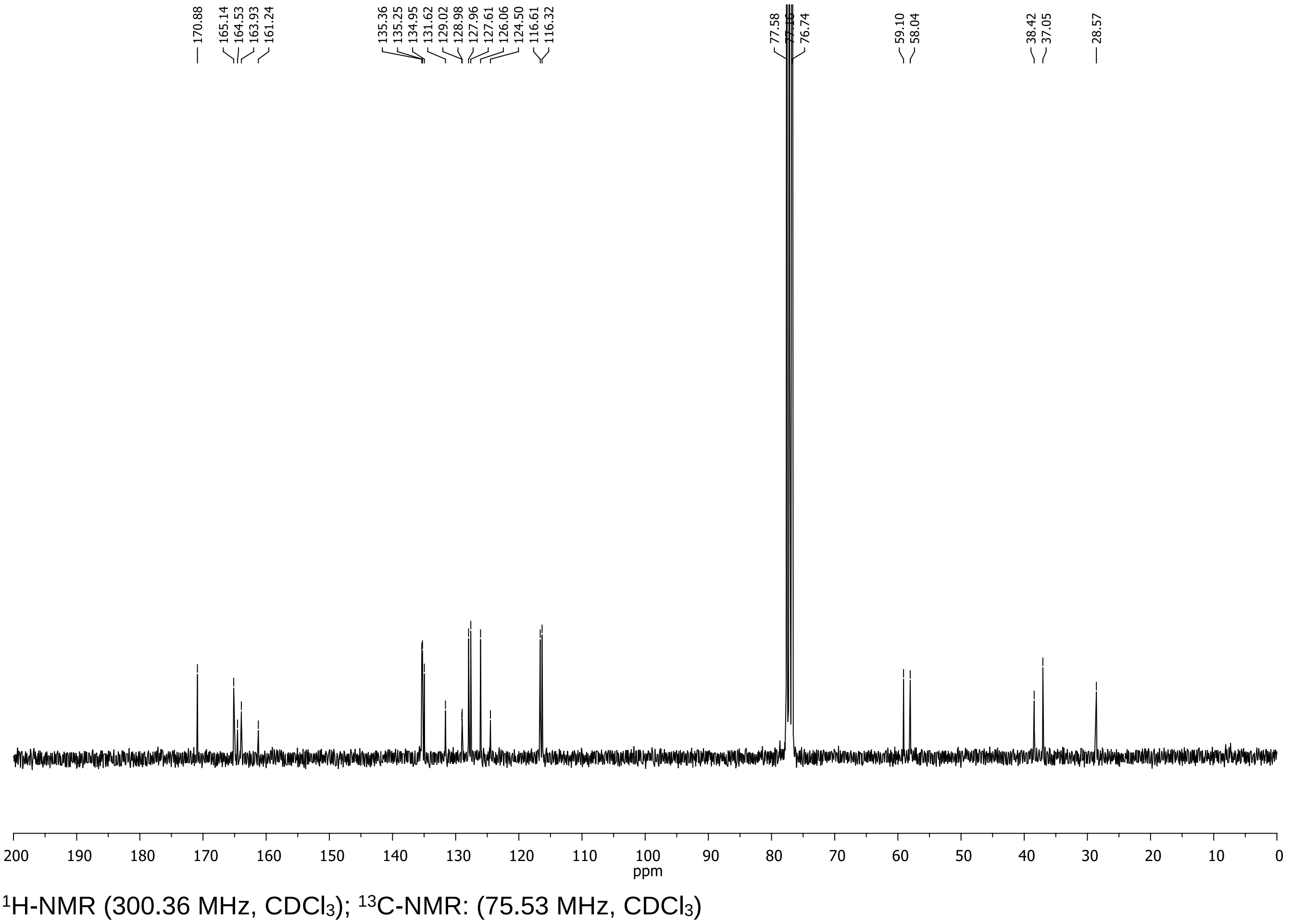

^{1}H-NMR (300.36 MHz, $CDCl_3$); ^{13}C-NMR: (75.53 MHz, $CDCl_3$)

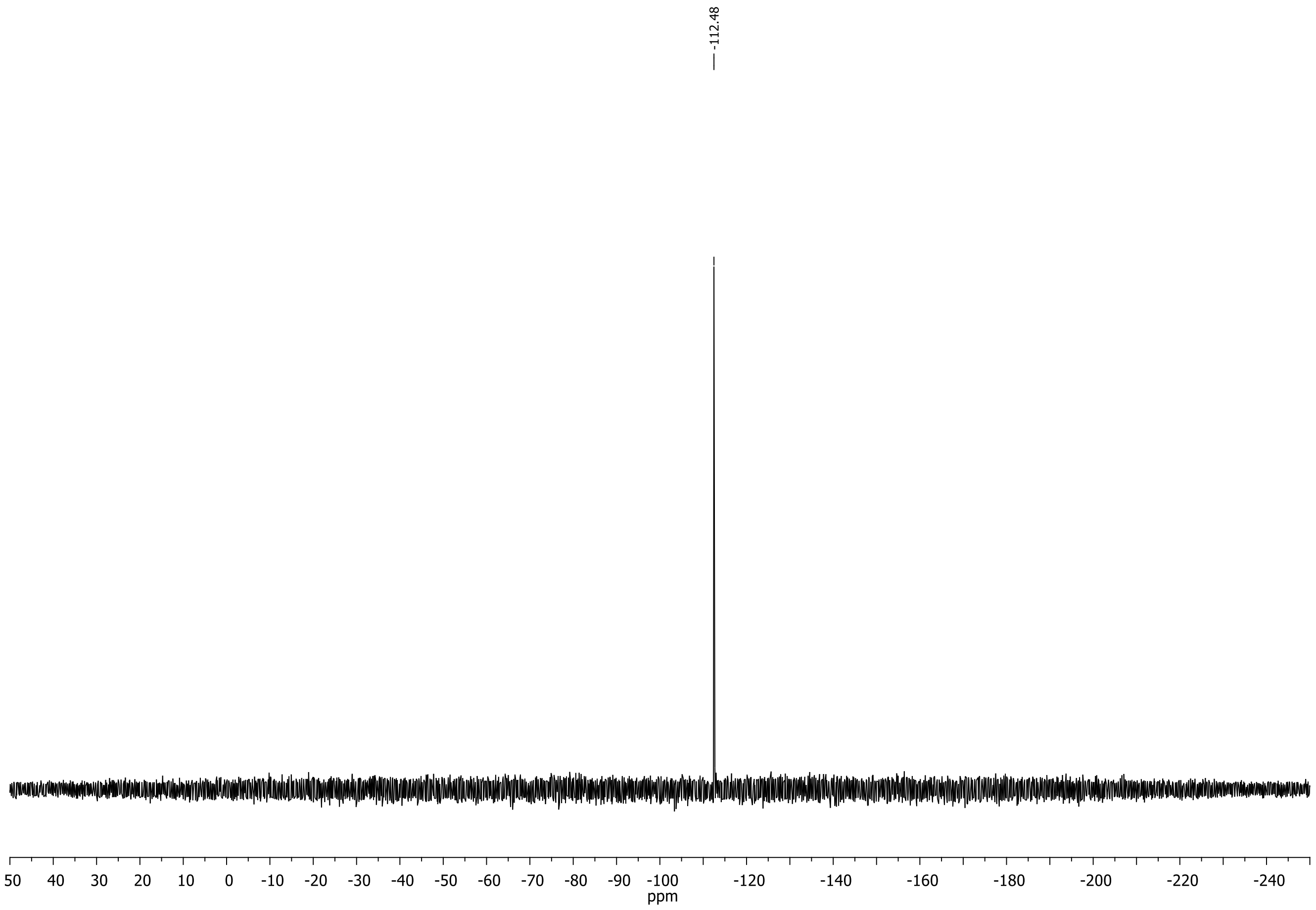

^{19}F-NMR (470 MHz, $CDCl_3$)

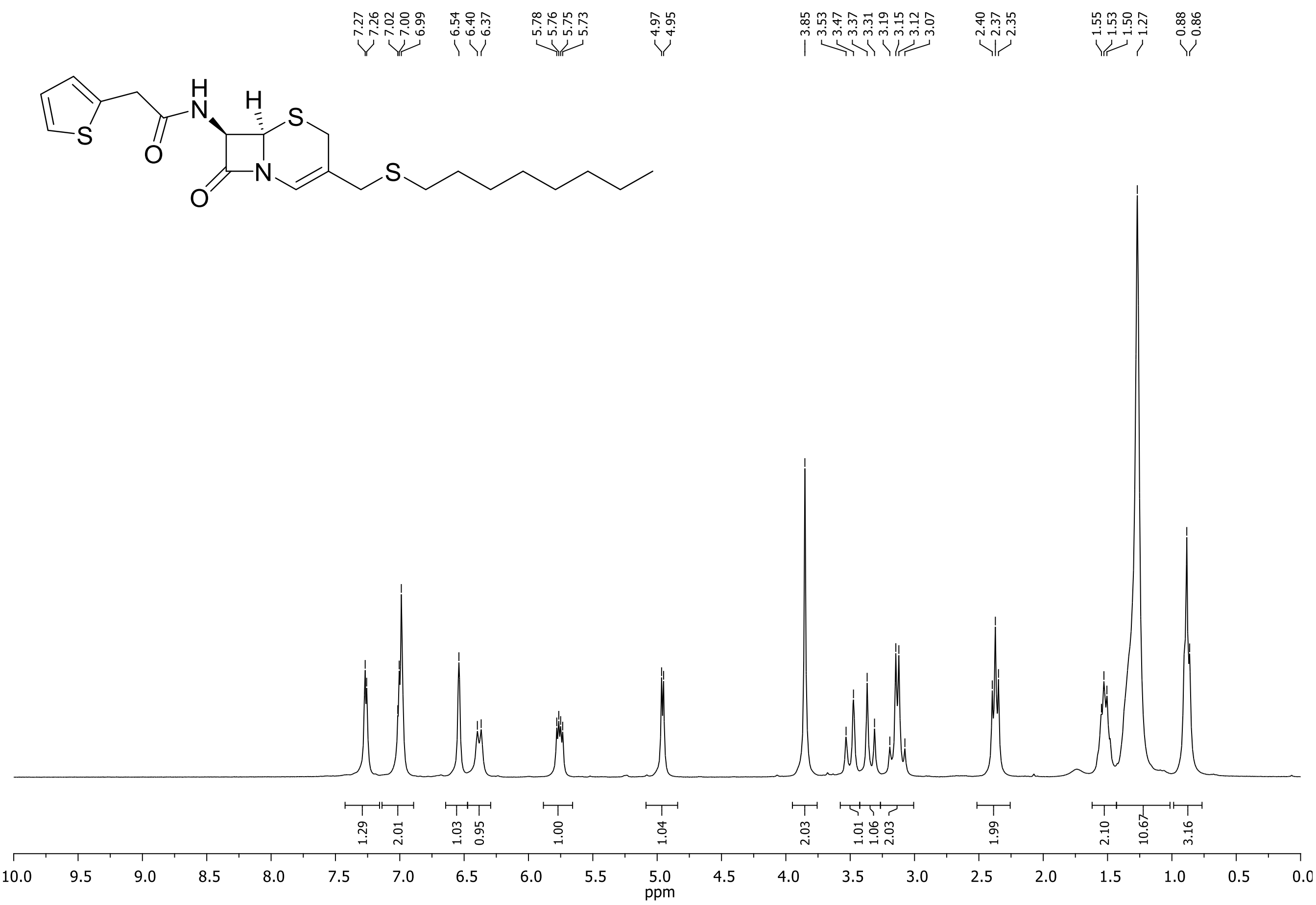

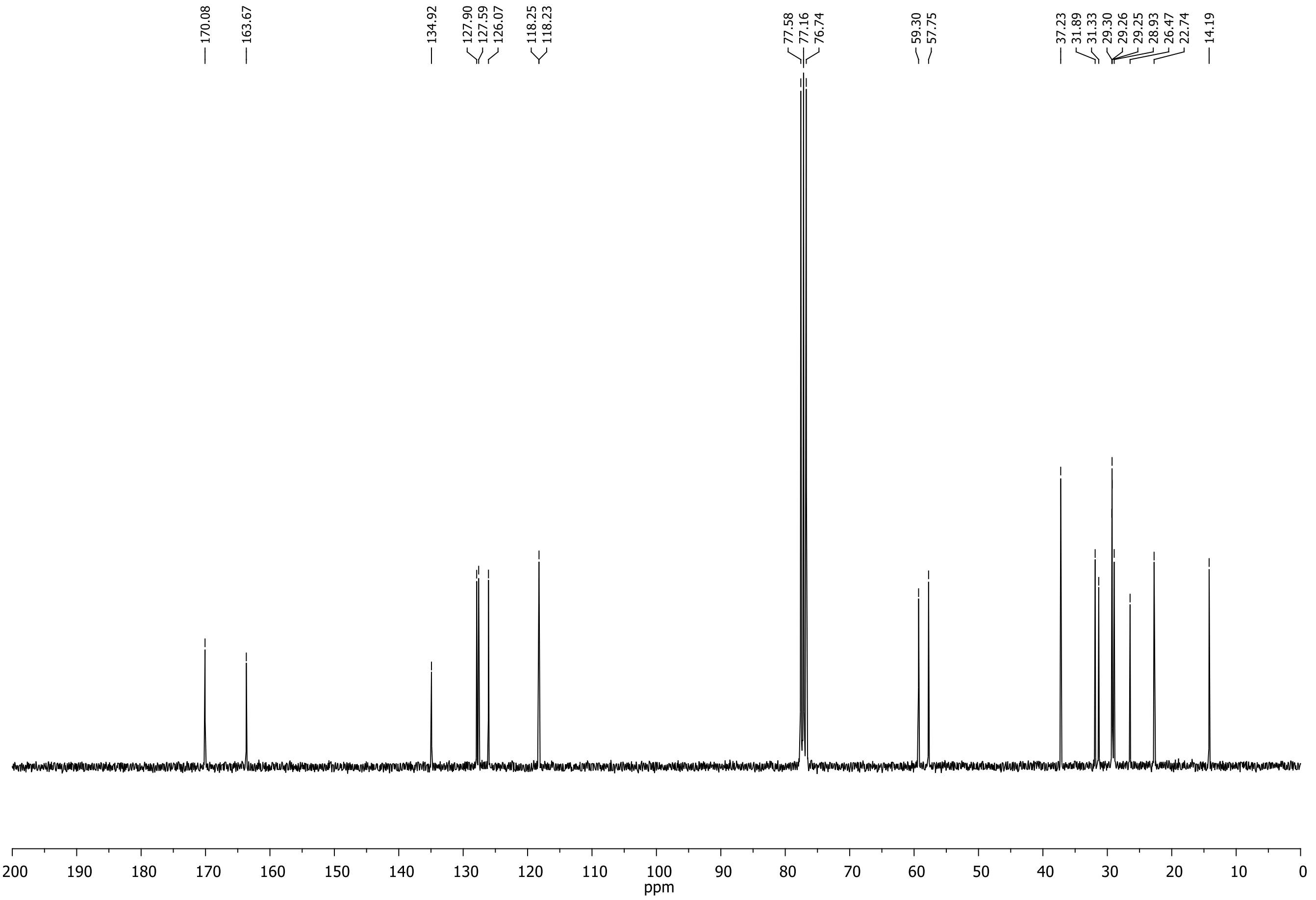

^{1}H-NMR (300.36 MHz, $CDCl_3$); ^{13}C-NMR: (75.53 MHz, $CDCl_3$)
